# Modular Rh‐Catalyzed Synthesis and Biological Profiling of Diverse Pentafluorobenzenesulfonamide Reactive Fragments

**DOI:** 10.1002/chem.202600009

**Published:** 2026-04-10

**Authors:** Julian Chesti, Jennifer A. Miles, George J. Wade, Scott Grossman, George W. Preston, Richard Bayliss, Stuart L. Warriner, Megan H. Wright, Adam Nelson

**Affiliations:** ^1^ School of Chemistry University of Leeds Leeds UK; ^2^ Astbury Centre for Structural Molecular Biology University of Leeds Leeds UK; ^3^ School of Molecular and Cellular Biology University of Leeds Leeds UK

**Keywords:** covalent probes, protein modification, reactive fragments, rhodium nitrenoids

## Abstract

Covalent protein modification can facilitate the elucidation of biological mechanisms and serve as a powerful strategy for drug discovery. The discovery of protein modifiers may be initiated by screens of diverse sets of reactive fragments. A modular synthesis of diverse pentafluorobenzenesulfonamide reactive fragments was developed, which was based on the reaction between diverse, readily‐available substrates and rhodium nitrenoid intermediates formed from pentafluorobenzenesulfonamide. The approach enabled the synthesis of 21 reactive fragments whose diversity stemmed from both that of the substrates and the rich chemistry of rhodium nitrenoids. The fragments were found to modify Aurora A kinase via several distinct pathways. Furthermore, five of the reactive fragments were profiled against HeLa cell lysate, which demonstrated that the constellation of modified cysteines was critically dependent on the specific reactive fragment used. Overall, the modular Rh‐catalyzed connective approach enabled the synthesis of reactive fragments with high structural and reactivity diversity. We envisage that such reactive fragments may provide useful starting points for the discovery of covalent modifiers of proteins, including chemical probes and drugs.

## Graphical abstract

A modular Rh‐catalyzed synthetic approach enabled the preparation of diverse reactive fragments from available substrates. The reactive fragments modified Aurora A kinase via several distinct modification reaction classes. In a complex biological environment, the constellation of modified proteins depended critically on the reactive fragment structure. The approach may provide useful starting points for the discovery of covalent chemical probes and drugs.

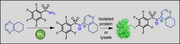

## 1 Introduction

Covalent modifiers of proteins can serve as useful chemical tools for interrogating biological mechanisms [1, 2]. Such chemical tools can enable investigation of the engagement of bioactive molecules with proteins in a cellular context [3, 4, 5, 6, 7, 8]. Furthermore, covalent drug discovery has enjoyed a recent resurgence that has led to FDA‐approved (US Food and Drug Administration‐approved) drugs in therapeutic areas including cancer and virology [2, 9, 10]. There are broadly two approaches to the discovery of high‐quality chemical tools for targeted protein modification [11, 12, 13]. Specifically, either an electrophile may be appended to an already‐optimized reversible ligand (“ligand first”), or a reactive fragment hit may be optimized using a fragment‐based discovery approach (“electrophile first”). These approaches have, respectively, broadly underpinned the discovery of ibrutinib [14] (that targets Bruton Tyrosine Kinase) and sotorasib [15] (that targets the G12C variant of K‐Ras [16]).

The structural diversity of reactive fragment sets, as explored in chemical space generally [17], is limited by the diversity of available building blocks and the reactions that are used to connect and functionalize them. Established sets [12, 18] have generally been prepared using robust reactions drawn from the narrow toolkit [19, 20] that dominates molecular discovery. The “direct‐to‐biology” discovery approach has recently emerged in which arrays of reactive fragments are prepared, usually by amide formation, and then, without purification, screened for protein modification [21, 22, 23, 24, 25, 26]. In addition, S(VI) exchange chemistry can enable the synthesis of diverse reactive fragments with complementary warheads [27].

**FIGURE 1:**
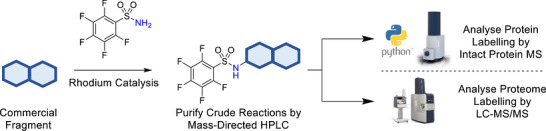
Envisaged approach for the synthesis and biological evaluation of pentafluorobenzenesulfonamide reactive fragments.

In this paper, we report the development of a modular synthesis of diverse pentafluorobenzenesulfonamides that harnessed a single set of reaction conditions. Fluorinated (het)aryl reagents can participate in nucleophilic aromatic substitution reactions, and have been exploited to target cysteine residues within proteins [26, 28, 29]. Furthermore, the reactivity of fluorinated aromatic probes may be rationally tuned [28, 29]. We envisaged that rhodium nitrenoid intermediates [30, 31, 32], formed from pentafluorobenzenesulfonamide itself [33], would have rich reactivity that would enable direct reaction with diverse substrates (e.g., by C−H insertion or aziridination) (Figure 1). The approach would therefore not be reliant on the availability of amine‐functionalized building blocks. We have demonstrated that the modular approach enables the synthesis of diverse perfluorobenzenesulfonamides via several alternative reaction types. Furthermore, we have profiled the reactivity of the resulting reactive fragment set against a specific protein (Aurora A kinase) and in a complex biological environment (HeLa cell lysate).

## 2 Results

### 2.1 Development and Execution of a Modular Connective Synthetic Approach

**FIGURE 2:**
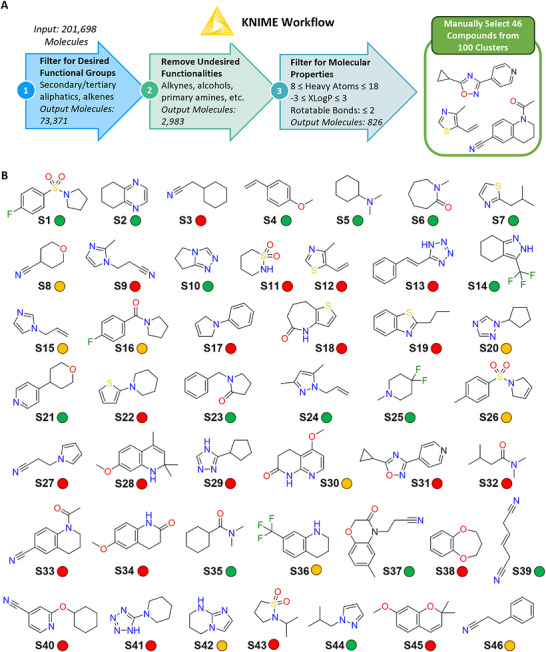
Design and reactivity screen of a diverse set of commercially available substrates. Panel A: overview of the KNIME workflow for the design of the substrate set. Panel B: outcome of a reactivity screen (green: product successfully purified, see Figure 3; orange: expected product mass detected by mass spectrometric analysis; red: expected product mass not detected by mass spectrometric analysis). Conditions: potential substrate (1.0 eq.), pentafluorobenzenesulfonamide (1.3 eq.), PhI(OAc)_2_ (2.0 eq.), 1 mol% Rh_2_esp_2_, MgO (3.7 eq.), 2‐phenylisobutyric acid (0.5 eq.), 5 Å molecular sieves, ^i^PrOAc, 18 h.

We initially designed a set of diverse building blocks that had the potential to be viable substrates for reaction with rhodium nitrenoids. A computational workflow, implemented using KNIME, was used to identify suitable substrates (Figure 2, Panel A, Figures S9–S11 and Tables S1 and S2). Here, a database of commercially‐available compounds was filtered to include compounds with potentially reactive functional groups (secondary and tertiary C−H bonds and alkenes) and appropriate molecular properties, and to exclude compounds with undesired functionality (e.g., alkynes, alcohols, primary amines). The selection of specific potentially reactive functional groups was informed by a limited study in which simple compounds with a single functional group had been evaluated as potential substrates (Supporting Information). The resulting 826 compounds were clustered into 100 clusters from which compounds were manually selected, with no more than one compound selected from each cluster. This enabled the design of a set of 46 commercially‐available compounds that were potential substrates (Figure 2, Panel B).

**FIGURE 3:**
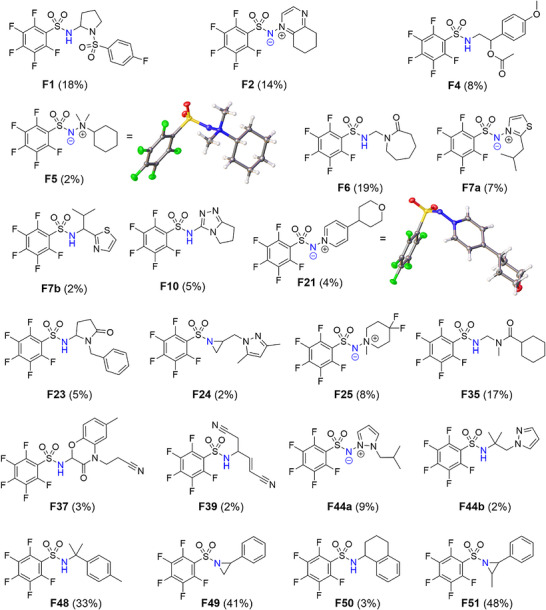
Structures and yields of isolated products from the reaction of available substrates with a rhodium nitrenoid. See Figure 2 for details of the reaction conditions and the substrates.

The set of substrates was screened for productive reaction under conditions that had previously been developed [33]. The reaction components were: potential substrate (1.0 eq, 0.3 mmol; final concentration: 0.48 M), pentafluorobenzenesulfonamide (1.3 eq.), diacetoxyiodobenzene (DAIB) (2.0 eq.), 2‐phenylisobutyric acid (0.5 eq.), MgO (3.7 eq.), and 5 Å molecular sieves (100 mg), with a total reaction volume of 620 µL. After 18 h, the reaction mixtures were analyzed by LC‐MS (Figure S12), and the products of potentially productive reactions were purified by mass‐directed HPLC and, if necessary, column chromatography. Overall, products with the expected mass were observed by LC‐MS analysis in 25 of the 46 reactions (Figure 2, Panel B), and, after purification, a set of 17 reactive fragments was obtained (Figure 3). In general, productive reactions yielded one or two products with expected molecular weights (Figure S12); isolated yields were generally low, in part because of poor product recovery by mass‐directed HPLC. The reactive fragments were complemented by **F48‐F51**, which had previously been prepared in our preliminary studies (Supporting Information). The structures of the reactive fragments were determined by NMR spectroscopic analysis; for example, the formation of the *N‐*amino ylid **F2** was evident from the desymmetrization of its pyrazine ring. In addition, the structures of **F5** and **F21** were determined by x‐ray crystallographic analysis.

The reactive fragments were formed via a range of distinct reactivity modes [32, 34] of rhodium nitrenoids (see Table S1): insertion into C−H bonds that were α to a hetaryl ring (→ **F7b**), α to nitrogen (→ **F1**, **F6**, **F23** and **F35**), α to oxygen (→ **F37**), tertiary (→ **F44b**); aziridination (→ **F4**, **F24** and **F39**); and reaction with the lone pair of a tertiary amine (→ **F5** and **F25**) or a hetarene (→ **F2**, **F7a**, **F21** and **F44a**). In two cases, initial formation of an aziridine was followed by ring‐opening with acetate (→ **F4**) or elimination (→ **F39**). In addition, the fragment **F10** was formed by formal insertion into a hetaryl C−H bond. In many cases, alternative outcomes would be reasonable, but were not observed in the isolated products. Although *N‐*amino ylids of hetarenes are known [35, 36], they have not, to our knowledge, previously been observed to be formed via rhodium nitrenoid chemistry.

### 2.2 Reactivity Profiling Against Aurora A Kinase

**FIGURE 4:**
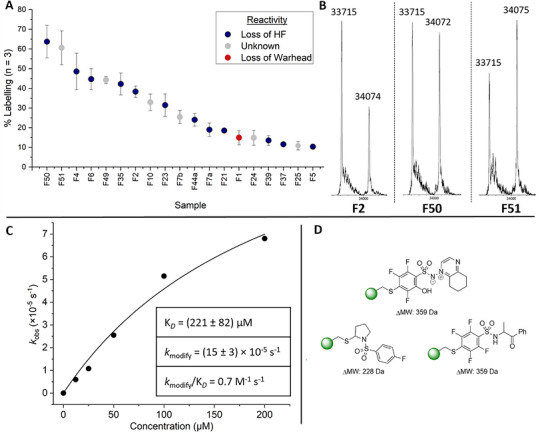
Reactive fragment screen against Aurora A kinase. Panel A: Mass spectrometric analysis of the average conversion of Aurora A kinase into chemically‐modified protein (*n* = 3 with *n = *2 for **F49**; bars: standard deviations). Conditions: Aurora A kinase (2 µM), reactive fragment (20 µM), pH 7.5 TRIS buffer, 24 h. Masses of observed adducts are indicated by color: substitution with loss of HF (blue), substitution with loss of C_6_F_5_SO_2_NH_2_ (red), and other adducts (grey) (see Panel D and main text). Panel B: deconvoluted mass spectra obtained following incubation with fragments **F2**, **F50**, and **F51** (unmodified protein mass: 33715). Panel C: Kinetic characterization of the modification of Aurora A kinase with **F50**. Panel D: Proposed adducts formed by reaction of exemplar reactive fragments with loss of HF (**F2**, top); loss of pentafluorophenylsulfonamide (**F1**, bottom left); and hydration, dehydrogenation, and loss of HF (see text; **F51**, bottom right).

The set of diverse reactive fragments was screened for modification of Aurora A kinase (Figure 4), together with four reactive fragments that had been prepared in our scoping studies (Supporting Information). These experiments enabled the investigation of the reactivity of the fragment set with a model protein. Aurora A kinase is a serine/threonine kinase that plays a key role in cell division, and is overexpressed in many cancers [37, 38, 39]. The reactive fragments (final concentration: 20 µM) were incubated with Aurora A kinase (2 µM) for 24 h, and the products were analyzed by mass spectrometry. In all cases, singly‐modified protein adducts were observed, together with unreacted protein. The observed masses were consistent with nucleophilic substitution with loss of HF (for 13 fragments) (Figure 4, Panel D, top) or pentafluorobenzenesulfonamide (for **F1**) (Figure 4, Panel D, bottom left); for **F10** and **F7b**, the observed mass changes (+315 and +399, respectively) did not correspond with obvious adducts. For **F1**, two out of three replicates were also found to contain a second minor adduct that was consistent with nucleophilic substitution with loss of HF. In addition, although the aziridines **F49** and **F51** were observed to modify the protein, the mass of the observed adduct was consistent with a hydration, a dehydrogenation, and a nucleophilic substitution with loss of HF (+345 and +359, respectively). We demonstrated that *N*‐acetyl cysteine methyl ester underwent the same reaction with **F51** (+359) in a 9:1 pH 7.4 buffer−DMSO (Figure S5). We propose that **F51** was oxidized [40] to a phenyl ketone by DMSO, and then reacted with *N*‐acetyl cysteine methyl ester via nucleophilic substitution (Figure 4, Panel D, bottom right); the molecular formulae of the intermediate and the product were confirmed by accurate mass spectrometry.

We characterized the kinetics of protein modification by **F50**, the reactive fragment for which the highest conversion of modification had been observed (Figure 4, Panel C). The conversion of the protein modification reaction was observed by mass spectrometry over ∼24 h at five different concentrations (12.5–200 µM **F50**), and initial rates, *k*_obs_, of modification were determined (Figure S1). It is notable that the maximum conversion was only ∼85%, perhaps because fragment degradation (t_½_ ∼10 h in pH 7.4 buffer; Figure S7) competed with modification. The protein modification kinetics were consistent with initial binding of the reactive fragment to the protein (with *K_D_* = 220 µM), followed by irreversible modification (with *k*_modify_ = 1.5 x 10^−4^ s^−1^). Additionally, we showed that, after incubation overnight with **F2** (but not **F50**), Aurora A kinase is ∼50% inhibited at high concentrations of **F2** (IC_50_: 2.7 ± 0.9 µM) (Figure S6).

**FIGURE 5:**
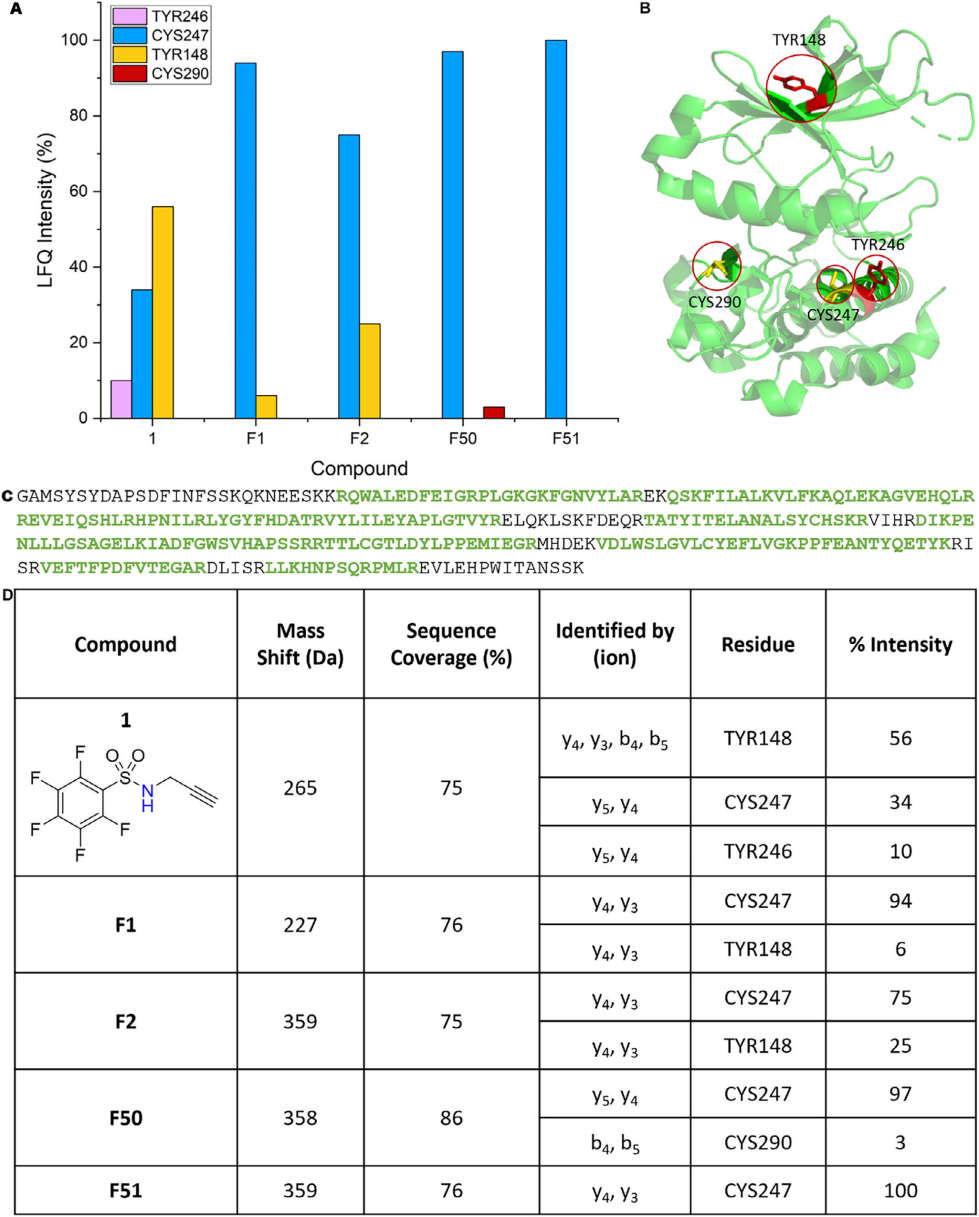
Aurora A modification site identification for selected reactive fragments by proteolysis and LC‐MS/MS analysis. Panel A: Bar chart showing the relative label‐free quantification (LFQ) intensities of peptides modified at different sites. Panel B: The structure of Aurora A kinase (PDB: 6VPG) with modification sites highlighted (red circles) and an overview of coverage (sequence coverage = 75%; green). Panel C: Relative LFQ intensities of modified peptides from selected reactive fragments. Panel D: Basis for the assignment of modification sites.

Modification sites were determined for four reactive fragments (**F1**, **F2**, **F50**, and **F51**) and the *N‐*propargylated pentafluorobenzenesulfonamide probe **1** (Figure 5 and Figure S2). The four reactive fragments were selected because they had been observed to undergo different types of protein modification reactions. These fragments were compared with **1**, a simple probe that participates in nucleophilic aromatic substitution reactions but has minimal other functionality for protein recognition. Initially, the reactive fragments (200 µM) were incubated with Aurora A kinase (20 µM) for 2–5 h, and the resulting modified protein samples were analyzed by mass spectrometry. After sequential treatment with tris(2‐carboxyethyl)phosphine (TCEP) and iodoacetamide, and precipitation, the samples were digested (trypsin/LysC), and the resulting peptides analyzed by LC‐MS/MS. Analysis of the relative label‐free quantification (LFQ) intensities of the modified peptides and their fragmentation patterns revealed that the four reactive fragments all predominantly modified Cys247; however, **F1** (Tyr148), **F2** (Tyr148), and **F50** (Cys290) were also observed to modify other residues.

### 2.3 Proteome‐Wide Reactivity Profiling

**FIGURE 6:**
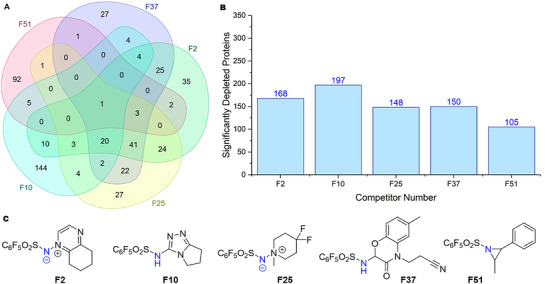
Depletion of proteins in the HeLa cell proteome by preincubation with reactive fragments (50 µM) prior to profiling with IAA (25 µM). Cut‐offs for the identification of depleted proteins were set to −2 log_2_(Difference) and +2 −log(*p*‐value) from a permutation‐corrected two‐sided *t*‐test (*n* = 3 replicate injections). Panels A and B: Number of significantly‐depleted proteins. Panel C: Structures of investigated fragments.

We also profiled five reactive fragments against proteins in HeLa cell lysate (Figures 6 and S8). The five reactive fragments were selected on the basis of their diverse chemotypes: an *N‐*aminopyrazinium ylid (**F2**), an *N‐*hetaryl sulfonamide (**F10**), an *N‐*aminoammonium ylid (**F25**), an *N‐*alkyl sulfonamide (**F37**), and a sulfonyl aziridine (**F51**). To enable enrichment of proteins containing reactive cysteine residues, we used the widely applied reagent iodoacetamide‐alkyne (IAA, also known as *N*‐hex‐5‐ynyl‐2‐iodo‐acetamide) [41, 42]. HeLa cell lysate (350 µg/mL) was incubated for 1 h with the selected reactive fragments (50 µM) or DMSO control, and then for 1 h with IAA (25 µM). Enrichment of the IAA‐labelled proteome was achieved on streptavidin beads following click reaction with a biotinylated azide reagent [43]. The enriched proteins were treated on‐bead sequentially with TCEP and iodoacetamide, and then digested with trypsin/LysC and desalted, and the resulting peptide solution was analyzed by LC‐MS/MS. Unfortunately, peptides derived from Aurora A kinase were not detected in these experiments. However, the enrichment of several other classical protein kinases was significantly reduced by some of the reactive fragments: **F10**: casein kinase II subunit alpha (Uniprot ID: P19784), GSK3β (P49841), and PAK2 (Q13177); and **F25** and **F37**: STK26 (Q9P289). Whilst enrichment of some proteins was reduced by pre‐incubation with more than one reactive fragment, the enrichment of many proteins was significantly reduced by only one of the five reactive fragments (**F2**: 35 proteins; **F10**: 144 proteins; **F25**: 27 proteins; **F37**: 27 proteins; **F51**: 92 proteins).

## 3 Discussion

Our modular synthetic approach enabled the direct attachment of the pentafluorobenzenesulfonamido group to a diverse range of substrates. This approach was not reliant on the availability of amine substrates for sulfonylation; indeed, the approach enabled the preparation of some fragments for which the appropriate amine substrates are not available (or likely to be stable): for example, α‐aminated analogues of **S1**, **S6**, **S23**, **S35**, and **S37**. The rich chemistry of Rh nitrenoids meant that reactive fragments could be prepared via multiple different pathways, including insertion into different types of C─H bonds, aziridination, and insertion into different types of nitrogen lone pairs; this meant that reactive fragments other than simple pentafluorobenzenesulfonamides were accessible.

**FIGURE 7:**
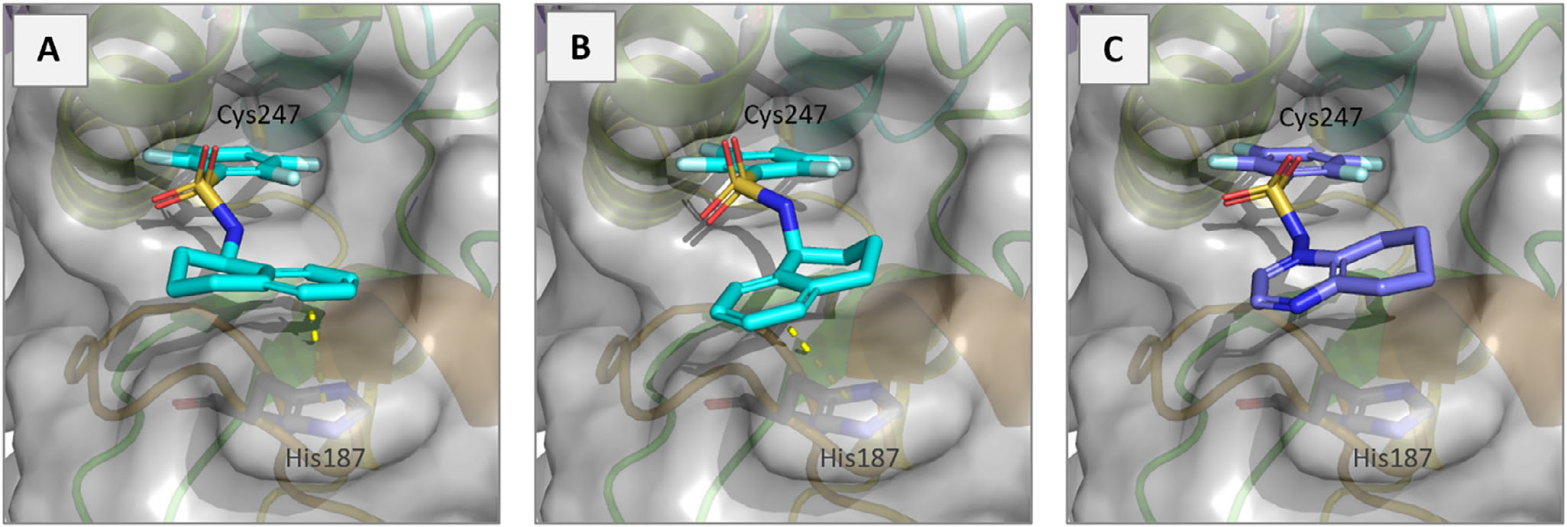
Models of Aurora A kinase (based on PDB 5ORL) following covalent docking of selected reactive fragments to Cys247: *R*‐**F50** (Panel A), *S*‐**F50** (Panel B), and **F2** (Panel C). The π‐π stacking interactions are shown as yellow dotted lines.

Although prepared under the same reaction conditions, the reactive fragments reacted via several different pathways with a model protein, Aurora A kinase. In addition to the expected reaction via nucleophilic aromatic substitution (with loss of HF), other pathways were also observed. For example, nucleophilic substitutions with loss of pentafluorobenzenesulfonamide were possible if the leaving group was α to nitrogen, and, although not observed, aziridine ring‐opening may, in principle, also be possible. The modification sites of specific reactive fragments were mapped, and were found to mainly target Cys247 in Aurora A kinase; however, modification of Tyr148 and Cys290 was also observed. All of the reactive fragments contain multiple electrophilic sites, opening the possibility of crosslinking; however, the observation of the same adducts on both the intact protein and on digested peptides strongly suggests that crosslinking was not observed. The solvent accessibility of cysteines in PDB structure 6VPG was assessed [44]. Remarkably, Cys247 is buried, yet modification of the two other cysteines was, at most, minimal: these other sites were Cys290 (on the activation loop) and Cys319 (which is buried). Notably, a reactive fragment was also observed to modify Cys247 in a previous high‐throughput crystallographic fragment screen [45] and a reactive fragment screen [26]. In addition, modification of Aurora A kinase by **F2** results in partial inhibition of kinase activity (Figure S6), suggesting that Cys247 provides an opportunity for covalent allosteric modulation. The most promising reactive fragment hit was the pentafluorobenzenesulfonamide **F50**. Characterization of the kinetics of its modification of Aurora A kinase revealed that its *k*_modify_/*K*_D_ parameter was 0.7 M^−1^ s^−1^. We used covalent docking [46] (based on PDB 5ORL) to rationalize the basis of the protein modification by **F2** and **F50** (Figure 7). These experiments suggested that both enantiomers of **F50** may engage in π/π stacking interactions with His187. On the basis of this kinetic and structural characterization, fragment **F50** may provide a useful starting point for the discovery of a covalent probe for Aurora A kinase. In line with other reactive fragment starting points [11, 12, 13], optimization of probe specificity for Aurora A kinase would be important: notably, both **F2** and **F50** were also observed to modify Nek7 kinase (Figure S4).

The proteome‐wide modification of reactive cysteine residues by five structurally diverse pentafluorobenzenesulfonamide reactive fragments was also profiled using IAA. The enrichment of 496 distinct proteins was reduced by pre‐incubation with at least one of the five profiled reactive fragments, of which 325 were reduced by pre‐incubation with just one fragment. It was therefore concluded that the structurally diverse reactive fragments reacted with different, albeit overlapping, constellations of proteins. The structure of the reactive fragments, and, in particular, the context of the pentafluorobenzenesulfonamide warhead, was therefore critical in determining which proteins were blocked from enrichment by IAA. We have used referenced protein annotations from the “Illuminating the Druggable Genome” initiative to determine that the 496 proteins were: targets of approved drugs (Tclin; 13 proteins), proteins with small molecule ligands (Tchem; 65 proteins), proteins with well‐studied biology (Tbio; 405 proteins) or proteins for which there is little available information (Tdark; 13 proteins) [47]. The families of these proteins included underrepresented families as well as those of strong historical drug discovery interest: kinases (including protein kinases) (12 proteins), other enzymes (170 proteins), transcription factors (5 proteins), epigenetic proteins (5 proteins), transporters (5 proteins), a GPCR, and an ion channel. Our reactive fragments may present significant opportunities for the development of probes for previously unliganded proteins (e.g., the 418 proteins in the Tbio and Tdark categories).

## 4 Conclusion

Overall, we developed a modular synthetic approach that enabled the preparation of a structurally diverse set of pentafluorobenzenesulfonamide reactive fragments. The structural and reactivity diversity of this fragment set ultimately stemmed from the multiple reaction pathways of Rh nitrenoids, which enabled attachment of the pentafluorobenzenesulfonamide warhead to diverse substrates. The value of the reactive fragment set was demonstrated by screening against an exemplar protein (Aurora A kinase). Here, reactive fragment hits were discovered that selectively modified Cys247 over the more accessible Cys290 on the activation loop, and suggested that Cys247 provides an opportunity for allosteric modulation of kinase activity. Furthermore, five diverse reactive fragments were shown to have distinct reactivity profiles with proteins in HeLa cell lysate. We anticipate that our synthetic approach, perhaps on a larger scale, may provide useful starting points for the discovery of useful chemical probes and drugs.

## Conflicts of Interest

The authors declare no conflicts of interest.

## Supporting information




**Supporting File 1**: chem70990‐sup‐0001‐SuppMat.pdf.

**Supporting File 2**: The authors have cited additional references within the Supporting Information [49, 50, 51, 52].

## Data Availability

The mass spectrometry proteomics data have been deposited to the ProteomeXchange Consortium via the PRIDE [48] partner repository with the dataset identifier PXD072598, and x‐ray crystal structures have been deposited to the Cambridge Crystallographic Data Centre with accession numbers 2520683 and 2520684. Other data is available as Supporting Information.
