# Supplementary material for: Modular Rh‐Catalyzed Synthesis and Biological Profiling of Diverse Pentafluorobenzenesulfonamide Reactive Fragments
Source: Chemistry. 2026 Apr 10;32(24):e00009. doi: 10.1002/chem.202600009 (PMC13290419; doi:10.1002/chem.202600009)
Supplement: Supplementary file 1 — Supporting File 1: chem70990‐sup‐0001‐SuppMat.pdf. [file CHEM-32-e00009-s001.pdf]

SUPPORTING INFORMATION

Unified Rh-catalysed Connective Synthesis and Biological Profiling of Diverse Pentafluorobenzenesulfonamide Reactive Fragments

Julian Chesti,[a,b] Jennifer A. Miles,[b,c] George J. Wade,[a,b] Scott Grossman,[a,b] George W. Preston, [a,b] Richard Bayliss,[b,c] Stuart L. Warriner,[a,b] Megan H. Wright*[a,b] and Adam Nelson*[a,b]

[a] School of Chemistry, University of Leeds, Leeds, LS2 9JT, UK
[b] Astbury Centre for Structural Molecular Biology, University of Leeds, Leeds, LS2 9JT, UK
[c] School of Molecular and Cellular Biology, University of Leeds, Leeds, LS2 9JT, UK
E-mail: m.h.wright@leeds.ac.uk; a.s.nelson@leeds.ac.uk

Contents

1. SUPPLEMENTARY FIGURES 3

2. BIOLOGY EXPERIMENTAL 12

2.1 GENERAL REAGENTS AND BUFFER PREPARATION 12

2.2 HELA CELL CULTURE 12

2.3 AURORA A KINASE SEQUENCE AND EXPRESSION 13

2.4 PROTEIN LABELLING EXPERIMENTS AND ENZYME INHIBITION ASSAYS 15

2.5 PROTEOMICS EXPERIMENTS 16

2.5.1 IDENTIFICATION OF SITE OF LABELLING ON AURORA A KINASE 16

2.5.2 HELA CELL LYSATE PULL-DOWN PROTEOMICS WORKFLOW 17

2.5.3 TANDEM LIQUID CHROMATOGRAPHY MASS SPECTROMETRY (LC-TIMS-MS/MS) FOR PROTEOMICS. 21

3. COMPUTATIONAL EXPERIMENTAL 23

3.1 KNIME WORKFLOW FOR THE SELECTION OF STRUCTURALLY-DIVERSE SUBSTRATES 23

3.2 FRAGPIPE PROCESSING OF PULL-DOWN PROTEOMICS DATA 32

3.3 PERSEUS PROCESSING OF FRAGPIPE DATA 33

3.4 FRAGPIPE WORKFLOW FOR AURORA A KINASE PEPTIDE MAPPING 33

3.5 MSCONVERT AND UNIDEC PARAMETERS FOR THE DECONVOLUTION OF PROTEIN MASS SPECTRA 36

3.6 COVALENT DOCKING OF FRAGMENTS ON AURORA A KINASE 37

4. CHEMISTRY EXPERIMENTAL 39

4.1 FRAGMENT REACTIVITY 39

4.2 GENERAL EXPERIMENTAL CONSIDERATIONS 40

4.3 INVESTIGATION OF FUNCTIONAL GROUP TOLERANCE TOWARDS PENTAFLUOROSULFONAMIDE NITRENE INSERTION 41

4.4 COMPOUND SYNTHESIS 42

4.5 NMR DATA 66

5. REFERENCES 107

1. Supplementary Figures

A

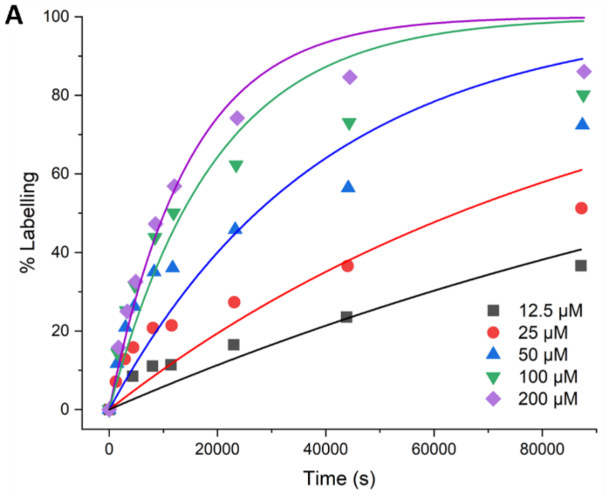

B

Plot	[Conc.]	k_{obs} (× 10^{-5} s^{-1})
■	12.5	0.6 ± 0.1
●	25	1.1 ± 0.2
▲	50	2.6 ± 0.5
▼	100	5.2 ± 0.9
◆	200	6.8 ± 0.7

Supplementary Figure 1: Kinetics data for fragment **F50**. Panel A) Exponential curve fitted to time course data points from incubations with **F50** (12.5-200 µM) and Aurora A kinase (2 µM) over a 24 hour period; Panel C) k_{obs} values calculated from exponentials fitted to time course data points.

A

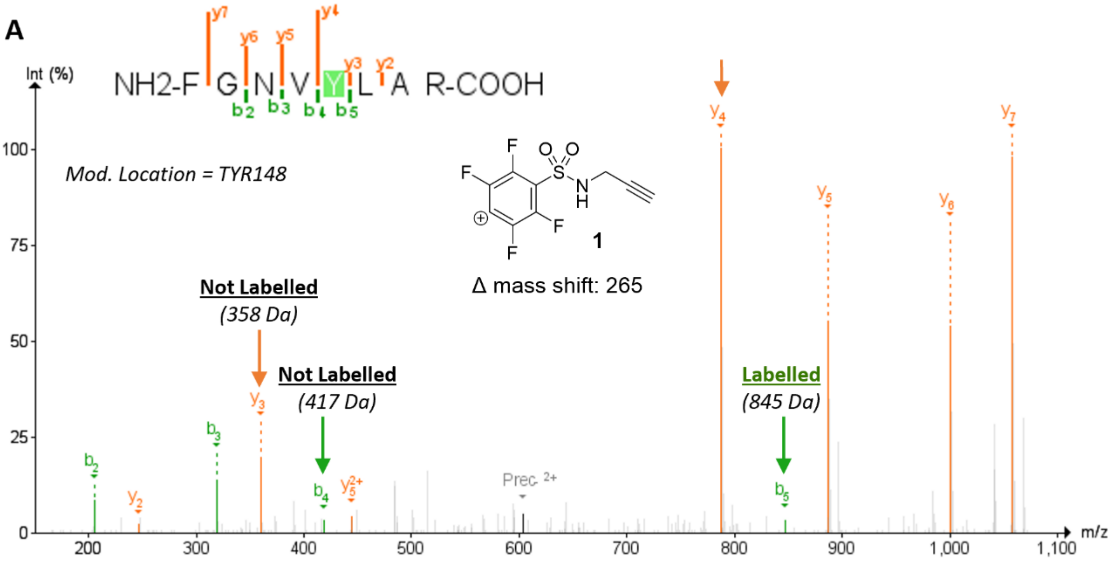

y_3: LAR-COOH *observed mass*: 358; *theoretical mass*: 358 no mass shift
y_4: YLAR-COOH *observed mass*: 786; *theoretical mass*: 521 mass shift of **265** Da
b_4: NH2-FGNV *observed mass*: 417; *theoretical mass*: 417 no mass shift
b_5: NH2-FGNVY *observed mass*: 845; *theoretical mass*: 580 mass shift of **265** Da

B

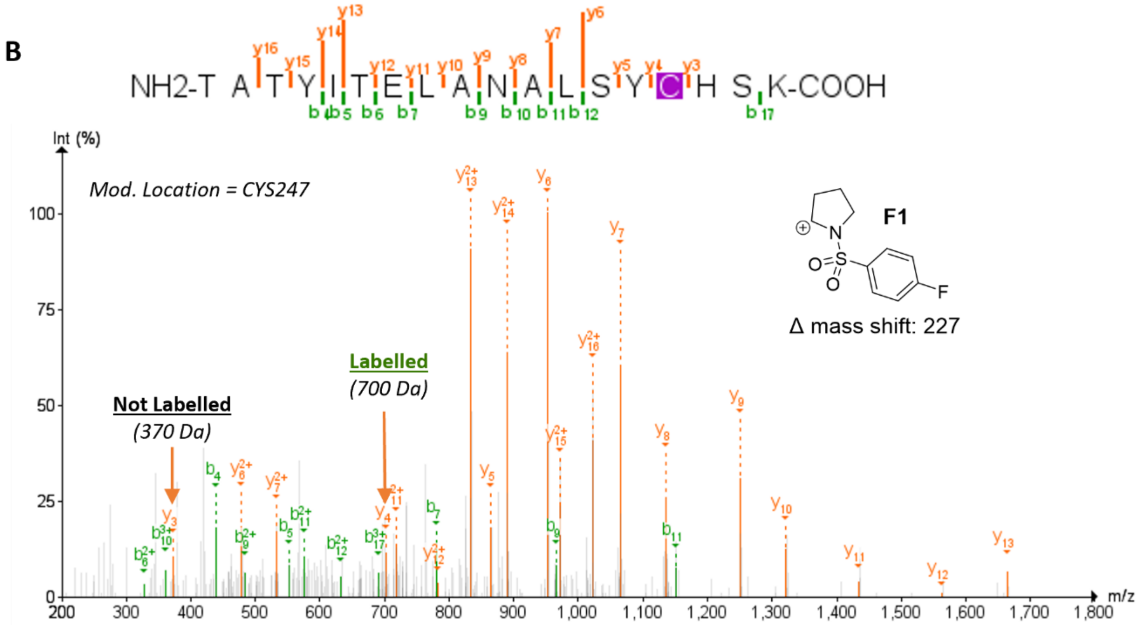

y_3: HSK-COOH *observed mass*: 370; *theoretical mass*: 370 no mass shift
y_4: CHSK-COOH *observed mass*: 700; *theoretical mass*: 473 mass shift of **227** Da

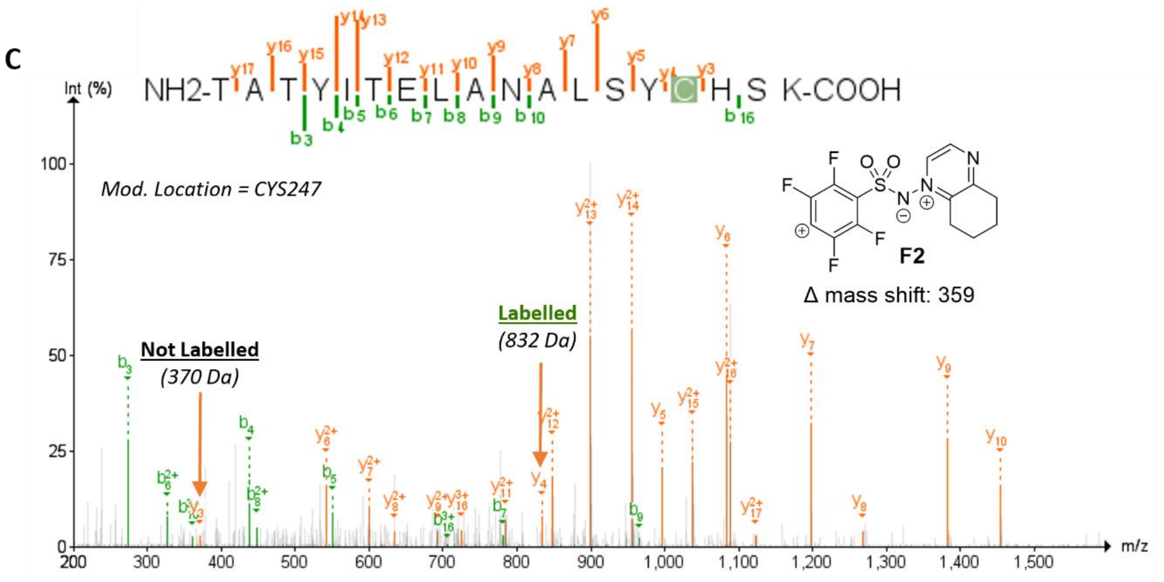

y_3: HSK-COOH *observed mass*: 370; *theoretical mass*: 370 no mass shift
y_4: CHSK-COOH *observed mass*: 832; *theoretical mass*: 473 mass shift of **359** Da

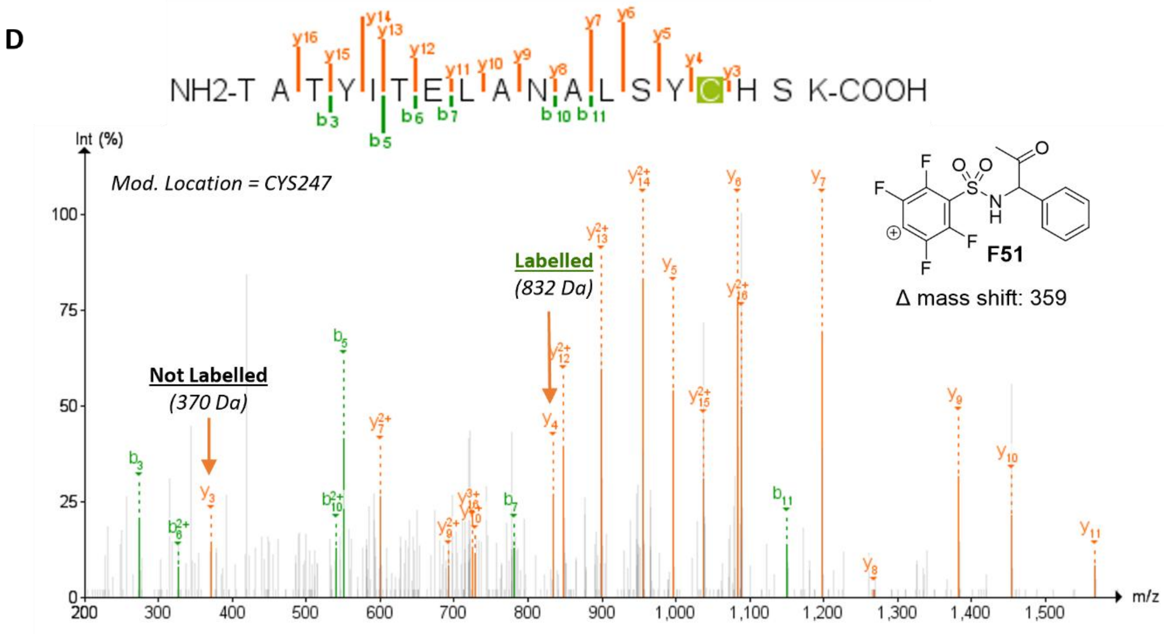

y_3: HSK-COOH *observed mass*: 370; *theoretical mass*: 370 no mass shift
y_4: CHSK-COOH *observed mass*: 832; *theoretical mass*: 473 mass shift of **359** Da

Supplementary Figure 2: Aurora A kinase MS2 data for the most abundant peptides modified by corresponding reactive fragments, key b and y fragmentations used to identified the site of labelling are reported below each spectrum; Panel A) TYR148 labelled with compound **1**; Panel B) CYS247 labelled with fragment **F1**; Panel C) CYS 247 labelled with reactive fragment **F2**; Panel D) CYS247 labelled with reactive fragment **F51**.

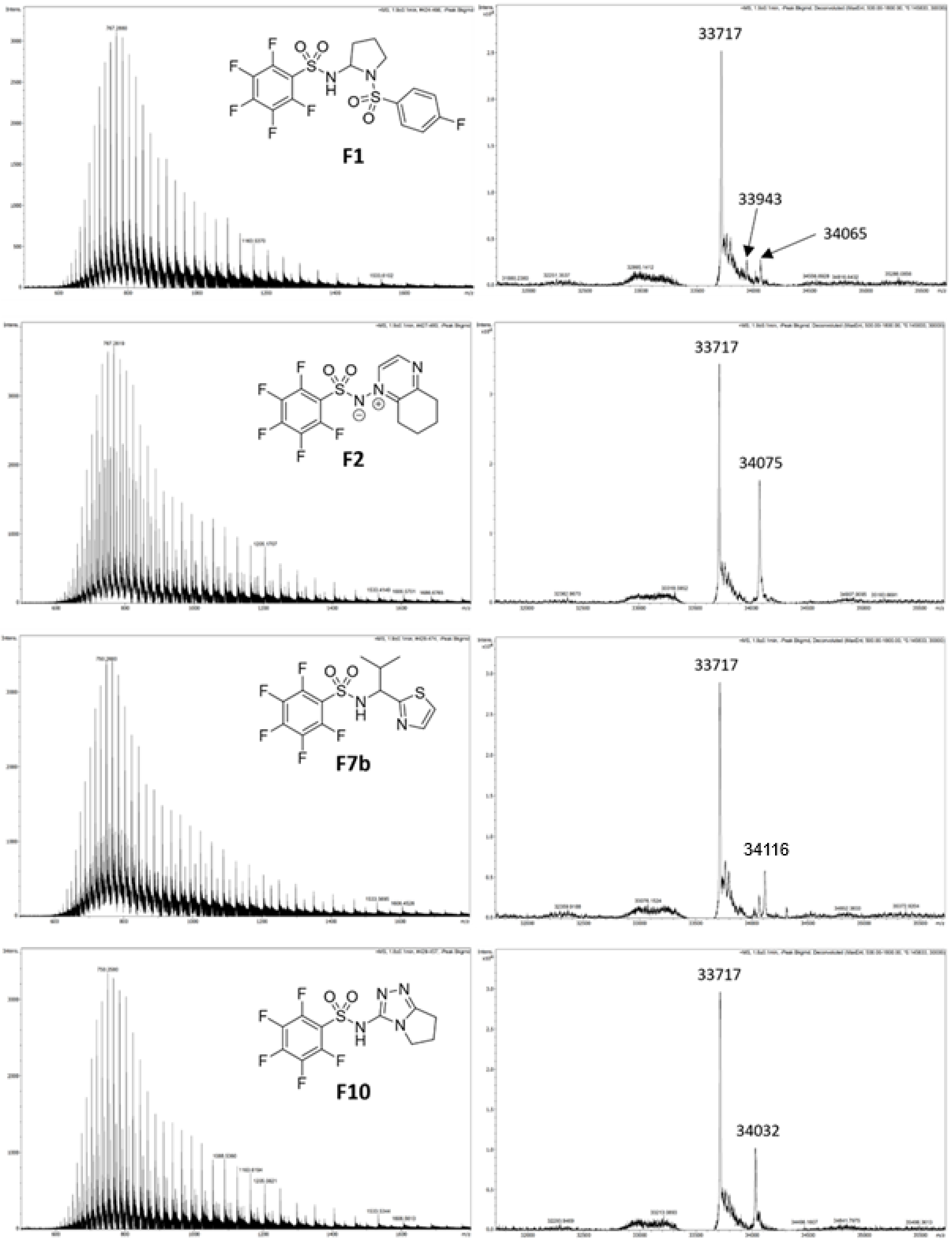
F1
33717
33943
34065
F2
33717
34075
F7b
33717
34116
F10
33717
34032

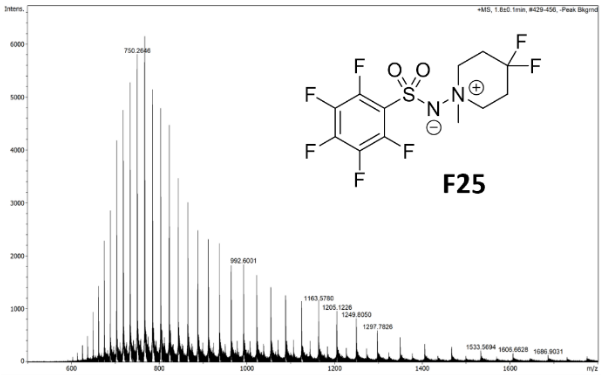
750.2646
992.6001
1163.5780
1205.1226
1249.8050
1297.7826
1533.5694
1606.6628
1686.9031
F25

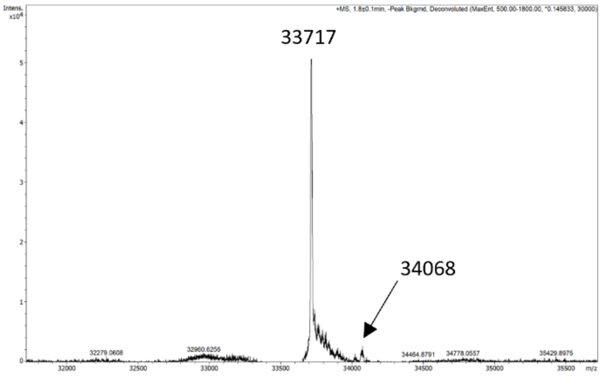
33717
34068

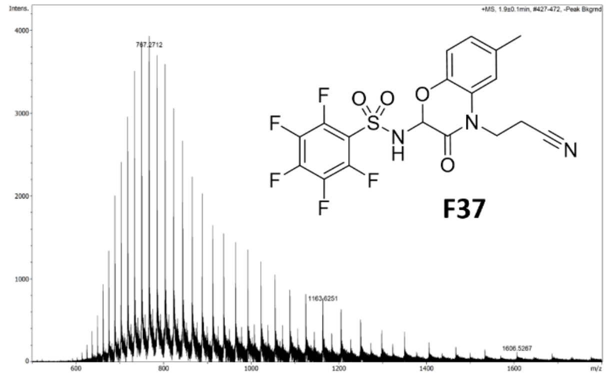
767.2712
1163.6251
1606.5267
F37

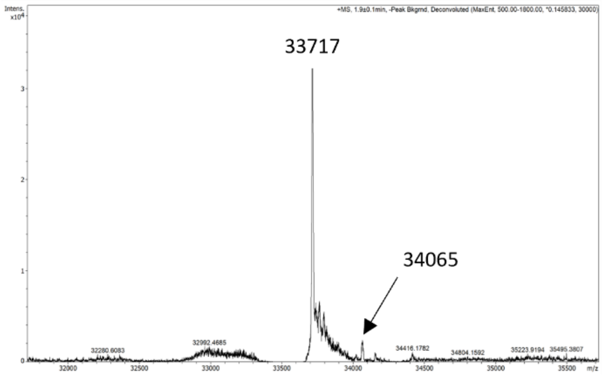
33717
34065

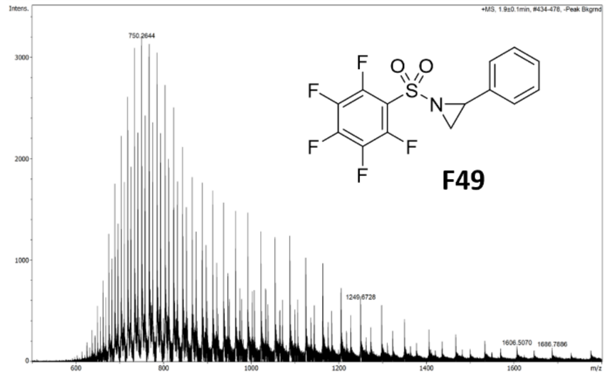
750.2644
1249.6728
1606.5070
1686.7886
F49

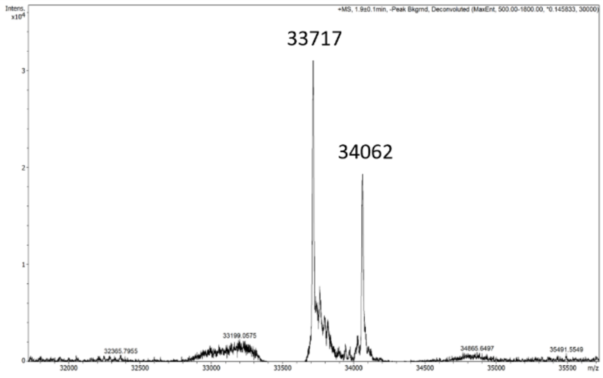
33717
34062

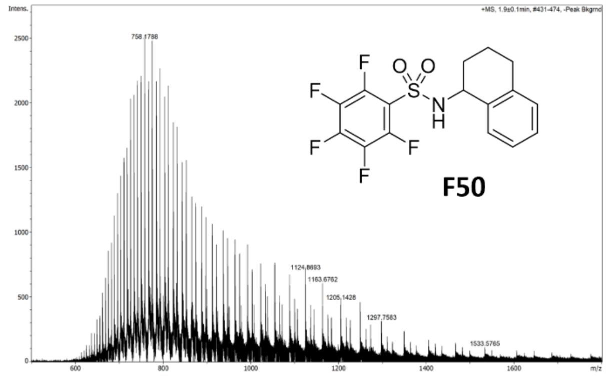
758.1788
1124.8693
1163.6762
1205.1428
1297.7583
1533.5765
F50

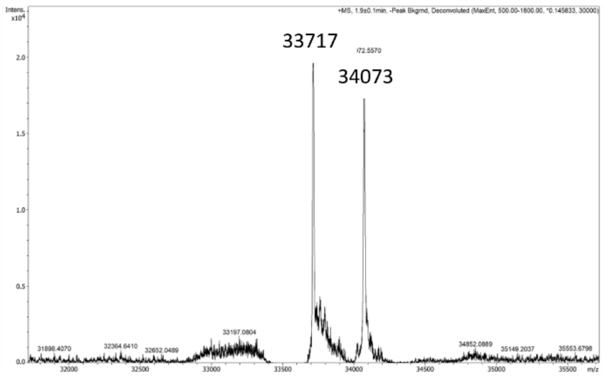
33717
34073

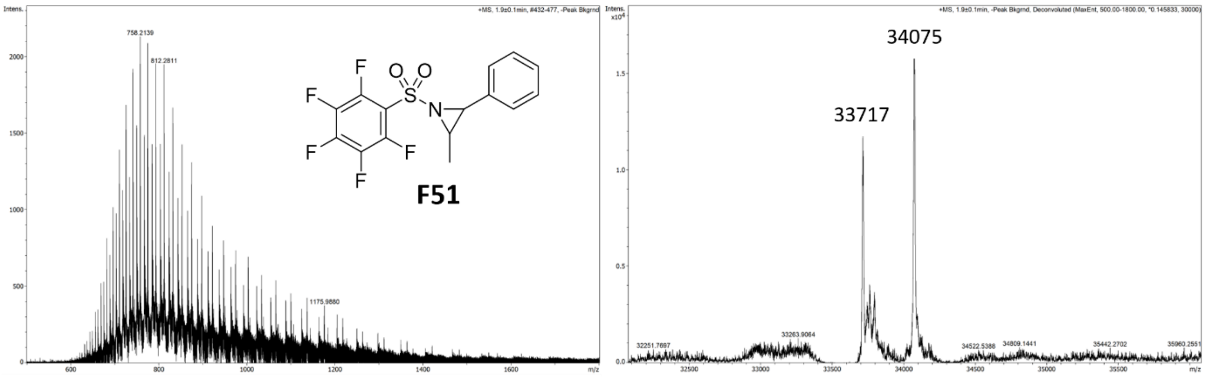

Supplementary Figure 3: Aurora A kinase MS data. Raw intact mass spectrometry spectra (left) and deconvoluted traces (right) after 24 hr incubations with corresponding compounds (20 μM).

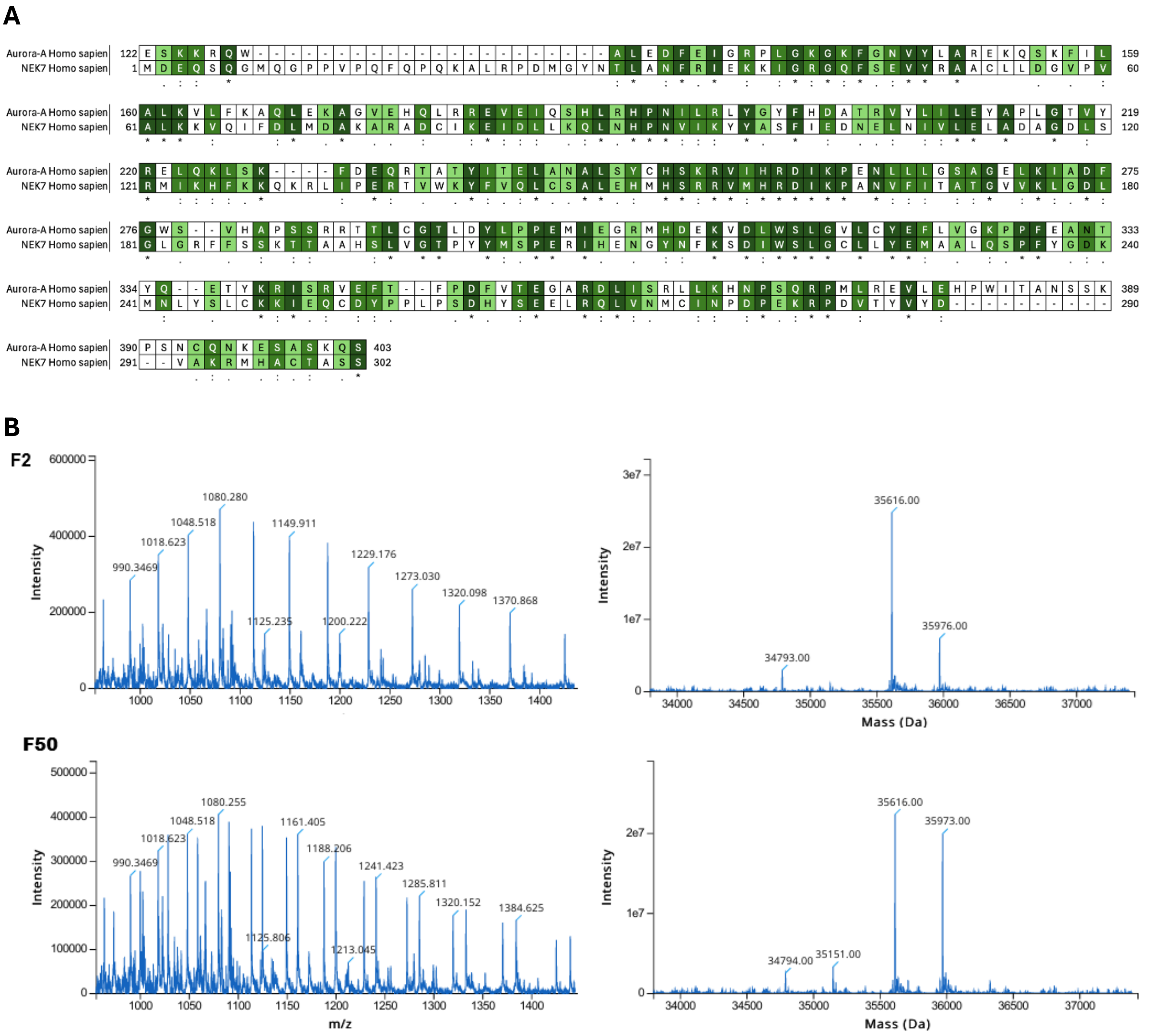

Supplementary Figure 4: Reactive fragment modification of Nek7 kinase. Panel A: Sequence alignment of Nek7 kinase and Aurora A kinase. Panel B: Nek7 kinase MS data. Raw intact mass spectrometry (left) and deconvoluted traces (right) after 24 hr incubations with corresponding compounds (20 μM).

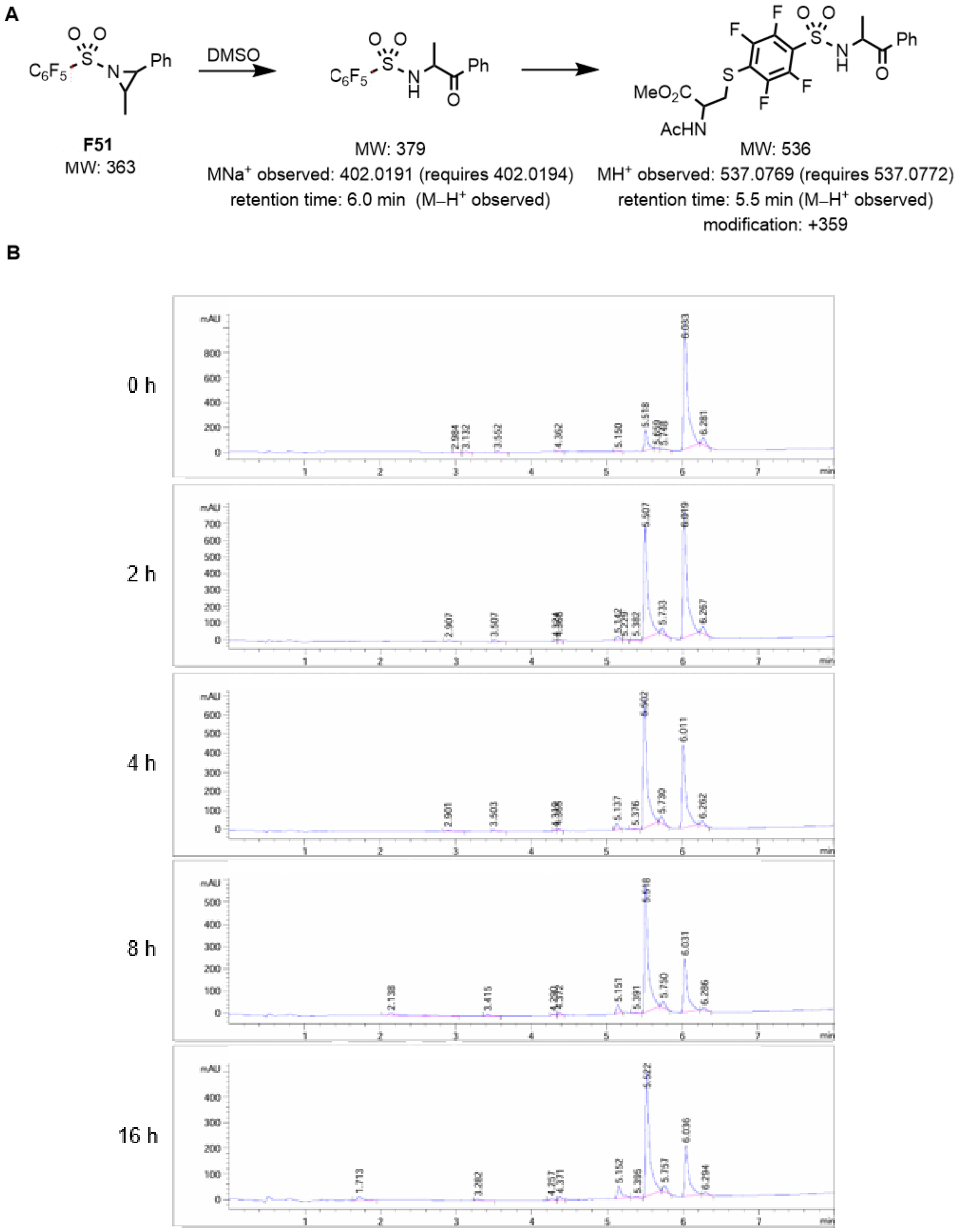

Supplementary Figure 5: Reaction between reactive fragment **F51** and *N*-acetyl cysteine methyl ester in 9:1 pH 7.4 buffer–DMSO. Panel A: Proposed mechanism for the modification of **F51**; the molecular formulae of the intermediate and product were confirmed by accurate mass spectrometry. Panel B: HPLC timecourse of the reaction.

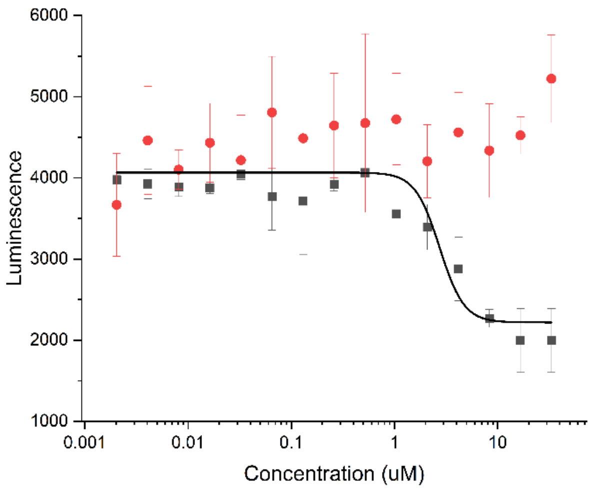

Supplementary Figure 6: Investigation of inhibition of Aurora A kinase by reactive fragments **F2** (black) and **F50** (red). Aurora A kinase was incubated overnight with the reactive fragments before determination of activity using an ADP-Glo assay kit.

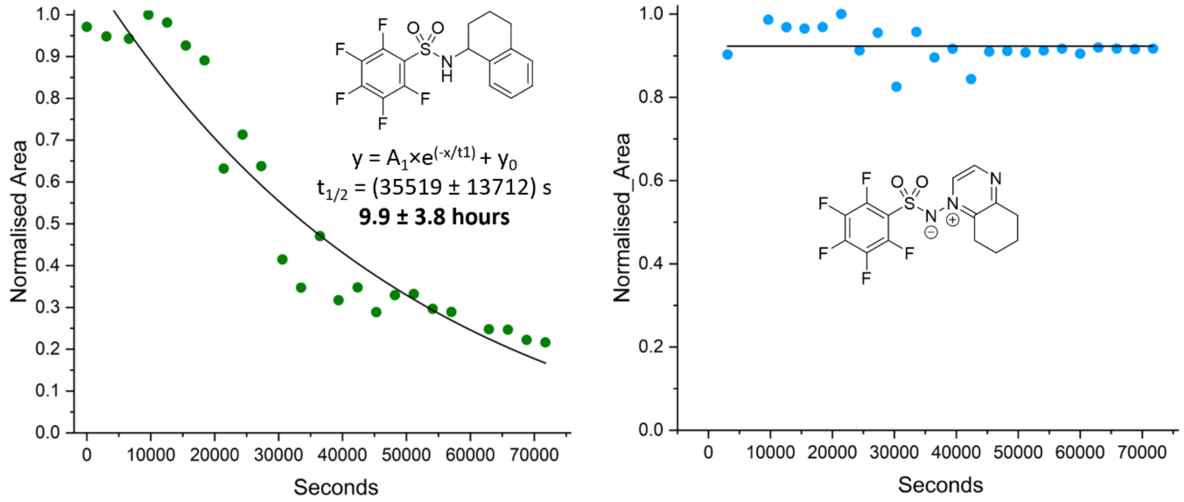

Supplementary Figure 7: Stability assays for compounds **F50** (left) and **F2** (right), samples (200 µM in 25mM TRIS buffer, 150 mM NaCl, 5 mM $MgCl_2$) were injected into a HPLC system every 50 minutes over a 24 hr time period. Left Panel: decomposition of fragment **F50** over 24 hr with a half-life of circa 10 hr. Right Panel: fragment **F2** over 24 hours showing no decomposition.

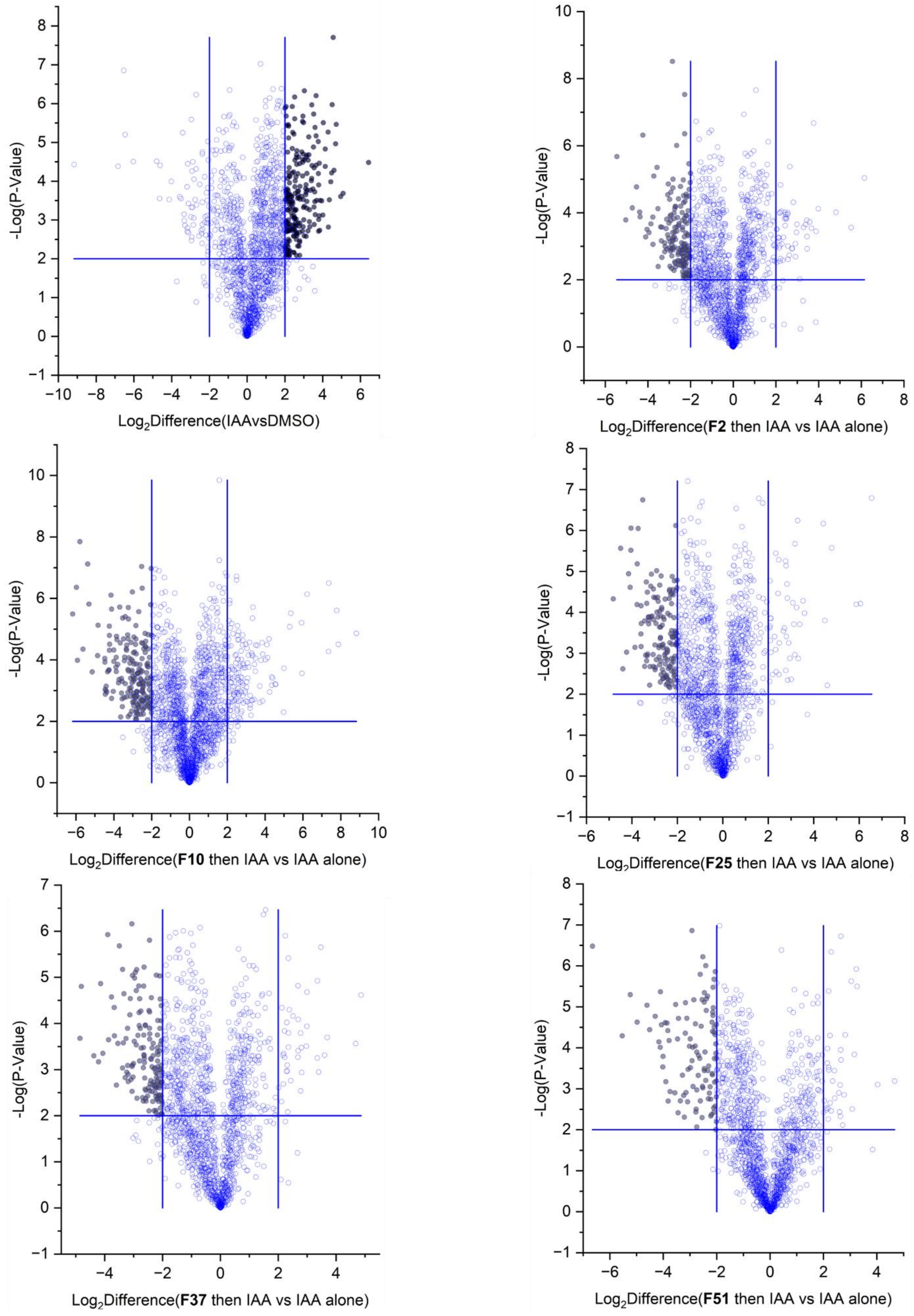

Supplementary Figure 8: Volcano plots from competition proteomics. Data was collected as single pull-down samples injected three times. Thresholds for identifying enriched or depleted proteins are set to - 2 Log_2(Difference) and +2 -Log(p-value) from a permutation-corrected two-side t-test. Depleted proteins are highlighted as filled light blue circles.

Feature in substrate	Observed reactivity	Example reactive fragment
C-H α to nitrogen in a sulfonamide	C-H insertion	**F1**
Hetarene with N lone pair (e.g. pyrazine, thiazole, pyridine, pyrazole)	Ylid formation	**F2**, **F7a**, **F21**, **F44a**
Alkene	Aziridine formation	**F4**, **F24**, **F39**, **F49**, **F51**
Basic tertiary amine	Ylid formation	**F5**, **F25**
C-H α to nitrogen in an amide or lactam	C-H insertion	**F6**, **F23**, **F35**
Benzylic C-H or C-H α to a hetarene (e.g. thiazole)	C-H insertion	**F7b**, **F48**, **F50**
1,2,4-triazole	Amination at 3 position	**F10**
C-H α to oxygen in an ether	C-H insertion	**F37**
Tertiary C-H	C-H insertion	**F44b**

Supplementary Table 1: Observed reactivity in connective reactions. This information may help identify modes of reactivity, and issues of chemoselectivity, relevant to other substrates with one or more of the listed features.

2. Biology Experimental

2.1 General Reagents and Buffer Preparation

Phosphate-Buffered Saline (PBS): pH 7.4 (purchased as tablets from Sigma-Aldrich, 79382) containing phosphate (10 mM), potassium chloride (2.7 mM) and Sodium chloride (137 mM). For use in cell cultures and proteomics a solution of PBS was purchase from Fischer Scientific (catalogue number 15374875).

TRIS (pH 7.5) Buffer: tris(hydroxymethyl)aminomethane (25 mM), NaCl (150 mM) and MgCl2 (5 mM) in H_2O (100 mL).

Cell Growth Media: High glucose DMEM (gibco reference number: 11995-065) supplemented with Foetal Bovine Serum (10% v/v) and penicillin streptomycin (1% v/v, gibco reference number: 15070-063).

Ammonium Bicarbonate buffer (ABC): pH 8.0 containing ammonium bicarbonate (50 mM) in H_2O.

2.2 HeLa Cell Culture

HeLa cells (Research Resource Identifier: CVCL_0030) were grown at 37 °C in Cell Growth Media (see section 2.1) in 75 cm^2 Nunc EasYFlask (ThermoFischer, Catalog Number: 156499) in a humidified atmosphere containing 5% CO_2. Once cell confluency of around 80% was reached, attached cells were trypsinised (0.05% trypsin, 0.05 M EDTA, phenol red, Corning part number: 25-051-CI), pelleted at 500 × g for 5 min and washed with PBS buffer to remove excess trypsin. Cells were resuspended in growth media, and one-tenth of cells were transferred to new 75 cm^2 flasks. Cell samples were discarded after the tenth passage. In general, cells were collected, pelleted and stored at –80 °C for proteomics assays once a confluency of around 80% was reached in 8 × T75 flasks (calculating 0.75 - 1.0 T75 flasks *per* proteomics sample).

2.3 Protein Sequence and Expression

Aurora A Kinase [116-389] (MW: 33716 Da):

GAMSYSYDAPSDFINFSSKQKNEESKKRQWALEDFEIGRPLGKGKFGNVYLAREKQSKFILALKVLFKAQLEKAGVEH
QLRREVEIQSHLRHPNILRLYGYFHDATRVYLILEYAPLGTVYRELQKLSKFDEQRTATYITELANALSYCHSKRVIHRDIK
PENLLLGSAGELKIADFGWSVHAPSSRRTTLCGTLDYLPPEMIEGRMHDEKVDLWSLGVLCYEFLVGKPPFEANTYQ
ETYKRISRVEFTFPDFVTEGARDLISRLLKHNPSQRPMLREVLEHPWITANSSK

Aurora A Kinase [122-403] (MW: 32896 Da):

GAMESKKRQWALEDFEIGRPLGKGKFGNVYLAREKQSKFILALKVLFKAQLEKAGVEHQLRREVEIQSHLRHPNILRL
YGYFHDATRVYLILEYAPLGTVYRELQKLSKFDEQRTATYITELANALSYCHSKRVIHRDIKPENLLLGSAGELKIADFGW
SVHAPSSRRTTLCGTLDYLPPEMIEGRMHDEKVDLWSLGVLCYEFLVGKPPFEANTYQETYKRISRVEFTFPDFVTEG
ARDLISRLLKHNPSQRPMLREVLEHPWITANSSKPSNCQNKESASKQS

Human Aurora A kinase domain 116-389 fused at the N-terminus to TPX2 7-20 in vector (pET28a+) [or Human Aurora A kinase domain 122-403 in vector (pet30TEV)] were transformed into RIL cells alongside the pCDF vector encoding lambda phosphatase. The plasmid TPX2 fused plasmid was purchased from GenScript. The proteins were overexpressed in LB, with growth at 37 °C until the O.D. at 600 nm reached 0.6-0.8. Expression was then induced with 0.5 mM IPTG overnight at 20 °C. The pelleted cells were resuspended in 10 ml of ice-cold lysis buffer per litre of grow (50 mM TRIS pH 7.5, 250 mM NaCl, 20 mM imidazole, 10% glycerol, 5 mM MgCl2, one EDTA-free protease inhibitor tablet per 50 ml of buffer). The resuspended cells were sonicated at 60% amplitude for 10 sec on, 20 sec off, 5 min total to lyse them. The soluble was collected at 17000 rpm for 5 min.

After filtering through a 0.45 µm filter the soluble was loaded onto a HisTrap HP. Any bound protein was eluted free in a gradient of lysis buffer including 500 mM imidazole. The His-tag was then cleaved overnight using TEV protease in dialysis at 4 °C into 50 mM TRIS pH 7.5, 250 mM NaCl, 10% glycerol, 5 mM $MgCl_2$. After dialysis, the cleaved protein was rebound to the HisTrap equilibrated in dialysis buffer. The Aurora interacted with the HisTrap still after cleavage so a gradient of 500 mM imidazole was used to elute off the tag-free protein.

The Aurora A containing fractions were concentrated down in a 10 kDa cut-off concentrator and loaded onto a SD200 16/600 size exclusion column equilibrated into 50 mM TRIS pH 7.5, 200 mM NaCl, 10% glycerol, 5 mM $MgCl_2$. In the final step Aurora A was concentrated down again and flash-frozen before storage at –80 °C.

<u>NEK7 (MW: 35616 Da)</u>

MDEQSQGMQGPPVPQFQPQKALRPDMGYNTLANFRIEKKIGRGQFSEVYRAACLLDGVPVALKKVQIFDLMDAK
ARADCIKEIDLLKQLNHPNVIKYYASFIEDNELNIVLELADAGDLSRMIKHFKKQKRLIPERTVWKYFVQLCSALEHMH
SRRVMHRDIKPANVFITATGVVKLGDLGLGRFFSSKTTAAHSLVGTPYYMSPERIHENGYNFKSDIWSLGCLLYEMAA
LQSPFYGDKMNLYSLCKKIEQCDYPPLPSDHYSEELRQLVNMCINPDPEKRPDVTYVYDVAKRMHACTASSLEHHH
HHH

Full-length (FL) Nek7 bearing a non-cleavable C-terminal 6×His tag (cloned in pET30; Novagen) was co-transformed with λ-phosphatase (in pCDF-Duet) into *Escherichia coli* BL21(DE3)-RIL competent cells (Thermo Fisher Scientific). Cultures were grown in Luria–Bertani (LB) medium supplemented with kanamycin (50 µg ml^{-1}), spectinomycin (50 µg ml^{-1}), and chloramphenicol (25 µg ml^{-1}). Co-expression with λ-phosphatase was employed to prevent autophosphorylation and to obtain homogeneous, unphosphorylated Nek7 protein. Cell pellets were harvested and lysed by sonication in lysis buffer containing 50 mM HEPES (pH 7.5), 300 mM NaCl, 20 mM imidazole, 5 % (v/v) glycerol, 1 mM $MgCl_2$, 0.2 mM $MnCl_2$ and a protease inhibitor tablet. The clarified lysate was loaded onto a 5 ml HisTrap affinity column (Cytiva), pre-equilibrated with lysis buffer, and eluted using a linear gradient of 20–250 mM imidazole. Eluted fractions containing Nek7 were pooled, concentrated and subjected to size-exclusion chromatography on a Superdex 200 16/600 column (Cytiva) equilibrated with buffer containing 50 mM HEPES (pH 7.5), 300 mM NaCl, 5 % (v/v) glycerol and 5 mM dithiothreitol (DTT). Fractions corresponding to monomeric Nek7 were collected, concentrated and analysed by SDS–PAGE to assess purity and flash frozen in liquid nitrogen.

2.4 Protein Labelling Experiments and Enzyme Inhibition Assays

Fixed timepoint, time courses and kinetics

Solutions of purified compounds in DMSO (20 mM) were diluted tenfold in DMSO in Eppendorf tubes. Stock solutions (2 mM) were then diluted a further tenfold in TRIS buffer to make the desired working solutions (200 µM, 1% DMSO). Working solutions of reactive fragments were added (10 µL) to Waters QuantRecovery MaxPeak vials (part number: 186009186) containing a solution of Aurora A kinase in TRIS buffer. The final volume of the labelling reactions was 100 µL so that the desired concentration of kinase (2 µM, 1 equiv.) and reactive fragment (20 µM, 1% DMSO, 10 equiv.) was achieved. The vials were then sealed with PTFE screwcaps and incubated on a Bruker Impact II QqTOF autosampler at 21 °C.

For kinetics experiments and the initial calculation of k_{modify}, concentrations of reactive fragments were varied starting from 12.5 µM, then 25 µM, 50 µM, 100 µM and finally 200 µM. Aurora A kinase concentration was kept constant at 2 µM. Aliquots of the samples (5 µL) were then injected at 0 min, 30 min, 1 hr, 2 hr, 3 hr, 6 hr, 12 hr and 24 hr time points. Exact time points in seconds were then calculated from the collected data. Raw Brucker .d files were then reprocessed using BafPipe software (see section 3.5). An exponential association curve (ExpAssoc1) was fitted to the plotted data points in OriginPro. The equation is reported below, where the time offset TD and the baseline value y were both set to 0, assuming that at T_0 none of the protein is modified by the reactive fragments. The amplitude A was set to 100 assuming that the labelling reaction will reach 100% at an end timepoint. From this equation, k_{obs} was calculated as 1/τ.

$$y = Yb + A \times \left[1 - e^{-\frac{(x-TD)}{\tau}}\right]$$

For the calculation of kinetic constants K_D and k_{modify}, the observed rate of labelling k_{obs} was plotted against concentration. The Michaelis-Menten equation (reported below) was then fitted to the data points in Oregano, where V_{max} corresponds to k_{modify} and K_m corresponds to K_D.

$$y = \frac{V_{max}x}{K_m + x}$$

Kinase inhibition assays

An ADP-Glo assay kit was used to test the effect of reactive fragments on the ATPase activity of Aurora-A kinase. Reactive fragments (final concentrations: ~1 nM to ~33 μM; final percentage of DMSO: 1%) were titrated into 50 nM Aurora-A 122-403 dephosphorylated protein in a buffer of 40 mM Tris pH 7.5, 150 mM NaCl, 10 mM magnesium chloride, 1 mM DTT, 0.1 mg/ml BSA, 0.01% Tween 20. The samples were incubated at room temperature overnight to allow covalent modification of Aurora-A to occur. Reactions were then initiated by the addition of 10 μM ATP and 100 μM kemptide as substrate (Cambridge Bioscience Ltd). The reactions were incubated at room temperature for 60 min before 5 μl was transferred into 5 ul of ADP-Glo Reagent (Promega) in a 384-well low volume low binding plate (Greiner Bio-one). After a further 40 min at room temperature, 10 μl of kinase detection reagent was added to each well and the reaction allowed to incubate for an additional 30 min. Luminescence was measured on a HIDEX Sense plate reader (HIDEX) using a 1 sec integration time. Control data was subtracted from the luminescence, and three repeats were averaged before being fitted to a sigmoidal plot using Origin (OriginLab) to allow IC_{50} values to be calculated.

2.5 Proteomics Experiments

2.5.1 Identification of Site of Labelling on Aurora A Kinase

Recombinant Aurora A kinase (3.71 mg/mL, 110 μM) domain [116-389], was allowed to thaw on ice. A control sample of kinase was prepared by transferring 7.3 μL of the thawed stock solution to a 1.5 mL low binding Eppendorf containing ammonium bicarbonate (ABC) buffer (32.7 μL). A second sample was prepared by transferring 7.3 μL of kinase stock into ABC buffer (28.7 μL) followed by a stock solution of reactive fragment (4μL, 2mM, 10% DMSO in ABC buffer). The final concentration of protein was 20 μM (c.a. 26 μg), DMSO 1% and fragment 200 μM. The samples were incubated for 2 to 5 hr at room temperature (circa 21 °C). An aliquot was taken of each sample (5 μL) and 1 μL injected onto a Bruker Impact II QqTOF mass spectrometer to verify sample integrity and extent of labelling.

Next, Waters RapiGest SF Surfactant (5.5 μL, 1% w/v in ABC buffer, Part Number: 186001861) was added to each protein sample followed by TCEP·HCl (5 μL, 55 mM in ABC buffer). Protein samples were then allowed to reduce and denature at 60 °C for 30 min with agitation (500 rpm). Alkylation was

carried out by transferring a solution of iodoacetamide (5 µL, 180 mM in ABC buffer) to each sample. Denatured proteins were then allowed to alkylate in the dark for 30 min at room temperature. Removal of excess reagents was achieved with overnight acetone (240 µL) precipitation at -20 °C.

Proteins were then pelleted by centrifugation at 14k × g for 10 min at 4 °C, the supernatant was discarded, and the pellets were washed with a 4:1 mixture of acetone/ABC buffer (300 µL). This process was repeated once more, and the protein pellets were allowed to air dry for a maximum of 30 min. Proteins were then resuspended in a RapiGest solution (18 µL, 0.11% in ABC buffer). Solubilisation was aided with sonication and incubation at 30 °C with agitation (1000 rpm). Proteins were then digested for 16 hr at 37 °C with agitation (500 rpm) with a working solution of Trypsin/LysC (2 µL, 0.1 µg/µL in ABC buffer, Promega, Product Code: V5073).

Samples were then spun at 10k × g for 1 minute and incubated for 15 min at 37 °C with agitation (500 rpm). Enzymatic digestion was stopped with TFA (4 µL, 3% in H_2O). Samples were incubated for a further 45 min at 37 °C with agitation (500 rpm) and centrifuged at 13k × g for 10 min. Samples for LC-MS/MS analysis were prepared by adding 88 µL of a 70% MeCN solution with 0.1% FA to each digest, then 20 µL of this digest solution was diluted further in 530 µL of a 0.1% FA in H_2O solution. Samples were centrifuged at 10k x g for 2 min and supernatant (50 µL) was transferred into a polypropylene vial for timsTOF LC-MS/MS analysis (see Section 3.4 for data analysis).

2.5.2 HeLa Cell Lysate Pull-down Proteomics Workflow

Lysate preparation: HeLa cell pellets were taken up in PBS buffer (1 mL) followed by cOmplete Mini, EDTA free protease inhibitor cocktail (100 µL from a 10× stock solution, Merck part number: 11836170001). Cells were physically lysed by sonication (3 × 10 s pulses at 40% intensity) on ice in low-binding Eppendorf tubes and spun at 13.3k rpm for 15 min. The supernatant was collected, and the protein concentration was determined by Pierce BCA assay. The lysate was then aliquoted into fresh 2 mL low-binding Eppendorf tubes (40 µL, 440 µg) and used immediately for pull-down experiments.

Lysate Incubation with Reactive Fragment Competitor and Iodoacetamide Alkyne: The volume of each sample was made up to 440 µL with PBS so that the concentration of protein in each sample was 1 mg/mL. A solution of competitor (44 µL, 500 µM, 10% DMSO in PBS) was added to corresponding Eppendorf tubes, a positive control sample with iodoacetamide alkyne (44 µL, 250 µM, 10% DMSO in PBS) and a DMSO control (44 µL, 10% in PBS) sample were also prepared. Samples were then incubated for 1 hr at 25 °C with agitation (500 rpm). Samples with reactive fragment competitors were then treated with iodoacetamide alkyne (48.9 µL, 250 µM, 10% DMSO) and incubate for a further hour with agitation (500 rpm). A 10% SDS solution in H_2O was then added to each Eppendorf so that the final concentration of SDS in each lysate sample was 1%.

CuAAC Ligation of Biotin Azide to Alkynylated Probe: A stock solution of 'click' reagents was prepared containing AzRB (azido-arginine-biotin)[1] using the following table:

Order of Addition	Reagent	Reagent Stock	Volume (µL)
1	AzRB	10 mM in DMSO	50
2	$CuSO_4$	50 mM in H_2O	100
3	TCEP	50 mM in H_2O	100
4	TBTA	10 mM in DMSO	50

Click solution (10 µL) was added to each Eppendorf so that the probe-to-biotin azide ratio was 1:1.1 equivalents (28 µM AzRB for 25 µM probe). The samples were then incubated for a further 1 hr at 25 °C with agitation (500 rpm). The click reaction was then quenched with EDTA (7.5 µL, 0.5 M in H_2O).

Protein Precipitation: Proteins were precipitated with 4 volumes of ice-cold methanol, 1.5 volumes of ice-cold $CHCl_3$ and 3 volumes of ice-cold water. Samples were vortexed and spun at 13k rpm for 10 min. The supernatant was discarded, and a further 4 volumes of methanol were added to each sample. Samples were gently shaken and spun again at 13k rpm for 10 min. Protein pellets were resuspended in 4 volumes of methanol and spun at 13k rpm for 10 min. Pellets were allowed to air-dry for 20 min and then were resuspended in 2% SDS in PBS (50 µL) so that the final concentration of lysate was 10 mg/mL.

Affinity Enrichment of Biotinylated Proteins: Pierce high capacity NeutrAvidin agarose beads (Thermo Scientific part number: 29202) were transferred into a 2 mL low-binding Eppendorf using a wide-bored pipette tip (50 µL of slurry per biotinylated sample). The beads were spun at 1.5k × g for 2 min and allowed to stand at room temperature for 2 min before removing the supernatant storage solution. The beads were then equilibrated with 4 volumes of 0.1% SDS in PBS, spun for 2 min at 1.5k × g and allowed to stand for 2 min before removing the supernatant solution. This equilibration process was repeated for a total of 3 times. Beads were then resuspended to the original starting volume with 0.1% SDS in PBS. Reconstituted protein samples were then diluted with PBS (1 mL) so that the final concentration of SDS was 0.1%. Equilibrated NeutrAvidin Beads (25 µL) were then added to each biotinylated lysate sample. Eppendorf tubes were then sealed with parafilm and incubated at room temperature on a head-to-tail mixer for 2 hr. Beads were then washed according to the following table. Samples were spun at 1.5k × g for 2 min and the beads were allowed to settle for 2 min before discarding the supernatant after each wash.

Washing Solution	Total Washes
0.5% SDS in PBS	3 × 500 µL
6M Urea in PBS	3 × 500 µL
H_2O	3 × 500 µL
50 mM TEAB in H_2O	3 × 500 µL

On-bead Protein Denaturing: After the final washing step the NeutrAvidin beads were resuspended in a TCEP·HCl solution (400 µL, 12.5 mM in 50 mM TEAB) to denature enriched proteins. Samples were incubated for approximately 1 hr at 30 °C with agitation (500 rpm). Incubated samples were then centrifuged at 1.5k × g for 2 min and the beads were allowed to settle at room temperature for 2 min before discarding the supernatant solution. Beads were then washed with TEAB (800 µL, 50 mM), centrifuged and the supernatant was discarded.

On-bead Protein Alkylation: Proteins were then treated with a solution of iodoacetamide (400 µL, 18.75 mM in 50 mM TEAB). Samples were left to stand for approximately 1 hr in the dark and

centrifuged at 1.5k × g for 2 min. The supernatant solution was removed after allowing the beads to settle for 2 min after centrifugation. Beads were washed with further TEAB (800 µL, 50 mM), centrifuged and the supernatant was discarded.

On-bead Protein Digestion: Lyophilised sequencing grade trypsin (Thermo Scientific product code: 13464189) was reconstituted (20 µL, 50 mM AcOH, 1 µg/µL) and added to each bead-treated sample (5 µL, 5 µg). Eppendorf tubes were then incubated at 37 °C for 15 to 17 hr with agitation (500 rpm). After incubation, samples were centrifuged at 5k × g for 5 min and allowed to stand for 2 min before collecting the supernatant digest in a separated 1.5 mL low-binding Eppendorf tube. Beads were then washed with a solution of acetonitrile (75 µL, 80% MeCN in H_2O with 0.1% formic acid), centrifuged at 1.5k × g for 2 min, allowed to stand for 2 min and the supernatant was collected. This step was repeated for a total of 3 times. The pH of the collected peptide solution was adjusted to 3 with trifluoroacetic acid (2.5 µL) and the samples were evaporated to dryness.

Peptide Desalting: Each sample was then reconstituted in a solution of trifluoroacetic acid (500 µL, 0.1% TFA in H_2O) and desalted on Waters Sep-Pak C18 cartridges (part number: WAT054960) connected to a vacuum manifold. The cartridges were first conditioned, equilibrated, then the peptides were loaded onto the cartridges and allowed to bind for 10 min. Peptides were then washed, desalted and then eluted in fresh 1.5 mL low-binding Eppendorf tubes. The following table shows the solutions used for the desalting process. For the peptide elution step, the cartridges were allowed to sit with the solution for 10 min before collection.

Step	Solution	Total Washes
1) Cartridge Conditioning	90% MeOH in H_2O + 0.1% TFA	3 × 1 mL
2) Cartridge Equilibration	0.1% TFA in H_2O	3 × 1 mL
	**now add digested peptides to cartridges**	
3) Peptide Washing	H_2O + 0.1% TFA	3 × 1 mL
4) Peptide Desalting	5% MeOH in H_2O + 0.1% TFA	3 × 1 mL
5) Peptide Elution	80% MeCN in H_2O + 0.1% TFA	2 × 500 µL

Desalted peptides were evaporated to dryness overnight in a vacuum concentrator and stored at –80 °C prior to timsTOF LC-MS/MS analysis.

timsTOF LC-MS/MS Sample Preparation: Lyophilised desalted peptides were reconstituted in an acetonitrile solution (16 µL, 4% MeCN with 0.1% formic acid). A 1 µL aliquot was taken for a NanoDrop Spectrophotometer reading to determine peptide concentration under the setting 1 Abs = 1 mg/mL. From this, an appropriate injection volume was established (typically between 1 and 3 µL). The resulting Brucker (.d) data files were then analysed on FragPipe and Perseus (see Sections 5.1.5 and 5.1.6).

2.5.3 Tandem Liquid Chromatography Mass Spectrometry (LC-tims-MS/MS) for Proteomics.

Tryptic peptides were separated using a nanoElute LC sample analyser (Bruker Daltonik GmbH, Germany) equipped with a two-column separation stage. The trap column was a Thermo Trap cartridge (5 mm) followed by an IonOpticks Aurora Elite C18 analytical column (150 mm, 0.1 mm, 1.7 µm particle diameter) held at 40 °C, with a flow rate of 0.5 µL/min and injection volume of 2 µL loaded at 84.7 bar. Mobile phase A was 0.1 % formic acid dissolved in MS-grade water, and mobile phase B comprised 0.1 % formic acid dissolved in MeCN. The gradient was as follows: initial 5 % B over 5 minutes, 10-40 % B over 30 minutes, to 95 % B in 2 minutes, followed by 8 minutes re-equilibration. The ESI module used a BrukerCaptive spray ionisation interface for the Bruker ZDV sprayer (20 µm). Mass detection was performed using a quadrupole to select precursor ions and a time-of-flight (TOF) analyser for spectra acquisition, which are coupled to a trapped ion mobility separation (TIMS) device for an additional dimension of ion separation. Ordinary collision-dissociation in the reaction cell was applied by nitrogen gas at 6 bar. The MS data were acquired under data-dependent acquisition mode (dda) using 10 parallel accumulation and serial fragmentation (PASEF) scans (1.16 s cycle time) in a mass range of 100-1,700 m/z under positive mode within a charge set from 0 as minimum and 5 as maximum. A capillary voltage of 1,500 V at 180 °C and 3.0 L/min of dry gas flow was applied in the source, and 0.60-1.60 V s/cm2 (1/K0) with a ramp and accumulation time of 100 ms at a rate of 9.52 Hz, and 100 % duty cycle for the TIMS settings. The Bruker tims control v3.1 and Hystar v6.030.0 were used as the data acquisition software. The raw Bruker TDF datasets (.d) were used as input in FragPipe (22.0) software

against the human (Homo sapiens) reference proteome added of common contaminants and decoys (Uniprot ID: UP000005640, 83,607 reviewed and unreviewed proteins).

3. Computational Experimental

3.1 KNIME Workflow for the Selection of Structurally-Diverse Substrates

KNIME version 4.7.7 with Chemistry Add-Ons was used to create a workflow for the identification and selection of structurally diverse libraries of commercially available substrates. The first six nodes (Supplementary Figure 9) were connected sequentially with the following settings:

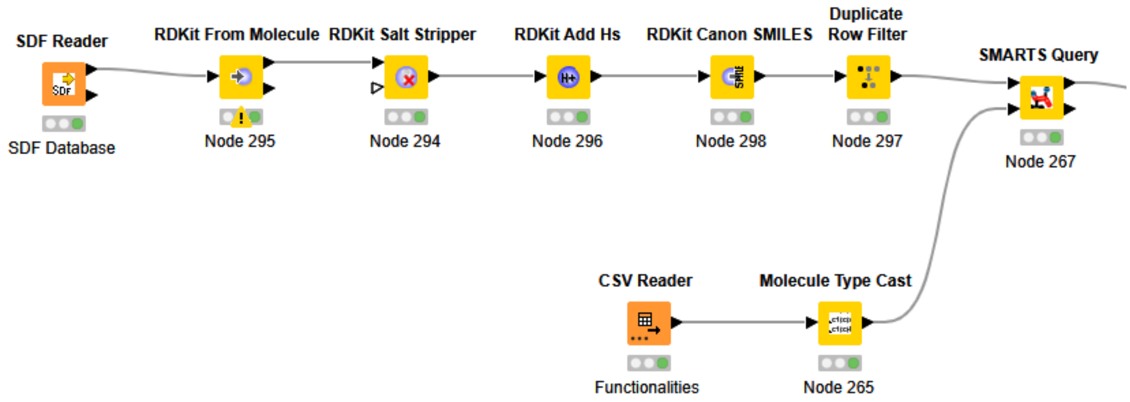

Supplementary Figure 9: Showing the nodes used in the first section of the KNIME workflow, up to the SMARTS Query node.

- **SDF Reader** node was used to read in-house chemical inventories as .sdf files with *Extract SDF blocks* checked.
- **RDKit from Molecule** node with *Molecule column* set to *SDF Molecule* and *Error Handling* set to *Send erroneous rows to second output*. All other node settings remained unchanged.
- **RDKit Salt Stripper** node was added to the workflow, *RDKit Mol column* was configured to read *RDKit Mol* with *Keep only largest fragment after salt stripping* checked. All other settings remained unchanged.
- **RDKit Add Hs** was then connected to the **RDKit Salt Stripper** node and made to read the *salt stripped molecule* column, this was done by selecting *salt stripped molecule* in the dropdown menu under RDKIT Mol column. All other settings remained unchanged.
- **RDKit Canon SMILES** node was made to read the *Molecule (RDKit Mol)(Added Hs)* column by selecting the corresponding column from the dropdown menu under RDKit Mol Column, all other settings were left unchanged.
- Duplicates were then removed with the **Duplicate Row Filter** node. *Manual Selection* and *enforce exclusion* were checked, the green *include* box was made to contain *Canonical SMILES*.

The **Duplicate Row Filter** node was then connected to the first input port of a **SMARTS Query** node to select molecules by functional groups. The second input port was connected to a **Molecule Type Cast** node and a **CSV Reader** node (Supplementary Figure 9). The following settings were used for the four nodes:

- The **CSV reader** node was made to read a .csv file containing SMARTS pattern shown in the table below.

**SMARTS**	**What is it?**
[HC](C)(C)C	aliphatic compounds with tertiary C-H bond
[H2C][CH2](C)	aliphatic compounds with secondary C-H bond
[#6]=[#6]	aliphatic alkenes

The following settings were used: Under *Input Location > Read from > Local File System,* and *Mode > File* was checked. Under *Reader Options > Format > Autodetect format* was selected and the following symbols were used: *Column delimiter contained a* **,** *(comma)* with *Row delimiter* checked, *quote char* and *quote escape char* both contained **"** *(double quote), Comment char* in *Reader Options contained* **#** *(hash). Has column header* was checked. All other settings remained unchanged.

- The **Molecule Type Cast** node was made to read *SMARTS* by using the dropdown menu under *Structure Column. Structure Type* was made to read *Smarts* by using the dropdown menu. The **Molecule type cast** node was then connected to the second input port of the **SMARTS query** node.

- The **SMARTS query** node was configured as follows: Under *Source > Molecule > Canonical SMILES* was selected from the dropdown menu and *SMARTS > SMARTS* was selected from the dropdown menu. Under *parameters* both boxes were left unchecked. All other settings were left unchanged.

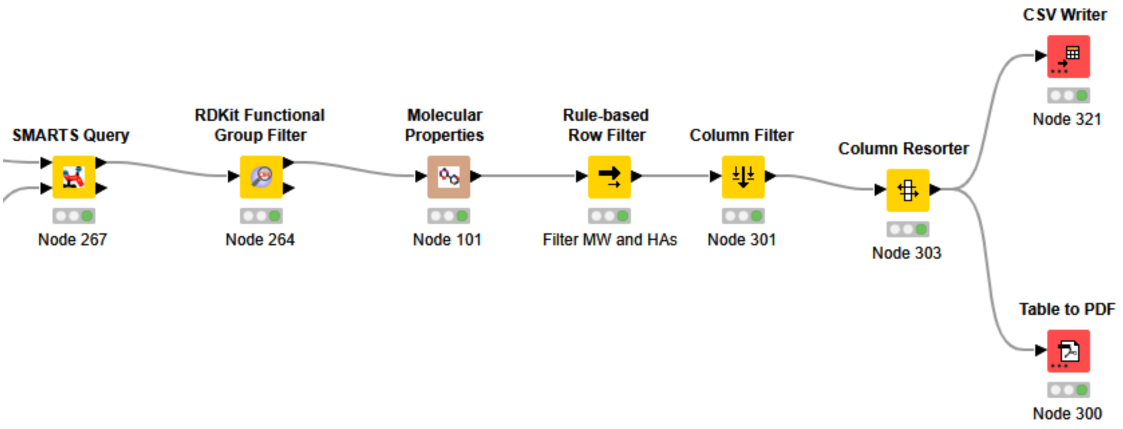

Supplementary Figure 10: Showing the nodes used in the second and final section of the KNIME workflow from SMARTS Query to the final writer nodes.

The SMARTS query node was then connected to a further sequence of five nodes with the following settings:

- Undesired functionalities were removed with the **RDKit Functional Group** filter (Supplementary Figure 10) with the following settings: *Select molecule column > RDKit Mol Column > Salt Stripped Molecule*; A bespoke functional group definition file was used (see Supplementary Table 2) and *Enable recording in the following new column: First Non-Matching Pattern* was checked.
- Molecules were then filtered by molecular properties using the **Molecular Properties** node. This was configured as follows: Under *Column Selection > SDF Molecule* was selected from the dropdown menu*; Enforce exclusion* was checked under the red *Exclude* box. The green *Include* box contained the following molecular properties: *Aromatic Atoms Count, Molecular Weight, No. of Heavy Atoms, Rotatable Bonds Count, Rotatable Bonds Count (non terminal), XLogP*. All other settings remained unchanged.
- A **Rule-based Row Filter** was then connected to the workflow, in *Expression* the following rules were added*:*

  $Heavy Atoms Count$ ≤ 8 => TRUE

  $Heavy Atoms Count$ ≥ 18 => TRUE

$XLogP$ ≥ 3.00 => TRUE

$XLogP$ ≤ -3.0 => TRUE

$Rotatable Bonds Count (non terminal)$ ≥ 2 => TRUE

All other settings remained unchanged.

Exclude TRUE matches was checked.

- Four optional nodes were then added which included a **Column Filter** node to select which columns to include in the output data file. A **Column Resorter** node to reorder the columns as desired. Lastly, a **CSV Writer** node and a **Table to PDF** node were added to complete the workflow and save a generated list of 826 substrates which were then clustered into 100 clusters.

Active	Functional Group	SMARTS Pattern
☐	AcidChloride	C(=O)Cl
☑	Aromatic Acid Chloride	[$(C-!@[a])](=O)(Cl)
☑	Aliphatic Acid Chloride	[$(C-!@[A;!Cl])](=O)(Cl)
☑	CarboxylicAcid	C(=O)[O;H,-]
☑	Aromatic Carboxylic Acid	[$(C-!@[a])](=O)([O;H,-])
☑	Aliphatic Carboxylic Acid	[$(C-!@[A;!O])](=O)([O;H,-])
☑	Alpha Amino Acid	[$(C-[C;!$(C=[!#6])]-[N;!H0;!$(N-[!#6;!#1]);!$(N-C=[O,N,S])])](=O)([O;H,-])
☑	SulfonylChloride	[$(S-!@[#6])](=O)(=O)(Cl)
☑	Aromatic Sulfonyl Chloride	[$(S-!@c)](=O)(=O)(Cl)
☑	Aliphatic Sulfonyl Chloride	[$(S-!@C)](=O)(=O)(Cl)
☐	Amine	[N;!H0;$(N-[#6]);!$(N-[!#6;!#1]);!$(N-C=[O,N,S])]
☑	Primary Amine	[N;H2;D1;$(N-!@[#6]);!$(N-C=[O,N,S])]
☑	Primary Aromatic Amine	[N;H2;D1;$(N-!@c);!$(N-C=[O,N,S])]
☑	Primary Aliphatic Amine	[N;H2;D1;$(N-!@C);!$(N-C=[O,N,S])]
☐	Secondary Amine	[N;H1;D2;$(N(-[#6])-[#6]);!$(N-[!#6;!#1]);!$(N-C=[O,N,S])]
☐	Secondary Aromatic Amine	[N;H1;D2;$(N(-[c])-[#6]);!$(N-[!#6;!#1]);!$(N-C=[O,N,S])]
☑	Secondary Aliphatic Amine	[N;H1;D2;$(N(-C)-C);!$(N-[!#6;!#1]);!$(N-C=[O,N,S])]
☐	Aromatic Amine	[N;!H0;$(N-c);!$(N-[!#6;!#1]);!$(N-C=[O,N,S])]
☐	Aliphatic Amine	[N;!H0;!$(N-c);$(N-C);!$(N-[!#6;!#1]);!$(N-C=[O,N,S])]
☐	Cyclic Amine	[N;!H0;R;$(N-[#6]);!$(N-[!#6;!#1]);!$(N-C=[O,N,S])]
☑	Boronic Acid	[$(B-!@[#6])](O)(O)
☑	Aromatic Boronic Acid	[$(B-!@c)](O)(O)
☑	Aliphatic Boronic Acid	[$(B-!@C)](O)(O)
☑	Isocyanate	[$(N-!@[#6])](=!@C=!@O)
☑	Aromatic Isocyanate	[$(N-!@c)](=!@C=!@O)
☑	Aliphatic Isocyanate	[$(N-!@C)](=!@C=!@O)
☑	Alcohol	[O;H1;$(O-!@[#6;!$(C=!@[O,N,S])])]
☑	Aromatic Alcohol	[O;H1;$(O-!@c)]
☑	Aliphatic Alcohol	[O;H1;$(O-!@[C;!$(C=!@[O,N,S])])]
☑	Aldehyde	[CH;D2;!$(C-[!#6;!#1])]=O

☑	Aromatic Aldehyde	[CH;D2;$(C-!@[a])](=O)
☑	Aliphatic Aldehyde	[CH;D2;$(C-!@C)](=O)
☐	Halogen	[$([F,Cl,Br,I]-!@[#6]);!$([F,Cl,Br,I]-!@C-!@[F,Cl,Br,I]);!$([F,Cl,Br,I]-[C,S](=[D1;O,S,N]))]
☐	Aromatic Halogen	[F,Cl,Br,I;$(*-!@c)]
☑	Aliphatic Halogen	[$([F,Cl,Br,I]-!@C);!$([F,Cl,Br,I]-!@C-!@[F,Cl,Br,I])]
☐	Not Fluorine Halogen	[$([Cl,Br,I]-!@[#6]);!$([Cl,Br,I]-!@C-!@[F,Cl,Br,I]);!$([Cl,Br,I]-[C,S](=[D1;O,S,N]))]
☑	Aliphatic Not Fluorine Halogen	[$([Cl,Br,I]-!@C);!$([Cl,Br,I]-!@C-!@[F,Cl,Br,I]);!$([Cl,Br,I]-[C,S](=[D1;O,S,N]))]
☑	Aromatic Not Fluorine Halogen	[$([Cl,Br,I]-!@c)]
☑	Bromine Halogen	[$([Br]-!@[#6]);!$([Br]-!@C-!@[F,Cl,Br,I]);!$([Br]-[C,S](=[D1;O,S,N]))]
☑	Aliphatic Bromine Halogen	[$(Br-!@C);!$(Br-!@C-!@[F,Cl,Br,I]);!$(Br-[C,S](=[D1;O,S,N]))]
☑	Aromatic Bromine Halogen	[$(Br-!@c)]
☑	Azide	[N;H0;$(N-[#6]);D2]=[N;D2]=[N;D1]
☑	Aromatic Azide	[N;H0;$(N-c);D2]=[N;D2]=[N;D1]
☑	Aliphatic Azide	[N;H0;$(N-C);D2]=[N;D2]=[N;D1]
☐	N-Carbonyl Aliphatic Amine	*C(=O)N(C(*)([H])[H])C(*)([H])[H]
☐	N-Boc Aliphatic Amine	*C([H])([H])N(C(=O)OC(C)(C)C)C(*)([H])[H]
☐	N-Acyl Aliphatic Amine	*C([H])([H])N(C(=O)C([H])([H])[H])C(*)([H])[H]
☐	N-Chloroacetamide Aliphatic Amine	*C([H])([H])N(C(=O)C[Br,I,Cl,F])C(*)([H])[H]
☑	Anhydride	[CX3](=[OX1])[OX2][CX3](=[OX1])
☑	Imide	[CX3](=[OX1])[NX3H0]([NX3H0]([CX3](=[OX1]))[CX3](=[OX1]))[CX3](=[OX1])
☑	Nitro	[$([NX3](=O)=O),$([NX3+](=O)[O-])][!#8]
☑	N-oxide	[$([#7+][OX1-]),$([#7v5]=[OX1]);!$([#7](~[O])~[O]);!$([#7]=[#7])]
☑	Aldehyde/formamide	[CX3H1](=O)[#6,#7]
☑	Imine	[$([CX3]([#6])[#6]),$([CX3H][#6])]=[$([NX2][#6]),$([NX2H])]
☑	Imide2	[CX3](=[OX1])[NX3H][CX3](=[OX1])
☑	Imide3	[CX3](=[OX1])[NX3H0]([#6])[CX3](=[OX1])
☑	Nitrate	[$([NX3](=[OX1])(=[OX1])O),$([NX3+]([OX1-])(=[OX1])O)]
☑	Isonitrile	[CX1-]#[NX2+]
☑	Hydrazine	[NX3][NX3]
☑	Hydrazone	[NX3][NX2]=[*]
☑	DiazoNitrogen	[$([#6]=[N+]=[N-]),$([#6-]-[N+]#[N])]
☑	Ketone	[#6][CX3](=O)[#6]
☑	Deuterium	[2H]
☑	13C	[13c,13C]
☑	15N	[15n,15N]
☑	Alkyl sulfide	[C][#16X2H0]
☑	Thiourea	[NX3][CX3]=[SX1][#6]
☑	Thioamide	[NX3][CX3]=[SX1]
☑	Carboxylate	[CX3](=[OX1])O
☑	aromatic lactone	[cX3](=[OX1])[oX2]
☑	aromatic enone	ccc=O
☑	acrylamide	[CX3]=C[CX3](=[OX1])[NX3]
☑	Oxime	[CX3](=N-O)
☑	Nitroso	[NX2](=O)
☑	Hydroxylamine	[NX3][OX2]

☑	Enamine	[n,NX3][CX3]=[CX3]
☐	Sulfone	[$([#16X4](=[OX1])(=[OX1])([#6])[#6]),$([#16X4+2]([OX1-])([OX1-])([#6])[#6])]
☑	Sulfinate	[$([#16X3](=[OX1])[OX2H0]),$([#16X3+]([OX1-])[OX2H0])]
☑	Sulfoxide	[$([#16X3]=[OX1]),$([#16X3+][OX1-])]
☑	Sulfonic acid and ester	[OX2,-]S(=O)(=O)(*)
☑	Thiol	[#16!H0]
☑	Isothiocyanate	[NX2]=[C]=[S]
☑	Azo	[NX2]=[NX2]
☑	Substituted alkene	[CX3]=C(C)(C)
☑	Amidine	[NX3][CX3]=[NX2]
☑	pyridinium eg	[n+]
☐	Chiral1	C[C@H](*)(*)
☐	Chiral2	C[C@@H](*)(*)
☐	Cyclic alkene removal	C[CX3]=C(c)(C)
☑	alkenyl halide	[F,Cl,Br,I][CX3]=[CX3]
☑	2-Halo pyridine/pyrimidine	c1([F,Cl,Br,I])[c,n]ccc[nH0]1
☑	2-Halo hetarenes	nc([F,Cl,Br,I])[c,n]
☑	1,2-diaminoarene	c([N;H2,H1])c[N;H2,H1]
☑	1,2-catechol	c([OH])c[OH]
☑	1,3-catechol	c([OH])[c,n]c[OH]
☑	1,3-diaminoarene	c([NH2])[c,n]c[NH2]
☑	1,4-catechol	c([OH])ccc[OH]
☑	thione	[#6]=S
☑	aniline (only)	cc([NH2])c
☑	ene-nitrile	[CX3]=[CX3]-C#N
☐	Substituted amide	[NX3][CX3]=[OX1]
☐	ethyl chain	[*][CH2][CH3]
☐	propyl chain	[*][CH2][CH2][CH3]
☑	sulfonyl halide	[$(S-!@[#6])](=O)(=O)[I,Br,Cl,F]
☑	zinc	[Zn]
☑	magnesium	[Mg]
☑	lithium	[Li]
☑	tin	[Sn]
☑	copper	[Cu]
☑	silicon	[Si]
☑	palladium	[Pd]
☑	boron	[B]
☑	Ruthenium	[Ru]
☑	Germanium	[Ge]
☑	1,2-dicarboxylic acid	c([$(C-!@[a])](=O)([O;H,-]))c([$(C-!@[a])](=O)([O;H,-]))
☑	1,3-dicarboxylic acid	c([$(C-!@[a])](=O)([O;H,-]))cc([$(C-!@[a])](=O)([O;H,-]))
☑	1,4-dicarboxylic acid	c([$(C-!@[a])](=O)([O;H,-]))ccc([$(C-!@[a])](=O)([O;H,-]))
☑	1,5-dicarboxylic acid	c([$(C-!@[a])](=O)([O;H,-]))cccc([$(C-!@[a])](=O)([O;H,-]))
☐	alkene	[c,n,C,N]C=C
☑	alkyne	[$([CX2]#C)]
☑	Hypervalent iodine (with I=O)	[I]=O
☑	Lactone	c[c,C](=O)O[c,C]
☑	Aromatic Urea	[#7][#6](=O)[#7]
☑	Urea	[NX3]C(=O)[NX3]
☑	Acetylenic Ester	[$([CX2]#C)]C(=[OX1])[OX2]C

☑	1,5 Hexadiyne	[$([CX2]#C)]CC[$([CX2]#C)]
☑	2,4 Hexadiyne	[$([CX2]#C)]C[$([CX2]#C)]
☑	Hexadiyne 2.0	[$([CX2]#C)][$([CX2]#C)]
☑	Heptdaiyne (O,N,C)	[$([CX2]#C)]C*C[$([CX2]#C)]
☑	1,5-diene	C=CCCC=C
☐	Phenyldialkyne 1,3	[$([CX2]#C)]ccc[$([CX2]#C)]
☐	Phenyldialkyne 1,4	[$([CX2]#C)]cccc[$([CX2]#C)]
☑	1,3-diene	C=CC=C
☑	1,4 diene	C=CCC=C
☑	Epoxide	C2OC2
☑	NH2	[NH2]
☐	Ene-dione	[CX3](=O)C=C[CX3](=O)
☑	Dihydropyrrole	C1C=CC[NH]1
☑	Acetal	CO[CX4]OC
☑	Pyran	Brcc([OX2])c
☑	Alpha-halo acid	[F,Cl,Br,I]cc([$(C-!@[a])](=O)([O;H,-]))
☑	Diacrylamide	C=CC(=O)N1CCN(C(=O)C=C)CC1
☑	Aldehyde 2.0	[CH](=O)[#6,#7]
☐	Amine Aromatic NH	[n;H1]
☑	Aliphatic NH	[C][NH1][C]
☐	Aniline NH	[c][NH1][*]
☐	Nitrile	[*][C](#N)
☑	Sulfomyl Chloride	[N][S](=O)(=O)(Cl)
☑	alpha Keto-Halides	[*][CX3](=[OX1])[#6][F,Cl,Br,I]
☑	Oxime 2	[#6][N](-O)[#6]
☑	Oxadiazole	[#6]N(-N=O-C(=C)-O)
☑	Methane Diamine	[#6][N][C][N][#6]
☑	Aromatic Methyldiamine	[n][C][N]
☑	Vinyl S(VI)	[C]=[C][S](=O)(=O)[#6,n,N]
☑	Enol Ethers	[C]=[C][O][#6]
☑	Imidates	[N]=[#6][O]
☑	Quat. Nitrogens	[$([NX4+])]
☑	Tertiary Sulfur	[S+](C)C
☑	Halogenated Succinamide	[N][Cl,Br]
☑	Phosphate Group	[OH][P](=O)[OH]
☐	Sulfonamide	[N][S](=O)(=O)
☐	beta-Keto Amide	[*][NH][#6](=O)[#6][#6](=O)[*]
☐	aplha-keto amide	[*][NH][#6](=O)[#6](=O)[*]
☐	DiAmine	[#7X3;H2,H1;!$(NC=O)].[#7X3;H2,H1;!$(NC=O)]
☐	Aromatic Diamine	[N][n]
☐	Sulfonamide	[#7][#16X4](=[OX1])=[OX1]

Supplementary Table 2: Bespoke list of functional groups used for the RDKIt Functional Group filter. The list was used as a tab delimited .txt file (in the format Name\tSmarts\tLabel) to filter compounds. Functional groups that are checked did not pass the filter and were removed by the node.

To aid substrate selection and limit bias a clustering workflow was integrated to group compounds with structurally similarity. This second workflow (Supplementary Figure 11) continued directly from the workflow described previously and was created with the following nodes and settings.

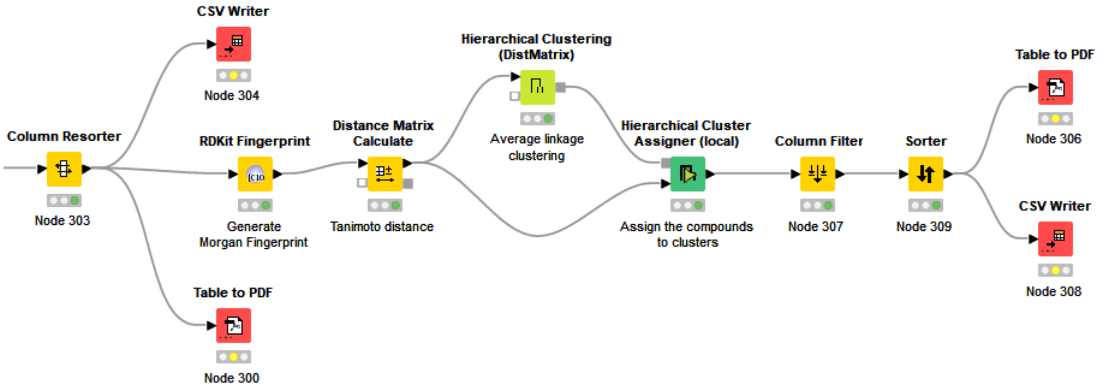

Supplementary Figure 11: Showing the KNIME workflow used for the hierarchical clustering of compounds.

- An **RDKit Fingerprint** node was connected to the **Column Resorter** node to generate Morgan Fingerprints of each compound. The following settings were implemented, under *Fingerprint type* > *Morgan* was selected using the dropdown menu. Under *RDKit Mol column* > *Salt Stripped Molecule* was selected from the dropdown menu. All other settings remained unchanged.
- The **Distance Matrix Calculate** node was then added to the workflow with *Distance selection* > *Tanimoto* selected from the dropdown menu. The green *include* box contained *MFP2* and *Enforce exclusion* was checked under the red *Exclude* box. *Chunk size* was set to 1,000. All other settings remained unchanged.
- A **Hierarchical Clustering (DistMatrix)** node was connected to the **Distance Matrix Calculate** node with *Distance matrix column* > *Distance* selected from the dropdown menu, *Linkage type* > *Average Linkage* selected from the dropdown menu and *Ignore missing values* checked. All other settings remained unchanged.
- A **Hierarchical Cluster Assigner (local)** node was connected to the **Distance Matrix Calculate** and the **Hierarchical Clustering (DistMatrix)** nodes. The Assigner was configured as follows, *cluster count* was checked under *Assign clusters based on,* and *Number of clusters* was set to *100*.
- Finally **Column Filter** and **Resorter** nodes were added together with **Table to PDF** and **CSV Write** nodes to save the generated clusters. Compounds were then manually picked form each cluster and used as substrates in rhodium catalysed nitrenoid reactions (see Supplementary Table 3).

Number	SMILES	Number	SMILES
1	S(c1ccc(F)cc1)(N2CCCC2)(=O)=O	25	C1CN(CCC1(F)F)C
2	c1cnc2c(n1)CCCC2	26	O=S(N1CC=CC1)(c2ccc(C)cc2)=O
3	C(CC1CCCCC1)#N	27	c1ccn(c1)CCC#N
4	c1c(ccc(c1)OC)C=C	28	c12c(NC(C)(C)C=C1C)cc(OC)cc2
5	C1CCCC(C1)N(C)C	29	n1c(n[nH]c1)C2CCCC2
6	C1CN(C(CCC1)=O)C	30	c1cnc2c(c1OC)CCC(N2)=O
7	s1c(ncc1)CC(C)C	31	C1CCN(C1)C(C)C
8	O1CCC(CC1)C#N	32	n1ccc(cc1)-c2noc(n2)C3CC3
9	C(CCn1ccnc1C)#N	33	C(N(C)C)(CC(C)C)=O
10	n12c(nnc1)CCC2	34	N1(CCCc2cc(ccc12)C#N)C(=O)C
11	S1(NCCCC1)(=O)=O	35	c12c(NC(=O)CC1)ccc(c2)OC
12	s1cnc(c1C=C)C	36	O=C(C1CCCCC1)N(C)C
13	[nH]1nnnc1/C=C/c2ccccc2	37	N1CCCc2ccc(cc12)C(F)(F)F
14	c12c(n[nH]c1CCCC2)C(F)(F)F	38	c1(cc2c(cc1)OCC(N2CCC#N)=O)C
15	n1cn(CC=C)cc1	39	O1c2c(OCCC1)cccc2
16	C(=O)(N1CCCC1)c2ccc(cc2)F	40	N#Cc1cc(OC2CCCCC2)ncc1
17	N1(CC=CC1)c2ccccc2	41	[nH]1nnnc1N2CCCCC2
18	O=C1CCCc2c(ccs2)N1	42	c12n(ccn1)CCCN2
19	s1c(nc2c1cccc2)CCC	43	S1(N(CCC1)C(C)C)(=O)=O
20	c1n(ncn1)C2CCCC2	44	CC(Cn1cccn1)C
21	n1ccc(cc1)C2CCOCC2	45	c1c(cc2c(c1)C=CC(O2)(C)C)OC
22	N1(CCCCC1)c2cccs2	46	c1cccc(c1)CCC#N
23	C1CCN(C1=O)Cc2ccccc2	47	C(\CC#N)=C/CC#N

24	Cc1cc(C)nn1CC=C		

Supplementary Table 3: Selected substrates, as SMILES strings, used in the rhodium catalysed nitrenoid synthesis of structurally diverse reactive fragment libraries.

3.2 FragPipe Processing of Pull-down Proteomics Data

Fragpipe (version 22.0) with MSFragger, IonQuant, diaTracer, DIA-NN, Python, Database Splitting and Spectral Library Generation add-ins was used to identify proteins found in pull-down proteomics samples.

- timsTOF LC-MS/MS data for samples treated with the corresponding reactive fragment as a .d file was uploaded in *Workflow* > *Input LC-MS Files* and *IM-MS (ion mobility, timsTOF only)* was checked.

The built-in workflow LFQ-MBR was used with the following modifications:

- Under *Workflow* > *Select a workflow, LFQ-MBR* was selected from the dropdown menu, the workflow was loaded by clicking *Load workflow*
- Under the *Database* tab the appropriate .fasta file was uploaded from UniProt (UP000005640 Homo sapiens (Human)) and decoys were added by clicked the *Add decoys* button.
- In the *MSFragger* tab *Run MSFragger* was checked, and in *Common Options* > *Peak Matching* > *Precursor mass tolerance*, *Da* was selected from the dropdown menu and the range was set to *-150 - 1,000* and *Fragment mass tolerance* was set to *20 PPM*. *Protein Digestion* under *Enzyme name 1, trypsin* was added. Under *No Cuts 1*, *P* was added and under *Mixed Cleavages 1*, *2* was added. Other settings in this section remained unchanged. Under *Modifications* > *Variable Modifications*, Cysteine was added and checked. This was achieved by inserting *C* under *Site (editable)*, *57.02146* under *Mass* (*editable*) and *3* was selected under the *Max occurrences* (*editable*) column. Under *Fixed Modifications, Cysteine* remained unchecked in the available menu.
- Under the tab *Validation* > *Crystal-C* was unchecked together with *RunMSBoost* under *Rescoring Using Deep Learning Prediction*.

- Under *Quant (MS1)* > *Advanced Options* > *Match between runs (MBR)* > *MBR top runs* was set to 2.

All other settings remained unchanged and the software was made to run by clicking *RUN* in the *Run* tab.

3.3 Perseus Processing of FragPipe data

Perseus (version 2.0.7.0) was used analyse data outputs from FragPipe with the following workflow;

- The combined_protein.tsv, found in the FragPipe output files, was dragged and dropped into the Perseus interface. *MaxLFQ Intensity* of relative samples were selected and moved to the column *Main* using the provided arrows. *Protein*, *Protein ID*, *Description* and *Gene* were added to the *Text* column in the same manner. All other settings remained unchanged and the data was loaded onto the software by clicking *Done*. This generated *matrix1*.
- Contaminants were removed by selecting the *Filter rows based on text column* in the *Filter rows* drop-down menu. In the new interface under *Search string, contam* was added. The function was executed by clicking *OK* to generate *matrix2*.
- A log_2 transformation was then carried out on the data by selecting the *transform* icon f(x). The function was executed by clicking *OK* to generate *matrix* 3. *Matrix3* was processed further by selecting *Filter rows based on valid values,* under *Min*. *Valids* > *Min*. *Number* was set to 3. All settings remain unchanged and the function was executed.

Missing values in the dataset were then replaced by using the *Replace missing* values *from normal distribution* function. Finally, experimental replicates were then combined using *Annot. rows* > *Categorical annotation rows*. IAA-treated control samples were combined separately to samples treated with the competitors followed IAA. To visualize the results either depletion or enrichment of the proteome, a t-test and volcano plot was generated by selecting the appropriate icon. For the visualisation of depleted proteins, under *Grouping* > *First group (right)*, the competition sample was selected. In the *Second group (left)* the IAA-treated controls were selected to generate the volcano plot.

3.4 FragPipe Workflow for Aurora A Kinase Peptide Mapping

Fragpipe (version 22.0) was used to localise modifications within peptides generated from bottom-up peptide mapping workflows on Aurora A kinase. MSFragger, IonQuant, diaTracer, DIA-NN, Python, Database Splitting and Spectral Library Generation add-ins were downloaded.

An initial search was carried out by modification of the factory-installed workflow 'Open' with the following settings:

- timsTOF LC-MS/MS data for Auora A kinase samples treated with the corresponding reactive fragment as a .d file was uploaded *Workflow* > *Input LC-MS Files, IM-MS (ion mobility, timsTOF only)* was checked
- Under *Workflow* > *Select a workflow, Open* was selected from the dropdown menu, the workflow was loaded by clicking *Load workflow*
- Under the *Database* tab the appropriate .fasta file was uploaded from UniProt (UP000005640 Homo sapiens (Human)) and decoys were added by clicked the *Add decoys* button.
- Under the *MSFragger* tab *Run MSFragger* was checked, and in *Common Options* > *Peak Matching* > *Precursor mass tolerance*, *Da* was selected from the dropdown menu and the range was set to *-150 - 1,000* and *Fragment mass tolerance* was set to *20 PPM*. *Protein Digestion* under *Enzyme name 1, trypsin* was added. Under *No Cuts 1*, *P* was added and under *Mixed Cleavages 1*, *2* was added. Under *Enzyme name 2, lysc* was added. Other settings in this section remained unchanged.
    - In *Modifications* > *Variable Modifications* and *Fixed Modifications* all boxes remained unchecked. Other settings in this section remained unchanged.
    - In *Advanced Options* and *Spectral Processing* all settings were left unchanged.
    - In *Open Search Options, Yes, keep delta mass* was selected from the dropdown menu under *Report mass shift as a variable mod* and *Localize mass shift (LOS)* was checked.
    - In *Advanced Peak Output Options*, *Write calibrated mzML* was checked. All other settings in this tab remained unchanged.
- Under *Validation* > *Crystal-C* was unchecked and in the same tab under *FDR Filter and Report, Remove contaminants was checked*. All other settings in this tab remained unchanged.
- All other settings remained unchanged and the software was made to run by clicking *RUN* in the *Run* tab.

The output data was then analysed manually by opening the psm.tsv file and looking for the mass of the incubated small molecule in the *Delta Mass* column. Observed mass modifications were then

added to a new workflow for label-free quantification. Datasets for triplicate control samples were run with triplicate samples treated with a corresponding compound. This was repeated for all 6 reactive fragments tested. The previous workflow was modified as follows for LFQ quantification of labelled peptides:

- Under the *MSFragger* > In *Common Options* > *Peak Matching* > *Precursor mass tolerance*, *Da* was changed to *-10 - 10* and *Fragment mass tolerance* was set to *20 PPM*. *Isotope error* was set to *0/1*. Other settings in this section were left unchanged.
    - Under *Modifications* > *Fixed modification*: *C (cysteine), S (serine), T (threonine), K (lysine), H (histidine), R (arginine), Y (tyrosine)* were unchecked. All other settings remained unchanged. Fragpipe was then run with the following modified settings.
    - Under *Advanced options* > *Mass Offsets* the following masses were added in the appropriate text box: -105.0248 -89.0299 -33.9877 -32.0085 -30.0106 -18.0106 -17.0265 -2.0157 -1.007825 -0.984 0 0.984 3.9949 12.0 13.9793 14.0157 15.9949 19.9898 21.969392 21.9819 23.95806 26.0157 27.9949 28.0313 28.990164 29.9742 31.972071 31.9898 37.9469 37.955882 42.0106 42.047 43.0058 43.9898 44.985078 47.9847 53.9193 57.02146 58.0055 61.9135 61.921774 68.026215 70.041865 71.0371 79.9568 79.9663 86.000394 100.016 114.042927 119.004099 128.095 146.0579 156.1011 162.0528 173.051 176.0321 178.0477 183.035399 189.046 203.0794 204.1878 210.1984 228.111 229.014009 238.2297 301.9865 340.1006 349.1373 365.1322 365.1322 406.1587 541.06111 [input fragment mass shift]. The corresponding mass change of the reactive fragment was added at the end of the list.
- Fragment mass shifts = **F7**: 310.0062; **F13**: 400.0433; **F43**: 400.0578; **F100**: 324.0578; **F101**: 388.00660; **F104**: 402.0006.
- Under *PTMs* > *PTM Profiling*, *Max fragment charge* was set to *2*, *Precursor tolerance* was set to *8 PPM*, Peak picking with was set to *3 PPM*. Under *Annotation* > *Annotation Source*, *Common mass shifts* was selected.
- For Label-free Quantification, under *Quant (MS1)* > *MS1 Quantification*, *Run MS1 quant* and *IonQuant* were selected. Under *Common, match between runs (MBR)* was checked and *Normalise intensities across runs* was checked. Under *Advanced options* > *Match between runs (MBR)* > *MBR Top runs* was set to *2*
- All other settings remained unchanged, and the software was run.

For this search, peptide data was visualised using the built-in tool under the *Run* tab and by clicking the *Open Fragpipe-PDV viewer* option. The output *combined_ion.tsv* file was then processed to

calculate the total intensities of modified residues and % modification relative to the total amount of modified peptides. Only peptides with robust MS2 fragmentation and reliable localisation of modified amino acid residues were included in calculations.

3.5 MSConvert and UniDec Parameters for the Deconvolution of Protein Mass Spectra

The following workflow was used for the manual deconvolution of intact protein MS data collected from fixed-timepoint incubation reactions with Aurora A kinase and reactive fragments.

- Raw intact protein MS data as .d files were converted to UniDec[2] readable mzML files using ProteoWizard MSConvert (version 3) software[3].
- Generated mzML files of MS spectra were loaded onto UniDec (version 7.02) in the UniChrome function and protein spectra were manually selected in the displayed chromatogram.
- Under *Peak Selection, Extraction, and Plotting* > *Peak Normalization* > *None* was checked and *Extract Normalization* > *None* was checked. All other parameters were left unchanged.
- The selected spectrum was then loaded onto the UniDec function by clicking *Open Selection in UniDec GUI*.
- The mass-to-charge range was set under *Data Processing* > *m/z: 500 to 1800* and *Use Background Subtraction* was checked, the data was then processed by clicking *Process Data*.
- Under *UniDec Parameters* the following parameters were used, *Charge Range: 10 to 100, Mass Range: 30000 to 40000 Da, Sample Mass Every (Da): 1.0*, the protein spectrum was then deconvoluted by clicking *Run UniDec*.
- Under *Peak Selection and Plotting* > *Peak Detection Range (Da)* was set to 50.0 and *Peak Detection Threshold* was set to 0.2 or set accordingly depending on peak height. *Peak Detection* was then clicked.
- Peaks were integrated by going to *Analysis* > *Integrate Peaks* (or ctrl + i).
- The amount of modified protein was calculated as a percentage using the following equation (where 'mod' stands for modified protein and 'unmod' stands for unmodified protein):

$$\%\,mod = \frac{Area_{mod}}{Area_{mod} + Area_{unmod}} \times 100$$

For the rapid deconvolution of kinetic assays, a bespoke script developed by Dr. Lawrence Collins was utilised (https://github.com/lawrencecollins/BafPipe). The script, which runs on Jupyter Notebooks,

rapidly unzips .d data files, converts them to mzML format and then batch processes the spectra on UniDec. Parameters for UniDec can be read by the script and loaded onto UniDec itself *via* an excel spreadsheet. The following table (Supplementary Table 4) incorporates the parameters that were used for these more complex experiments that generated large data sets.

Parameter	Input	Comments
Directory	C:\Users\[..]	
Start Scan	150	
End Scan	155	
Species Unlabelled	33715	Mass of unlabelled protein (Da)
Species Labelled	34068	Mass of labelled protein (Da)
Tolerance (Da)	5	Peak matching tolerance
Config masslb	30000	Deconvolution window low mass
Config massub	40000	Deconvolution window high mass
Config massbins	1	Mass bins for deconvolution - sample mass every
Config peakwindow	10	
Config peakthresh	0.075	
Config minmz	600	m/z lower bounds (defaults to 0)
Config maxmz	1800	m/z upper bounds (defaults to 10e12)
Config startz	1	
Config endz	100	
Config numz	100	
Config numit	60	number of iterations of deconvolution algorithm
Color Unlabelled	red	
Color Labelled	green	

Supplementary Table 4: Parameters used for deconvolution of large datasets using software created by Dr. Lawrence Collins (BafPipe)

3.6 Covalent Docking of Fragments on Aurora A Kinase

Fragments **F50** and **F2** were uploaded onto Schödinger Maestro (Version 14.1.138) as a single .sdf file. Compounds were then prepared for docking using the built-in *LigPrep* function with preset factory settings and pH set to 7.5 ± 0.5. Aurora A kinase (PDB: 5ORL) was then loaded into the software *via* the *Get PDB* function under the File tab. The protein was prepared using the built-in function, *Protein*

Preparation Workflow, with preinstalled factory settings. A small molecule that labelled CYS247 *via* a disulfide bond was manually deleted from the structure. Covalent docking was then performed with the prepared fragments using the covalent docking (*CovDock*) function. Under *Receptor*, *Centroid of selected residue*s was selected with the following residue numbers: 246 (TYR), 190 (HIE), 280 (HIS), 272 (ILE), 275 (PHE), 184 (ILE), 188 (LEU), 193 (ILE), 252 (VAL). Under *Reaction Type* > *Reaction Type*, *Custom (from file)* was selected from the dropdown menu. A bespoke reaction for the substitution of fluorine on the perfluorobenzenesulfonamide with a cysteine thiol was uploaded under *Custom chemistry file*. The resulting bespoke .cdock reaction file contained the following text:

```
# Nucleophilic substitution of a fluoride by a receptor thiol

# Receptor CYS, sulfur is <1>
RECEPTOR_SMARTS_PATTERN 2,[C,c]-[S;H1,-1]

# Ligand, heavily fluorinated aromatic ring, atom for substitution is <2>
LIGAND_SMARTS_PATTERN 1,[c]([F])

# Neutralize the CYS if necessary
CUSTOM_CHEMISTRY ("<1>",("charge",0,(1)))

# Add ligand-receptor single bond
CUSTOM_CHEMISTRY ("<1>|<2>",("bond",1,(1,2)))

# Remove the fluoride leaving group
CUSTOM_CHEMISTRY ("<2>[F]",("delete",2))
```

The docking simulation was then allowed to run. Simulations were run on all five carbon centres on the aromatic pentafluorobenzene ring. Once completed, results for S_NAr reactions between cysteine thiol and reactive fragment on carbon-4 were selected and reprocessed on PyMol.

4. Chemistry Experimental

4.1 Fragment Reactivity

Fragment stability in buffer

Analysis was performed using an Agilent 1290 Infinity II HPLC system (Agilent, Santa Clara, CA, United States), with a diode array detector. Chromatographic separations were performed using an Agilent InfinityLab Poroshell 120 EC-C18 (2.1 x 50 mm, 2.7 um) at a column temperature of 40 °C. The mobile phase used was A - TRIS buffer (95 %) and B - acetonitrile (5%) and the gradient running to 5 % TRIS buffer over 5 minutes at a flow rate of 0.5 mL/min. The DAD recorded the chromatogram at a wavelength of 254 nm.

Compounds **F50** and **F2** (5 µL, 20 mM in DMSO) were transferred to LC-MS vials together with 1,4-dicyanobenzene (5 µ, 20 mM in DMSO) as an internal standard. TRIS buffer was then added (490 µL) so that the final concentration of compound and internal standard was 200 µM with 1% DMSO. Samples were then analysed every 50 minutes over a 24 hour time period.

The UV peak areas corresponding to the compound and internal standard (1,4-dicyanobenzene) were integrated using the instrument software and processed in Excel. For each time point, the total peak area of the compound was divided by the total peak area of the internal standard to give a *Probe/Standard* ratio. This ratio was then normalised to the maximum value observed across the dataset to account for variations in injection volume and detector response. The resulting *Normalised* values were plotted against time and fitted with a first-order exponential decay function (*ExpDec1*) in OriginPro to determine the compound's half-life in buffer ($t_{1/2}$=9.9 hours, see Supplementary Figure 7).

Fragment reactivity with *N*-acetyl cysteine methyl ester

To a 2 mL vial was added sequentially **F51** (1.0 mg, 2.8 µmol, 1.0 equiv.) in DMSO (0.14 mL) and *N*-acetyl-L-cysteine methyl ester (0.5 mg, 2.8 µmol, 1.0 equiv.) in PBS (1.26 mL) (final concentration of both components: 2 mM in 10% DMSO in PBS). The reaction was left at room temperature without agitation, and analysed after 0, 2, 4, 8, and 16 hr by analytical HPLC-MS. The molecular weights of the intermediate and the product were determine by accurate mass spectrometry. For the analysis, a 10

µL aliquot of the reaction at each time point was injected onto an Agilent 1290 Infinity II Preparative and Analytical HPLC system with mass spectrometer (LC/MSD XT) and Diode Array Detector [column: Agilent Technologies Poroshell 120 EC-C18 2.7 µm (3.0 x 50 mm) with guard column; column temperature: ambient; Solvent A: Water with 0.1% formic acid; Solvent B: Acetonitrile with 0.1% formic acid; DAD wavelength: 250 nm; flow rate: 0.8 mL/min]. The following gradient programme was used: 0 min (2% B); 0.5 min (2% B); 6.5 min (98% B); 7.5 min (98% B); 7.6 min (2% B); 8.0 min (2% B).

4.2 General Experimental Considerations

Commercially available starting materials were obtained from Sigma–Aldrich, Fluorochem, Alfa Aesar, BLDpharm, Santa Cruz Biotechnology and Apollo Scientific. Water-sensitive reactions were performed under nitrogen in oven-dried glassware cooled under nitrogen before use. Anhydrous trimethylamine was obtained from a SureSeals bottle supplied by Sigma–Aldrich. All other solvents used were of chromatography or analytical grade. An IKA RV 10 rotary evaporator was used to remove solvents under reduced pressure.

Thin layer chromatography (TLC) was performed using glass-backed silica (Merck silica gel 60 F254) plates obtained from Merck. Ultraviolet lamp (λ_{max} = 254 nm) and/or $KMnO_4$ stain was used for visualization. R_F values are only reported for compounds that were purified by flash column chromatography. Flash column chromatography was performed using silica gel 60 (35-70 µm particles) supplied by Merck. Accurate Mass spectra were acquired on a Bruker Impact II QqTOF spectrometer equipped with a VIPHESI source using either electrospray or atmospheric pressure chemical ionisation. Samples were introduced using an HTC PAL autosampler and Bruker Elute Pump. HPLC columns were heated to 40 degrees Celsius unless otherwise stated. Samples passed through a Bruker Diode array UV-detector before entering the mass spectrometer. Perkin-Elmer One FT-IR spectrometer was used to analyse the infrared spectra.

Mass directed auto-purification (MDAP) of reactions was performed using an Agilent 1290 Infinity II Preparative HPLC system with mass spectrometer (LC/MSD XT), diode array detector and fraction collector. Initially, samples were analysed by analytical HPLC/MS (for data, see Supplementary Figure 12) [column: Agilent Technologies Poroshell 120 EC-C18 2.7 µm (3.0 x 50 mm) column with guard column; column temperature: ambient; solvent A: water with 0.1% formic acid; solvent B: acetonitrile with 0.1% formic acid; gradient programme: 2% B, 0 min → 2% B, 0.22 min → 98% B, 2.36 min →

98% B, 2.86 min → 2% B, 2.96 min → 2% B, 5.79 min; flow rate: 0.9 mL/min]. Productive reactions were then purified by mass-directed HPLC. The system ran in positive mode with an Agilent Technologies PLRP-S, 300Å, 8 µM particle size, 150x25 mm column at ambient temperature with a binary solvent system: MeCN and H_2O with 0.1% formic acid. The flow rate was set to 21.09 mL/min; the solvent gradient was automatically determined by the instrument.

Proton (^1H), carbon (^{13}C) and (^{19}F) NMR data was collected on a Bruker 400 or 500 MHz spectrometer. Data was collected at 298 K unless otherwise stated. Chemical shifts (δ) are given in parts per million (ppm) and they are referenced to the residual solvent peak. Coupling constants (*J*) are reported in Hertz (Hz) and splitting patterns are reported in an abbreviated manner: app. (apparent), s (singlet), d (doublet), t (triplet), q (quartet), m (multiplet), br. (broad). Assignments were made using COSY, HSQC, HMBC and NOESY experiments. The ^{13}C NMR spectra of perfluorophenyl-substituted compounds were complicated by fluorine-carbon coupling. The signals for carbon atoms on these substituents were complicated by coupling to ^{19}F (typically: $^1J_{CF}$ 255–265 Hz; $^2J_{CF}$ 13–15 Hz; $^3J_{CF}$ 1–6 Hz). In some cases, not all splitting patterns could be assigned, and are marked (†). Signals that were not observed are noted; in these cases, the presence of fluorine was confirmed by ^{19}F NMR spectroscopy. Diastereotopic protons are noted with -H superscript a and b (i.e -H^a). Signals for rotamers are denoted using subscripts (A and B).

4.3 Investigation of Functional Group Tolerance towards Pentafluorosulfonamide Nitrene Insertion

Initial scoping reactions were trialled with selected substrates bearing diverse functional groups. The methods for each specific compound in Section 4.4. Substrates were manually chosen from an available inventory, these included benzylic substrates (**S48**, **S50**, **S52**), allylic (**S49**, **S51**), aliphatic compounds (**S53**) with limited functionality (Supplementary Scheme 1) and an aromatic amine (**S56**) and alcohol (**S57**). Alkynes were also included as substrates (**S54**, **S55**), the incorporation, if tolerated, of these functionalities into products could serve as useful biorthogonal handles for the attachment of fluorescent or biotin tags.

Reactions with secondary benzylic substrates yielded the expected C–H insertion products **F48**, **F50** and **F52.** Styrenes (**S49**, **S51**) yielded aziridination products **F49** and **F51**. Interestingly, the aliphatic cyclohexane ring also proved to be a good substrate for this reaction yielding the corresponding C–H insertion product **F53**. It was established that substrates containing alcohols (**S56**), amines (**S57**) or

alkynes (**S54**, **S55**) were not compatible with this chemistry. Aromatic amines and alcohols with benzylic groups also did not yield the expected C–H insertion products and the reactions often returned the starting sulfonamide. The alkyne functionalities were also found to be incompatible with this nitrene insertion reaction.

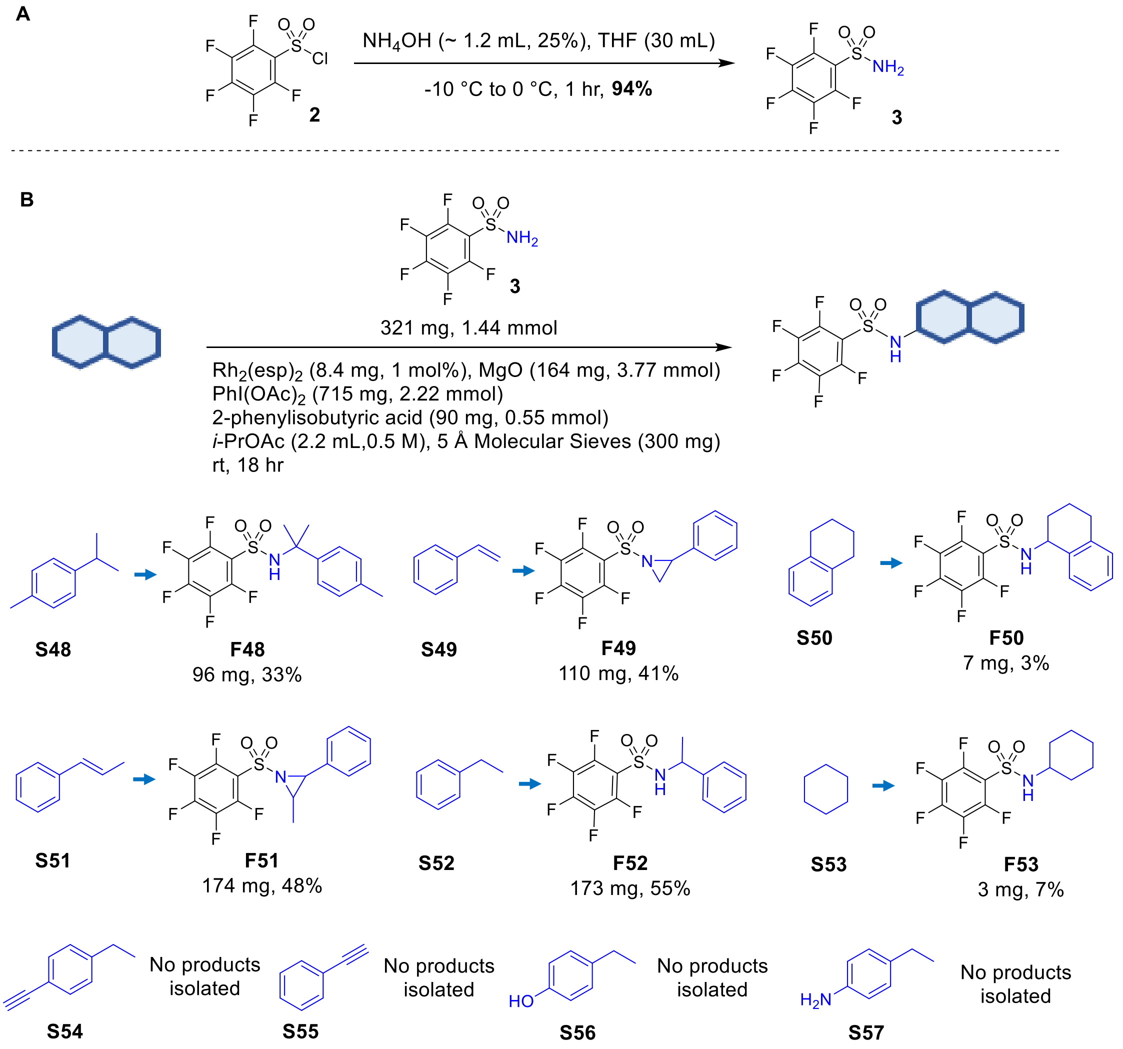

Supplementary Scheme 1: A) Synthesis of the sulfonamide building block **3** from the corresponding commercially available sulfonyl chloride. B) General reaction scheme of the rhodium-catalysed nitrenoid insertion reaction between a generic substrate and the sulfonamide **3**, substrates that were used for the substrate scoping experiments are reported below in blue together with the structure of the products or the outcome of the reaction.

4.4 Compound Synthesis

General Procedure A – Rhodium Catalysed Nitrenoid Chemistry

A round bottom flask was charged with 2,3,4,5,6-pentafluorobenzenesulfonamide (100 mg, 0.4 mmol, 1.3 equiv), 2-phenylisobutyric acid (25.4 mg, 0.2 mmol, 0.5 equiv), MgO (46 mg, 1.1 mmol, 3.7 equiv) and 5 Å molecular sieves (100 mg) and the corresponding solid substrates (0.3 mmol, 1.0 equiv). The magnesium oxide and molecular sieves were pre-dried at 120 °C for at least 18 hr and heated with a heat gun under vacuum before use. The flask was then sealed, evacuated and back-filled with nitrogen three time. For liquid substrates (0.3 mmol, 1.0 equiv), these were dissolved in *i*-PrOAc (150 µL) in a separate microwave vial and then transferred to the solid reagents, the transfer was made complete with further *i*-PrOAc (150 µL). A solution of $Rh_2(esp)_2$ (120 µL, 9.5 mM in *i*-PrOAc) was transferred to the solid reagents. Further *i*-PrOAc was added to bring the reaction to the desired volume (620 µL) and concentration (0.5 M). The mixture was then allowed to stir for five min, unstoppered, and $PhI(OAc)_2$ (200 mg, 0.6 mmol, 2.0 equiv) was added. The flask was re-sealed, flushed with nitrogen and allowed to stir at room temperature overnight under N_2. The crude mixture was then diluted with methanol (10 mL), filtered over Celite and the cake was washed with further methanol (20 mL). Removal of solvent under reduced pressure afforded a crude paste or oil which was dissolved in acetonitrile (500 µL). Crude product mixtures were initially analysed by analytical LC-MS; if the expected product mass was found, the crude mixture was purified by mass-directed HPLC.

2,3,4,5,6-Pentafluorobenzenesulfonamide (1)

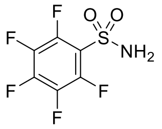

Pentafluorobenzenesulfonyl chloride (892 µL, 6.0 mmol) was added to THF (30 mL) in a round bottom flask and the solution was cooled to -15 °C in an ice bath with NaCl. NH_4OH (28% NH_3 in H_2O, c.a. 1 mL) was slowly added drop-wise over a period of 20 min until the reaction mixture maintained a stable pH of 7. The solution was then allowed to stir at room temperature for one hr after which the solvent was removed under reduced pressure to yield a pale-yellow solid which was transferred into a separation funnel with H_2O (100 mL) and EtOAc (50 mL). The two layers were separated and the aqueous was extracted with further EtOAc (2 x 50 mL). The combined organic layers were dried over Na_2SO_4, filtered and the solvent was removed to give a yellow precipitate. Purification was achieved by washing the precipitate with $CHCl_3$ (10 mL) and filtering the suspension to yield the *sulfonamide* as a colourless solid (1.39 g, 94%). δ_H (500 MHz, $CDCl_3$) 8.50 (2H, br. s, NH_2). δ_C (125 MHz, $CDCl_3$) 143.3 (ddq, *J* 263.0, 13.0 and 4.4 Hz, C-2 and -6), 142.8 (dtt, *J* 263.7, 13.4 and 5.4 Hz, C-4), 137.5 ($d^†$, *J* 259.0 Hz, C-3 and -5), 119.2-118.9 ($m^†$, C-1). δ_F (376 MHz, $CDCl_3$) –139.02- –139.08 (2F, m, 3- and 5-F or 2- and 6-F), –148.80- –148.92 (1F, m, 4-F), –160.43- –160.54 (2F, m, 3- and 5-F or 2- and 6-F). Data is consistent with literature reported values[4].

F1

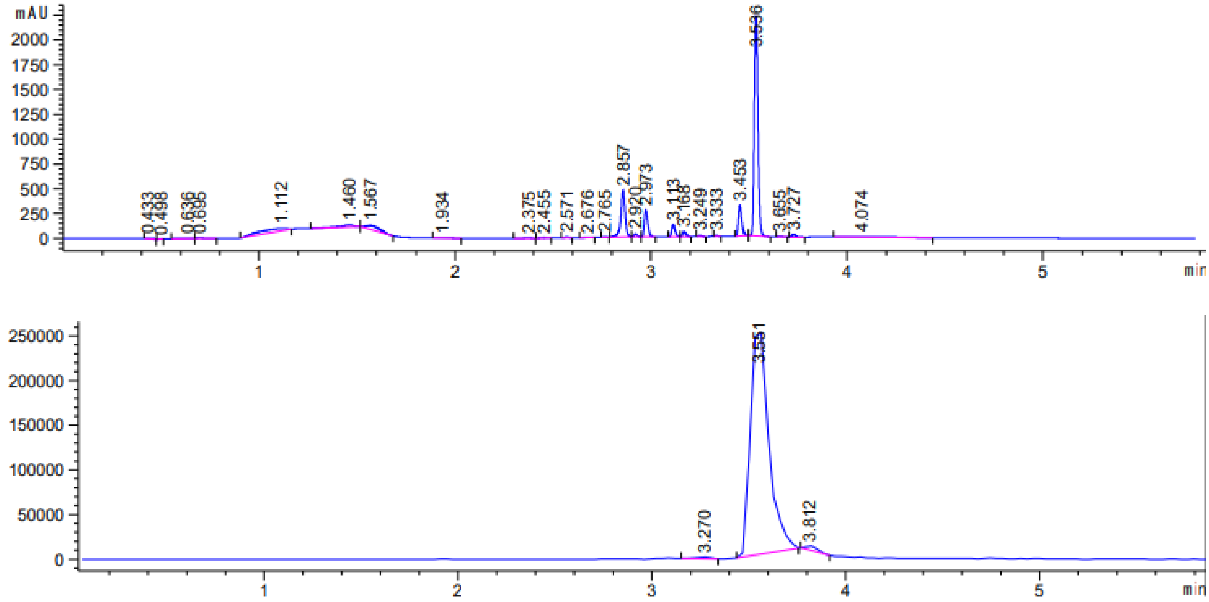
mAU
min
0.433
0.498
0.636
0.695
1.112
1.460
1.567
1.934
2.375
2.455
2.571
2.676
2.765
2.857
2.920
2.973
3.249
3.333
3.453
3.536
3.655
3.727
4.074
3.270
3.551
3.812

F2

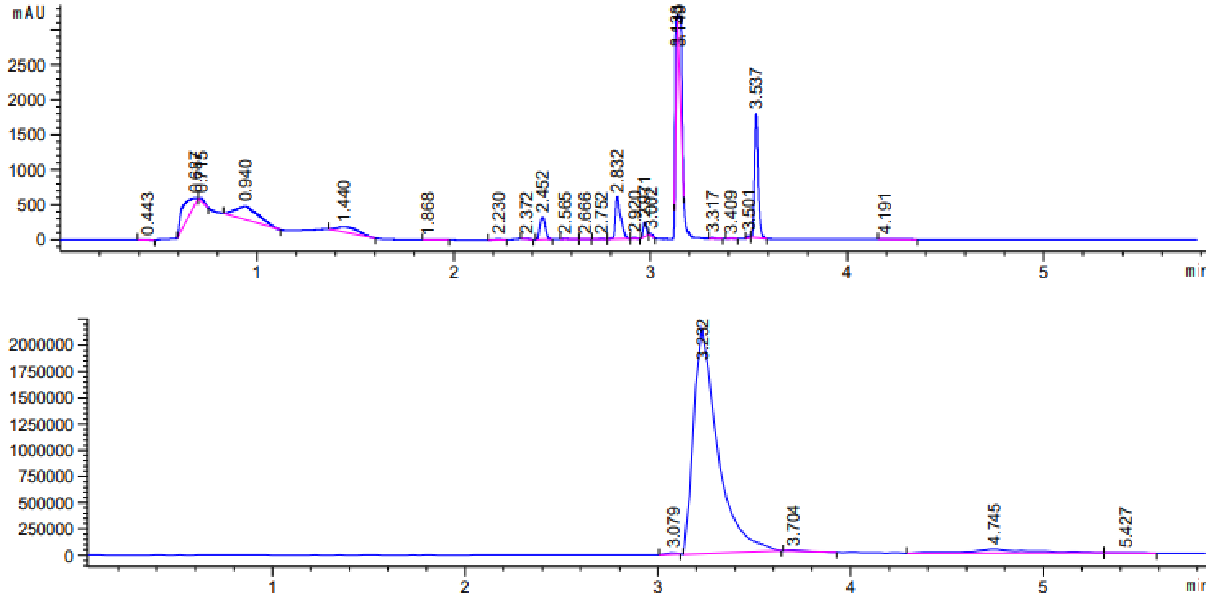
mAU
min
0.443
0.940
1.440
1.868
2.230
2.372
2.452
2.565
2.666
2.752
2.832
2.920
3.317
3.409
3.501
3.537
4.191
3.079
3.704
4.745
5.427

F5

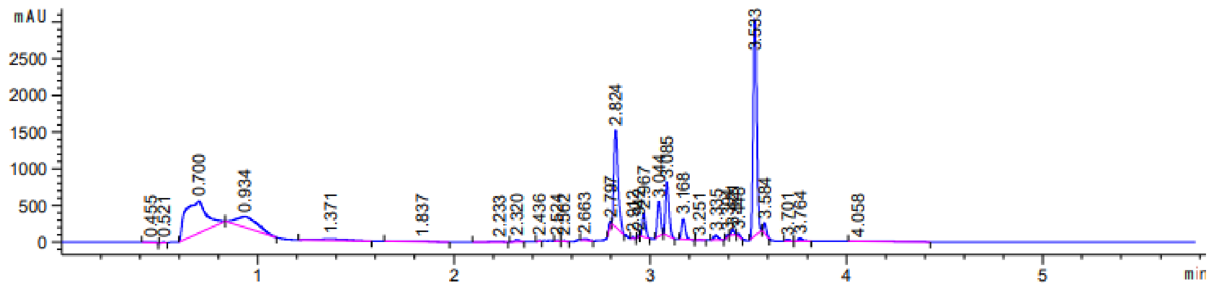

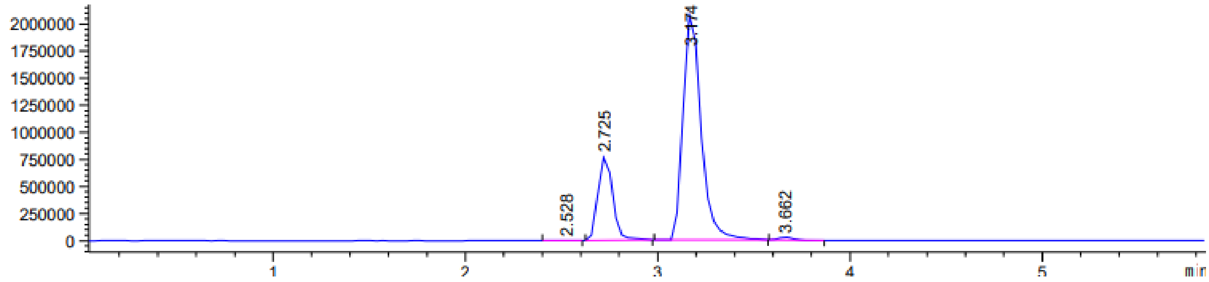

F6

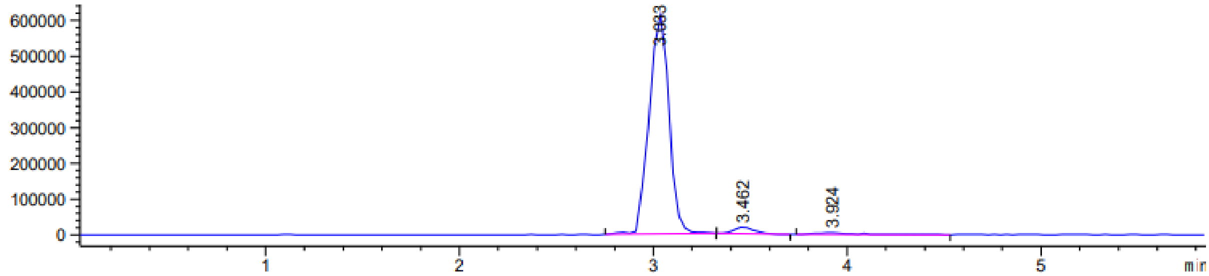

F7a and F7b

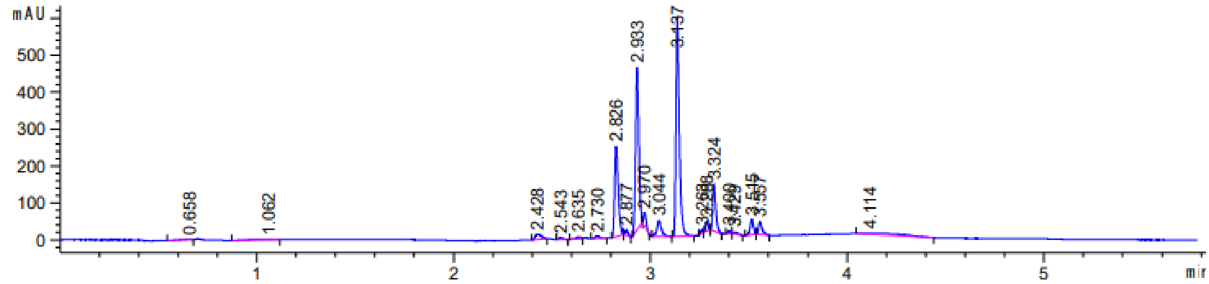

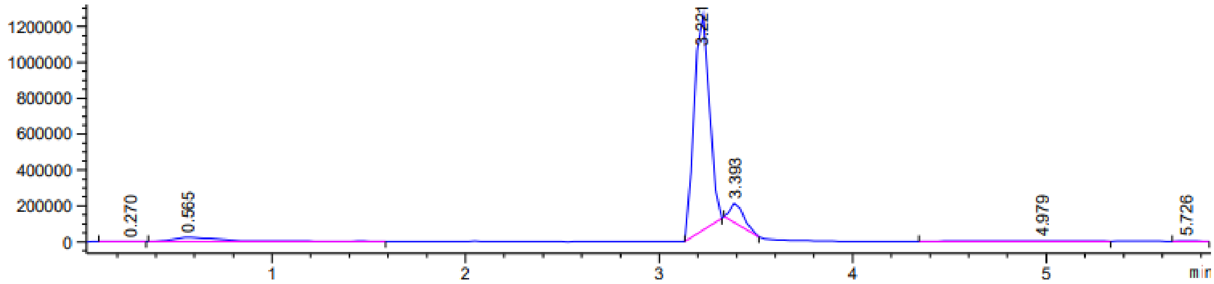

F10

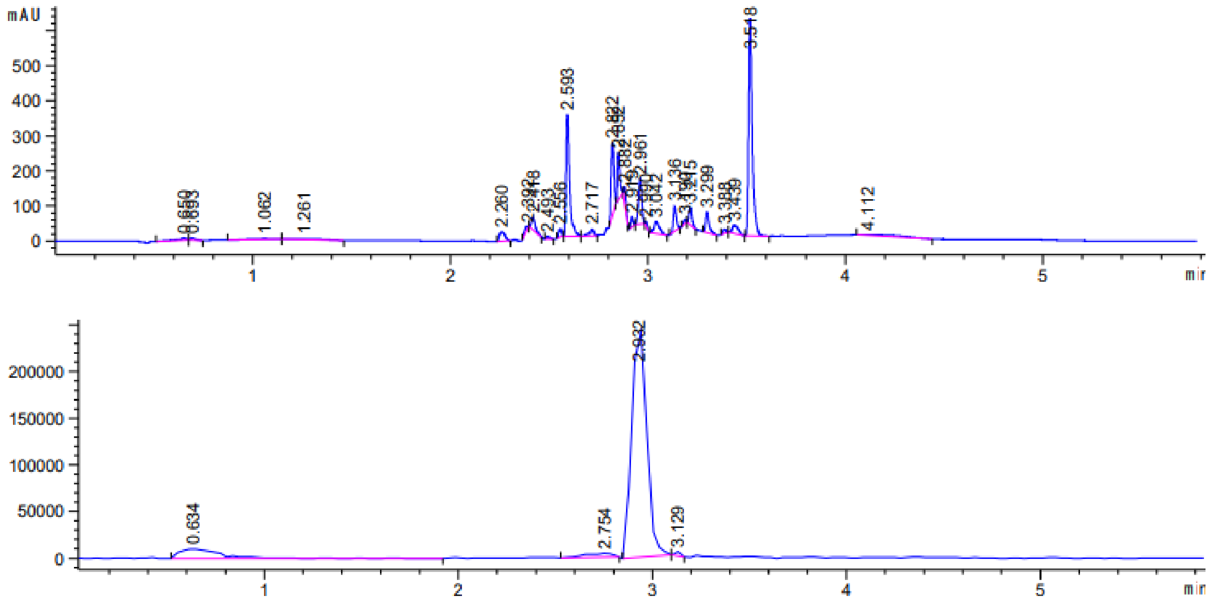

mAU
500
400
300
200
100
0
1
2
3
4
5
min
2.593
3.518
1.062
1.261
2.260
2.717
4.112
3.299
200000
150000
100000
50000
0
0.634
2.754
2.932
3.129

F21

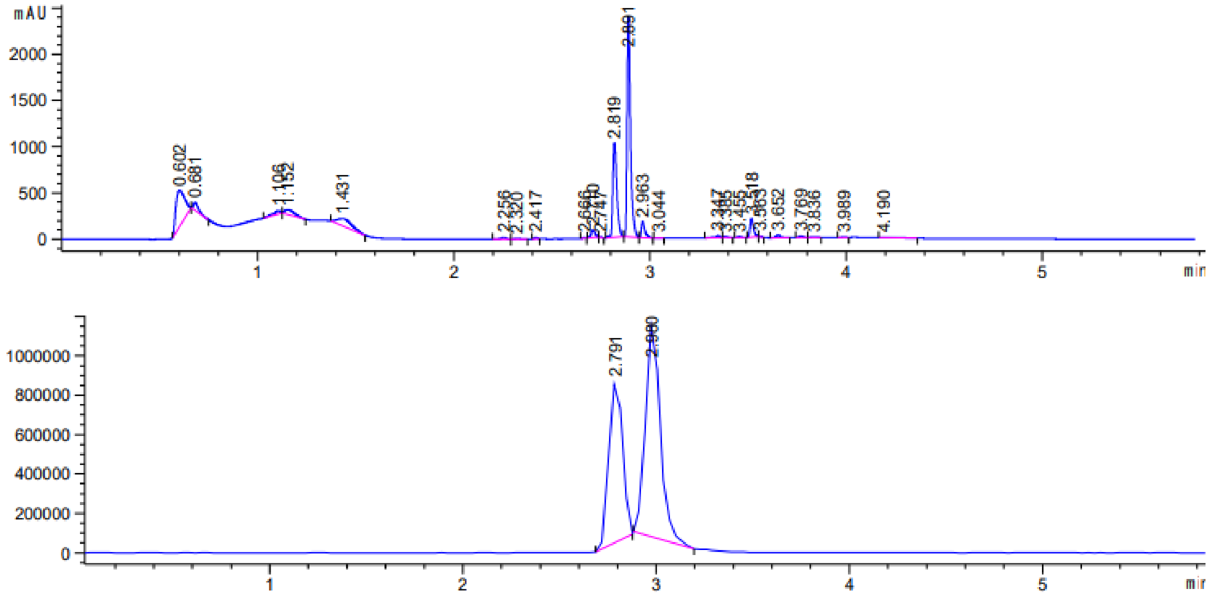

mAU
2000
1500
1000
500
0
1
2
3
4
5
min
0.602
0.681
1.431
2.819
2.891
2.963
3.044
3.652
3.989
4.190
1000000
800000
600000
400000
200000
0
2.791
2.990

F23

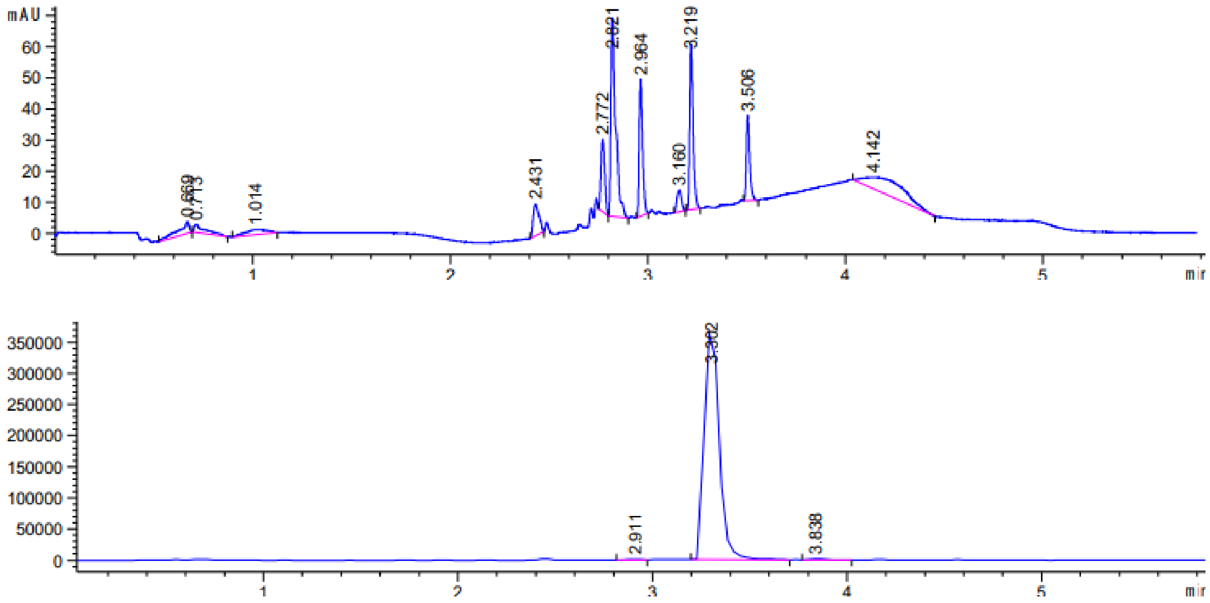
mAU
60
50
40
30
20
10
0
1.014
2.431
2.772
2.964
3.160
3.219
3.506
4.142
350000
300000
250000
200000
150000
100000
50000
2.911
3.838
1
2
3
4
5

F24

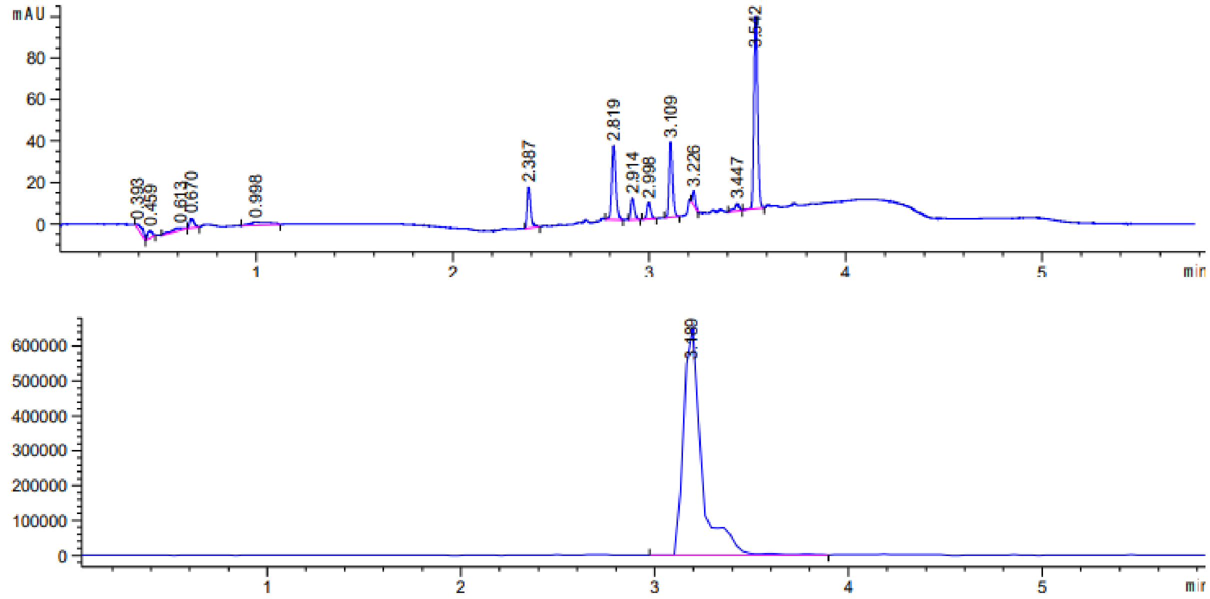
mAU
80
60
40
20
0
0.393
0.459
0.670
0.998
2.387
2.819
2.914
2.998
3.109
3.226
3.447
600000
500000
400000
300000
200000
100000
1
2
3
4
5

F25

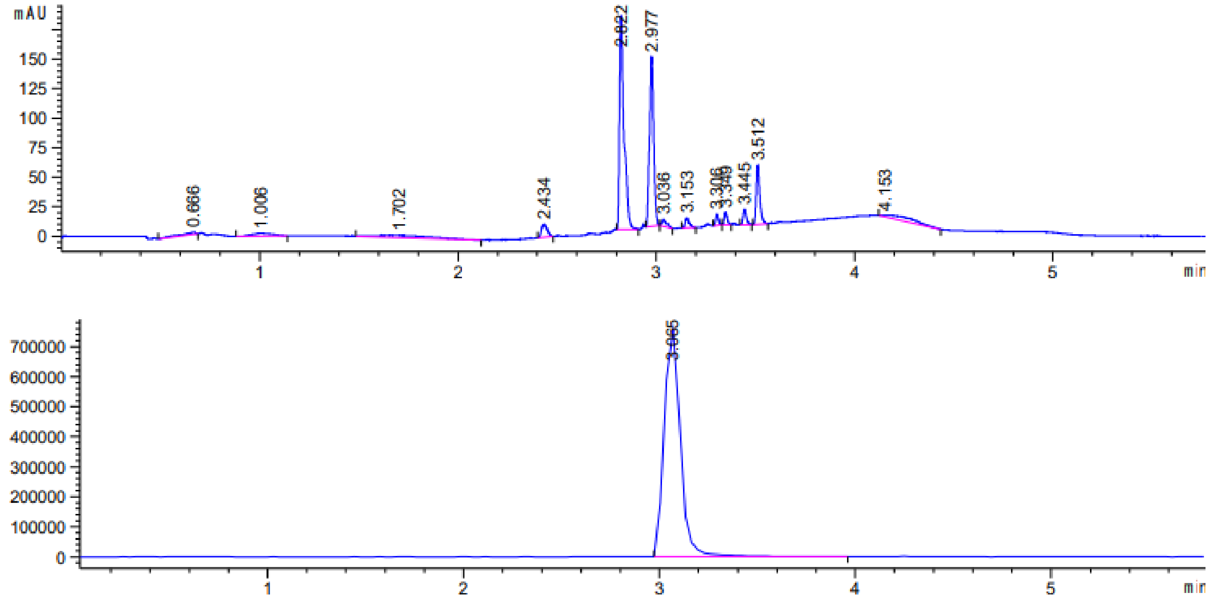
mAU
150
125
100
75
50
25
0
0.666
1.006
1.702
2.434
2.822
2.977
3.036
3.153
3.306
3.349
3.445
3.512
4.153
1
2
3
4
5
min
700000
600000
500000
400000
300000
200000
100000
0
3.065

F35

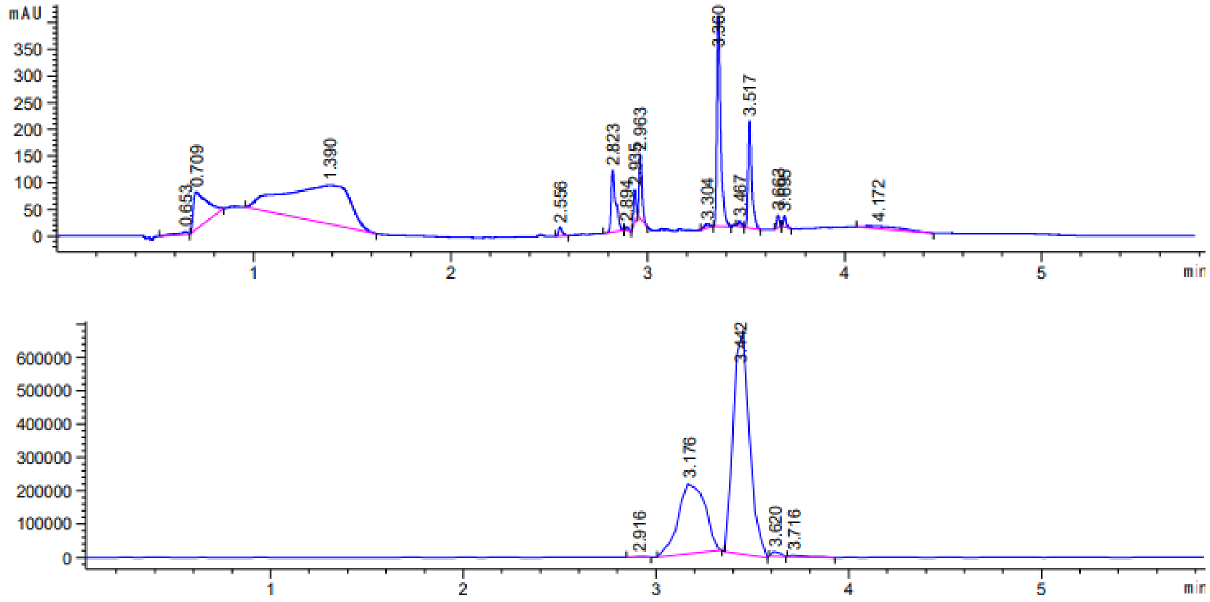
mAU
350
300
250
200
150
100
50
0
0.653
0.709
1.390
2.556
2.823
2.894
2.935
2.963
3.304
3.360
3.467
3.517
3.663
3.695
4.172
1
2
3
4
5
min
600000
500000
400000
300000
200000
100000
0
2.916
3.176
3.442
3.620
3.716

F37

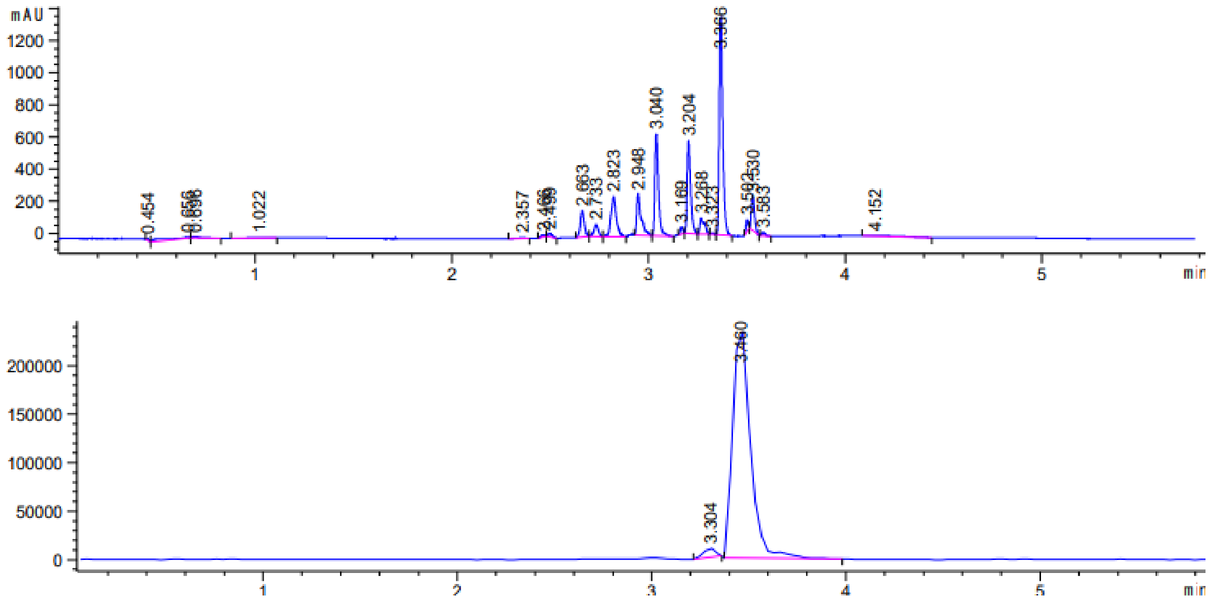
mAU
1200
1000
800
600
400
200
0
0.454
1.022
2.357
2.663
2.733
2.823
2.948
3.040
3.169
3.204
3.268
3.583
4.152
1
2
3
4
5
min
200000
150000
100000
50000
0
3.304

F39

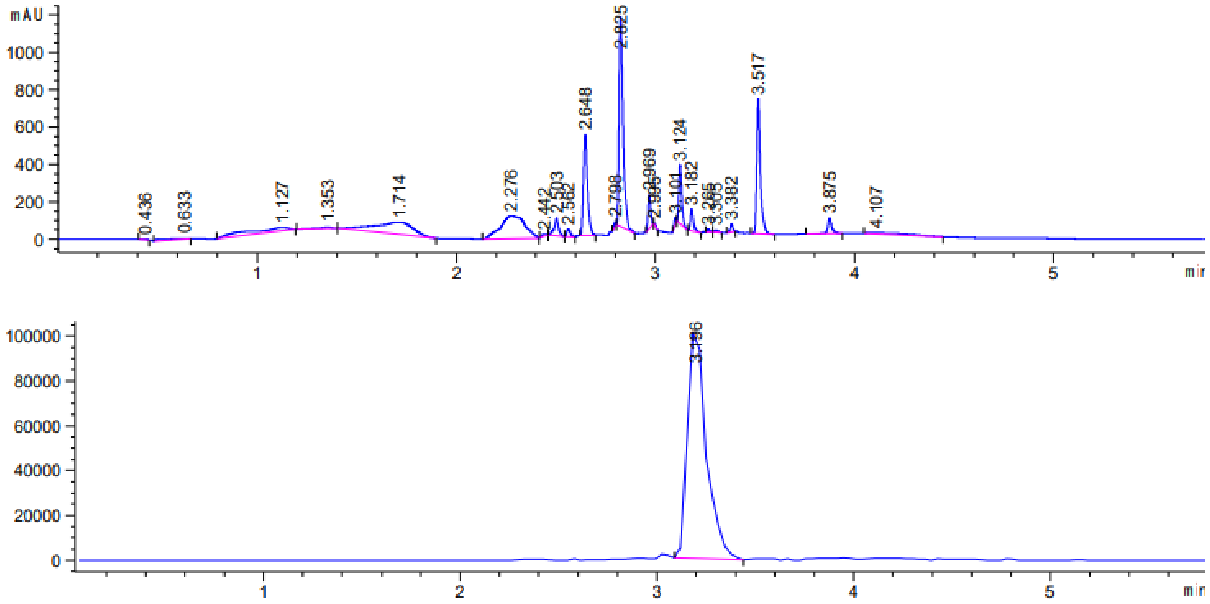
mAU
1000
800
600
400
200
0
0.436
0.633
1.127
1.353
1.714
2.276
2.442
2.503
2.562
2.648
2.798
2.825
2.969
3.101
3.124
3.182
3.382
3.517
3.875
4.107
1
2
3
4
5
min
100000
80000
60000
40000
20000
0

F44a and F44b

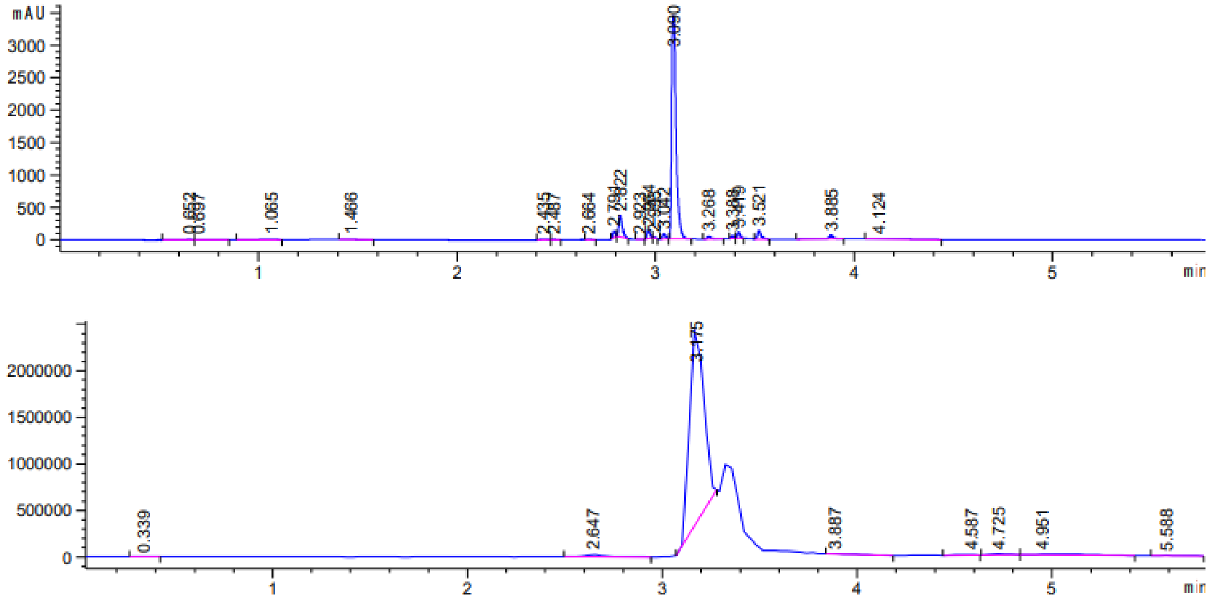

Supplementary Figure 12: Analytical HPLC/MS data for reactive fragments purified by mass-directed HPLC. For each reactive fragment, chromatograms are shown for absorbance at 254 nm (top) and detection of a parent ion (MH^+ or MNa^+; bottom).

2,3,4,5,6-Pentafluoro-*N*-(1-((4-fluorophenyl)sulfonyl)pyrrolidin-2-yl)benzenesulfonamide (F1)

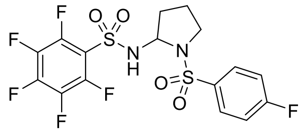

Prepared according to General Procedure A with substrate **S1**. The crude reaction mixture was purified by reverse phase MDAP-HPLC eluting with H_2O/CH_3CN (with 0.1% formic acid) over 12 min gave the *sulfonamide* as an amber oil (27 mg, 18%). ν_{max}/cm^{-1} 1645, 1592, 1520, 1494, 1349, 1295, 1239, 1166, 1153, 1095, 988, 839, 820, 727, 711, 673, 645, 607, 580, 546, 511 and 488. δ_H (500 MHz, CD_3OD) 7.83-7.80 (2H, m, fluorophenyl 2- and 6-H), 7.34-7.31 (2H, m, fluorophenyl 3- and 5-H), 5.29-5.27 (1H, dd, *J* 1.96 and 7.12 Hz, pyrrolidinyl 2-H), 3.43-3.38 (1H, m, pyrrolidinyl 5-H^a), 3.07-3.02 (1H, m, pyrrolidinyl 5-H_b), 2.09-2.00 (1H, m, pyrrolidinyl 3-H^a), 1.96-1.91 (1H, m, pyrrolidinyl 4-H^a), 1.83-1.73 (1H, m, pyrrolidinyl 4-H^b) 1.65-1.58 (1H, m, pyrrolidinyl 3-H^b). δ_C (125 MHz, CD_3OD) 166.9 (d, *J* 254 Hz, fluorophenyl C-4) 146.2 (ddq, *J* 263.0, 13.0 and 4.4 Hz, C-2 and -6), 145.0 (dtt, *J* 263.7, 13.4 and 5.4 Hz, C-4), 139.2 (d, *J* 259 Hz, fluorophenyl C-3 and -5), 134.9 (d, *J* 3.22 Hz, fluorophenyl C-1), 131.4 (d, *J* 9.5 Hz, fluorophenyl C-2 and -6), 119.4 (C-1), 117.6 (d, *J* 23 Hz, fluorophenyl C-3 and -5), 71.8 (pyrrolidinyl C-2), 49.2° (pyrrolidinyl C-5), 35.5 (pyrrolidinyl C-4), 23.9 (pyrrolidinyl C-3). °Peak covered by CD_3OD. δ_F (376 MHz, CD_3OD) –106.9 (s, phenyl F), –139.1 (2F, m, 3- and 5-F or 2- and 6-F), –152.0 (1F, m, 4-F), –164.0- –164.1 (2F, m, 3- and 5-F or 2- and 6-F). HRMS: $C_{16}H_{12}F_6N_2O_4S_2$ [M+H] requires: 475.0215; found: 475.0214.

((Perfluorophenyl)sulfonyl)(5,6,7,8-tetrahydroquinoxalin-1-ium-1-yl)amide (F2)

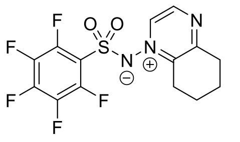

Prepared according to General Procedure D with substrate **S2**. The crude reaction mixture was purified by reverse phase MDAP-HPLC eluting with H_2O/CH_3CN (with 0.1% formic acid) over 12 min gave the *amide* as a colourless solid (17 mg, 14%). ν_{max}/cm^{-1} 1566, 1470, 1391, 1276, 1192, 1171, 1135, 1085, 1052, 1005, 954, 929, 854, 815, 737, 696, 641, 603, 568, 482 and 470. δ_H (500 MHz, CD_3OD) 8.75 (1H, app. br. d, *J* 3.28 Hz, quinoxalinyl 3-H), 8.65 (1H, app. br. s, quinoxalinyl 2-H), 3.10-3.07 (4H, m, quinoxalinyl 5- and 8-H_2), 1.93 (4H, br. s, quinoxalinyl 6- and 7-H_2). δ_C (125 MHz, CD_3OD) 160.6 (quinoxalinyl C-4a or C-8a), 144.5 (d†, *J* 263.0 Hz, aryl C-2 and -6), 144.3 (quinoxalinyl C-2), 143.91 (d†, *J* 263.7 Hz, perfluorophenyl C-4), 143.94 (quinoxalinyl C-4_a or C-8_a), 137.9 (d†, *J* 258.1 Hz, perfluorophenyl C-3 and -5), 118.8 (m†, perfluorophenyl C-1), 32.73 (quinoxalinyl C-5 or C-8), 26.6 (quinoxalinyl C-5 or C-8), 21.29 (quinoxalinyl C-6 or C-7), 21.27 (quinoxalinyl C-6 or C-7). δ_F (376 MHz, CD_3OD) –136.44- –136.50 (2F, m, perfluorophenyl 2- and 6-F or 3- and 5-F), –147.87- –147.98 (1F, m, perfluorophenyl F-4), –159.01- –159.11 (2F, m, perfluorophenyl 2- and 6-F or 3- and 5-F). HRMS: $C_{14}H_{10}F_5N_3O_2S$ [M+H] requires: 380.0487; found: 380.0486.

1-(4-Methoxyphenyl)-2-((perfluorophenyl)sulfonamido)ethyl acetate (F4)

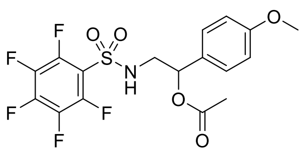

Prepared according to General Procedure A with substrate **S4**. The crude reaction mixture was purified by reverse phase MDAP-HPLC eluting with H_2O/CH_3CN (with 0.1% formic acid) over 12 min. Further purification by column chromatography was required, eluting with acetone/hexane 15:85, to give the *acetate* as a colourless solid (7.1 mg, 5%). R_F = 0.19 (acetone/hexane 20:80). ν_{max}/cm^{-1} 1737, 1645, 1613, 1518, 1495, 1361, 1299, 1242, 1169, 1099, 1030, 989, 832, 727, 605 and 578. δ_H (500 MHz, CD_3OD) 7.21-7.19 (2H, m, phenyl 2- and 6-H), 6.81-6.79 (2H, m, phenyl 3- and 5-H), 5.70-5.68 (1H, t, *J* 6.2 Hz, ethyl 1-H), 3.76 (3H, s, methoxy H_3), 3.56-3.55 (2H, m, ethyl 2-H_2), 2.05 (3H, s, acetate H_3). δ_C (125 MHz, CD_3OD) 171.7 (carbonyl), 161.2 (phenyl C-4), 145.4 (d†, *J* 263.7 Hz, perfluorophenyl C-2 and -6), 144.8 (d†, *J* 257.4 Hz, perfluorophenyl C-4), 139.1 (d†, *J* 257.4 Hz, perfluorophenyl C-3 and 5), 130.7 (phenyl C-1), 129.1 (phenyl C-2 and -6), 118.8 (m†, perfluorophenyl C-1), 114.7 (phenyl C-3 and -5), 75.0 (ethyl C-1), 55.6 (methoxy), 48.6 (ethyl C-2), 20.9 (acetate *C*H_3). δ_F (376 MHz, CD_3OD) –139.22- –139.27 (2F, m, perfluorophenyl 3- and 5-F or 2- and 6-F), –151.42- –151.53 (1F, m, perfluorophenyl

4-F), –162.87- –162.96 (2F, m, perfluorophenyl 3- and 5-F or 2- and 6-F). HRMS: $C_{17}H_{14}F_5NO_5S$ [M+Na] requires: 462.0405; found: 462.0410.

2-Cyclohexyl-2,2-dimethyl-1-((perfluorophenyl)sulfonyl)hydrazin-2-ium-1-ide (F5)

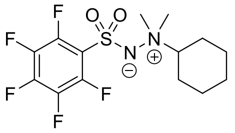

Prepared according to General Procedure A with substrate **S5**. The crude reaction mixture was purified by reverse phase MDAP-HPLC eluting with H_2O/CH_3CN (with 0.1% formic acid) over 12 min gave the *hydrazinide* as a colourless solid (2.6 mg, 2%). ν_{max}/cm^{-1} 1644, 1592, 1519, 1491, 1365, 1294, 1237, 1152, 1096, 1026, 987, 837, 729.35, 672, 598, 579 and 548. δ_H (500 MHz, CD_3OD) 3.51-3.45 (1H, tt, *J* 11.8 and 3.3 Hz, cyclohexyl 1-H), 3.25 (6H, s, dimethyl H_3), 2.32-2.29 (2H, m, cyclohexyl, 2 and 6-H^a), 1.95-1.92 (2H, m, cyclohexyl 3- and 5-H^a), 1.69-1.66 (1H, cyclohexyl, 4-H^a), 1.57-1.49 (2H, qd, *J* 12.1, 12.0 and 3.4 Hz, 2- and 6- H^b), 1.38-1.31 (2H, m, 3- and 5-H^a), 1.22-1.16 (1H, tt, *J* 13.0 and 3.6 Hz cyclohexyl 4-H^b). δ_C (125 MHz, CD_3OD) 81.1 (cyclohexyl C-1), 52.5 (dimethyl C_2), 27.7 (cyclohexyl C-2 and -6) 26.6 (cyclohexyl C-3 and -5), 26.1 (cyclohexyl C-4). Fluorinated carbons not visible due to low sample concentration. δ_F (376 MHz, CD_3OD) –139.76- –139.87 (2F, m, perfluorophenyl 3- and 5-F or 2- and 6-F), –154.30- –154.40 (1F, m, perfluorophenyl 4-F), –163.60- –163.70 (2F, m, perfluorophenyl 3- and 5-F or 2- and 6-F). $C_{14}H_{17}F_5N_2O_2S$ [M+H] requires: 373.1004; found: 373.1005.

2,3,4,5,6-Pentafluoro-*N*-((2-oxoazepan-1-yl)methyl)benzenesulfonamide (F6)

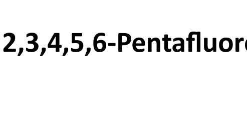

Prepared according to General Procedure A with substrate **S6**. The crude reaction mixture was purified by reverse phase MDAP-HPLC eluting with H_2O/CH_3CN (with 0.1% formic acid) over 12 min gave the *sulfonamide* as a colourless solid (22 mg, 19%). ν_{max}/cm^{-1} 1631, 1519, 1493, 1445, 1354, 1297, 1262, 1198, 1167, 1097, 1049, 988, 890, 868, 824, 728, 643, 602, 577, 530, 485 and 439. δ_H (500 MHz, $CDCl_3$) 4.75 (2H, s, methyl H_2), 3.49-3.47 (2H, m, oxoazepanyl 2-H_2), 2.42-2.40 (2H, m, oxoazepanyl 6-H_2), 1.76-1.68 (4H, m, oxoazepanyl 3- and 4-H_2), 1.59-1.54 (2H, m, oxoazepanyl 5-H_2). δ_C (125 MHz, $CDCl_3$) 179.0 (oxoazepanyl C-2), 145.8 (ddq, *J* 263.0, 13.0 and 4.4 Hz, C-2 and -6), 145.0 (dtt, *J* 263.7, 13.4 and 5.4 Hz, C-4) 139.2 ($d^†$, *J* 259 Hz, C-3 and -5), 119.1-118.8 ($m^†$, C-1), 56.9 (methyl), 50.1 (oxoazepanyl C-7), 37.5 (oxoazepanyl C-6), 30.7 (oxoazepanyl C-3 or C-4), 29.5 (oxoazepanyl C-3 or C-4), 24.3 (oxoazepanyl C-

5). δ_F (376 MHz, $CDCl_3$) –139.5 (2F, m, 3- and 5-F or 2- and 6-F), –151.0- –151.1 (1F, m, 4-F), -163.0- –163.1 (2F, m, 3- and 5-F or 2- and 6-F). HRMS: $C_{13}H_{13}F_5N_2O_3S$ [M+H] requires: 373.0640; found: 373.0644.

(2-Isobutylthiazol-3-ium-3-yl)((perfluorophenyl)sulfonyl)amide (F7a)

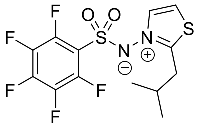

Prepared according to General Procedure A with substrate **S7a**. The crude reaction mixture was purified by reverse phase MDAP-HPLC eluting with H_2O/CH_3CN (with 0.1% formic acid) over 12 min gave the *sulfonyl amide* as a colourless solid (8 mg, 7%). ν_{max}/cm^{-1} 1643, 1518, 1492, 1317, 1295, 1152, 1096, 988, 901, 605, 579 and 530. δ_H (500 MHz, CD_3OD) 7.96 (1H, d, *J* 4.0 Hz, thiazolyl 4-H), 7.90 (1H, d, *J* 4.0 Hz, thiazolyl 5-H), 3.08 (2H, d, *J* 7.3 Hz, isobutyl 3-H_2), 2.24-2.16 (1H, m, isobutyl 2-H), 1.00 (6H, d, *J* 6.6 Hz, isobutyl 1-H_3 and methyl H_3). δ_C (125 MHz, CD_3OD) 172.4 (thiazolyl C-2), 146.0 ($d^†$, *J* 263.0 Hz, perfluorophenyl C-2 and -6), 144.4 ($d^†$, *J* 263.7 Hz, perfluorophenyl C-4), 139.3 ($d^†$, *J* 258.1 Hz, perfluorophenyl C-3 and -5), 139.3 (thiazolyl C-4), 120.8 (thiazolyl C-5), 120.0 ($m^†$, perfluorophenyl C-1), 38.4 (isobutyl C-3), 29.8 (isobutyl C-2), 22.5 (isobutyl C_2). δ_F (376 MHz, CD_3OD) –138.91- –138.97 (2F, m, pentafluorophenyl 3- and 5-F or 2- and 6-F), –152.76- –152.87 (1F, m, pentafluorophenyl 4-F), –163.07- –152.87 (2F, m, pentafluorophenyl 3- and 5-F or 2- and 6-F). HRMS: $C_{13}H_{11}F_5N_2O_2S_2$ [M+H] requires: 387.0255; found: 387.0254.

2,3,4,5,6-Pentafluoro-*N*-(2-methyl-1-(thiazol-2-yl)propyl)benzenesulfonamide (F7b)

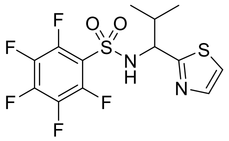

Prepared according to General Procedure A with substrate **S7b**. The crude reaction mixture was purified by reverse phase MDAP-HPLC eluting with H_2O/CH_3CN (with 0.1% formic acid) over 12 min. Further purification by column chromatography was required, eluting with acetone/hexane 30:70, to give the *sulfonamide* as a colourless solid (2 mg, 2%). R_F = 0.17 (acetone/hexane 30:70). ν_{max}/cm^{-1} 1736, 1643, 1518.67, 1491, 1373, 1322, 1294, 1235, 1153, 1095, 1017, 988, 898, 819, 756, 727, 657, 602, 578, 537 and 489. δ_H (500 MHz, CD_3OD) 8.17-8.16 (1H, d, *J* 4.0 Hz, thiazole 4-H), 8.18-8.11 (1H, d, *J* 4.0 Hz, thiazole 5-H), 5.52-5.51 (1H, d, *J* 6.2 Hz, 3-H), 2.63-2.67 (1H, m, propyl 2-H), 1.08 (3H, d, *J* 2.1 Hz, propyl 3-H_3 or methyl H_3), 1.07 (3H, d, *J* 2.1 Hz, propyl 3-H_3 or methyl H_3). δ_C (125 MHz, CD_3OD) 170.4 (thiazole C-4), 139.7 (thiazole C-5), 123.2 (thiazole C-2), 61.1 (propyl C-1), 35.8 (propyl C-2), 20.1 (propyl C-3 or methyl

C), 17.9 (propyl C-3 or methyl C). Fluorinated carbons not visible due to low sample concentration. δ_F (376 MHz, CD_3OD) –138.91- –138.96 (2F, m, 3- and 5-F or 2- and 6-F), –152.53- –152.64 (1F, m, 4-F), –162.89- –162.99 (2F, m, 3- and 5-F or 2- and 6-F). HRMS: $C_{13}H_{11}F_5N_2O_2S_2$ [M+H] requires: 387.0255; found: 387.0241.

N-(6,7-Dihydro-5H-pyrrolo[2,1-c][1,2,4]triazol-3-yl)-2,3,4,5,6-pentafluorobenzenesulfonamide (F10)

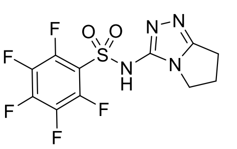

Prepared according to General Procedure A with substrate **S10**. The crude reaction mixture was purified by reverse phase MDAP-HPLC eluting with H_2O/CH_3CN (with 0.1% formic acid) over 12 min. Further purification by flash column chromatography was required, eluting with acetone/hexane 40:60 → 50:50, to give the *sulfonamide* a colourless solid (1.8 mg, 2%). R_F = 0.24 (acetone/hexane 40:60). ν_{max}/cm^{-1} 1642, 1583, 1518, 1491, 1395, 1343, 1294, 1152, 1095, 1052, 987, 950, 761, 727, 689, 644, 611, 579, 558 and 498. δ_H (500 MHz, CD_3OD) 3.88-3.86 (2H, t, *J* 7.2 Hz, pyrrolo 5-H_2), 2.93-2.90 (2H, t, *J* 7.3 Hz, pyrrolo 7-H_2), 2.69-2.63 (2H, p, *J* 14.5 and 7.8 Hz, pyrrolo 6-H_2). δ_C (125 MHz, CD_3OD) 158.6 (triazole C-7a), 149.2 (triazole C-3), 145.6 ($d^†$, *J* 258.1 Hz, C-2 and -6), 144.2 ($d^†$, *J* 263.7 Hz, C-4), 139.2 ($d^†$, *J* 258.1 Hz, C-3 and -5), 120.9 ($m^†$, C-1), 44.1 (pyrrolo C-5), 27.9 (pyrrolo C-6), 22.3 (pyrrolo C-7). δ_F (376 MHz, CD_3OD) –139.66- –139.71 (2F, m, 3- and 5-F or 2- and 6-F), –152.9- –153.07 (1F, m, 4-F), –163.46- –163.56 (2F, m, 3- and 5-F or 2- and 6-F). HRMS: $C_{11}H_7F_5N_4O_2S$ [M+H] requires: 355.0283; found: 355.0286.

((Perfluorophenyl)sulfonyl)(4-(tetrahydro-2H-pyran-4-yl)pyridin-1-ium-1-yl)amide (F21)

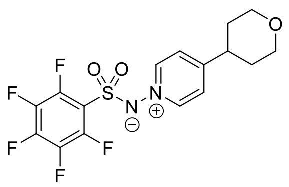

Prepared according to General Procedure A with substrate **S21**. The crude reaction mixture was purified by reverse phase MDAP-HPLC eluting with H_2O/CH_3CN (with 0.1% formic acid) over 12 min. Further purification by flash column chromatography was required, eluting with acetone/hexane 40:60, to give the *amide* as a colourless solid (4.7 mg, 4%). R_F = 0.15 (acetone/hexane 40:60). ν_{max}/cm^{-1} 1721, 1642, 1519, 1491, 1450, 1370, 1295, 1151, 1096, 987, 898, 862, 727, 639, 603, 579, 532, 489 and 456. δ_H (500 MHz, CD_3OD) 8.52-8.51 (2H, m, pyridinyl 2- and 6-H), 7.82-7.80 (2H, m, pyridinyl 3- and 5-H), 4.08-4.05 (2H, m, pyranyl 2-H_2 or 6-H_2), 3.60-3.55 (2H, td, *J* 11.6 and 2.6 Hz, pyranyl 2-H_2 or 6-H_2), 3.17-3.11 (1H, m, pyranyl 4-H), 1.82-1.77 (4H, m, pyranyl 3- and 5-H_2). δ_C (125 MHz, CD_3OD)

164.6 (pyridinyl C-4), 146.7 (pyridinyl C-2 and -6), 127.7 (pyridinyl C-3 and -5), 68.6 (pyranyl C-2 and -6), 42.1 (pyranyl C-4), 33.4 (pyranyl C-3 and -5). Fluorinated carbons not visible due to low sample concentration. δ_F (376 MHz, CD_3OD) –138.91- –138.97 (2F, m, perfluorophenyl 3- and 5-F or 2- and 6-F), –153.02- –151.07 (1F, m, perfluorophenyl 4-F), –163.18- –163.28 (2F, m, perfluorophenyl 3- and 5-F or 2- and 6-F). HRMS: $C_{16}H_{13}F_5N_2O_3S$ [M+H] requires: 409.0640; found: 409.0640.

_N_-(1-Benzyl-5-oxopyrrolidin-2-yl)-2,3,4,5,6-pentafluorobenzenesulfonamide (F23)

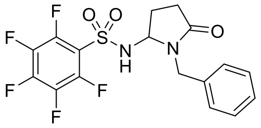

Prepared according to General Procedure A with substrate **S23**. The crude reaction mixture was purified by reverse phase MDAP-HPLC eluting with H_2O/CH_3CN (with 0.1% formic acid) over 12 min gave the *sulfonamide* as a colourless rotameric and diastereotopic solid (6 mg, 5%). ν_{max}/cm^{-1} 1681, 1519, 1497, 1444, 1363, 1295, 1249, 1161, 1098, 1064.84, 990, 969, 735, 695, 666, 642, 606, 578 and 535. δ_H (500 MHz, Acetone-d_6) 7.31-7.23 (5H, m, benzyl H_5), 5.20-5.17 (1H, m, pyrrolidinyl 2-H), 4.81-4.78 (1H, d, *J* 15.3 Hz, benzylic H), 4.09-4.06 (1H, d, *J* 15.3 Hz, benzylic H), 2.59-2.52 (1H, m, pyrrolidinyl 4-H^a), 2.49-2.42 (1H, m, pyrrolidinyl 3-H^a), 2.33-2.27 (1H, ddd, *J* 13.6, 9.8 and 7.8 Hz, pyrrolidinyl 4-H^b), 2.00 (1H, ddd, *J* 16.4, 6.7 and 3.6 Hz pyrrolidinyl 3-H^b). δ_C (125 MHz, CD_3OD) 174.3 (pyrrolidinyl C-5), 145.8 ($d^†$, *J* 263.0 Hz, C-2 and -6), 144.8 ($d^†$, *J* 263.7 Hz, C-4), 139.0 ($d^†$, *J* 258.1 Hz, C-3 and -5), 138.0 (benzyl C-1), 129.3 (benzyl C-3 and -5), 128.5 (benzyl C-2 and -6), 128.1 (benzyl C-4), 118.4 ($m^†$, C-1), 69.6 (pyrrolidinyl C-$2_{A\ or\ B}$), 69.5 (pyrrolidinyl C-$2_{A\ or\ B}$), 43.7 (benzylic *C*) 28.8 (pyrrolidinyl C-4), 27.2 (pyrrolidinyl C-$3_{A\ or\ B}$), 27.1 (pyrrolidinyl C-$3_{A\ or\ B}$). δ_F (376 MHz, CD_3OD) –138.94- –138.99 (2F, m, 2- and 6-F or 3- and 5-F), –149.65- –149.70 (1F, m, 4-F), –161.55- –161.65 (2F, m, 2- and 6-F or 3- and 5-F). HRMS: $C_{17}H_{13}F_5N_2O_3S$ [M+H] requires: 421.0640; found: 421.0647.

3,5-Dimethyl-1-((1-((perfluorophenyl)sulfonyl)aziridin-2-yl)methyl)-1H-pyrazole (F24)

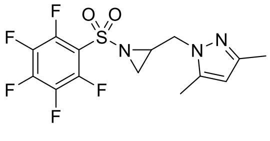

Prepared according to General Procedure A with substrate **S24**. The crude reaction mixture was purified by reverse phase MDAP-HPLC eluting with H_2O/CH_3CN (with 0.1% formic acid) over 12 min gave the *pyrazole* as a colourless solid (2.4 mg, 2%). ν_{max}/cm^{-1} 1643, 1561, 1518, 1489, 1417, 1325, 1292, 1152, 1094, 987, 921, 819, 643, 605, 580 and 530. δ_H (500 MHz, CD_3OD) 6.38 (1H, s, 4-H), 5.81-5.75 (1H, m, aziridinyl 2-

H), 5.21-5.18 (1H, m, methyl 1-H), 4.95-4.90 (3H, m, aziridinyl 3-H_2 and methyl 1-H), 2.38 (3H, s, methyl 3-H_3 or 5-H_3), 2.15 (3H, s, methyl 3-H_3 or 5-H_3). δ_C (125 MHz, CD_3OD) 146.3 (C-3 or C-5), 143.7 (C-3 or C-5), 131.1 (aziridinyl C-2), 118.8 (methyl C-1), 106.4 (C-4) 48.2 (aziridinyl C-3), 12.1 (methyl C-3 or C-5) 11.9 (methyl C-3 or C-5). Fluorinated carbons not visible due to low sample concentration. δ_F (376 MHz, CD_3OD) –138.74- –138.79 (2F, m, perfluorophenyl 2- and 6-F or 3- and 5-F), –152.09- –152.20 (1F, m, perfluorophenyl F-4), –162.81- –162.90 (2F, m, perfluorophenyl 2- and 6-F or 3- and 5-F). HRMS: $C_{14}H_{12}F_5N_3O_2S$ [M+H] requires: 382.0643; found: 382.0646.

(4,4-Difluoro-1-methylpiperidin-1-ium-1-yl)((perfluorophenyl)sulfonyl)amide (F25)

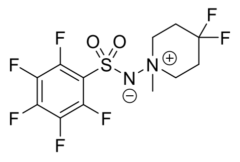

Prepared according to General Procedure A with substrate **S25**. The crude reaction mixture was purified by reverse phase MDAP-HPLC eluting with H_2O/CH_3CN (with 0.1% formic acid) over 12 min gave the *sulfonyl amide* as a colourless solid (9.4 mg, 8%). ν_{max}/cm^{-1} 1643, 1518, 1377, 1290, 1149, 1095, 1055, 1014, 985, 962, 834, 729, 702, 650, 607, 571 and 503. δ_H (500 MHz, CD_3OD) 4.00-3.96 (2H, m, piperidinyl 2 and 6-H^a), 3.57-3.52 (2H, m, piperidinyl 2- and 6-H^b), 3.37 (3H, s, methyl H_3), 2.88-2.74 (2H, dtt, *J* 33.4, 13.9 and 4.2 Hz, piperidinyl 3- and 5-H^a), 2.20-2.14 (2H, m, piperidinyl 3- and 5-H^b). δ_C (125 MHz, CD_3OD) 144.6 ($d^†$, *J* 258.1 Hz, perfluorophenyl C-2 and -6), 144.0 ($d^†$, *J* 263.7 Hz, perfluorophenyl C-4), 139.3 ($d^†$, *J* 258.1 Hz, perfluorophenyl C-3 and -5), 123.5 ($m^†$, perfluorophenyl , C-1), 124.4-118.6 (dd, *J* 244.1 and 239.2 Hz, piperidinyl C-4), 64.9 (d, *J* 10.5 Hz, piperidinyl C-2 and -6), 54.4 (methyl), 30.5-30.1 (dd, *J* 27.3 and 24.8 Hz, piperidinyl C-3 and -5). δ_F (376 MHz, CD_3OD) –98.9- –99.6 (1F, d, *J* 241.8 Hz, piperidinyl 4-F_A or 4-F_B), –103.5- –104.5 (1F, m, piperidinyl 4-F_A or 4-F_B), –139.71- –139.77 (2F, m, perfluorophenyl 3- and 5-F or 2- and 6-F), –154.04- –154.15 (1F, m, perfluorophenyl 4-F), –163.47- –163.57 (2F, m, perfluorophenyl 3- and 5-F or 2- and 6-F). HRMS: $C_{12}H_{11}F_7N_2O_2S$ [M+H] requires: 381.0502; found: 381.0504.

***N*-Methyl-*N*-(((perfluorophenyl)sulfonamido)methyl)cyclohexanecarboxamide (F35)**

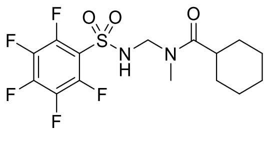

Prepared according to General Procedure A with substrate **S35**. The crude reaction mixture was purified by reverse phase MDAP-HPLC eluting with H_2O/CH_3CN (with 0.1% formic acid) over 12 min gave the *carboxamide* as a colourless solid (21 mg, 17%). ν_{max}/cm^{-1} 1636, 1520, 1497, 1452, 1413, 1358, 1299, 1171, 1100, 1058, 991, 607 and 578. δ_H (500 MHz, $CDCl_3$) 6.79 (1H, br. s, sulfonamide N*H*), 4.67 (2H, s, aminal methyl 1-

H_2), 3.10 (3H, s, aminal methyl H_3), 2.41-2.35 (1H, tt, *J* 11.5 and 3.4 Hz, 1-H), 1.80-1.77 (2H, m, 3- and 5-H_A), 1.69-1.66 (1H, m, 4-H^a), 1.64-1.61 (2H, m, 2- and 6-H^a), 1.42-1.35 (2H, m, 2- and 6-H^b), 1.29-1.20 (3H, m, 3- and 5-H^b and 4-H^b). δ_C (125 MHz, $CDCl_3$) 178.4 (carbonyl), 144.4 (ddq, *J* 263.0, 13.0 and 4.4 Hz, perfluorophenyl C-2 and -6), 143.9 (dtt, *J* 263.7, 13.4 and 5.4 Hz, perfluorophenyl C-4), 138.0 (dtt, *J* 258.1, 12.6 and 5.2 Hz, perfluorophenyl C-3 and -5), 117.9-117.6 (app. tt, *J* 15.2 and 2.2 Hz, perfluorophenyl C-1), 58.1 (aminal methyl C-1), 41.0 (C-1), 35.7 (methyl) 28.8 (C-2 and -6) 25.8 (C-4) 25.7 (C-3 and -5). δ_F (376 MHz, $CDCl_3$) -136.81- -136.88 (2F, m, perfluorophenyl 3- and 5-F or 2- and 6-F), -146.27- -146.38 (1F, m, perfluorophenyl 4-F), -158.57- -158.74 (2F, m, perfluorophenyl 3- and 5-F or 2- and 6-F). $C_{15}H_{17}F_5N_2O_3S$ [M+H] requires: 401.0953; found 401.0953.

_N_-(4-(2-Cyanoethyl)-6-methyl-3-oxo-3,4-dihydro-2H-benzo[b][1,4]oxazin-2-yl)-2,3,4,5,6-pentafluorobenzenesulfonamide (F37)

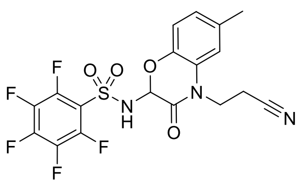

Synthesised according to General Procedure A with substrate **S37**. The crude reaction mixture was purified by reverse phase MDAP-HPLC eluting with H_2O/CH_3CN (with 0.1% formic acid) over 12 min to give the *sulfonamide* as a colourless solid (4 mg, 3%). ν_{max}/cm^{-1} 1695, 1521, 1500, 1450, 1373, 1300, 1175, 1102, 1026, 992, 816, 606 and 579. δ_H (500 MHz, CD_3OD) 7.13 (1H, s, benzoxazinyl 5-H), 6.85-6.83 (1H, d, *J* 8.1 Hz, benzoxazinyl 7-H), 6.45-6.44 (1H, d, *J* 8.1 Hz, benzoxazinyl 8-H), 5.86 (1H, s, benzoxazinyl 2-H), 4.34-4.22 (2H, m, cyanoethyl 1-H_2), 2.85-2.83 (2H, t, *J* 6.9 Hz, cyanoethyl 2-H_2), 2.35 (3H, s, methyl H_3). δ_C (125 MHz, CD_3OD) 161.6 (benzoxazinyl C-3), 140.6 (benzoxazinyl C-8a), 135.3 (benzoxazinyl C-6), 128.1 (benzoxazinyl C-4a), 126.3 (benzoxazinyl C-7), 118.7 (cyano *C*N), 118.4 (benzoxazinyl C-8), 117.0 (benzoxazinyl C-5), 80.8 (benzoxazinyl C-2), 38.7 (cyanoethyl C-1), 21.0 (methyl), 16.2 (cyanoethyl C-2). Fluorinated carbons not visible due to low sample concentration. δ_F (376 MHz, CD_3OD) –138.60- –138.66 (2F, m, 3- and 5-F or 2- and 6-F), –149.85- –149.96 (1F, m, 4-F), –162.70- –162.79 (2F, m, 3- and 5-F or 2- and 6-F). HRMS: $C_{18}H_{12}F_5N_3O_4S$ [M+Na] requires: 484.0361; found: 484.0359.

(E)-_N_-(1,4-Dicyanobut-3-en-2-yl)-2,3,4,5,6-pentafluorobenzenesulfonamide (F39)

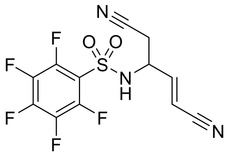

Prepared according to General Procedure A with substrate **S39**. The crude reaction mixture was purified by reverse phase MDAP-HPLC eluting with H_2O/CH_3CN (with 0.1% formic acid) over 12 min. Further purification by flash column chromatography was required, eluting with acetone/hexane 20:80, to give the *sulfonamide* as a colourless solid (2.4 mg, 2%). R_F = 0.08 (acetone/hexane 20:80). ν_{max}/cm^{-1} 1646.52, 1522.78, 1368.69, 1301.36, 1174.28, 1102.46, 992.43, 964.75, 728.84, 647.90, 609.74, 580.16, 543.88, 530.08, 488.70, 452.41, 440.31 and 431.59. δ_H (500 MHz, CD_3OD) 6.73-6.69 (1H, dd, *J* 16.3 and 5.6 Hz, alkenyl 3-H), 5.86-5.83 (1H, dd, *J* 16.3 and 1.7 Hz, alkenyl 4-H), 4.59-4.55 (1H, m, butenyl 2-H), 2.90-2.85 (1H, dd, *J* 17.0 and 4.8 Hz, butenyl 1-$H_{a\ or\ b}$), 2.73-2.67 (1H, dd, *J* 17.0 and 9.3 Hz, butenyl 1-$H_{a\ or\ b}$). δ_C (125 MHz, CD_3OD) 151.9 (butenyl C-3), 117.5 (cyano), 117.2 (cyano), 103.7 (butenyl C-4), 53.5 (butenyl C-2), 23.6 (butenyl C-1). Fluorinated carbons not visible due to low sample concentration. δ_F (376 MHz, CD_3OD) –138.63- –138.69 (2F, m, 3- and 5-F or 2- and 6-F), –150.4 (1F, m, 4-F), –162.67- –162.77 (2F, m, 3- and 5-F or 2- and 6-F).

(1-Isobutyl-1H-pyrazol-2-ium-2-yl)((perfluorophenyl)sulfonyl)amide (F44a)

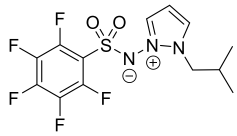

Prepared according to General Procedure A with substrate **S44a**. The crude reaction mixture was purified by reverse phase MDAP-HPLC eluting with H_2O/CH_3CN (with 0.1% formic acid) over 12 min. Further purification by column chromatography was required, eluting with acetone/hexane 20:80, to give the *amide* as a colourless solid (10 mg, 9%). R_F = 0.19 (acetone/hexane 30:70). ν_{max}/cm^{-1} 1723, 1642, 1518, 1491, 1379, 1317, 1293, 1246, 1147, 1096, 988, 927, 900, 823, 774, 727, 645, 599, 578 and 537. δ_H (500 MHz, $CDCl_3$) 7.57-7.55 (2H, m, pyrazolyl 3- and 5-H), 6.50 (1H, t, *J* 3.1 Hz, pyrazolyl 4-H), 4.20 (2H, d, *J* 7.6 Hz, isobutyl H_2), 2.40-2.29 (1H, m, isobutyl H), 0.94 (6H, d, *J* 6.7 Hz, isobutyl H_6). δ_C (125 MHz, $CDCl_3$) 144.4 (d†, *J* 263.7, perfluorophenyl C-2 and -6), 142.9 (d†, *J* 257.4 Hz, perfluorophenyl C-4), 137.8 (d†, *J* 257.4 Hz, perfluorophenyl C-3 and -5), 133.0 (pyrazolyl C-5), 130.3 (pyrazolyl C-3), 118.9-118.7 (m†, perfluorophenyl C-1), 104.8 (pyrazolyl C-4), 55.9 (isobutyl *C*H_2), 28.1 (isobutyl *C*H), 19.7 (isobutyl (*C*$H_3)_2$). δ_F (376 MHz, $CDCl_3$) –135.9- –136.0 (2F, m, perfluorophenyl 2- and 6-F or 3- and 5-F), –149.2- –149.3 (1F, m, perfluorophenyl F-4), –159.7- –159.8 (2F, m, perfluorophenyl 2- and 6-F or 3- and 5-F). HRMS: $C_{13}H_{12}F_5N_3O_2S$; requires: 370.0643; found: 370.0646.

2,3,4,5,6-Pentafluoro-*N*-(2-methyl-1-(1H-pyrazol-1-yl)propan-2-yl)benzenesulfonamide (F44b)

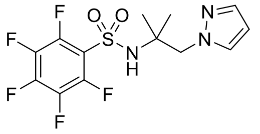

Prepared according to General Procedure A with substrate **S44b**. The crude reaction mixture was purified by reverse phase MDAP-HPLC eluting with H_2O/CH_3CN (with 0.1% formic acid) over 12 min gave the *sulfonamide* as a colourless solid (2 mg, 2%). ν_{max}/cm^{-1} 1644, 1519, 1497, 1397, 1355, 1296, 1249, 1167, 1099, 1029, 990, 869, 834, 758, 649, 609 and 575. δ_H (500 MHz, CD_3OD) 7.72 (1H, dd, *J* 2.3 and 0.4 Hz, pyrazolyl 5-H), 7.52 (1H, dd, *J* 2.0 and 0.5 Hz, pyrazolyl 3-H), 6.34 (1H, t, *J* 2.2 Hz, pyrazolyl 4-H), 4.30 (2H, s, propanyl 3-H_2), 1.24 (6, s, propanyl 1-H_3 and methyl H_3). δ_C (125 MHz, CD_3OD) 140.2 (pyrazolyl C-3), 132.9 (pyrazolyl C-5), 106.5 (pyrazolyl C-4), 61.8 (propanyl C-2), 59.0 (pyrazolyl C-3), 25.3 (propanyl C-1 and methyl). Fluorinated carbons not visible due to low sample concentration. δ_F (376 MHz, CD_3OD) –139.74- –139.79 (2F, m, 3- and 5-F or 2- and 6-F), –150.94- –151.04 (1F, m, 4-F), –162.71- –162.80 (2F, m, 3- and 5-F or 2- and 6-F). HRMS: $C_{13}H_{12}F_5N_3O_2S$ [M+H] requires: 370.0643; found: 370.0645.

2,3,4,5-Pentafluoro-*N*-(2-(*p*-tolyl)propan-2-yl)benzenesulfonamide (F48)

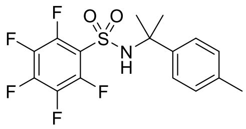

2,3,4,5,6-Pentafluorobenzenesulfonamide (247 mg, 1.0 mmol), 5 Å molecular sieves (300 mg), MgO (114 mg, 2.8 mmol), 2-phenylisobutyric acid (90 mg, 0.55 mmol) and $Rh_2(esp)_2$ (5.8 mg, 1 mol%) were added to a round bottom flask which was then sealed and flush with three alternating cycles of vacuum and nitrogen. A solution of *p*-cymene **S48** (120 µL, 0.8 mmol) in *i*-PrOAc (1.2 mL) was added to the solid reagents, the transfer was made complete with further *i*-PrOAc (1.0 mL). The mixture was allowed to stir for five min, unstoppered and $PhI(OAc)_2$ (496 mg, 1.5 mmol) was added. The flask was then resealed and flush with nitrogen. The reaction was allowed to stir overnight at room temperature under nitrogen and then filtered over Celite, the Celite cake was washed with EtOAc (10 mL) and the crude product was then purified by flash column chromatography, eluting with EtOAc/hexane 2:98 → 15:85, to yield the *sulfonamide* as a colourless solid (96 mg, 33%). R_F = 0.48 (EtOAc/hexane 30:70). ν_{max}/cm^{-1} 3297, 1723, 1518, 1497, 1351, 1292, 1167, 1147, 1095, 989, 820, 724, 612, 534. δ_H (500 MHz, $CDCl_3$) 7.19-7.17 (2H, m, tolyl 2- and 6-H_2), 6.97-6.95 (2H, m, tolyl 3- and 5-H_2), 5.32 (1H, s, sulfonamide N*H*), 2.25 (3H, s, tolyl H_3), 1.77 (6H, s, propanyl 1-H_3 and 3-H_3). δ_C (125 MHz, $CDCl_3$) 138.7 (tolyl C-4), 138.0 (tolyl C-1), 128.7 (tolyl C-3 and -5), 125.7 (tolyl C-2 and -6), 58.9 (propanyl C-2), 29.6 (propanyl C-1 and -3), 20.7 (tolyl *C*H_3). Fluorinated carbons not reported due to low sample concentration. δ_F (376 MHz, $CDCl_3$)

−136.58- −136.63 (2F, m, 3- and 5-F or 2- and 6-F), −148.23- −148.34 (1F, m, 4-F), −160.15- −160.25 (2F, m, 3- and 5-F or 2- and 6-F). HRMS: $C_{16}H_{14}F_5NO_2S$ [M+Na] requires: 402.0559; found: 402.0558.

1-((Perfluorophenyl)sulfonyl)-2-phenylaziridine (F49)

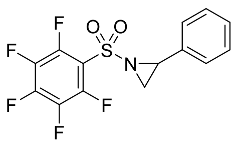

2,3,4,5,6-Pentafluorobenzenesulfonamide (247 mg, 1.0 mmol), 5 Å molecular sieves (200 mg), MgO (114 mg, 2.8 mmol), $Rh_2(esp)_2$ (5.8 mg, 1 mol%) and 2-phenylisobutyric acid (62 mg, 0.4 mmol) were added to an oven dried round bottom flask which was then flushed three times with alternating cycles of nitrogen and vacuum. A solution of styrene **S49** (88 µL, 0.8 mmol) in *i*-PrOAc (1 mL) was added to the solid reagents, the transfer was made complete with further *i*-PrOAc (0.5 mL). The reaction was allowed to stir for five min, unstoppered and $PhI(OAc)_2$ (496 mg, 1.5 mmol) was added to the mixture. The reaction vessel was resealed, flushed with nitrogen and the solution was stirred under nitrogen overnight. The reaction was then filtered over Celite, the cake was washed with EtOAc (10 mL), the crude mixture was then purified by flash column chromatography, eluting with EtOAc/hexane 2:98 → 5:95, to give the *phenylaziridine* as a colourless solid (110 mg, 41%). R_F = 0.42 (EtOAc/hexane 10:90). ν_{max}/cm^{-1} 1643, 1522, 1537, 1385, 1349, 1305.80, 1236, 1194, 1171, 1100, 993, 905, 786, 772, 730, 695, 627 and 542. δ_H (500 MHz, $CDCl_3$) 7.36-7.34 (3H, m, phenyl 3- and 5-H and phenyl 4-H), 7.28-7.26 (2H, m, phenyl 2- and 6-H), 4.07-4.04 (1H, dd, *J* 7.3 and 4.9 Hz, 2-H), 3.26 (1H, d, *J* 7.3 Hz, 1-H^a), 2.62 (1H, d, *J* 4.8 Hz, 1-H^b). δ_C (125 MHz, $CDCl_3$) 145.2 (ddq, *J* 263.0, 13.0 and 4.4 Hz, perfluorophenyl C-2 and -6), 145.0 (dtt, *J* 263.7, 13.4 and 5.4 Hz, C-4) 138.1 (d†, *J* 259 Hz, perfluorophenyl C-3 and -5) 134.0 (phenyl C-1), 129.05 (phenyl C-4), 128.95 (phenyl C-3 and -5), 126.7 (phenyl C-2 and -6), 114.8-114.9 (m†, perfluorophenyl C-1), 42.7 (C-2), 37.6 (C-1). δ_F (376 MHz, $CDCl_3$) −133.6- −134.9 (2F, m, perfluorophenyl 3- and 5-F or 2- and 6-F), −143.6- −143.8 (1F, m, perfluorophenyl 4-F), −158.0- −158.2 (2F, m, perfluorophenyl 3- and 5-F or 2- and 6-F). HRMS: $C_{14}H_8F_5NO_2S$ [M+H] requires: 350.0269; found: 350.0272.

2,3,4,5,6-Pentafluoro-*N*-(1,2,3,4-tetrahydronaphthalen-1-yl)benzenesulfonamide (F50)

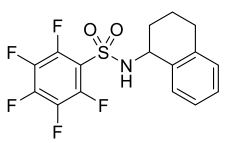

2,3,4,5,6-Pentafluorobenzenesulfonamide (247 mg, 1.0 mmol), 5 Å molecular sieves (200 mg), MgO (114 mg, 2.8 mmol), $Rh_2(esp)_2$ (5.8 mg, 1 mol%) and 2-phenylisobutyric acid (62 mg, 0.4 mmol) were added to a round bottom flask

which was then flushed three times with alternating cycles of nitrogen and vacuum. A solution of tetrahydronaphthalene **S50** (104 µL, 0.8 mmol) in *i*-PrOAc (1 mL) was added to the solid reagents, the transfer was made complete with further *i*-PrOAc (0.5 mL). The reaction was allowed to stir for five min, unstoppered and $PhI(OAc)_2$ (496 mg, 1.5 mmol) was added to the mixture. The reaction vessel was resealed, flushed with nitrogen and the solution was stirred under nitrogen overnight. The reaction was then filtered over Celite and the Celite cake was washed with EtOAc (10 mL), the crude was purified by flash column chromatography, eluting with EtOAc/hexane 2:98. A further purification by reverse phase MDAP-HPLC was required eluting with H_2O/CH_3CN (with 0.1% formic acid) over 12 min to give the *sulfonamide* as an off-white solid (7 mg, 3%). R_F = 0.30 (EtOAc/hexane 10:90). ν_{max}/cm^{-1} 3309, 1644, 1519, 1492.60, 1362, 1299, 1172, 1100, 991, 907, 762, 735, 650, 605, 579 and 530. δ_H (500 MHz, $CDCl_3$) 7.22-7.18 (1H, m, naphthalenyl 5-H), 7.16-7.14 (2H, m, naphthalenyl 7- and 8-H), 7.10 (1H, d, *J* 7.5 Hz, naphthalenyl 8-H), 5.25 (1H, d, *J* 8.1 Hz, sulfonamide N*H*), 4.79-4.75 (1H, dt, *J* 8.2 and 5.3 Hz naphthalenyl 1-H), 2.85-2.79 (1H, dt, *J* 16.9 and 5.8 Hz, naphthalenyl 4-H^a), 2.76-2.70 (1H, dt, *J* 14.4 and 6.9 Hz, naphthalenyl 4-H^b), 1.98-1.89 (2H, m, naphthalenyl 2-H_2), 1.87-1.80 (2H, m, naphthalenyl 3-H_2). δ_C (125 MHz, $CDCl_3$) 144.5 (ddq, *J* 263.0, 13.0 and 4.4 Hz, C-2 and -6), 144.0 ($d^†$, *J* 263.7 Hz, C-4), 138.0 ($d^†$, *J* 257.4 Hz, C-3 and -5), 137.6 (naphthalenyl C-4a), 134.2 (naphthalenyl C-8a), 129.7 (naphthalenyl C-8), 128.8 (naphthalenyl C-7 or C-8), 128.4 (naphthalenyl C-5), 126.6 (naphthalenyl C-7 or C-8) 117.8 ($m^†$, C-1) 53.3 (naphthalenyl C-1), 30.5 (naphthalenyl C-2), 28.7 (naphthalenyl C-4), 19.8 (naphthalenyl C-3). δ_F (376 MHz, $CDCl_3$) −136.52- −136.57 (2F, m, 3- and 5-F or 2- and 6-F), −146.02- −146.08 (1F, m, 4-F), −158.41- −158.51 (2F, m, 3- and 5-F or 2- and 6-F). HRMS: $C_{16}H_{12}F_5NO_2S$ [M+Na] requires: 400.0401; found: 400.0406.

2-Methyl-1-((perfluorophenyl)sulfonyl)-3-phenylaziridine (F51)

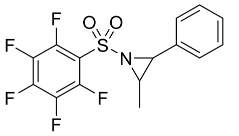

2,3,4,5,6-Pentafluorobenzenesulfonamide (247 mg, 1 mmol), 5 Å molecular sieves (200 mg), MgO (114 mg, 2.85 mmol), $Rh_2(esp)_2$ (5.8 mg, 1 mol%) and 2-phenylisobutyric acid (62 mg, 0.38 mmol) were added to a round bottom flask which was then flushed three times with alternating cycles of nitrogen and vacuum. A solution of trans-β-methylstyrene **S51** (100 µL, 0.77 mmol) in *i*-PrOAc (1 mL) was added to the solid reagents, the transfer was made complete with further *i*-PrOAc (0.5 mL). The reaction was allowed to stir for five min, unstoppered and $PhI(OAc)_2$ (496 mg, 1.54 mmol) was added to the mixture. The reaction vessel was resealed, flushed with nitrogen and the solution was stirred under nitrogen overnight. The reaction was then filtered over Celite and the crude was purified by flash column chromatography,

eluting with EtOAc/hexane 2:98 → 5:95, to give the *phenylaziridine* as an off-white solid (174 mg, 48%). R_F = 0.42 (EtOAc/hexane 10:90). ν_{max}/cm^{-1} 1644, 1520, 1496, 1354, 1301, 1167, 1100, 1036, 991, 890, 794, 749, 729, 700, 647, 627 and 546. δ_H (500 MHz, $CDCl_3$) 7.34-7.30 (3H, m, phenyl 3- and 5-H and phenyl 4-H), 7.24-7.21 (2H, m, phenyl 2- and 6-H), 4.00 (1H, d, *J* 4.6 Hz, 3-H), 3.18-3.13 (1H, m, 2-H), 1.88-1.86 (3H, d, *J* 6.0 Hz, methyl). δ_C (125 MHz, $CDCl_3$) 44.7 (ddq, *J* 263.0, 13.0 and 4.4 Hz, perfluorophenyl C-2 and -6), 144.4 (dtt, *J* 263.7, 13.4 and 5.4 Hz, C-4) 138.0 (d†, *J* 259 Hz, C-3 and -5), 134.6 (phenyl C-1), 128.9 (phenyl C-3 and -5), 128.8 (phenyl C-4), 126.4 (phenyl C-2 and -6), 117.0-116.8 (m†, perfluorophenyl C-1), 51.6 (C-3 or C-2), 51.3 (C-2 or C-3), 15.2 (C-1). δ_F (376 MHz, $CDCl_3$) –135.37- –135.43 (2F, m, perfluorophenyl 3- and 5-F or 2- and 6-F), –144.95- –145.06 (1F, m, perfluorophenyl 4-F), –158.58- –158.74 (2F, m, perfluorophenyl 3- and 5-F or 2- and 6-F). HRMS: $C_{15}H_{10}F_5NO_2S$ [M+H] requires: 364.0425; found: 364.0429.

N-Cyclohexyl-2,3,4,5,6-pentafluorobenzenesulfonamide (F52)

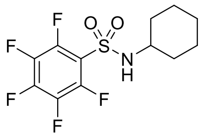

2,3,4,5,6-Pentafluorobenzenesulfonamide (247 mg, 1.0 mmol), 5 Å molecular sieves (200 mg), MgO (114 mg, 2.8 mmol), $Rh_2(esp)_2$ (5.8 mg, 1 mol%) and 2-phenylisobutyric acid (62 mg, 0.4 mmol) were added to a round bottom flask which was then flushed three times with alternating cycles of nitrogen and vacuum. A solution of cyclohexane **S52** (83 µL, 0.8 mmol) in *i*-PrOAc (1.0 mL) was added to the solid reagents, the transfer was made complete with further *i*-PrOAc (0.5 mL). The reaction was allowed to stir for five min, unstoppered and $PhI(OAc)_2$ (496 mg, 1.5 mmol) was added to the mixture. The reaction vessel was resealed, flushed with nitrogen and the solution was stirred under nitrogen overnight. The reaction was then filtered over Celite and the Celite cake was washed with EtOAc (10 mL), the crude was purified by flash column chromatography, eluting with EtOAc/hexane 2:98. A further purification by reverse phase MDAP-HPLC was required eluting with H_2O/CH_3CN (with 0.1% formic acid) over 12 min to give the *sulfonamide* as an amber oil (3 mg, 1%). R_F = 0.28 (EtOAc/hexane 10:90). δ_H (500 MHz, $CDCl_3$) 4.95 (1H, br. d, *J* 7.4 Hz, sulfonamide N*H*), 3.44-3.37 (1H, m, cyclohexyl 1-H), 1.88-1.85 (2H, m, cyclohexyl 2- and 6-H^a), 1.71-1.69 (2H, m, cyclohexyl 3- and 5-H^a), 1.60-1.57 (1H, m, cyclohexyl 4-H^a), 1.36-1.11 (5H, m, cyclohexyl 3- and 5-H^a, 4-H^a, 2- and 6-H^a). δ_C (125 MHz, $CDCl_3$) 53.9 (cyclohexyl C-1), 33.9 (cyclohexyl C-2 and -6), 25.1 (cyclohexyl C-3 and -5), 24.7 (cyclohexyl C-4). Fluorinated carbons not visible due to low sample concentration. δ_F (376 MHz, $CDCl_3$) –137.06- –137.11 (2F, m, 3- and -5-F or 2- and 6-F), –146.36- –146.47 (1F, m, 4-F), –158.50- –158.60 (2F, m, 3- and 5-F or 2- and 6-F). Values are consistent with reported literature[5].

2,3,4,5,6-Pentafluoro-*N*-(1-phenylethyl)benzenesulfonamide (F53)

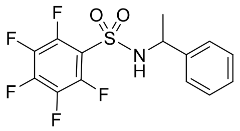

2,3,4,5,6-Pentafluorobenzenesulfonamide (321 mg, 1.4 mmol), MgO (164 mg, 4.1 mmol), 5 Å molecular sieves (300 mg), 2-phenylisobutyric acid (90 mg, 0.55 mmol) and $Rh_2(esp)_2$ (8.4 mg, 1 mol%) were added to a round bottom flask which was then sealed and flush with three alternating cycles of vacuum and nitrogen. A solution of ethylbenzene **S53** (136 µL, 1.1 mmol) in *i*-PrOAc (1.2 mL) was added to the solid reagents, the transfer was made complete with further *i*-PrOAc (1.0 mL). The mixture was allowed to stir for five min, unstoppered and $PhI(OAc)_2$ (715 mg, 2.2 mmol) was added. The flask was then resealed and flush with nitrogen. The reaction was allowed to stir overnight at room temperature under nitrogen and then filtered over Celite, the Celite cake was washed with EtOAc (10 mL) and the crude product was then purified by flash column chromatography, eluting with EtOAc/hexane 10:90, to yield the *sulfonamide* as a colourless solid (173 mg, 55%). R_F = 0.28 (EtOAc/hexane 10:90). ν_{max}/cm^{-1} 3289, 1519, 1426, 1352, 1295, 1163, 1096, 1020, 991, 881, 767, 702, 623, 602 and 531. δ_H (500 MHz, $CDCl_3$) 7.21-7.17 (3H, m, phenyl 3- and 5-H and phenyl 4-H), 7.13-7.11 (2H, m, phenyl 2- and 6-H), 5.36 (1H, d, *J* 8.4 Hz, sulfonamide N*H*), 4.73-4.67 (1H, dq, *J* 14.2 and 7.0 Hz, ethyl 1-H), 1.58 (3H, d, *J* 7.0 Hz, ethyl 2-H_3). δ_C (125 MHz, $CDCl_3$) 144.1 (ddq, *J* 263.0, 13.0 and 4.4 Hz, C-2 and -6), 143.6 (dtt, *J* 263.7, 13.4 and 5.4 Hz, C-4), 139.9 (phenyl C-1), 137.5 (d†, *J* 257.4 Hz, C-3 and -5), 128.8 (phenyl C-3 and -5), 128.4 (phenyl C-4), 126.1 (phenyl C-2 and -6), 116.9 (app. tq, *J* 14.3, 4.0 and 1.6 Hz, C-1), 55.0 (benzylic C-1), 22.9 (benzylic C-2). δ_F (376 MHz, $CDCl_3$) –133.97- –134.02 (2F, m, 3- and 5-F or 2- and 6-F), –143.65- –143.76 (1F, m, 4-F), –158.05- –158.15 (2F, m, 3- and 5-F or 2- and 6-F). HRMS: $C_{14}H_{10}F_5NO_2S$ [M+Na] requires: 374.0245; found: 374.0250.

2,3,4,5,6-Pentafluoro-*N*-(prop-2-yn-1-yl)benzenesulfonamide (Probe 1)

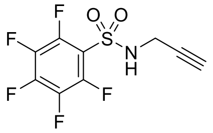

Propargylamine hydrochloride (101 mg, 1.1 mmol) was transferred into a round bottom flask containing K_2CO_3 (166 mg, 1.2 mmol) and CH_3CN (5 mL). The mixture was stirred for ten min and cooled to 0 °C in an ice bath. 2,3,4,5,6-Pentafluorosulfonyl chloride (150 µL, 1.0 mmol) was then added to the solution drop-wise. The reaction was brought to room temperature and allowed to stir overnight. The solvent was then removed under reduced pressure and the resulting residue was dissolved in CH_2Cl_2 (10 mL). The crude

mixture was transferred into a separation funnel and washed with an aqueous hydrochloric acid solution (50 mL, 0.1 M) followed by a saturated solution of aqueous $NaHCO_3$ (50 mL) and brine (40 mL). The organic layer was dried over Na_2SO_4, filtered and the solvent was removed under reduced pressure to give an oil which was purified by flash column chromatography, eluting with EtOAc/hexane 15:85, to give the *sulfonamide* as a colourless solid (50 mg, 17%). ν_{max}/cm^{-1} 3319, 3292, 1644, 1520, 1424, 1366, 1352.12, 1300, 1180, 1099, 1084, 1068, 989, 930, 860, 818, 702, 642, 618, 601, 580 and 523. δ_H (500 MHz, $CDCl_3$) 5.29 (1H, br. s, sulfonamide N*H*), 4.08-4.06 (2H, dd, *J* 6.3 and 2.4 Hz, propynyl 1-H_2), 2.15 (1H, t, *J* 2.4 Hz, propynyl 3-H). δ_C (125 MHz, $CDCl_3$) 145.6 (ddq, *J* 263.0, 13.0 and 5.4 Hz, C-2 and -6), 144.3 (dtt, *J* 263.7, 13.4 and 5.4 Hz, C-4), 138 (d†, *J* 259.0 Hz, C-3 and -5), 116.6-116.3 (tq, *J* 14.3, 4.0 and 1.6 Hz, C-1), 76.6 (propynyl C-2), 73.7 (propynyl C-3), 33.2 (propynyl C-1). δ_F (376 MHz, $CDCl_3$) –136.23- –136.29 (2F, m, 3- and 5-F or 2- and 6-F), –145.39- -145.50 (1F, m, 4-F), –158.66- –158.76 (2F, m, 3- and 5-F or 2- and 6-F).

4.5 NMR Data

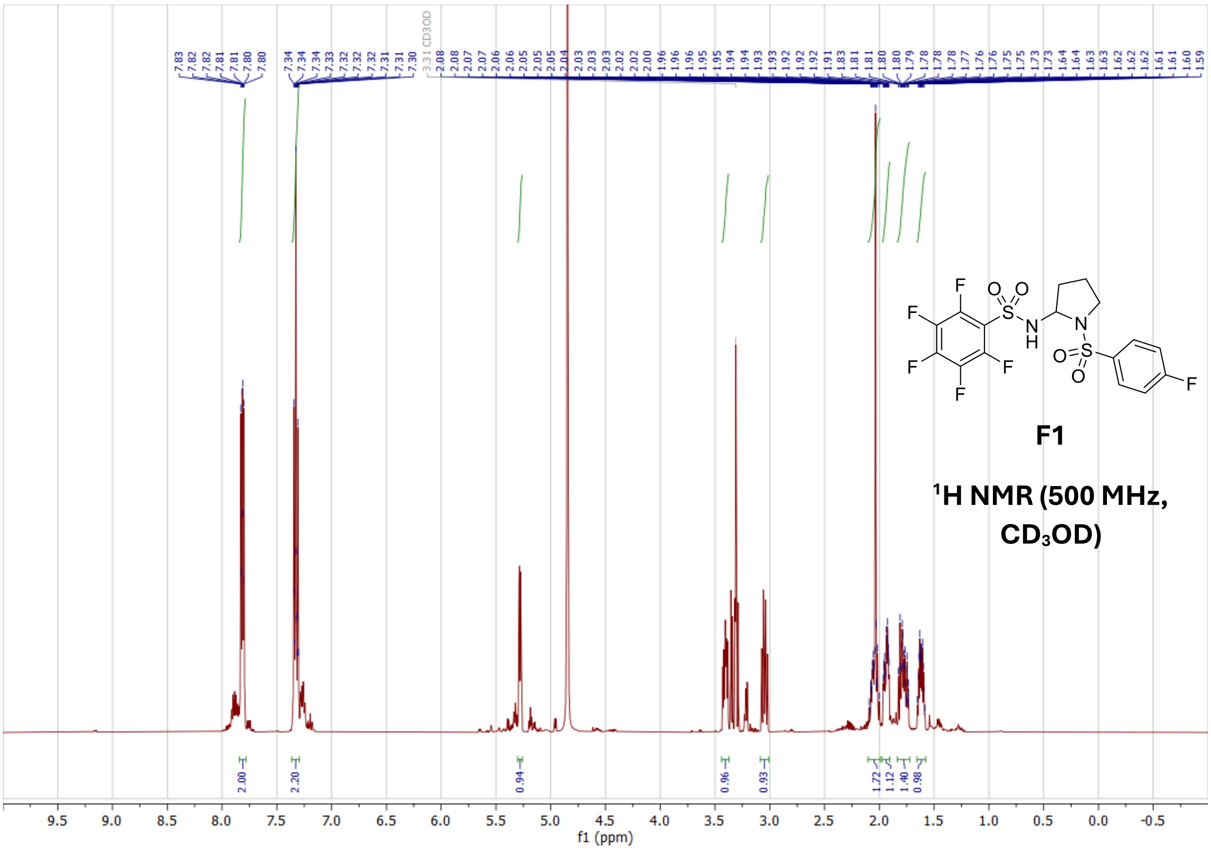

F1

^{1}H NMR (500 MHz, CD_3OD)

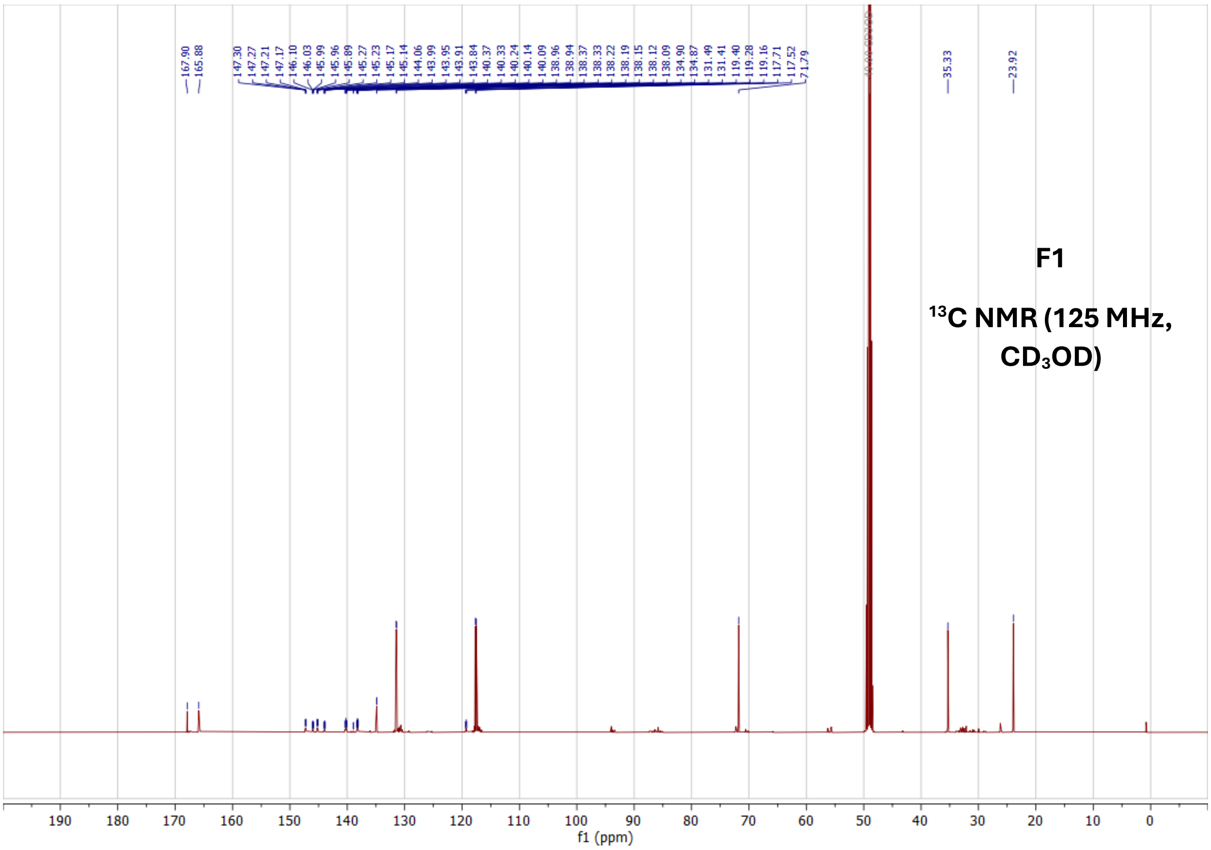

F1

^{13}C NMR (125 MHz, CD_3OD)

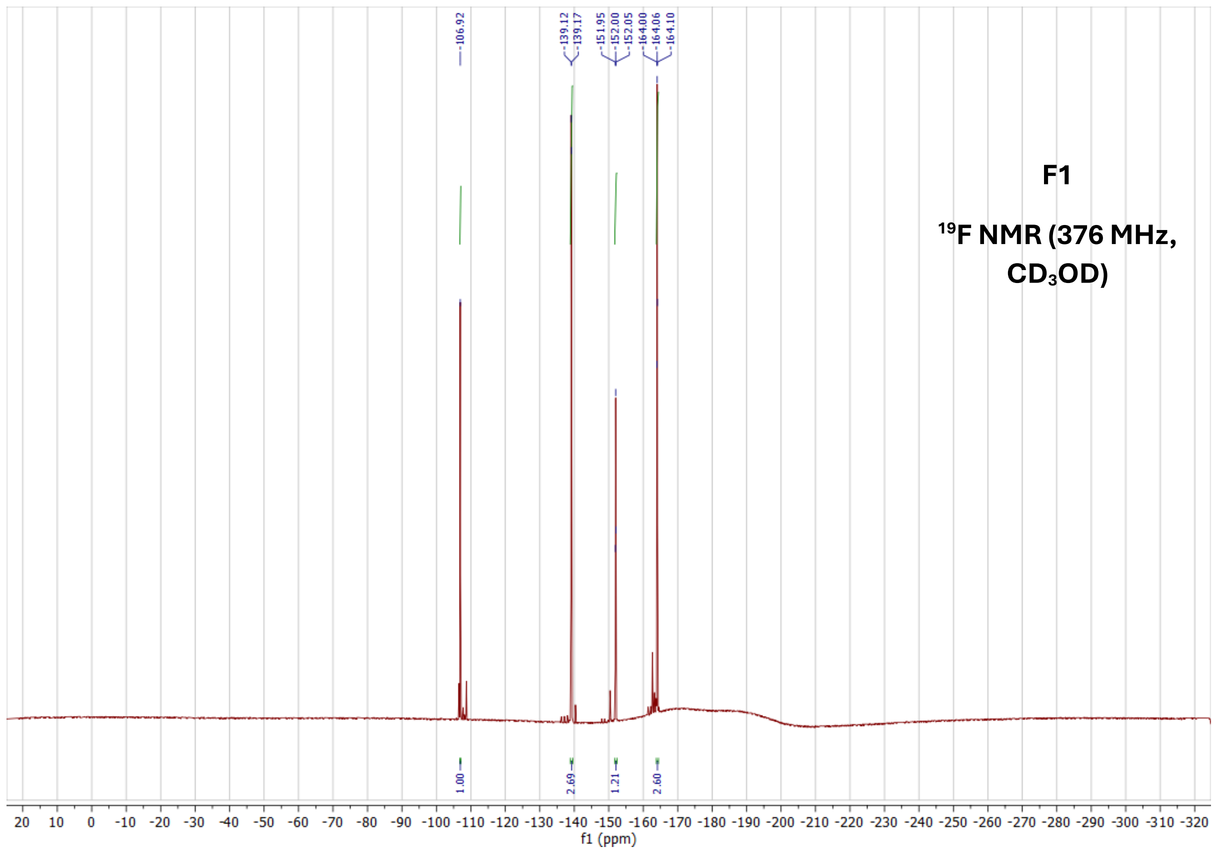
-106.92
-139.12
-139.17
-151.95
-152.00
-152.05
-164.00
-164.06
-164.10
F1
19F NMR (376 MHz, CD3OD)
1.00
2.69
1.21
2.60
20 10 0 -10 -20 -30 -40 -50 -60 -70 -80 -90 -100 -110 -120 -130 -140 -150 -160 -170 -180 -190 -200 -210 -220 -230 -240 -250 -260 -270 -280 -290 -300 -310 -320
f1 (ppm)

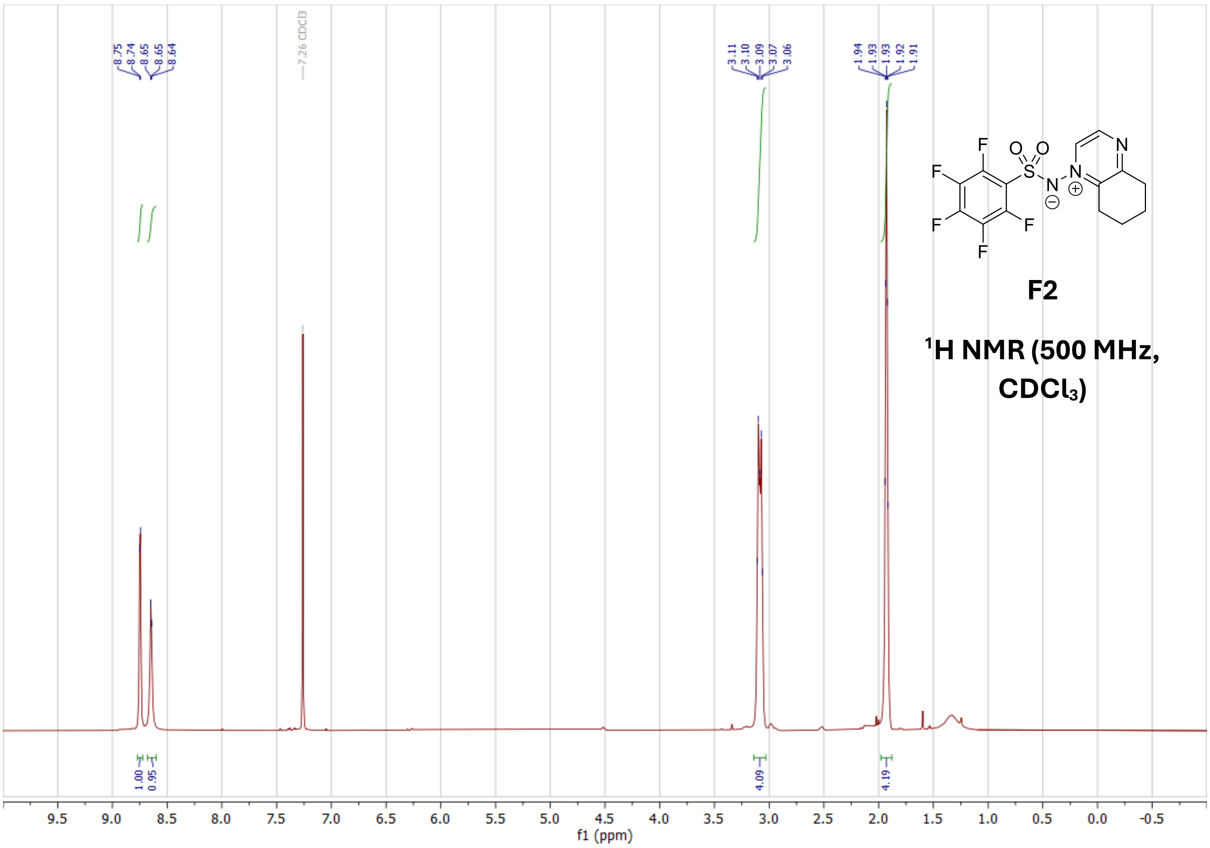
F2
1H NMR (500 MHz, CDCl3)
7.26 CDCl3
1.00
0.95
4.09
4.19
f1 (ppm)

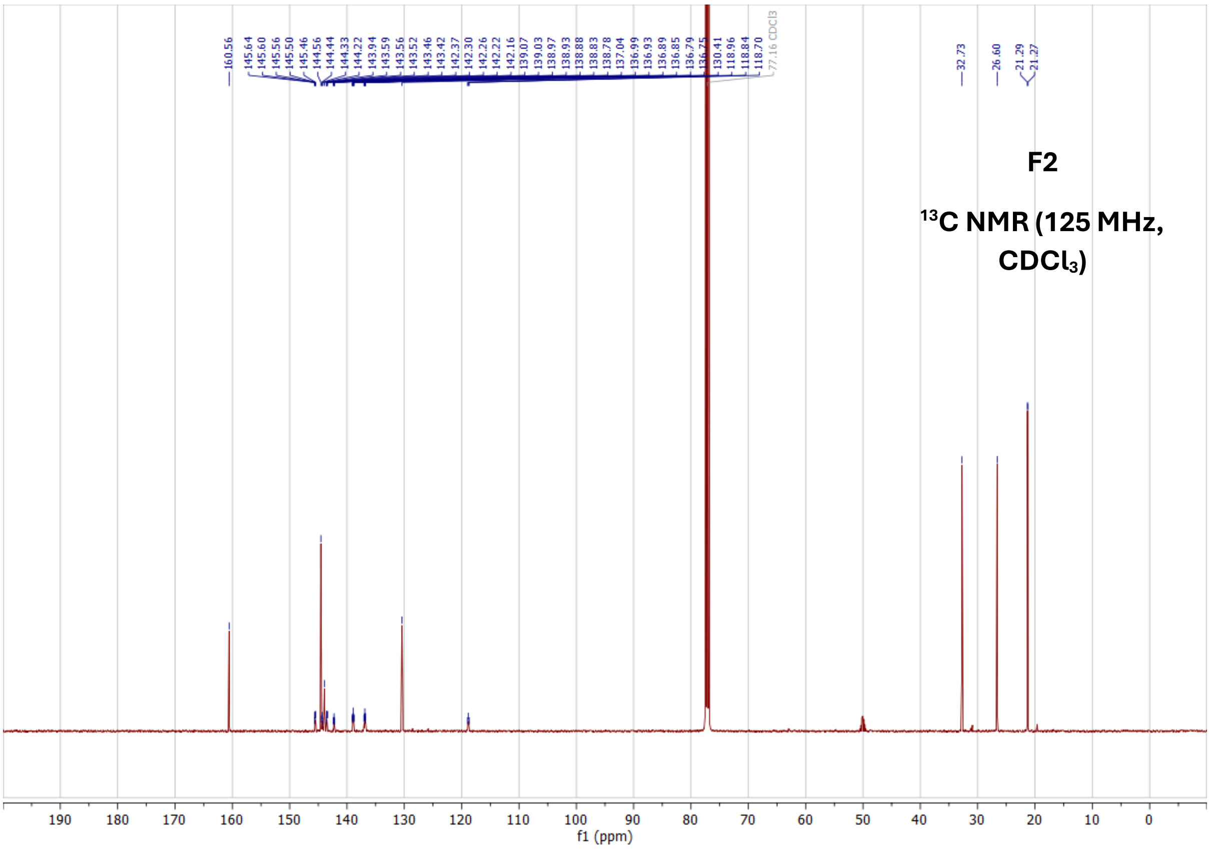
F2
13C NMR (125 MHz, CDCl3)
77.16 CDCl3
f1 (ppm)

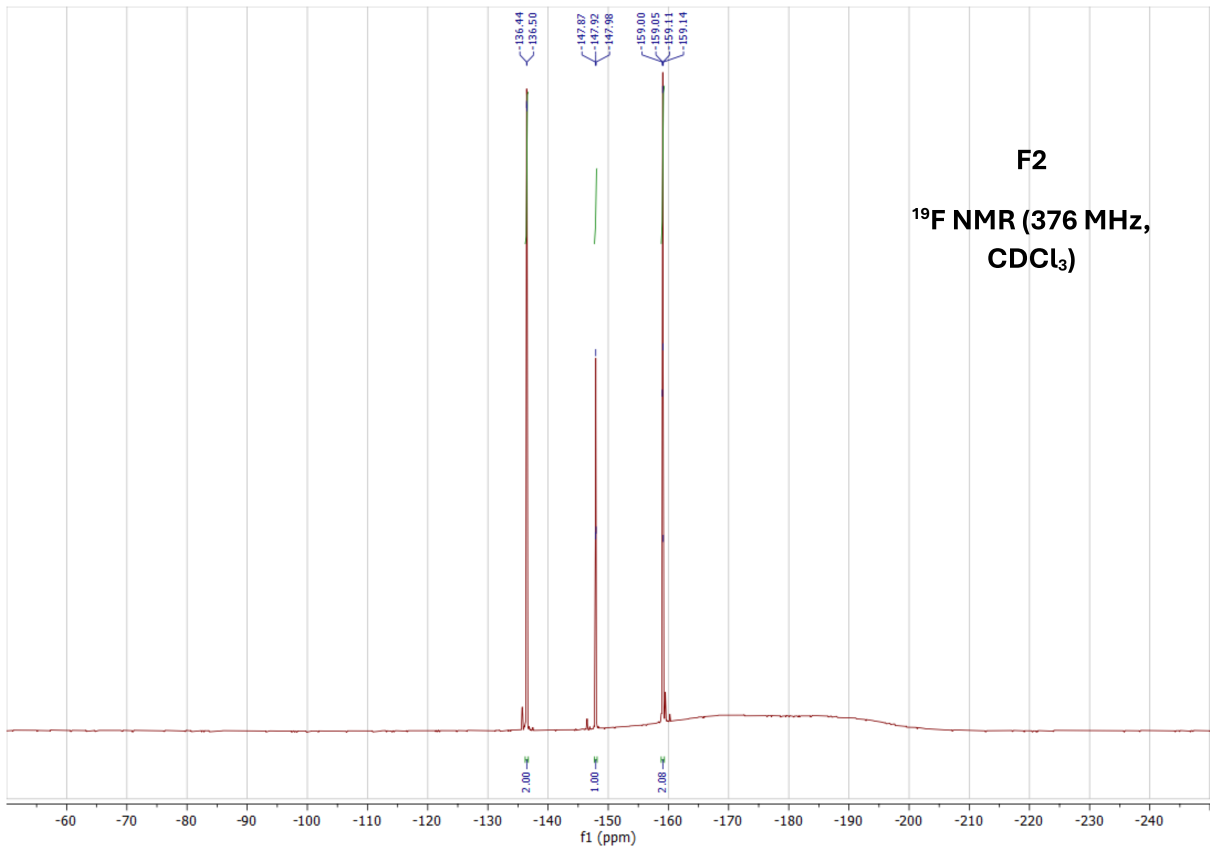
F2
^{19}F NMR (376 MHz, $CDCl_3$)
-136.44
-136.50
-147.87
-147.92
-147.98
-159.00
-159.05
-159.11
-159.14
2.00
1.00
2.08
f1 (ppm)

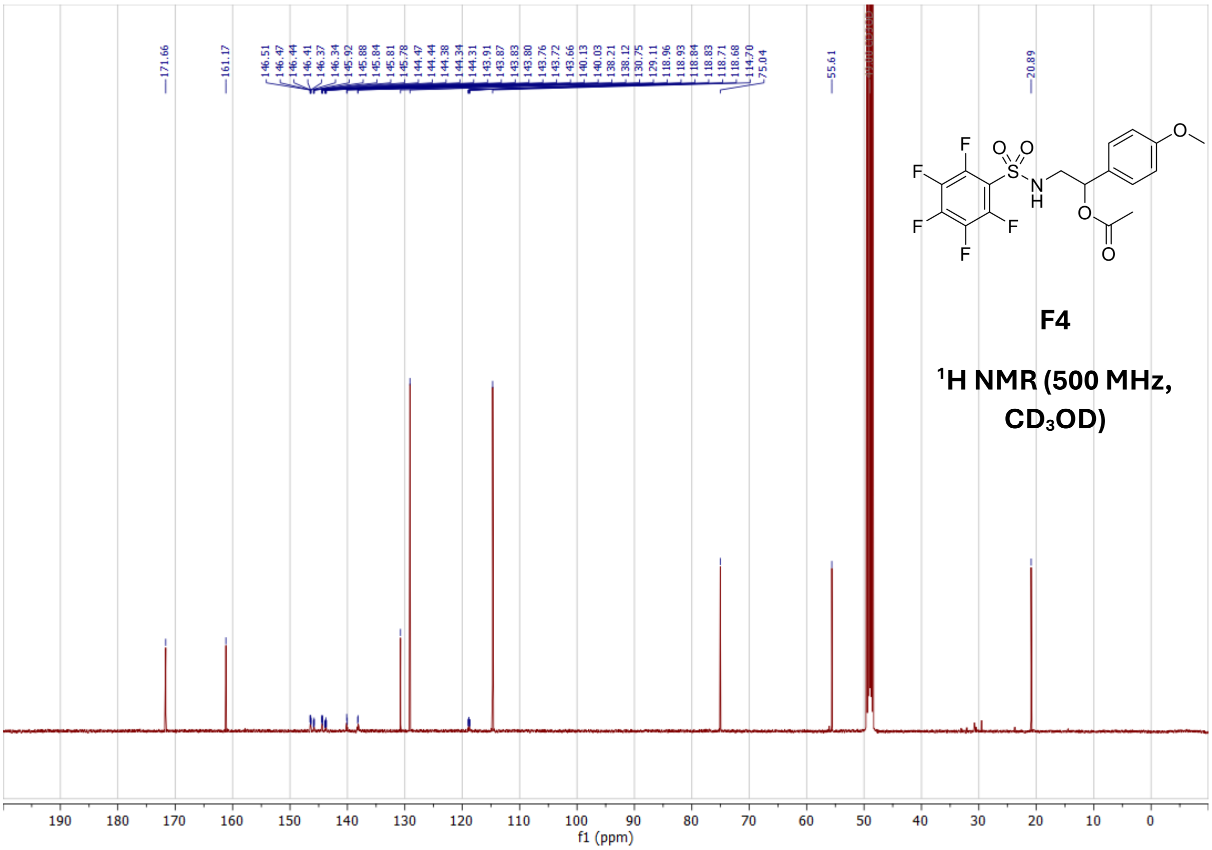
171.66
161.17
146.51
146.47
146.44
146.41
146.37
146.34
145.92
145.88
145.84
145.81
145.78
144.47
144.44
144.38
144.34
144.31
143.91
143.87
143.83
143.80
143.76
143.72
143.66
140.13
140.03
138.21
138.12
130.75
129.11
118.96
118.93
118.84
118.83
118.71
118.68
114.70
75.04
55.61
20.89
F
F
F
F
F
O
O
S
N
H
O
O
O
F4
^{1}H NMR (500 MHz, CD$_3$OD)
f1 (ppm)

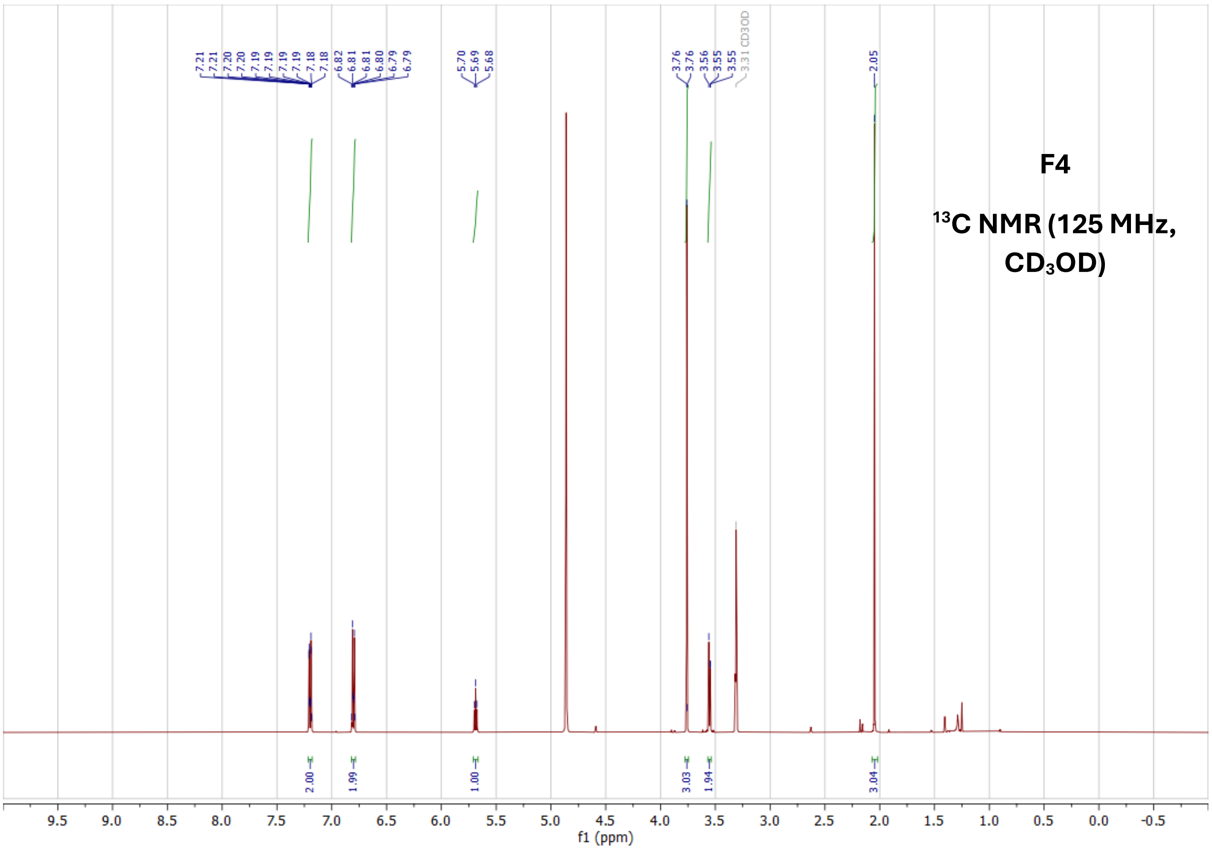
7.21
7.21
7.20
7.19
7.19
7.19
7.19
7.18
7.18
6.82
6.81
6.81
6.80
6.79
6.79
5.70
5.69
5.68
3.76
3.76
3.56
3.55
3.55
3.31 CD3OD
2.05
F4
^{13}C NMR (125 MHz, CD$_3$OD)
2.00
1.99
1.00
3.03
1.94
3.04
f1 (ppm)

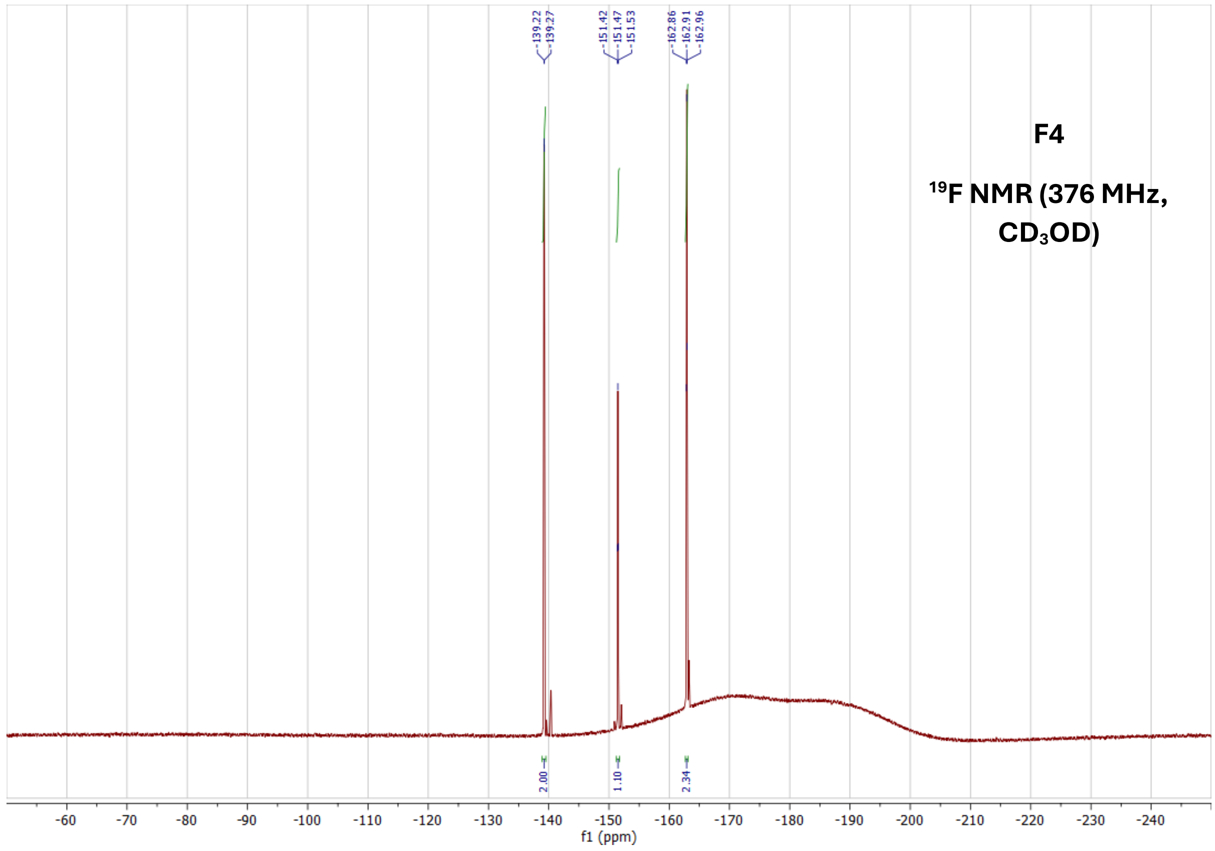

-139.22
-139.27
-151.42
-151.47
-151.53
-162.86
-162.91
-162.96
F4
19F NMR (376 MHz, CD3OD)
2.00
1.10
2.34
-60 -70 -80 -90 -100 -110 -120 -130 -140 -150 -160 -170 -180 -190 -200 -210 -220 -230 -240
f1 (ppm)

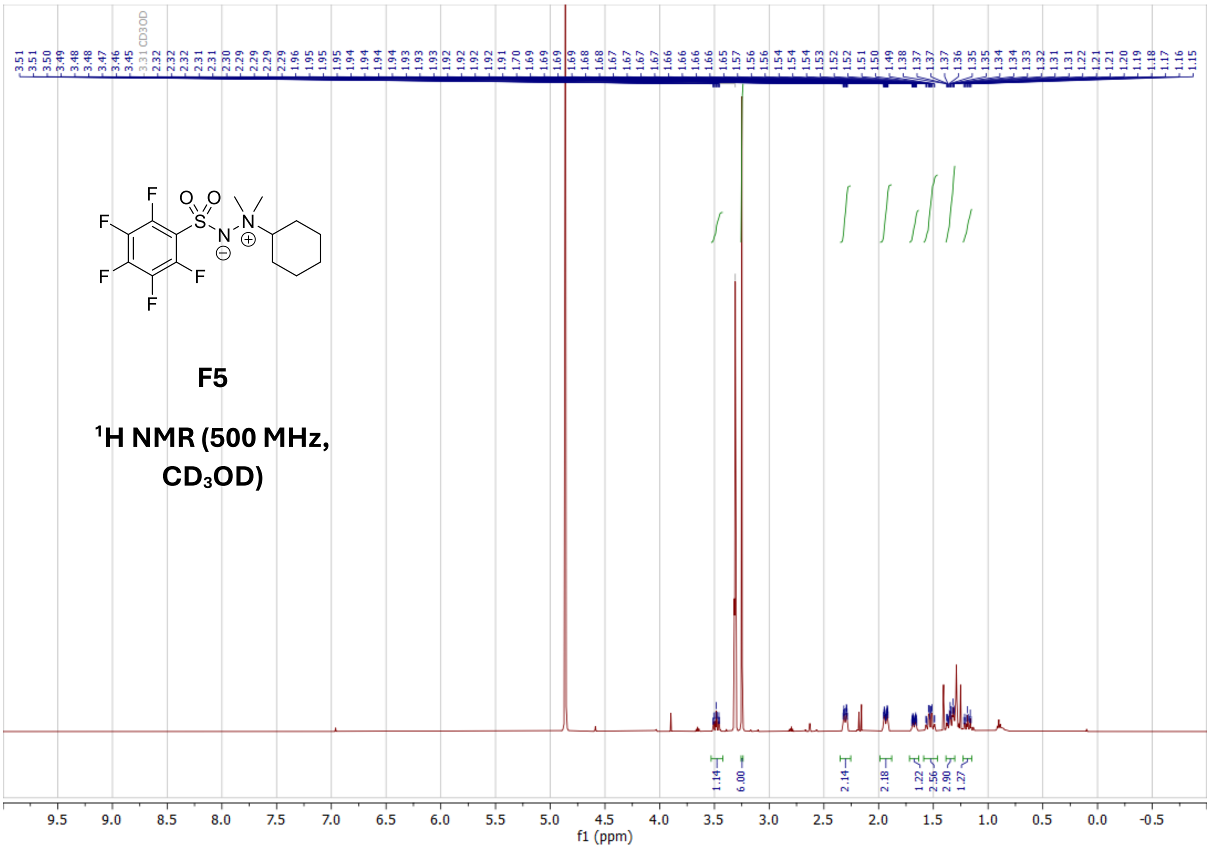
F5
1H NMR (500 MHz, CD3OD)
1.14
6.00
2.14
2.18
1.22
2.56
2.90
1.27
f1 (ppm)

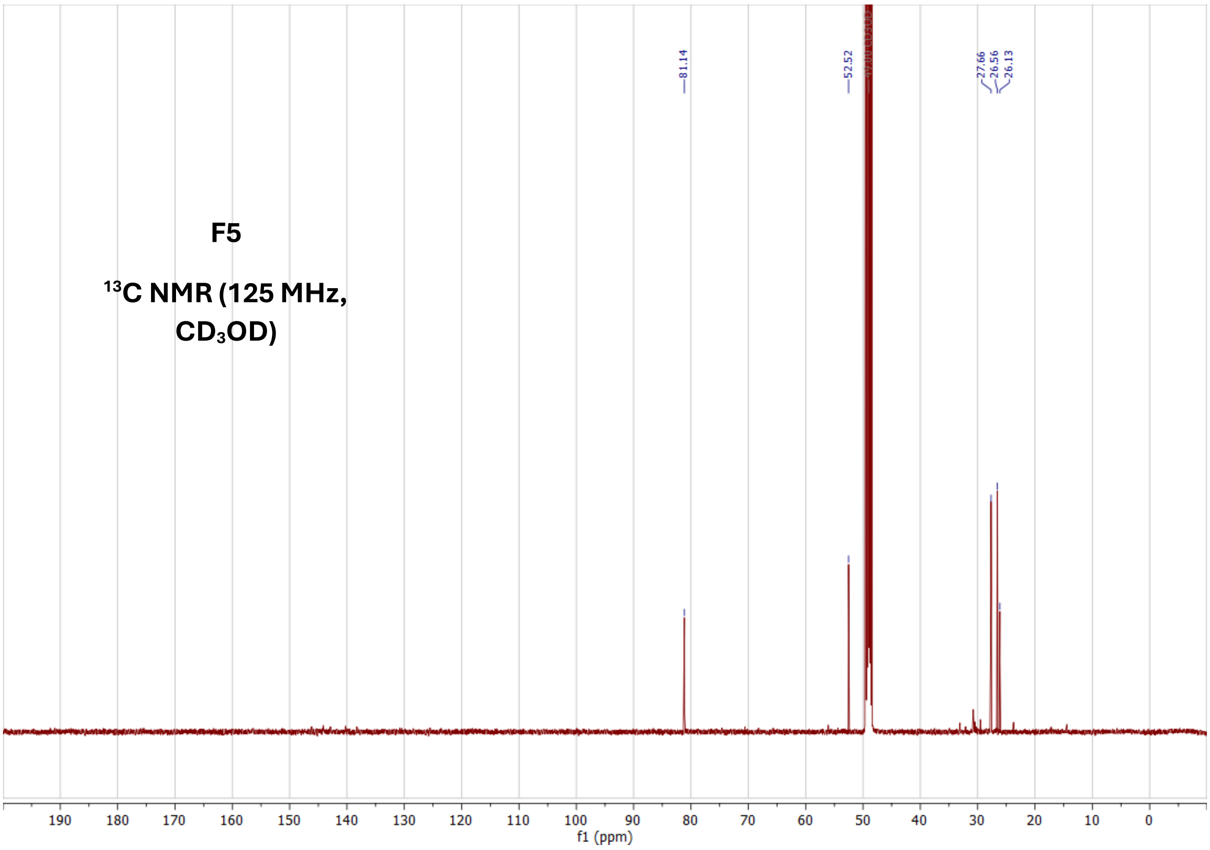
F5
13C NMR (125 MHz, CD3OD)
81.14
52.52
27.66
26.56
26.13
f1 (ppm)

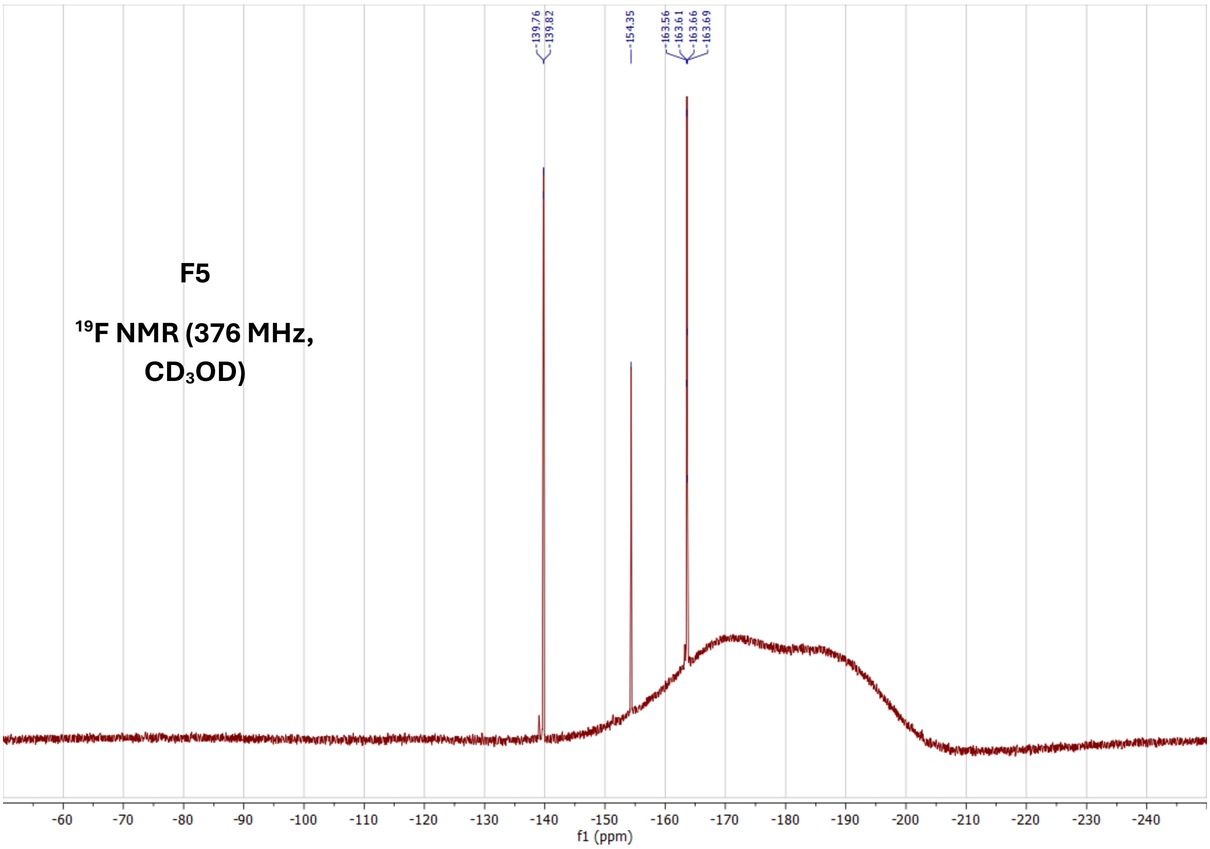
F5
^{19}F NMR (376 MHz, CD_3OD)
-139.76
-139.82
-154.35
-163.56
-163.61
-163.66
-163.69
-60 -70 -80 -90 -100 -110 -120 -130 -140 -150 -160 -170 -180 -190 -200 -210 -220 -230 -240
f1 (ppm)

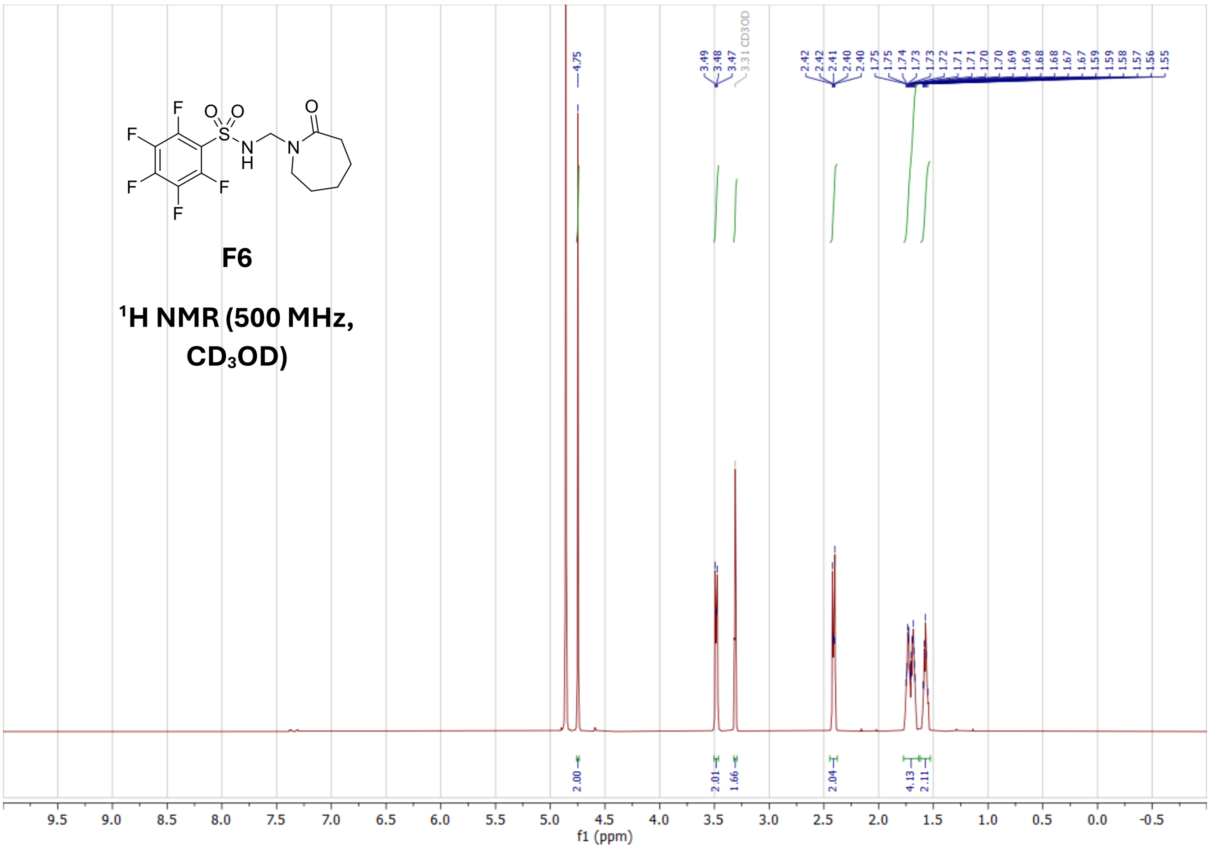

F6

^{1}H NMR (500 MHz, CD_3OD)

F6

^{13}C NMR (125 MHz, CD_3OD)

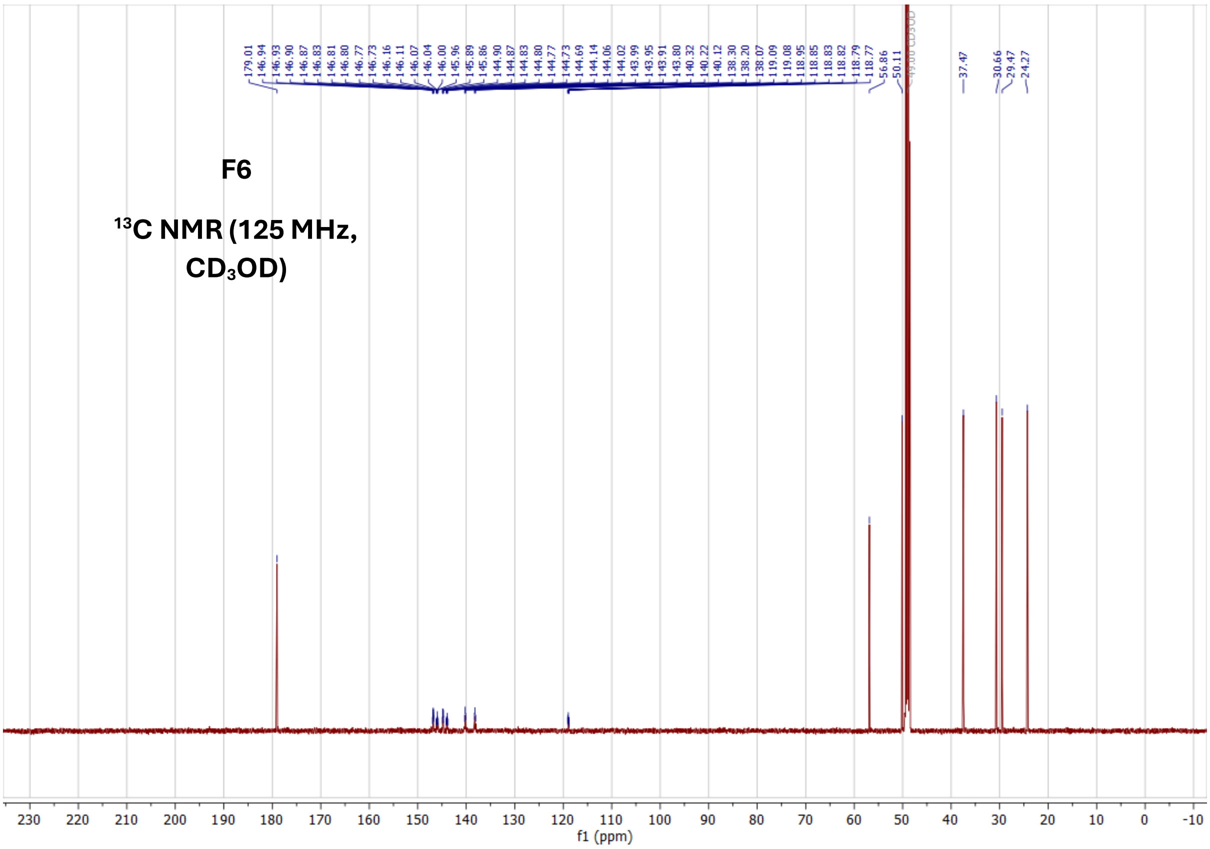

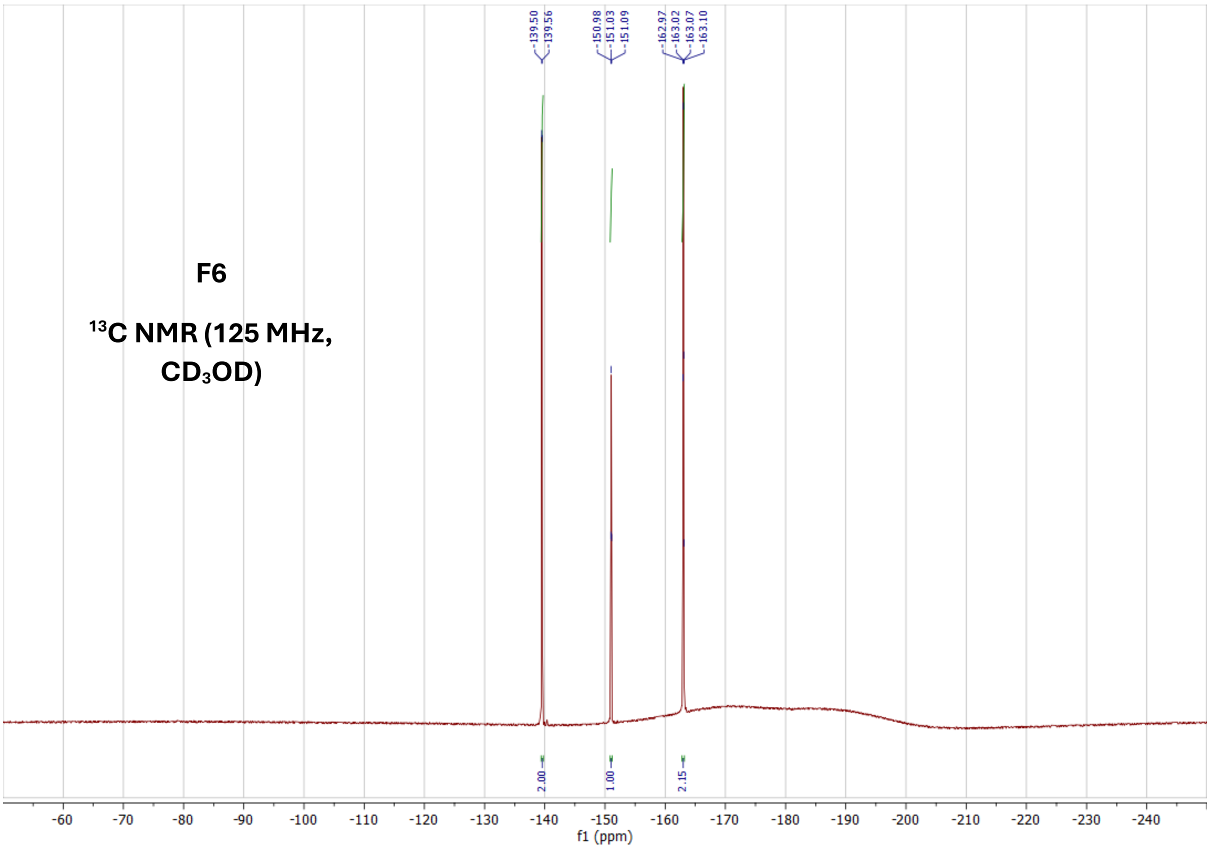
F6
13C NMR (125 MHz, CD3OD)
-139.50
-139.56
-150.98
-151.03
-151.09
-162.97
-163.02
-163.07
-163.10
2.00
1.00
2.15
-60 -70 -80 -90 -100 -110 -120 -130 -140 -150 -160 -170 -180 -190 -200 -210 -220 -230 -240
f1 (ppm)

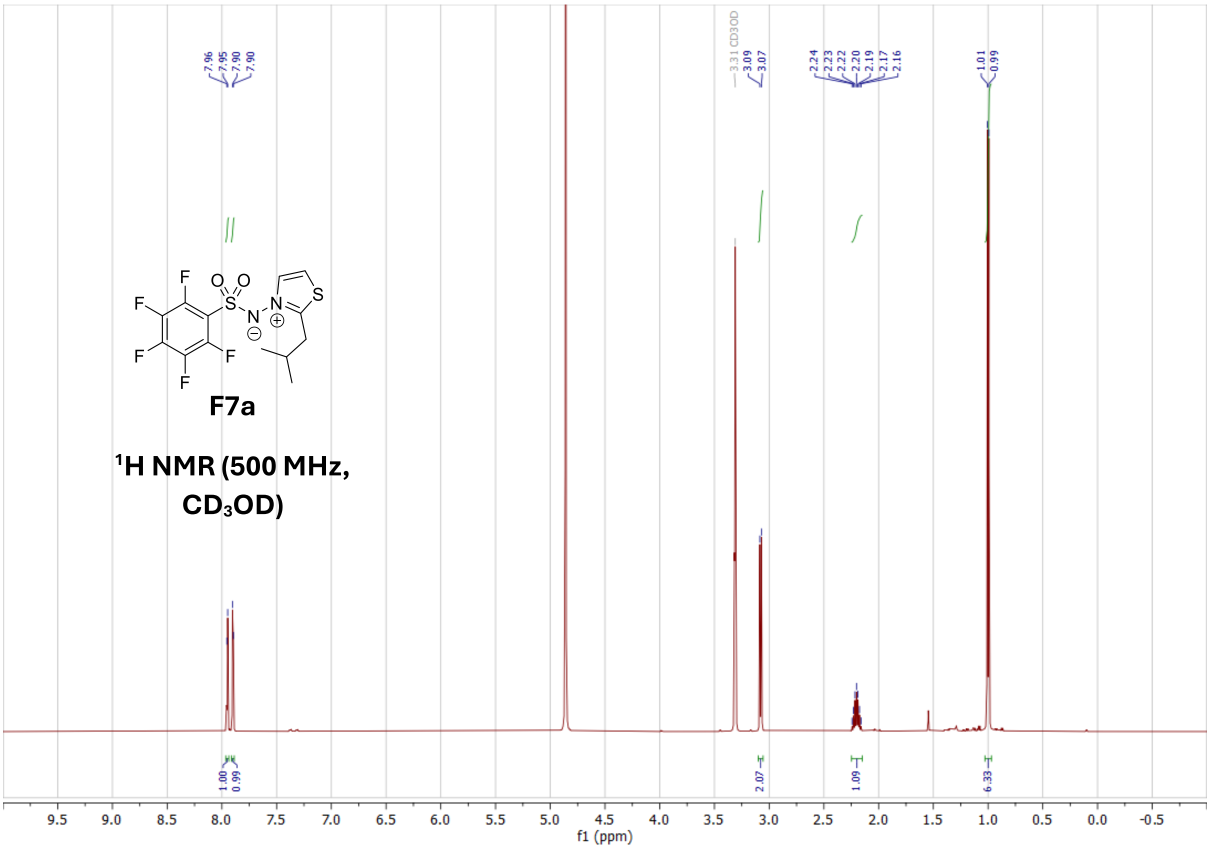
7.96
7.95
7.90
7.90
3.31 CD3OD
3.09
3.07
2.24
2.23
2.22
2.20
2.19
2.17
2.16
1.01
0.99
F7a
¹H NMR (500 MHz, CD₃OD)
1.00
0.99
2.07
1.09
6.33
f1 (ppm)

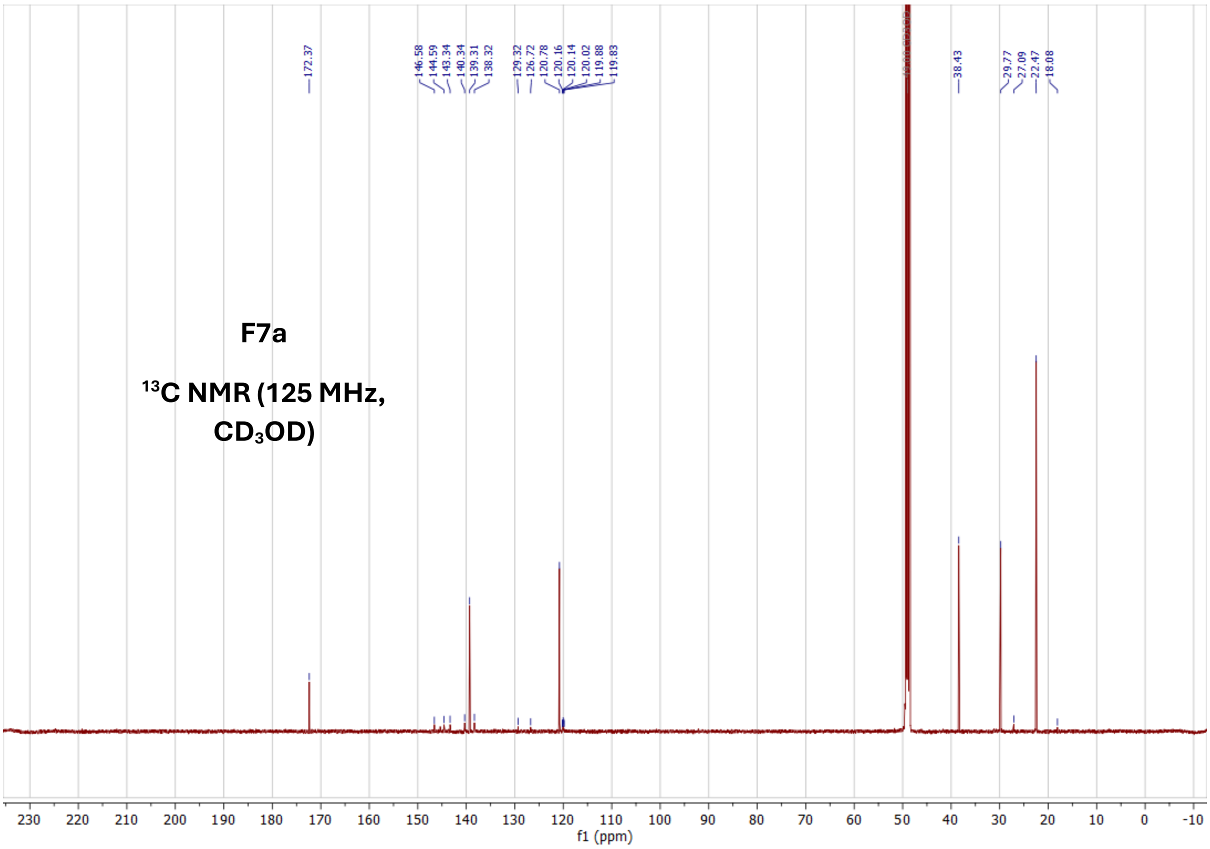
172.37
146.58
144.59
143.34
140.34
139.31
138.32
129.32
126.72
120.78
120.16
120.14
120.02
119.88
119.83
38.43
29.77
27.09
22.47
18.08
F7a
¹³C NMR (125 MHz, CD₃OD)
f1 (ppm)

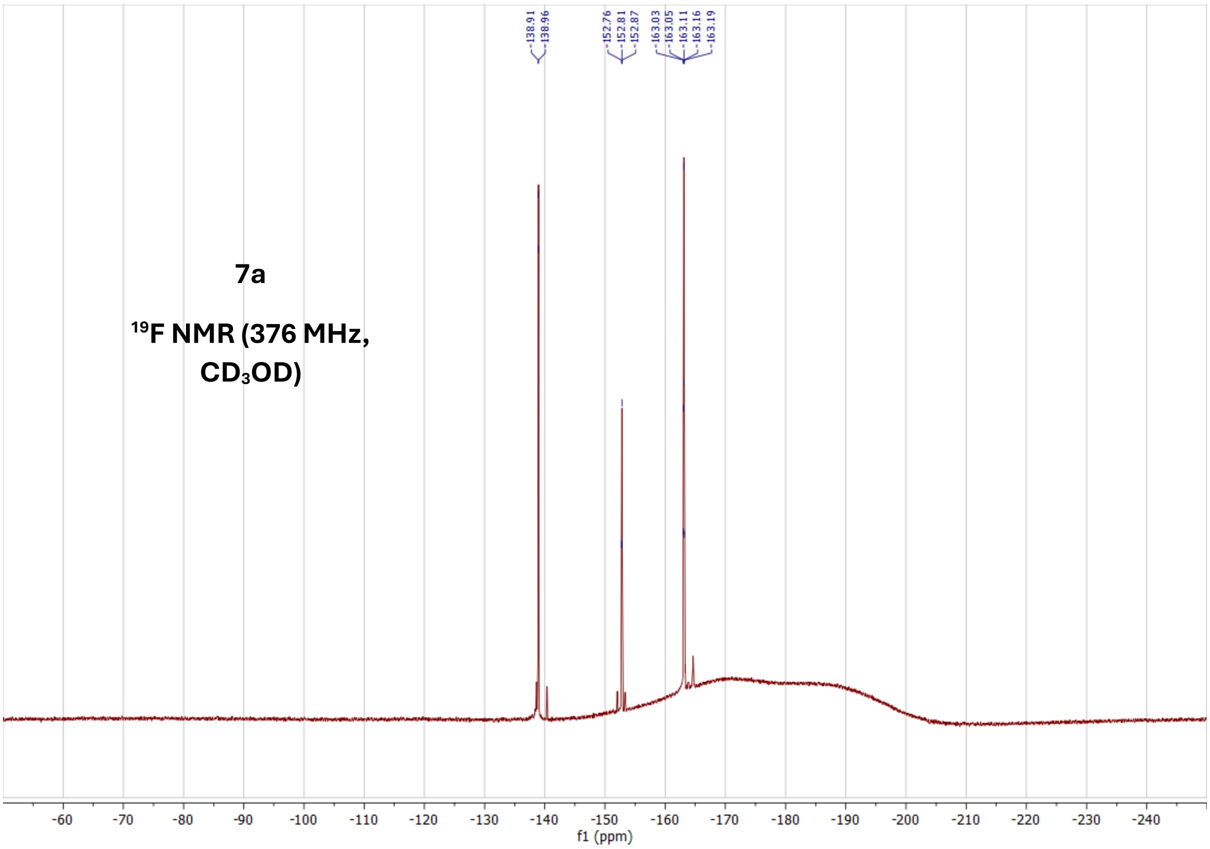

7a

^{19}F NMR (376 MHz, CD_3OD)

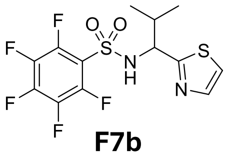

F7b

^{1}H NMR (500 MHz, CD_3OD)

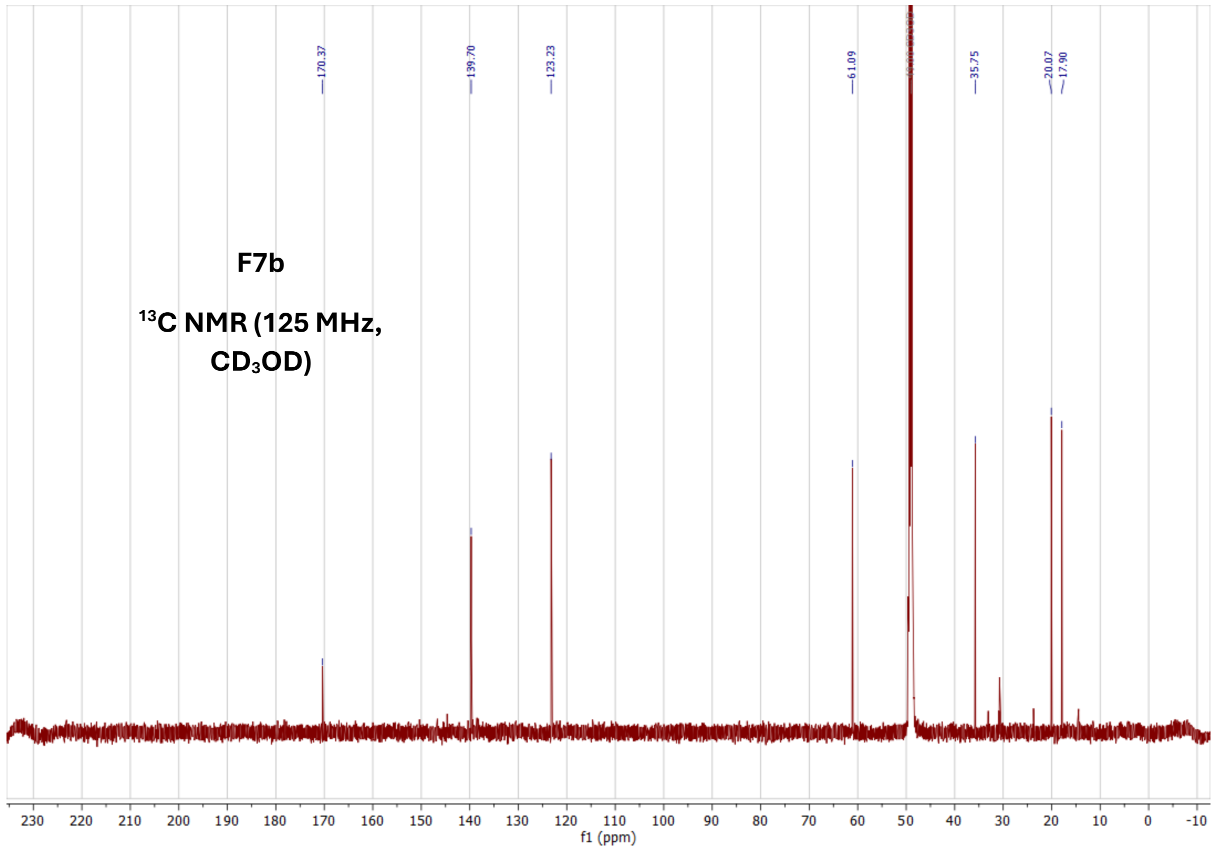

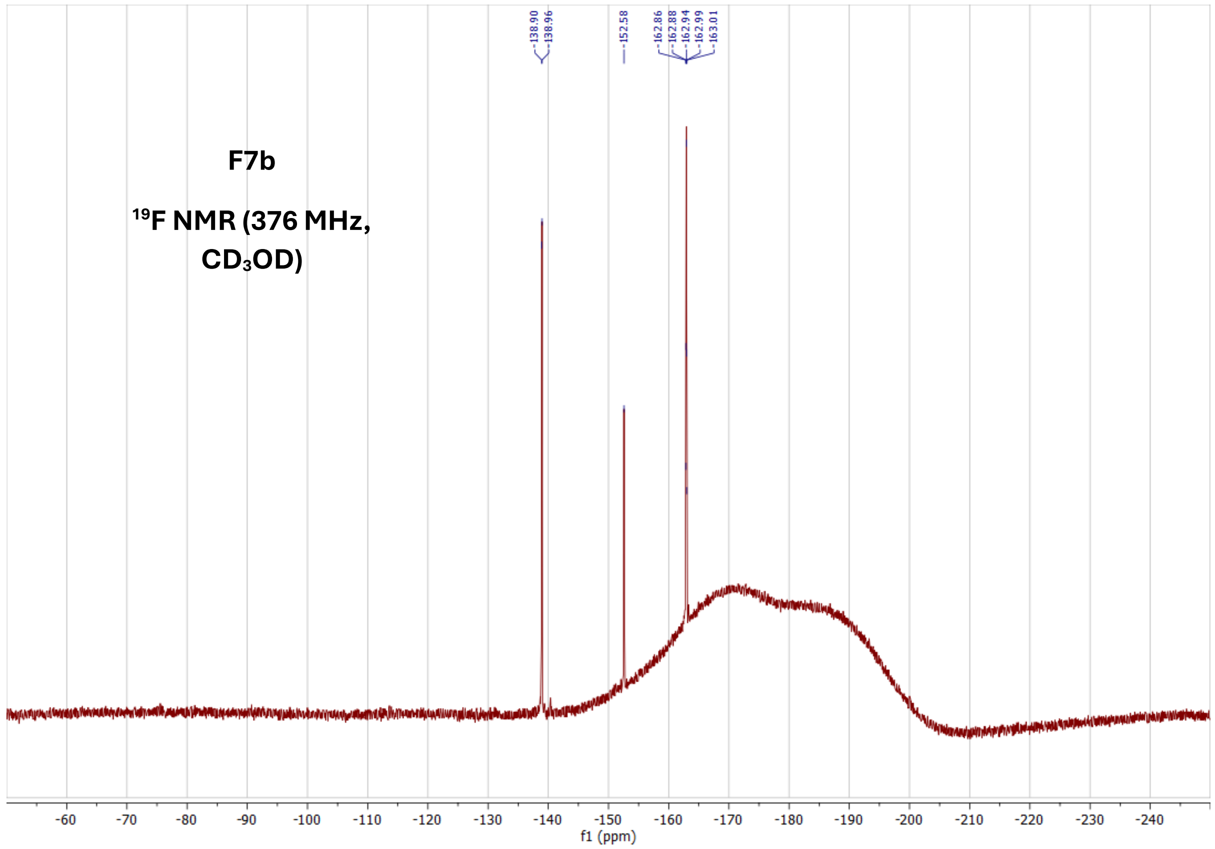
F7b
^{19}F NMR (376 MHz, CD_3OD)
-138.90
-138.96
-152.58
-162.86
-162.88
-162.94
-162.99
-163.01
-60 -70 -80 -90 -100 -110 -120 -130 -140 -150 -160 -170 -180 -190 -200 -210 -220 -230 -240
f1 (ppm)

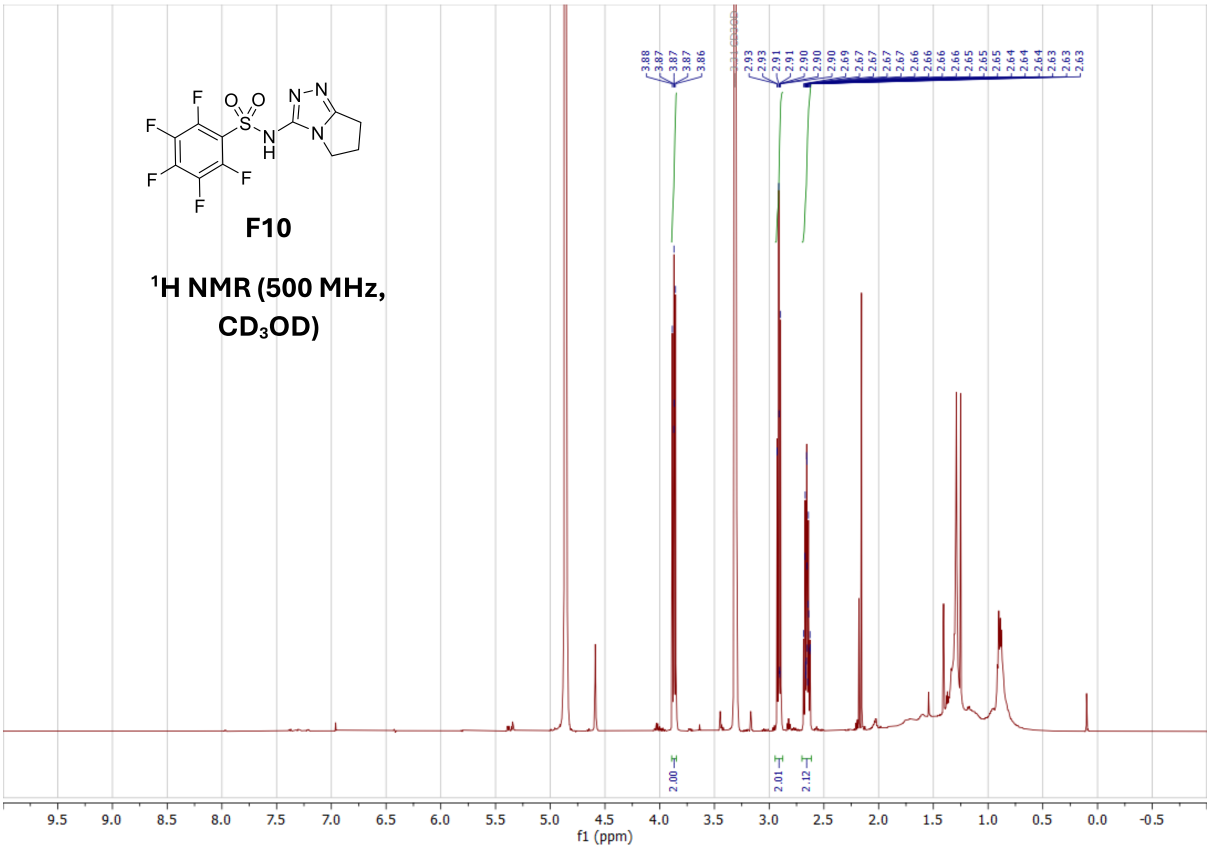
F10
1H NMR (500 MHz, CD3OD)
3.88 3.87 3.87 3.87 3.87 3.86
2.93 2.93 2.91 2.91 2.90 2.90 2.90 2.69 2.67 2.67 2.67 2.67 2.66 2.66 2.66 2.65 2.65 2.65 2.64 2.64 2.64 2.63 2.63 2.63
2.00
2.01
2.12
f1 (ppm)

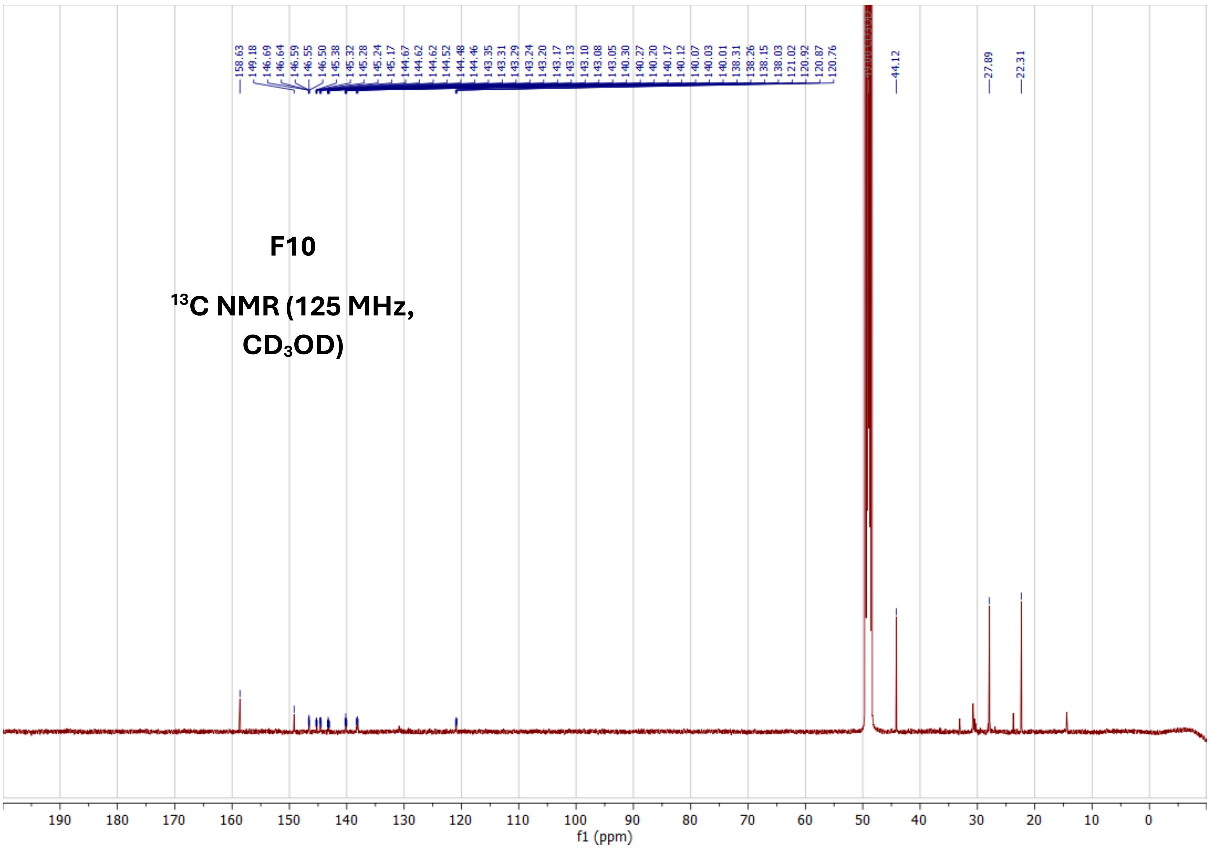
F10
13C NMR (125 MHz, CD3OD)
158.63 149.18 146.69 146.64 146.59 146.55 146.50 145.38 145.32 145.28 145.24 145.17 144.67 144.62 144.62 144.52 144.48 144.46 143.35 143.31 143.29 143.24 143.20 143.17 143.13 143.10 143.08 143.05 140.30 140.27 140.20 140.17 140.12 140.07 140.03 140.01 138.31 138.26 138.15 138.03 121.02 120.92 120.87 120.76
44.12
27.89
22.31
f1 (ppm)

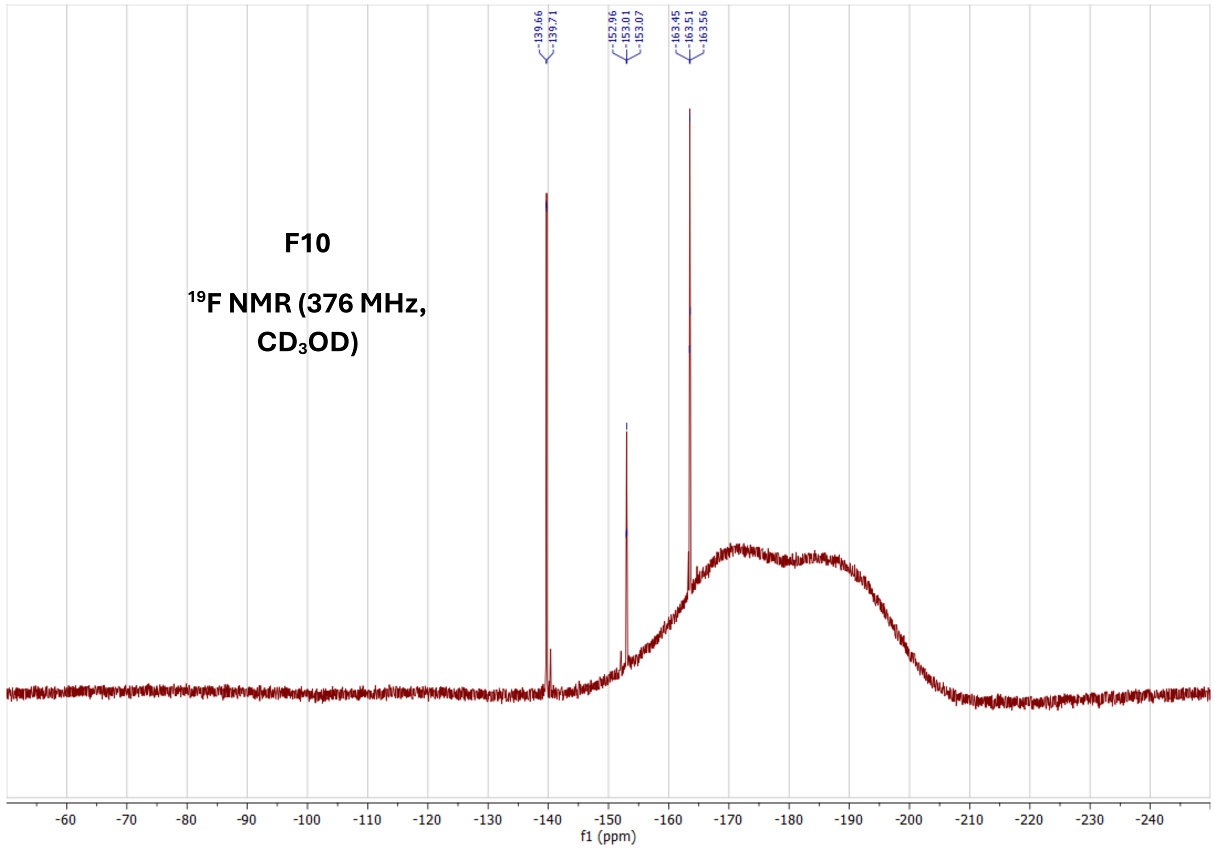
F10
^{19}F NMR (376 MHz, CD_3OD)
-139.66
-139.71
-152.96
-153.01
-153.07
-163.45
-163.51
-163.56
-60 -70 -80 -90 -100 -110 -120 -130 -140 -150 -160 -170 -180 -190 -200 -210 -220 -230 -240
f1 (ppm)

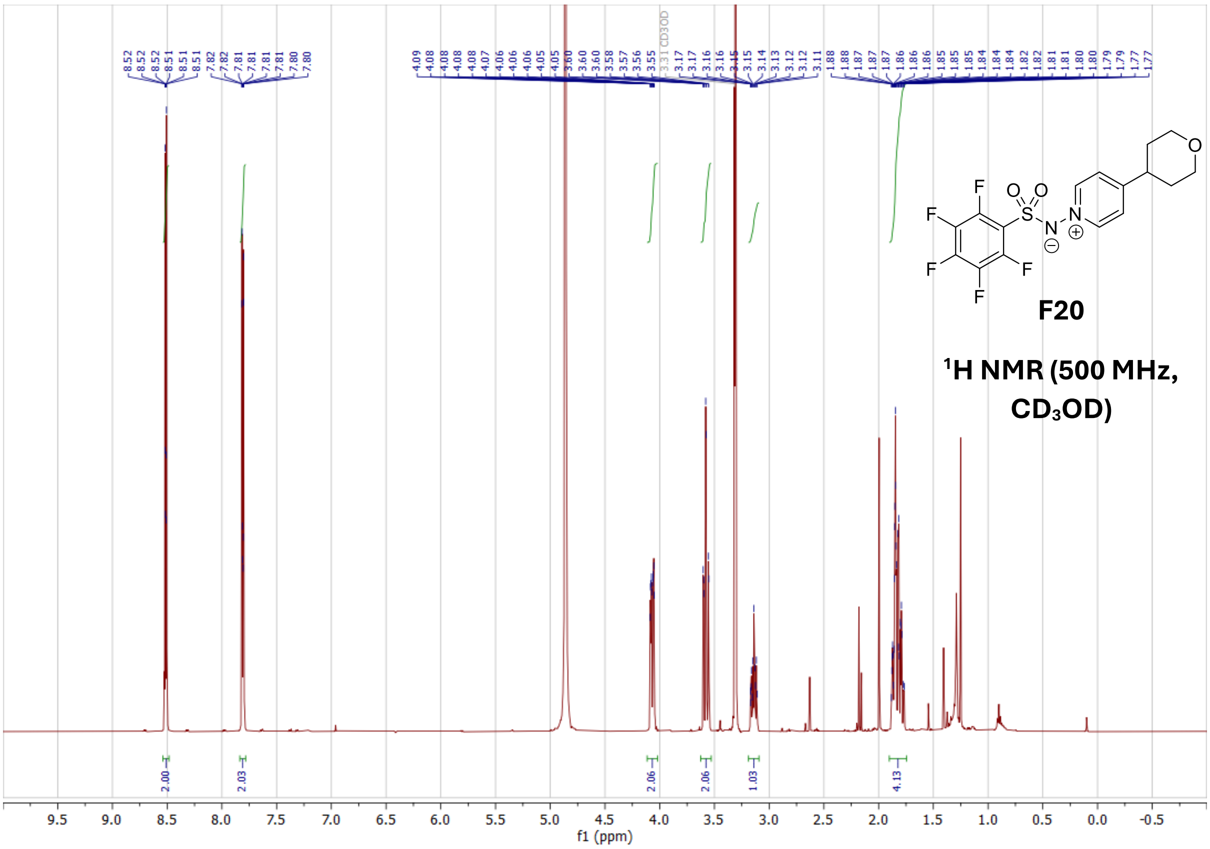

F20

^{1}H NMR (500 MHz, CD_3OD)

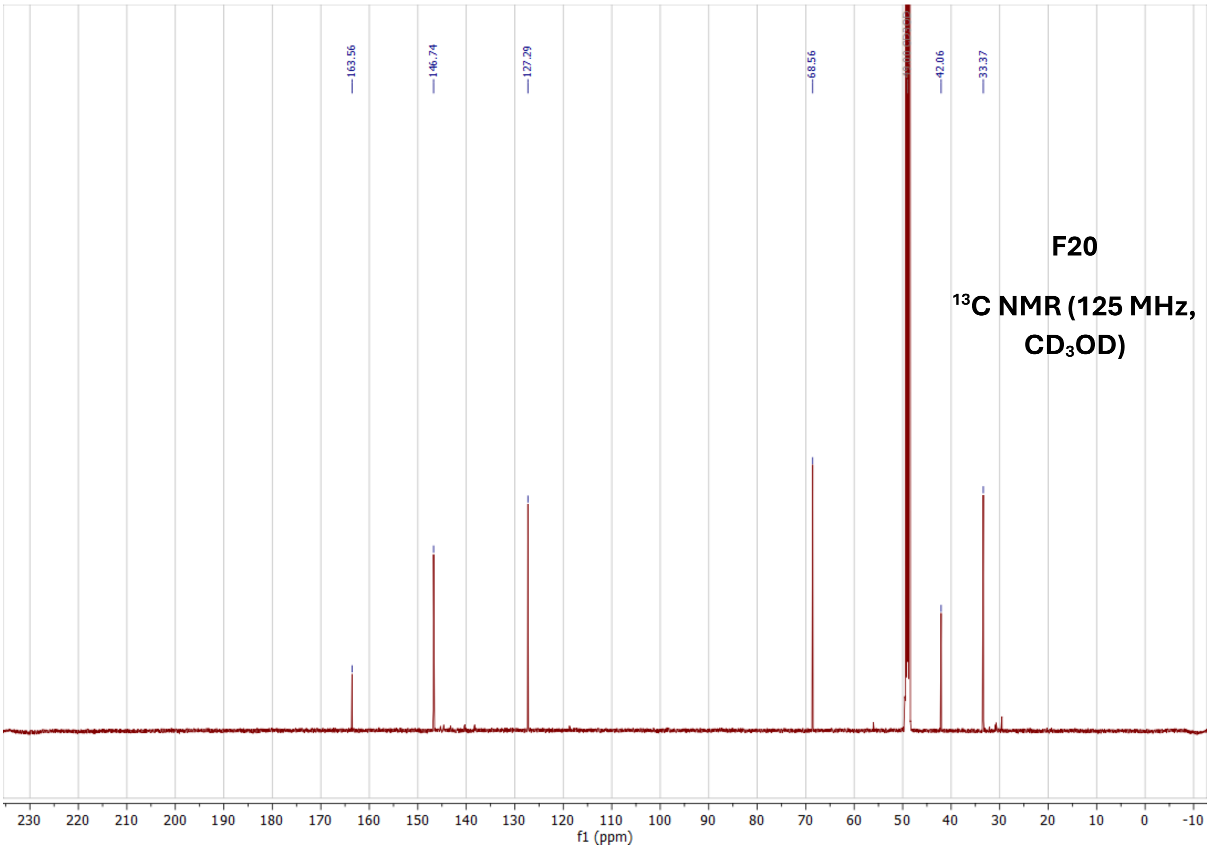

F20

^{13}C NMR (125 MHz, CD_3OD)

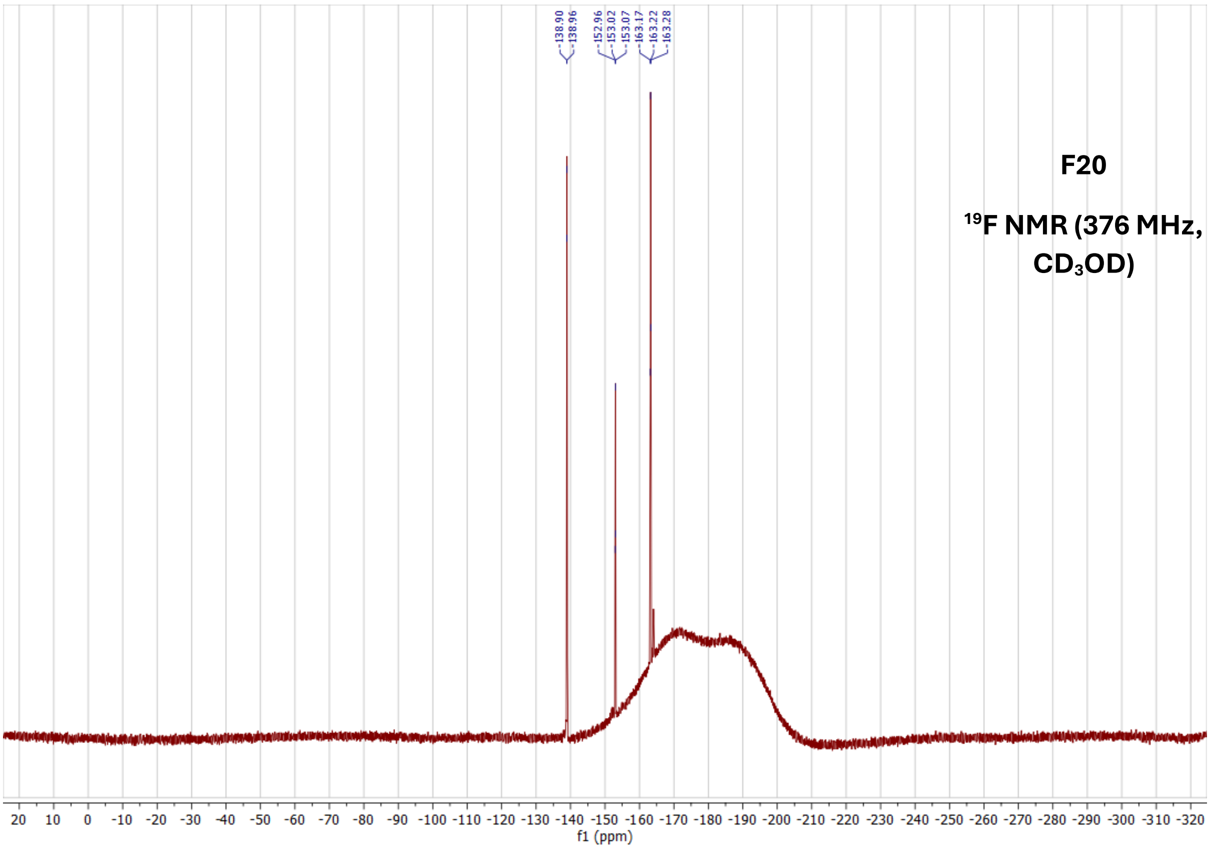
-138.90
-138.96
-152.96
-153.02
-153.07
-163.17
-163.22
-163.28
F20
^{19}F NMR (376 MHz, CD_3OD)
20 10 0 -10 -20 -30 -40 -50 -60 -70 -80 -90 -100 -110 -120 -130 -140 -150 -160 -170 -180 -190 -200 -210 -220 -230 -240 -250 -260 -270 -280 -290 -300 -310 -320
f1 (ppm)

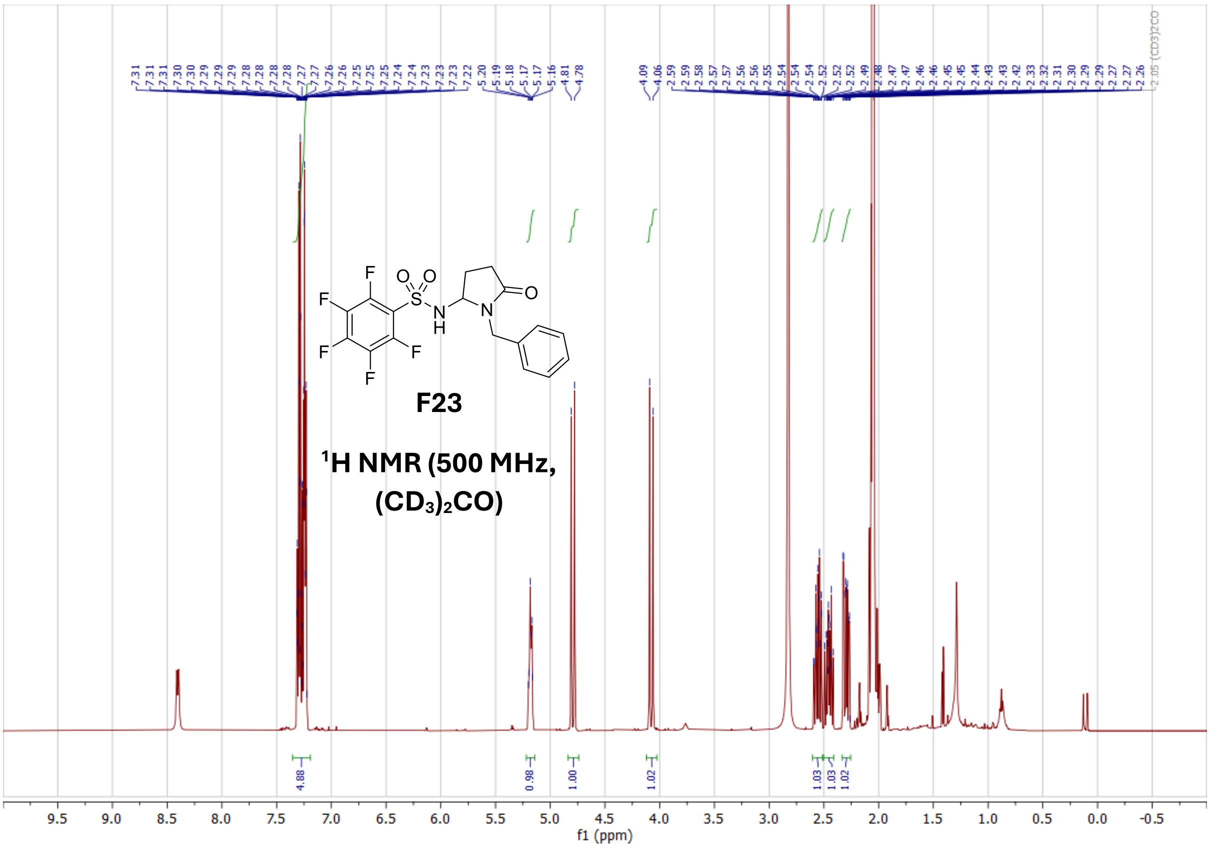
F23
^{1}H NMR (500 MHz, $(CD_3)_2CO$)
f1 (ppm)

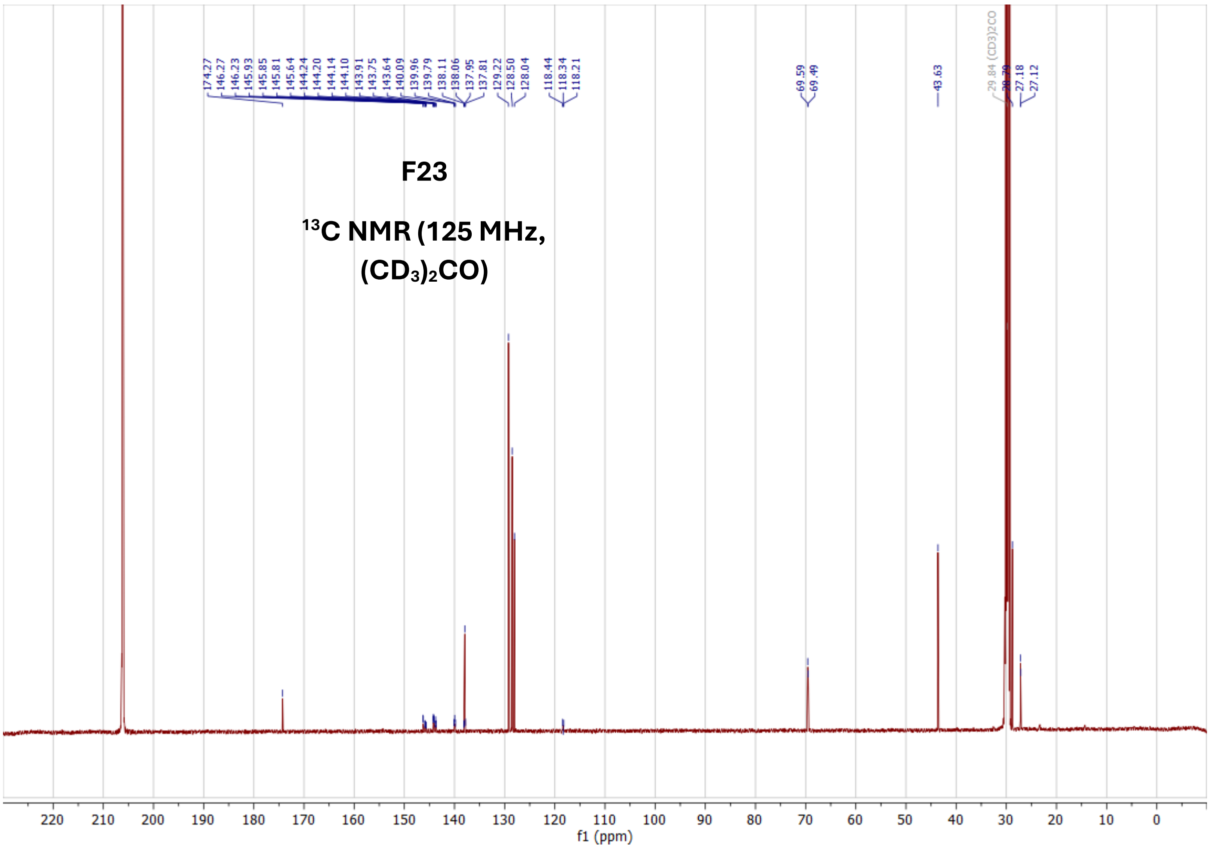
F23
^{13}C NMR (125 MHz, $(CD_3)_2CO$)
f1 (ppm)

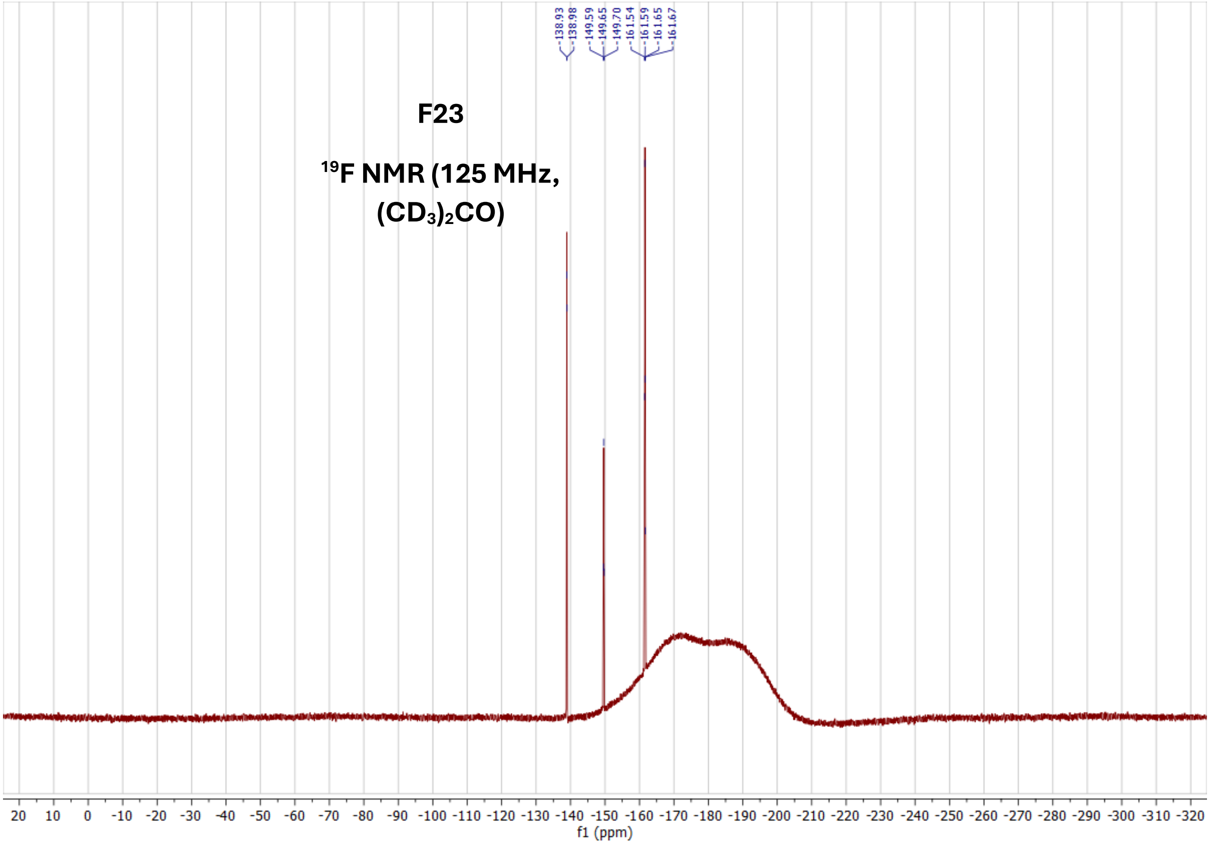
F23
^{19}F NMR (125 MHz, $(CD_3)_2CO$)
-138.93
-138.98
-149.59
-149.65
-149.70
-161.54
-161.59
-161.65
-161.67
20 10 0 -10 -20 -30 -40 -50 -60 -70 -80 -90 -100 -110 -120 -130 -140 -150 -160 -170 -180 -190 -200 -210 -220 -230 -240 -250 -260 -270 -280 -290 -300 -310 -320
f1 (ppm)

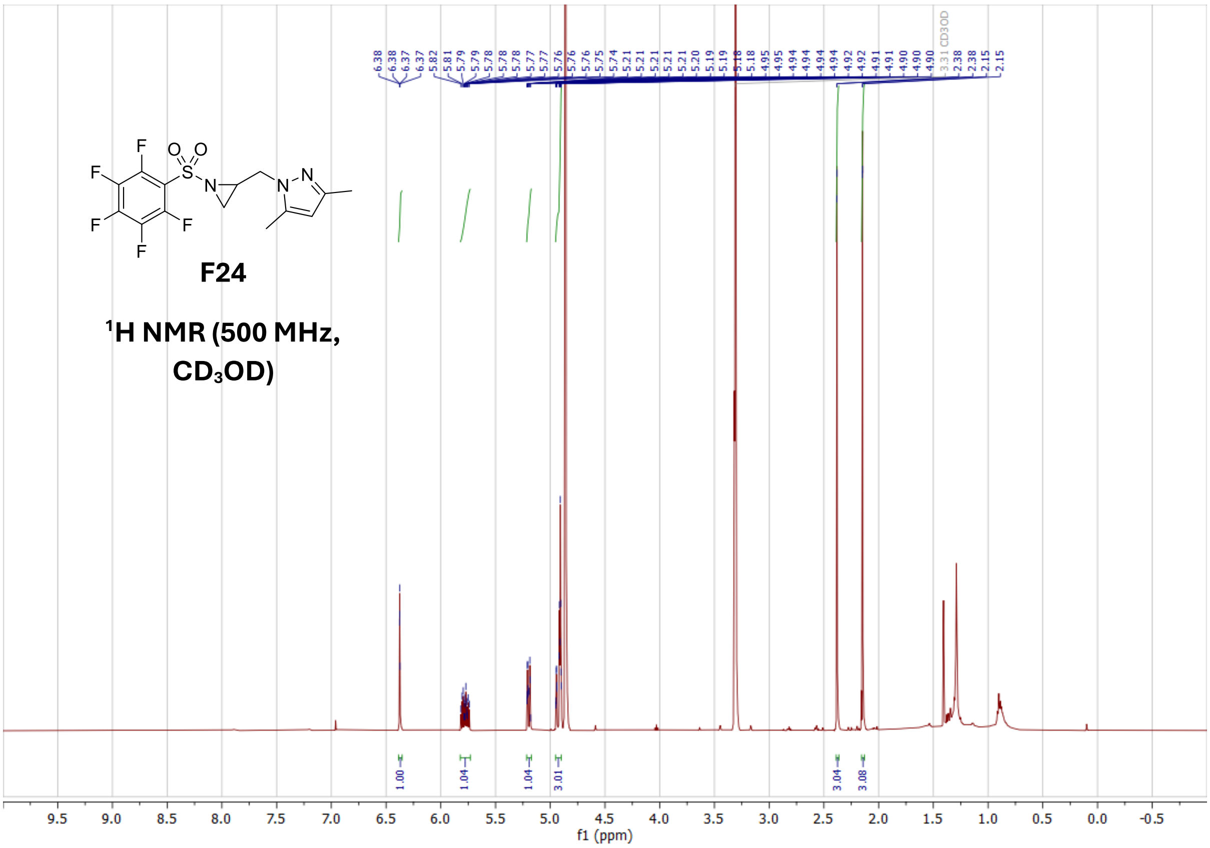
F24
1H NMR (500 MHz, CD3OD)
6.38 6.38 6.37 6.37 5.82 5.81 5.79 5.79 5.78 5.78 5.78 5.77 5.77 5.76 5.76 5.75 5.74 5.21 5.21 5.21 5.21 5.20 5.19 5.19 5.18 5.18 4.95 4.95 4.94 4.94 4.94 4.92 4.92 4.91 4.91 4.90 4.90 3.31 CD3OD 2.38 2.38 2.15 2.15
1.00 1.04 1.04 3.01 3.04 3.08
f1 (ppm)

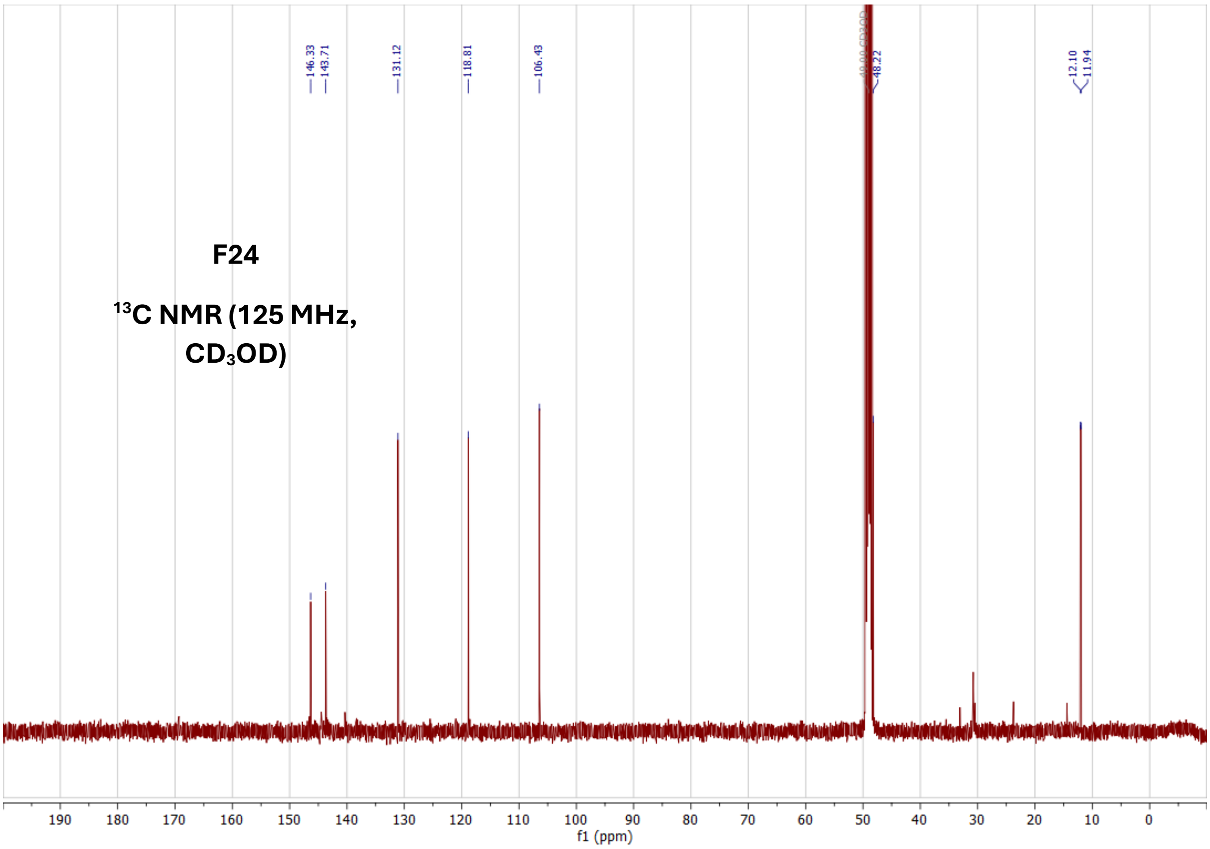
F24
13C NMR (125 MHz, CD3OD)
146.33 143.71 131.12 118.81 106.43 49.00 CD3OD 48.22 12.10 11.94
f1 (ppm)

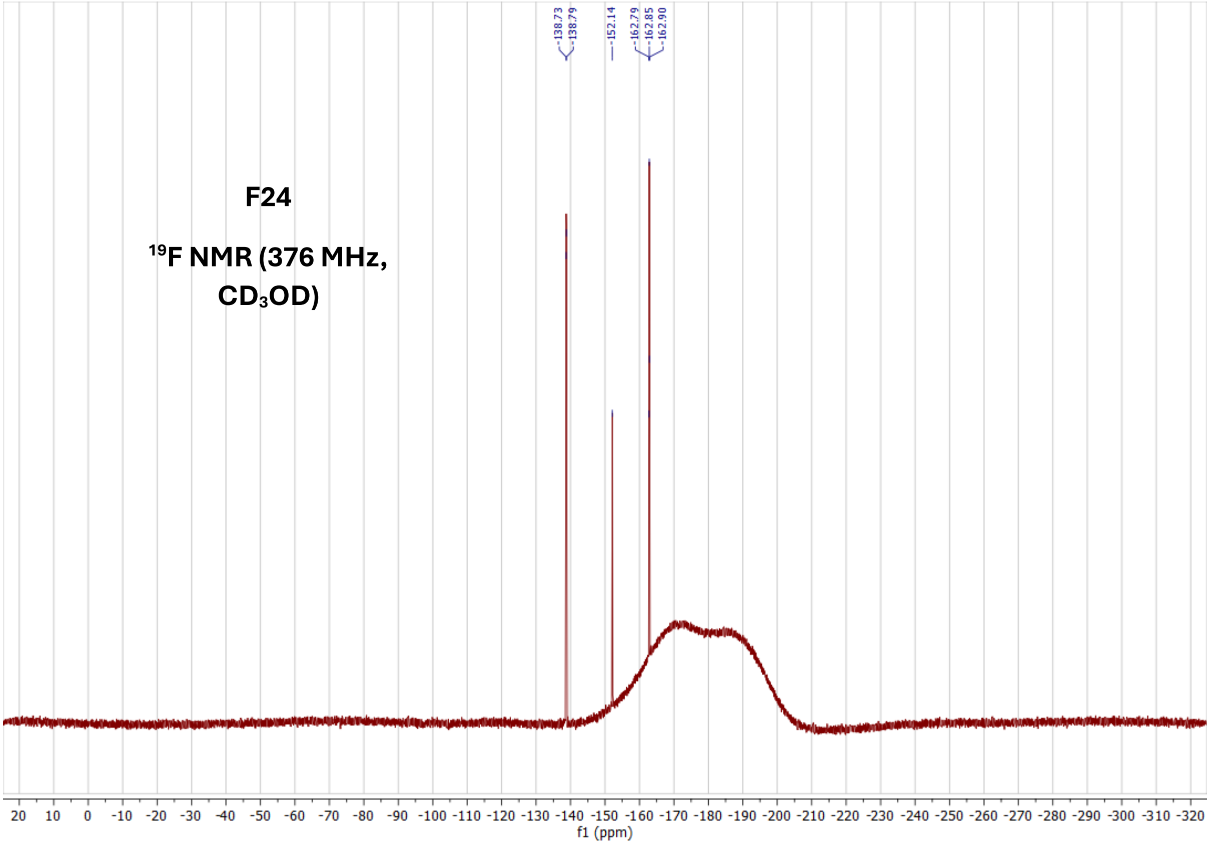
-138.73
-138.79
-152.14
-162.79
-162.85
-162.90
F24
^{19}F NMR (376 MHz, CD_3OD)
20 10 0 -10 -20 -30 -40 -50 -60 -70 -80 -90 -100 -110 -120 -130 -140 -150 -160 -170 -180 -190 -200 -210 -220 -230 -240 -250 -260 -270 -280 -290 -300 -310 -320
f1 (ppm)

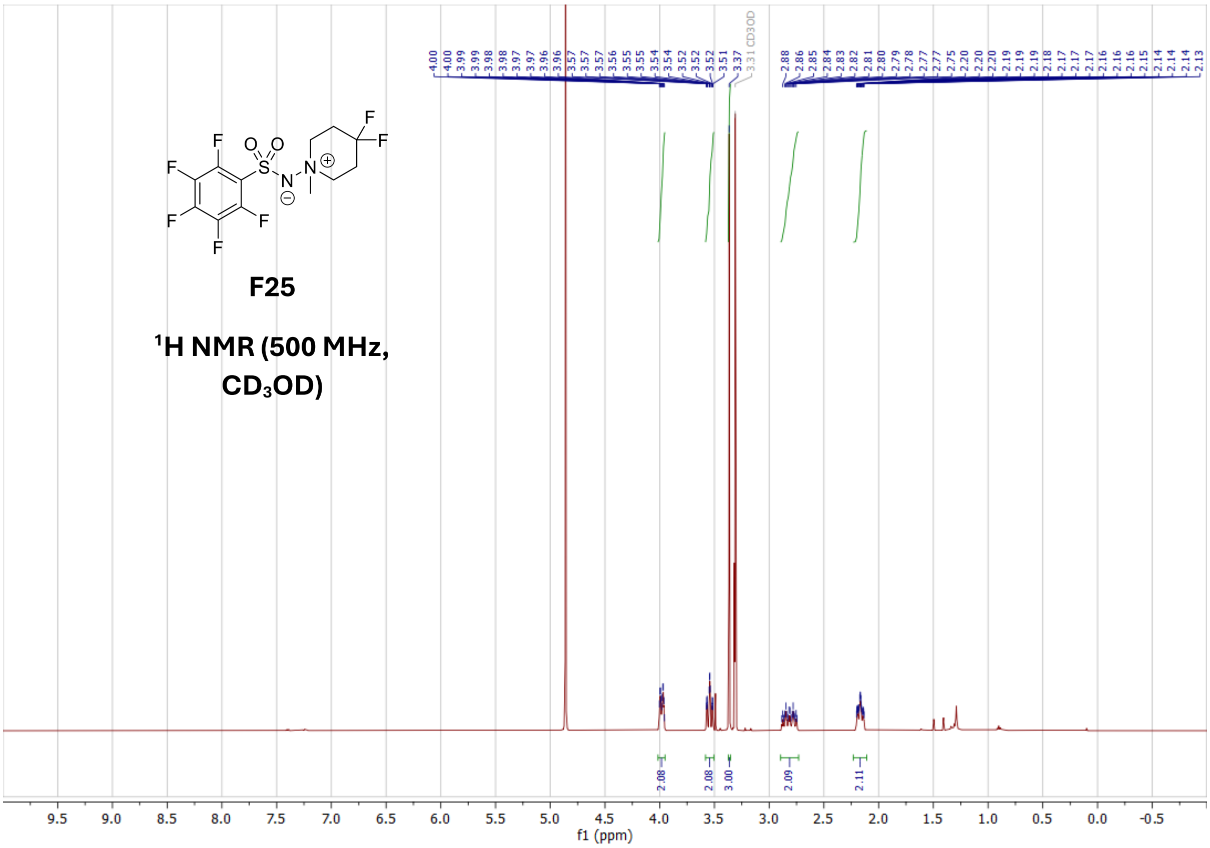
F25
^{1}H NMR (500 MHz, CD_3OD)
f1 (ppm)

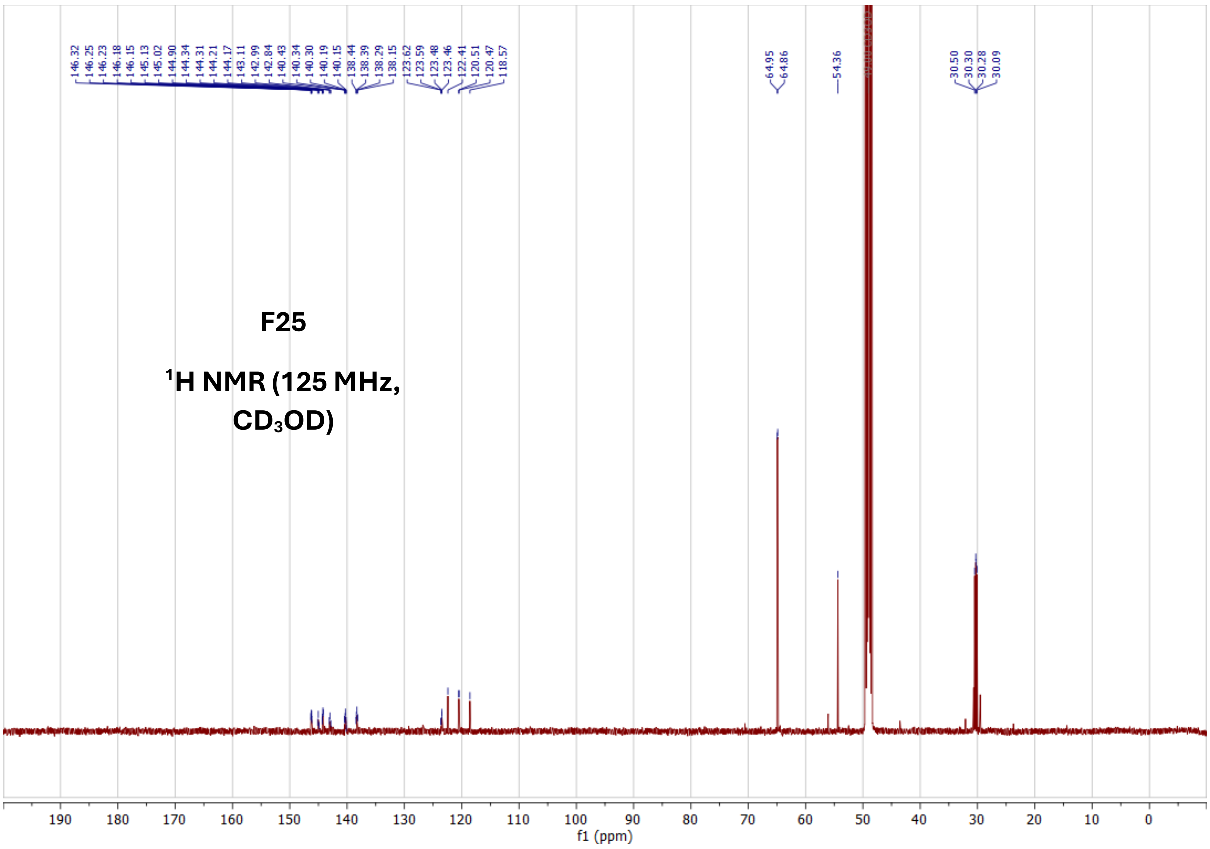
F25
^{1}H NMR (125 MHz, CD_3OD)
f1 (ppm)

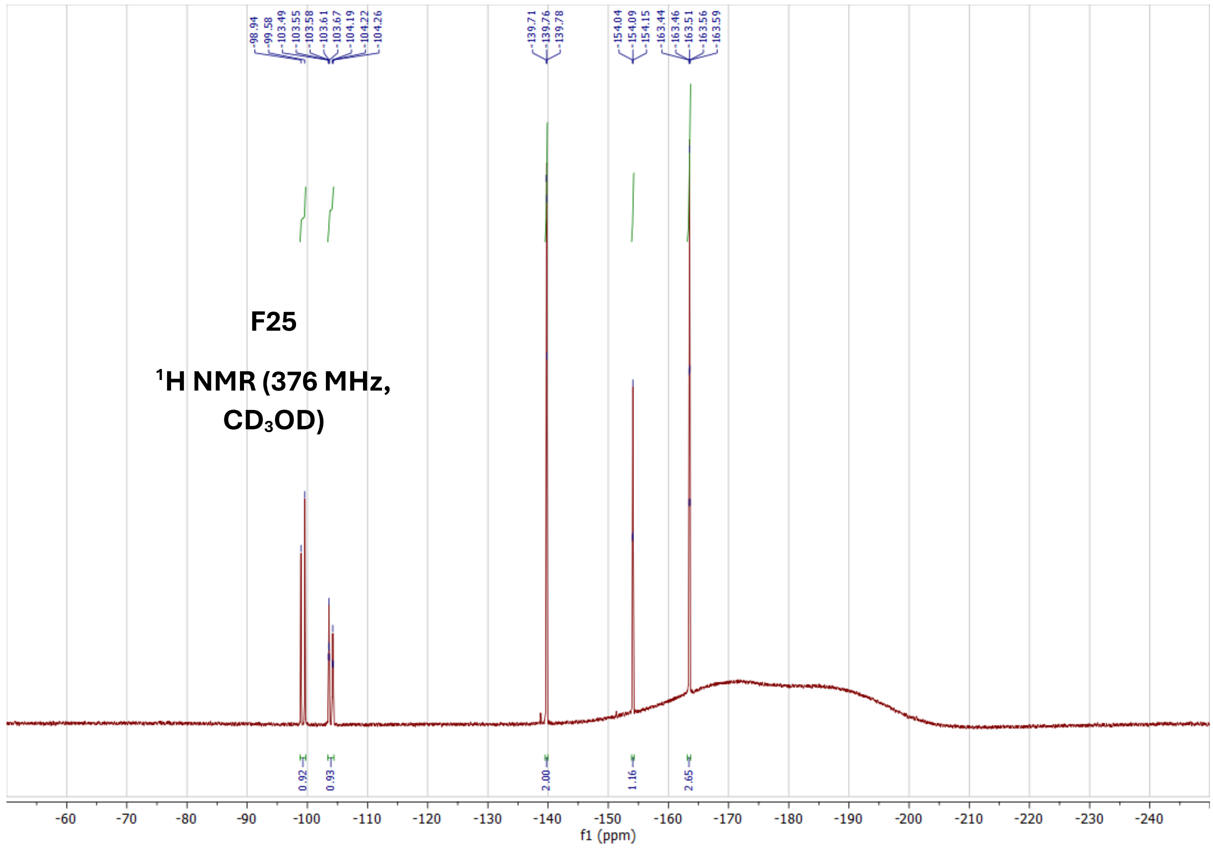
F25
^{1}H NMR (376 MHz, CD_3OD)
f1 (ppm)

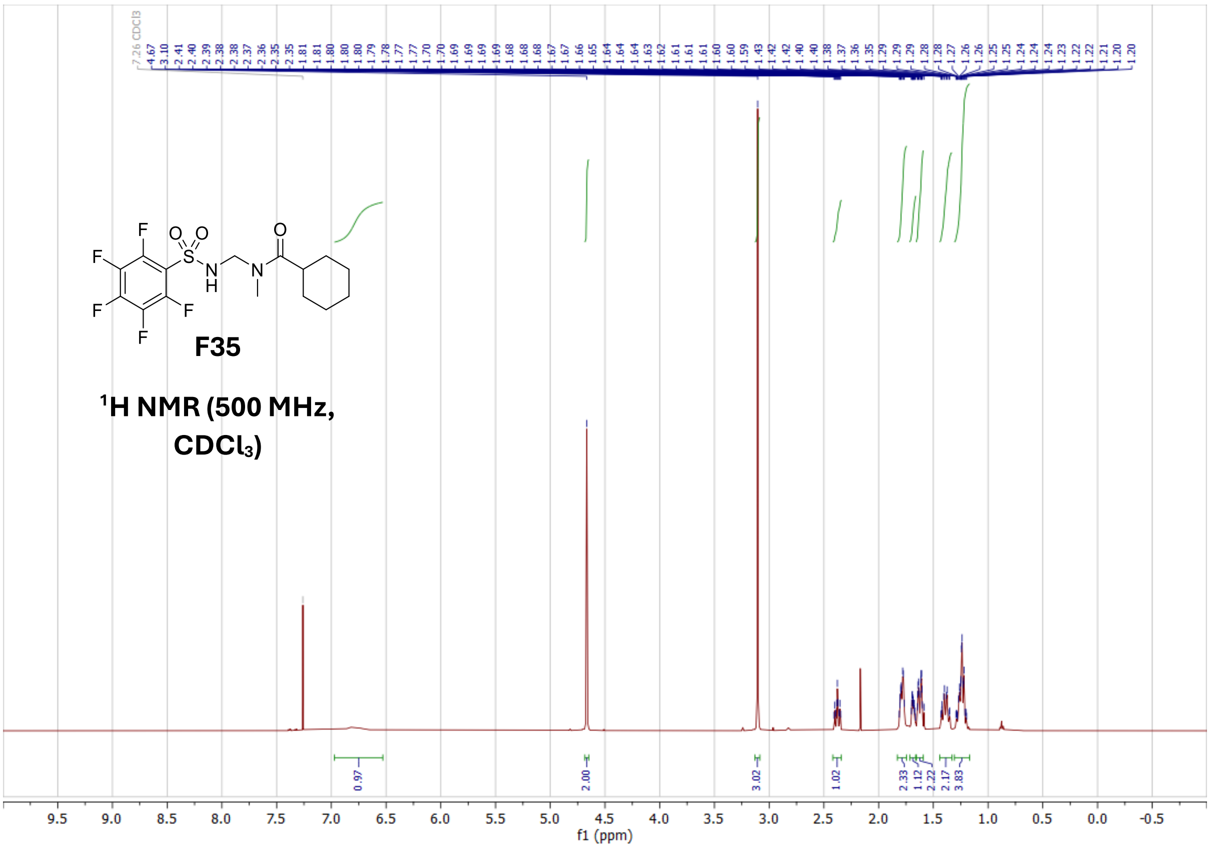
F35
^{1}H NMR (500 MHz, CDCl$_3$)
f1 (ppm)

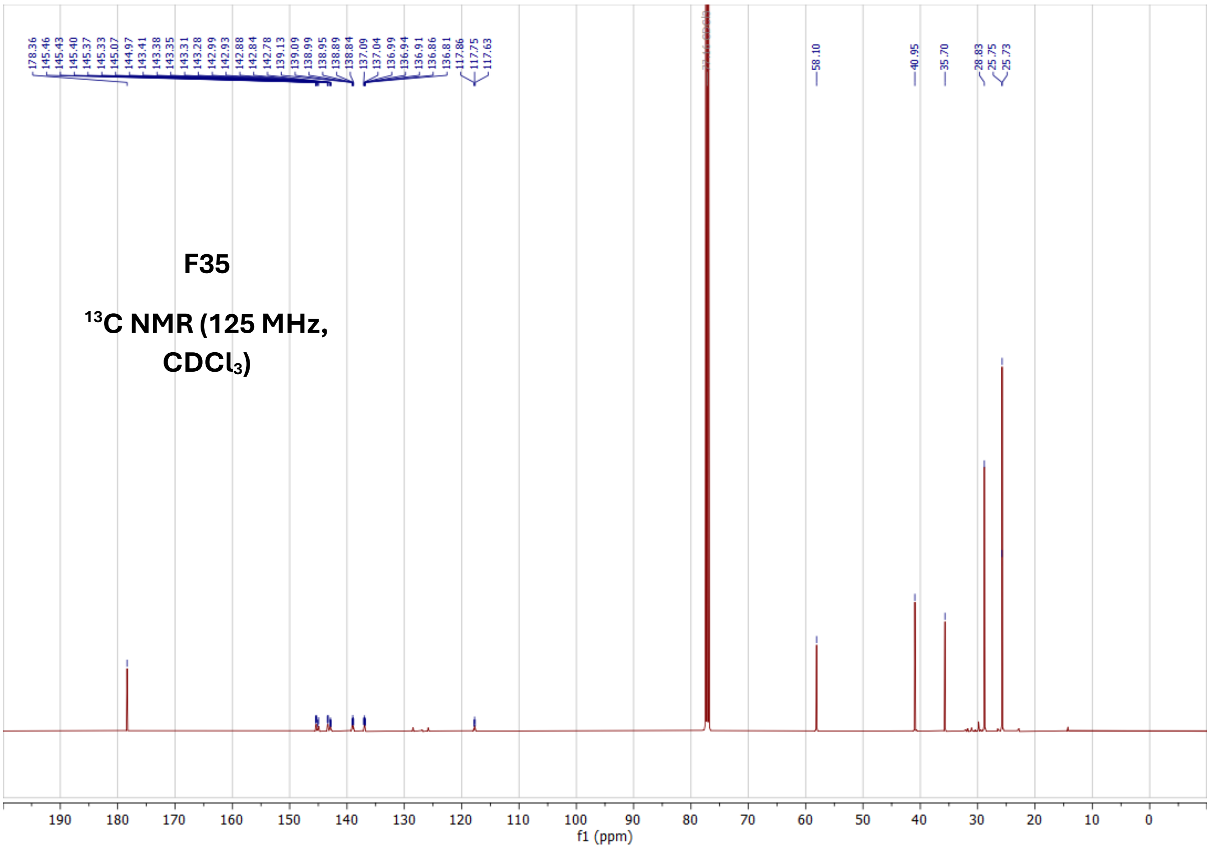
F35
^{13}C NMR (125 MHz, CDCl$_3$)
f1 (ppm)

F35

^{19}F NMR (376 MHz, $CDCl_3$)

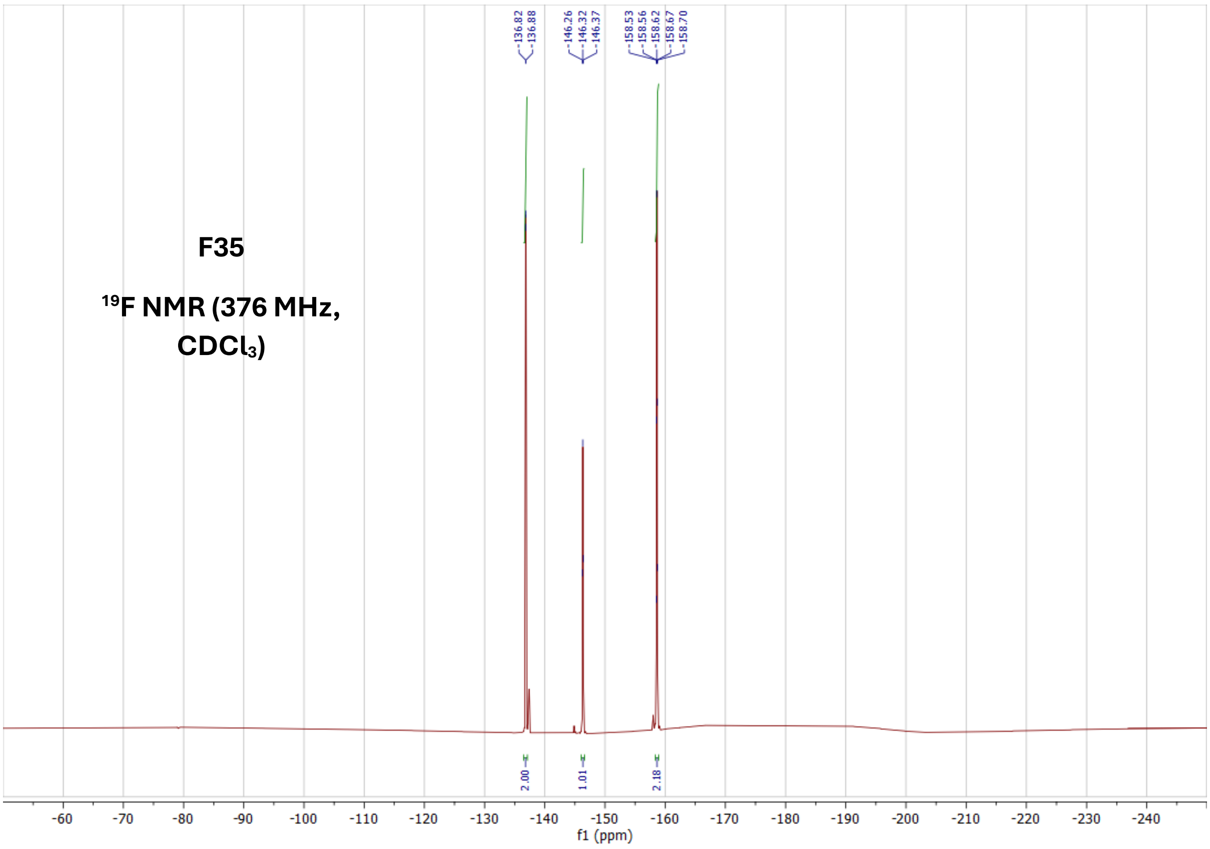

F37

^{1}H NMR (500 MHz, CD$_3$OD)

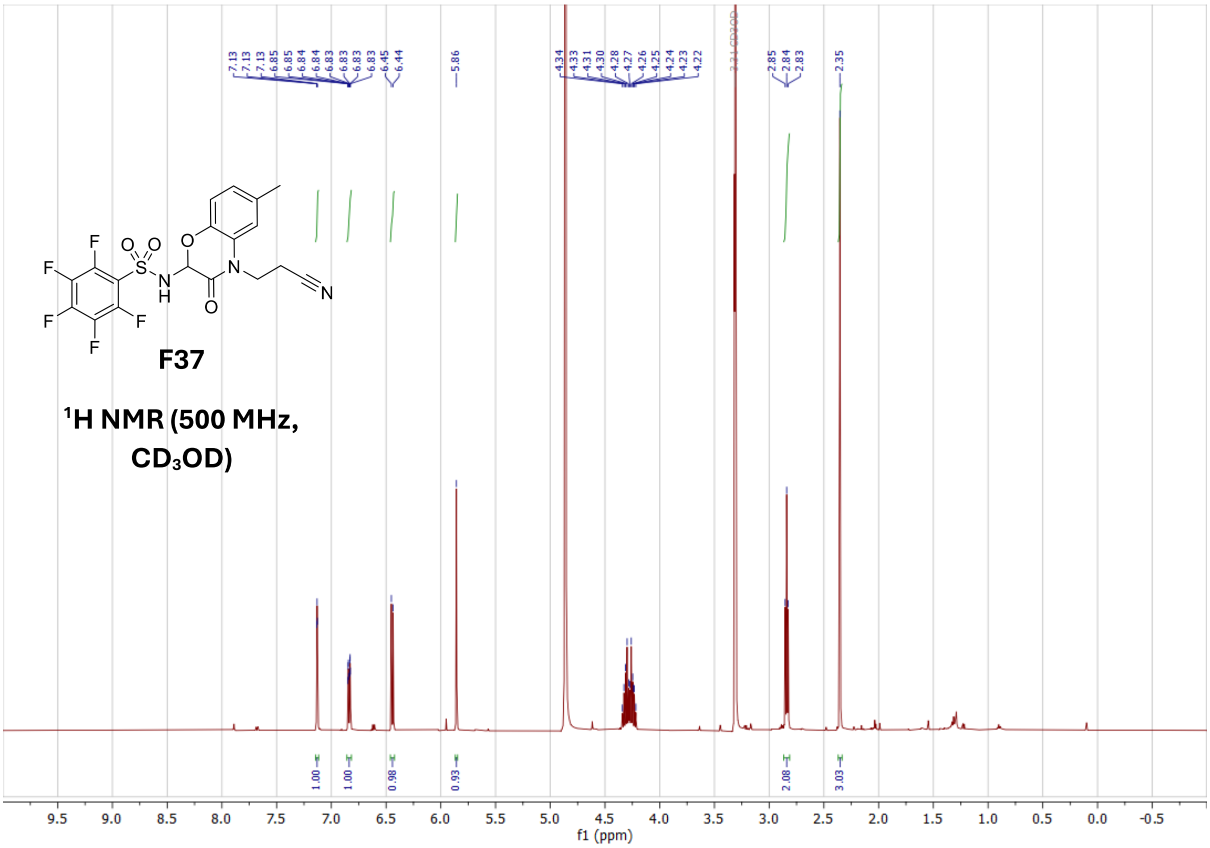

F37

^{13}C NMR (125 MHz, CD$_3$OD)

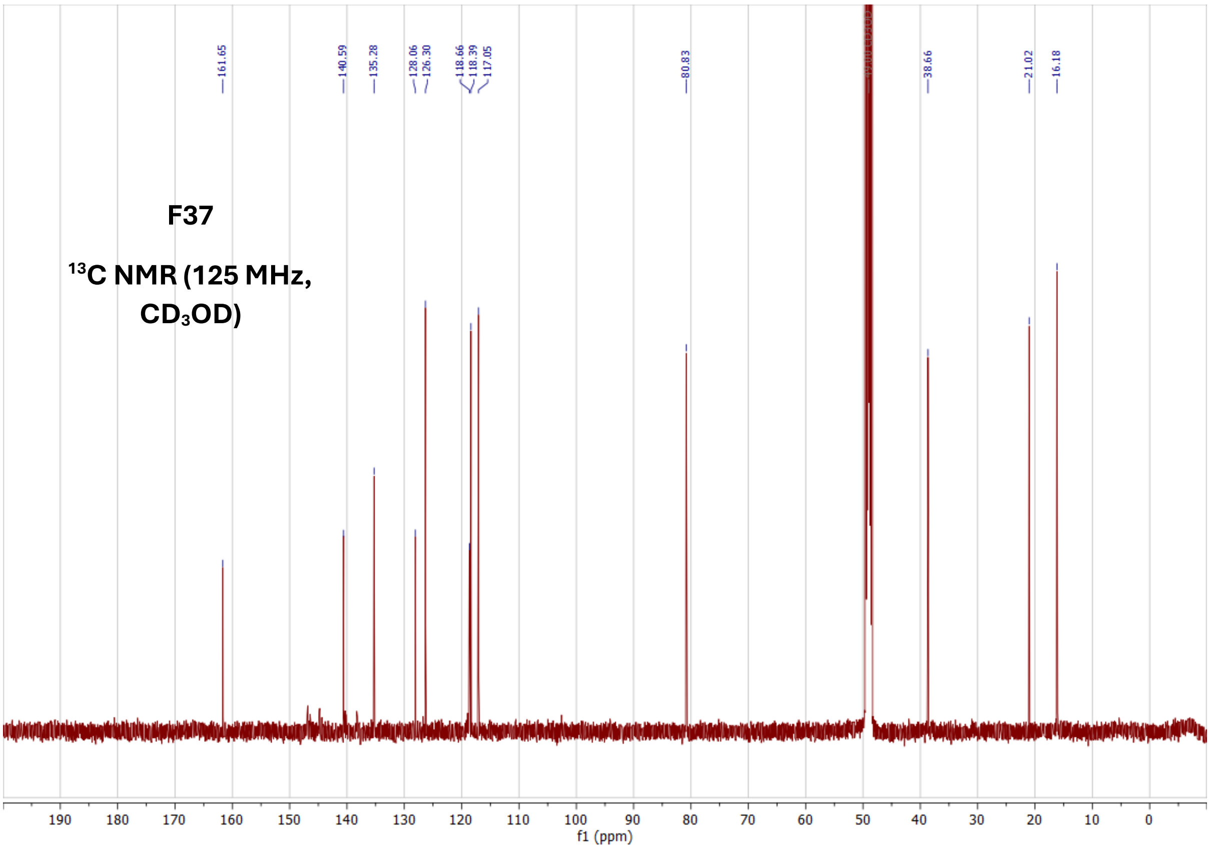

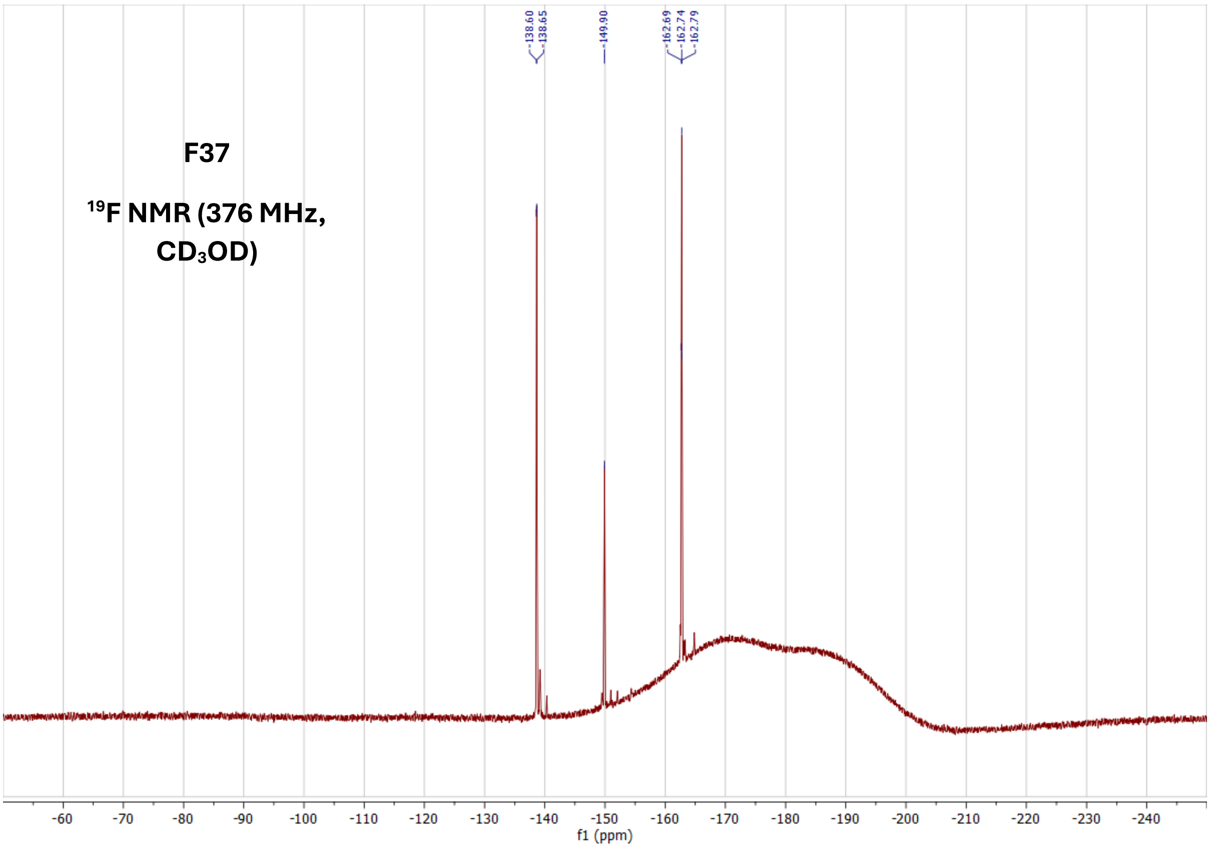
-138.60
-138.65
-149.90
-162.69
-162.74
-162.79
F37
19F NMR (376 MHz, CD3OD)
-60
-70
-80
-90
-100
-110
-120
-130
-140
-150
-160
-170
-180
-190
-200
-210
-220
-230
-240
f1 (ppm)

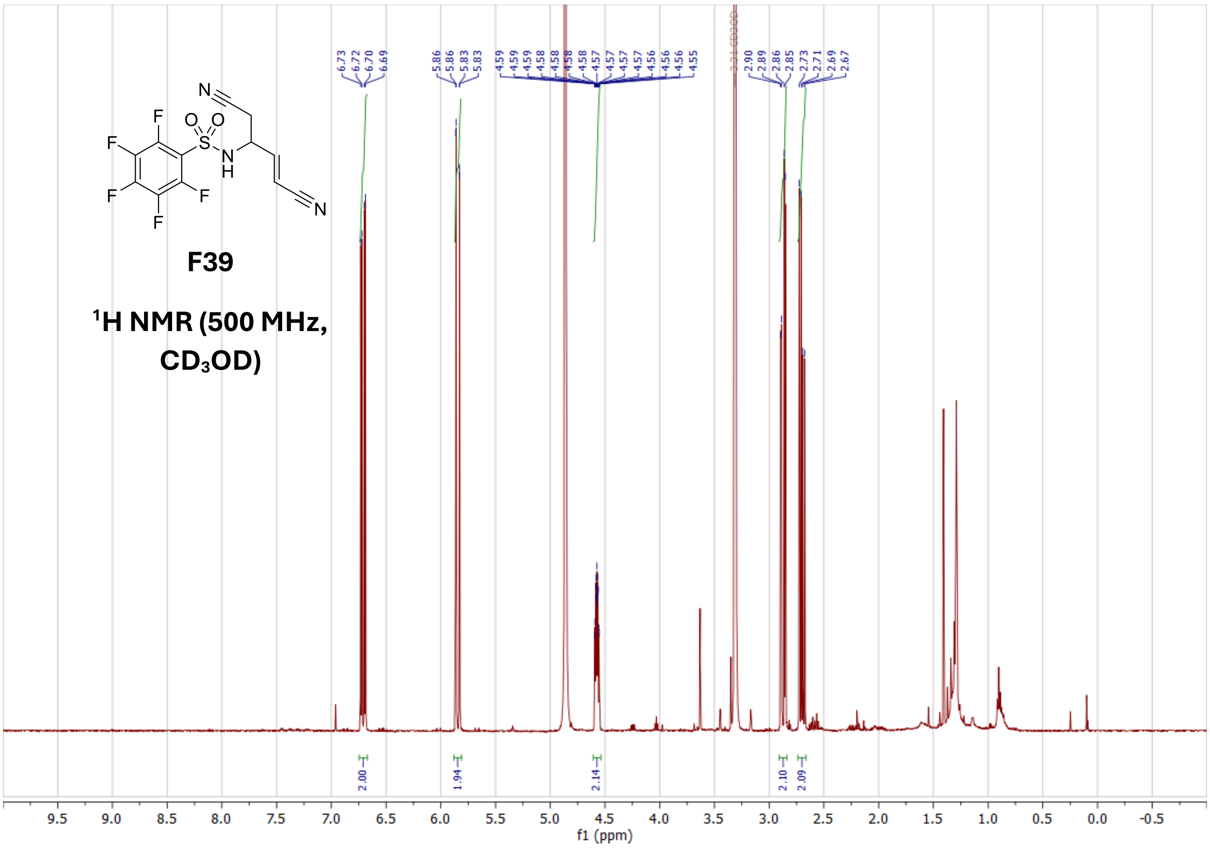
F39
1H NMR (500 MHz, CD3OD)
f1 (ppm)

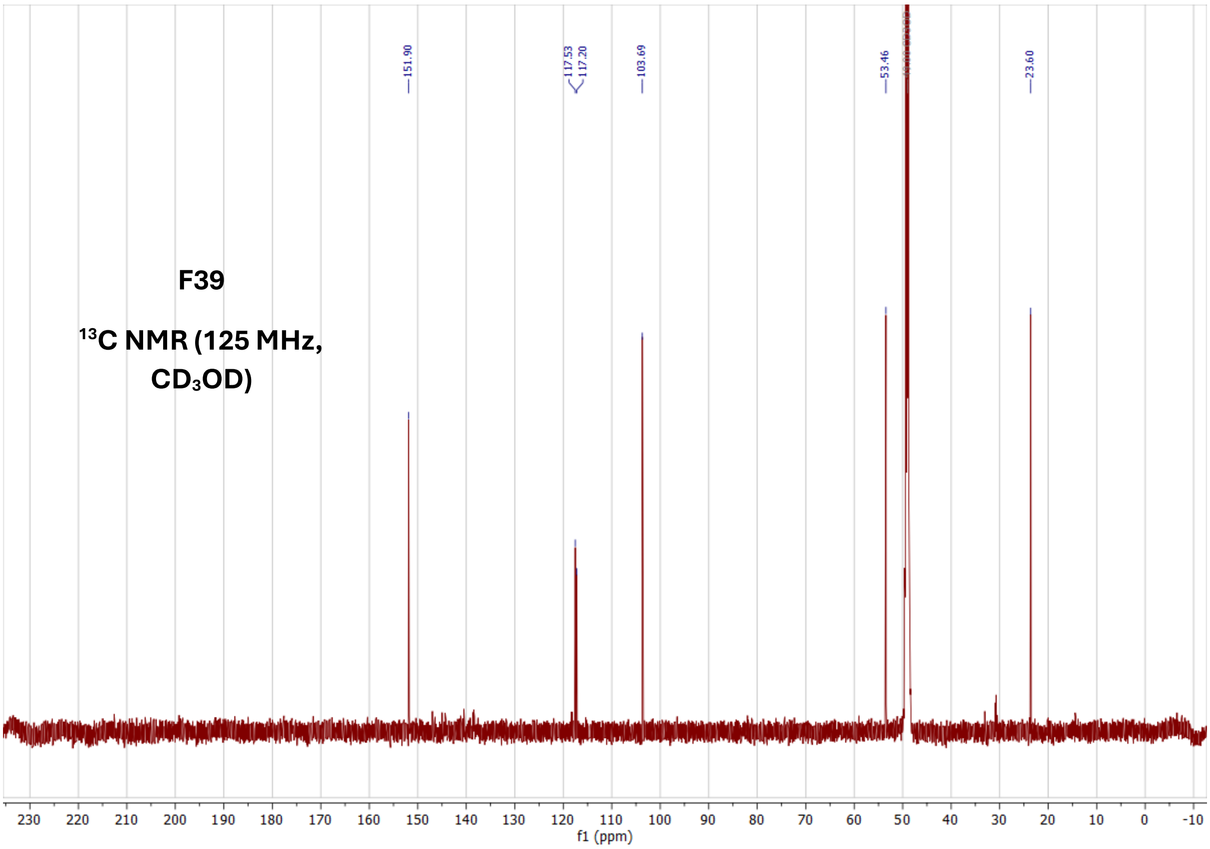
F39
13C NMR (125 MHz, CD3OD)
f1 (ppm)

F39

^{19}F NMR (376 MHz, CD_3OD)

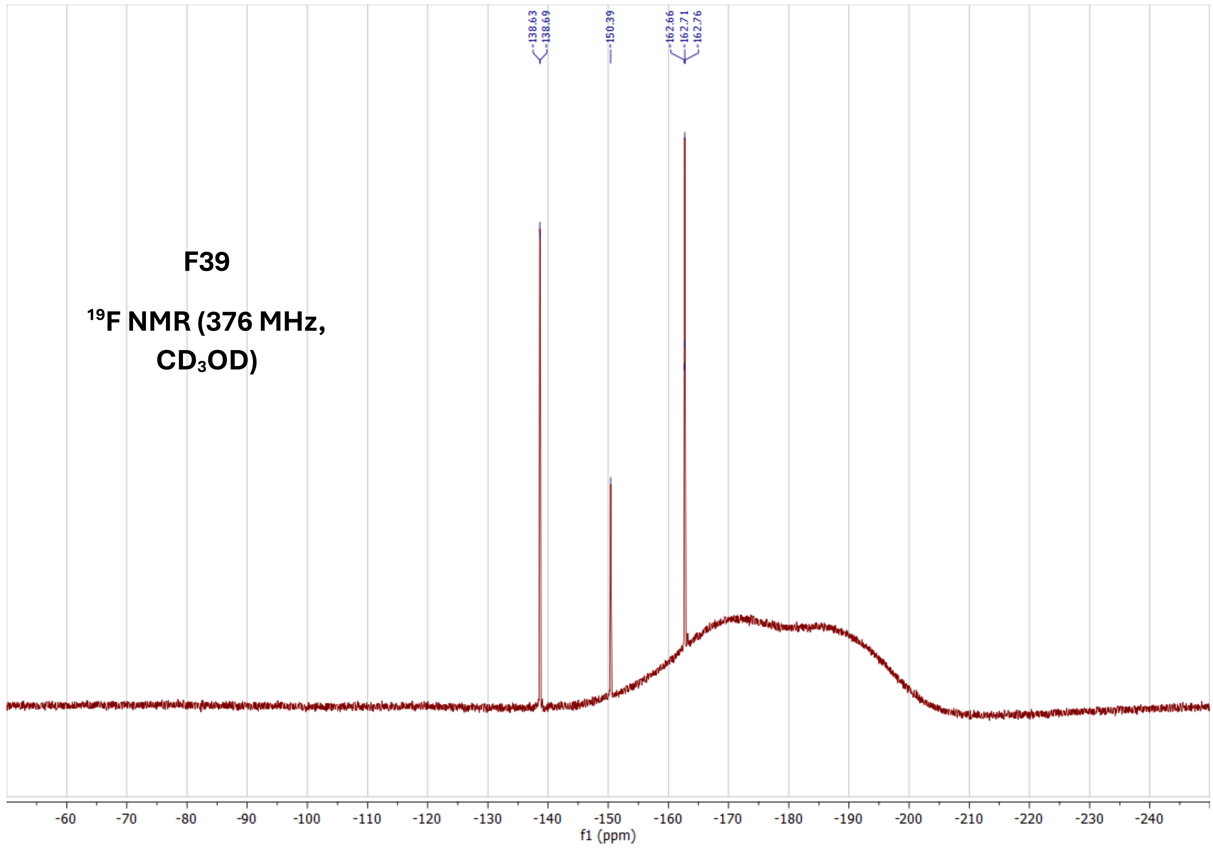

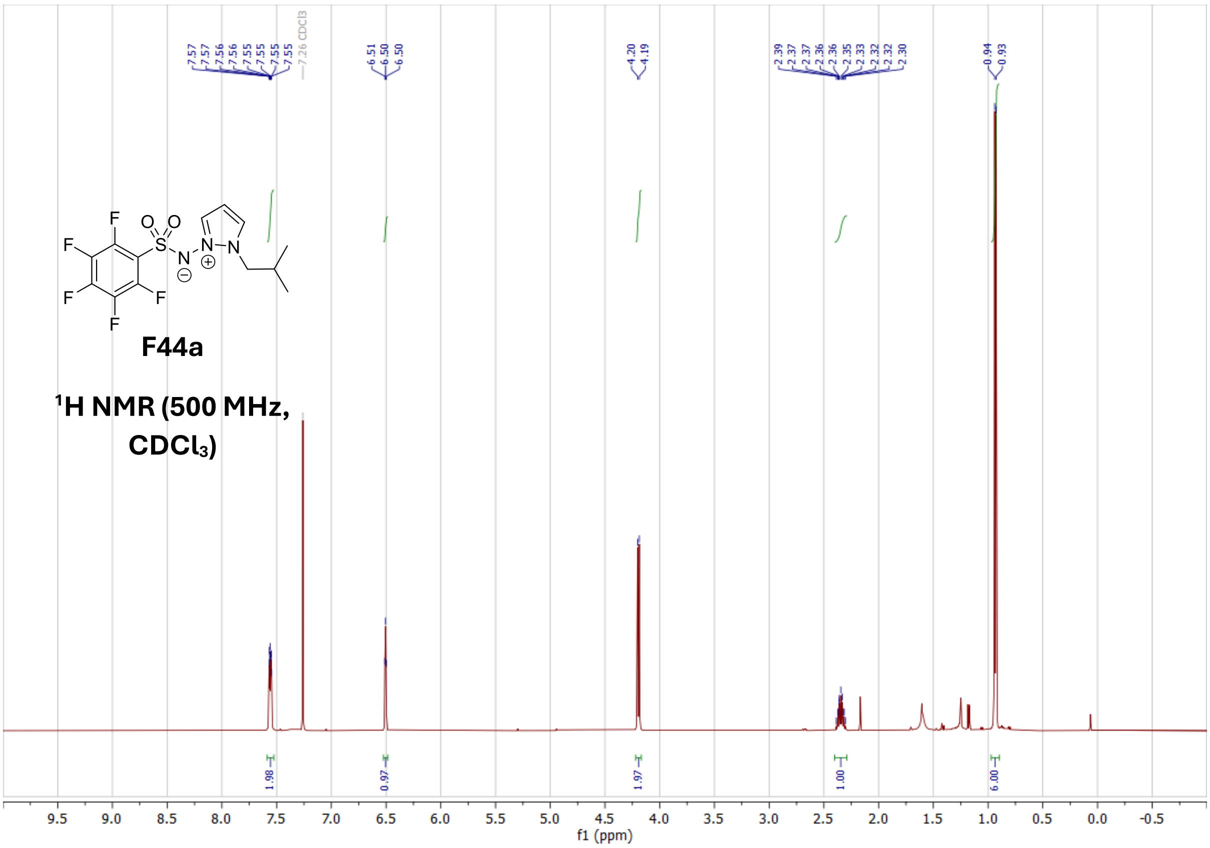
F44a
1H NMR (500 MHz, CDCl3)
f1 (ppm)

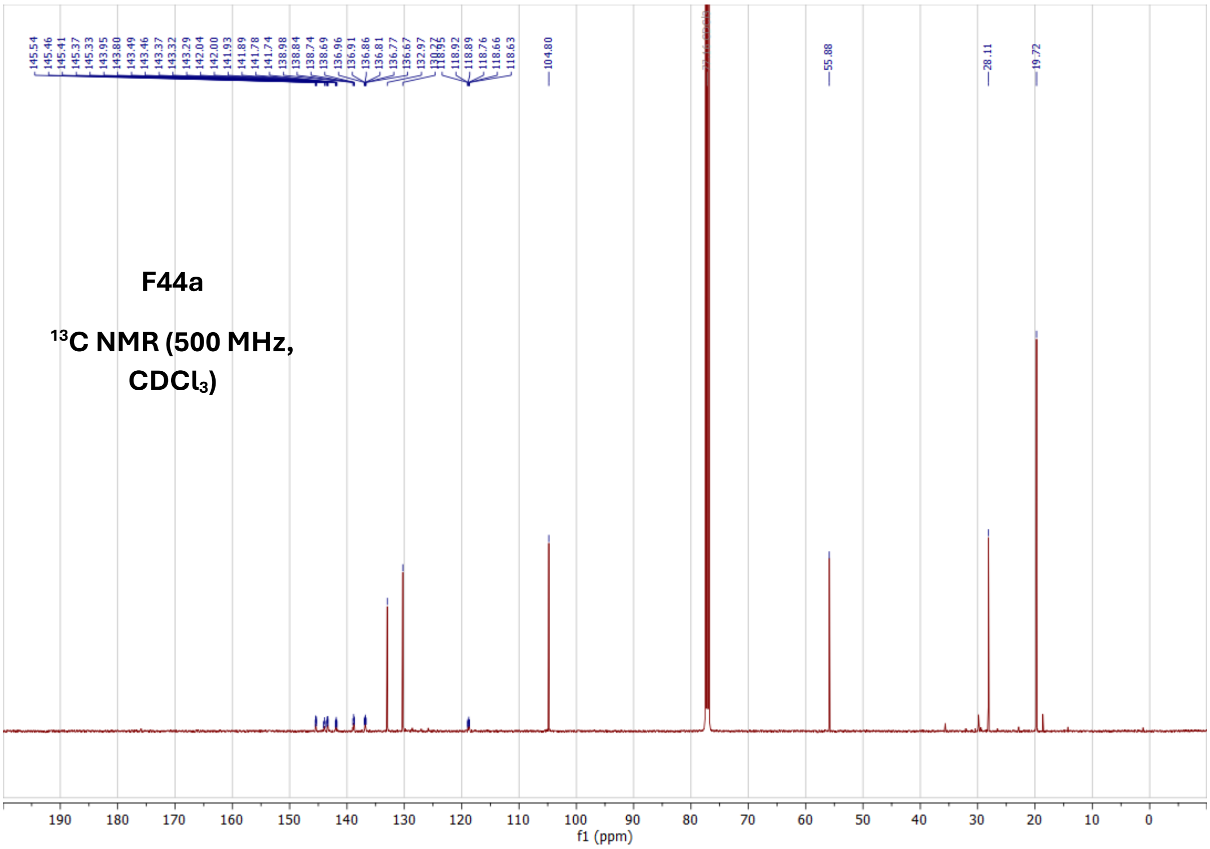
F44a
13C NMR (500 MHz, CDCl3)
f1 (ppm)

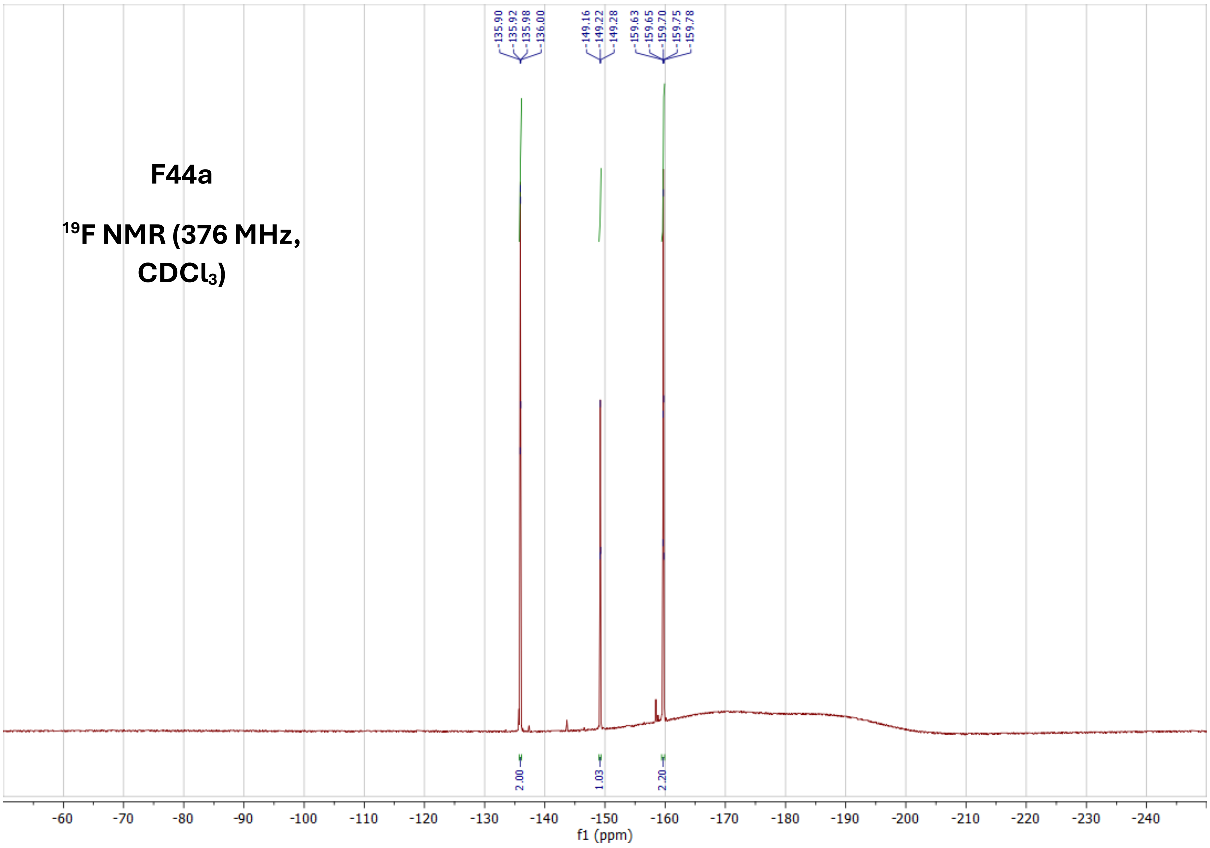
F44a
19F NMR (376 MHz, CDCl3)
-135.90
-135.92
-135.98
-136.00
-149.16
-149.22
-149.28
-159.63
-159.65
-159.70
-159.75
-159.78
2.00
1.03
2.20
-60
-70
-80
-90
-100
-110
-120
-130
-140
-150
-160
-170
-180
-190
-200
-210
-220
-230
-240
f1 (ppm)

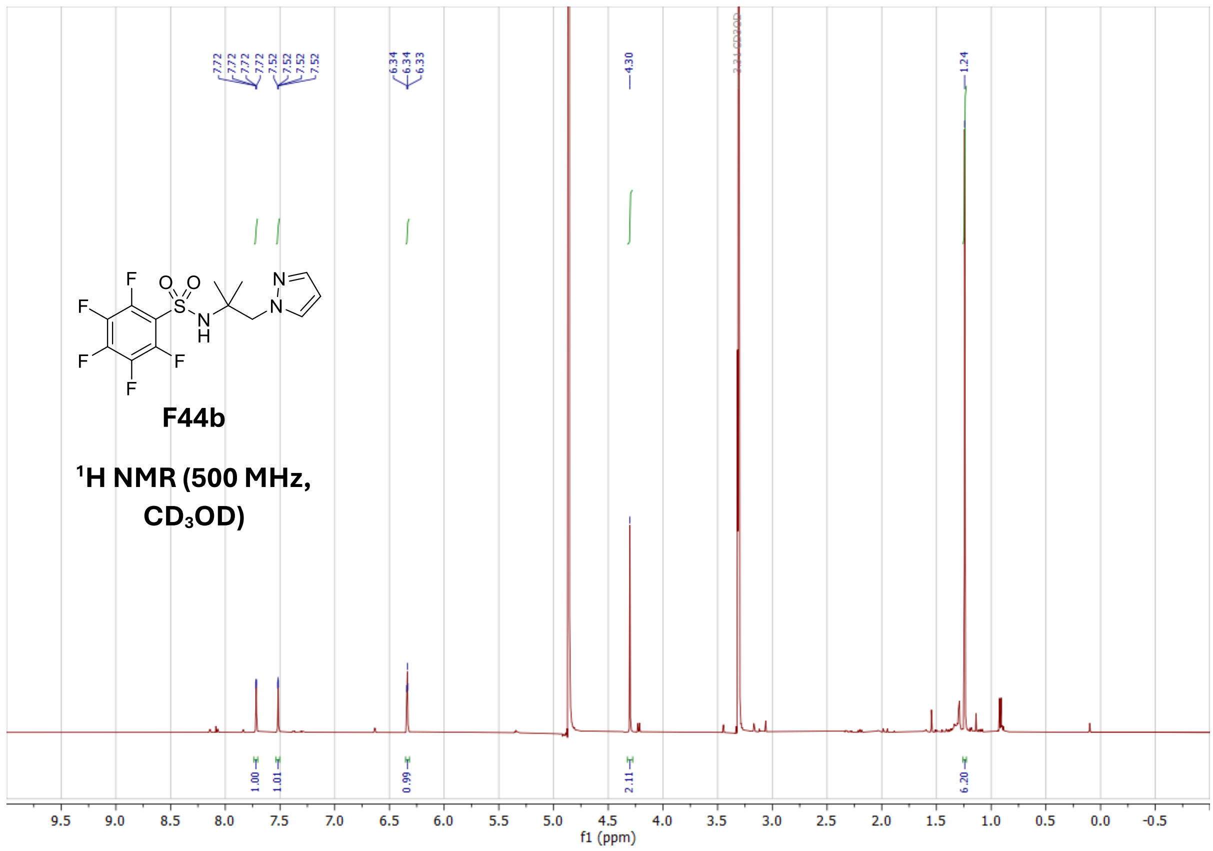
7.72
7.72
7.72
7.72
7.52
7.52
7.52
7.52
6.34
6.34
6.33
4.30
1.24
F44b
¹H NMR (500 MHz, CD₃OD)
1.00
1.01
0.99
2.11
6.20
f1 (ppm)

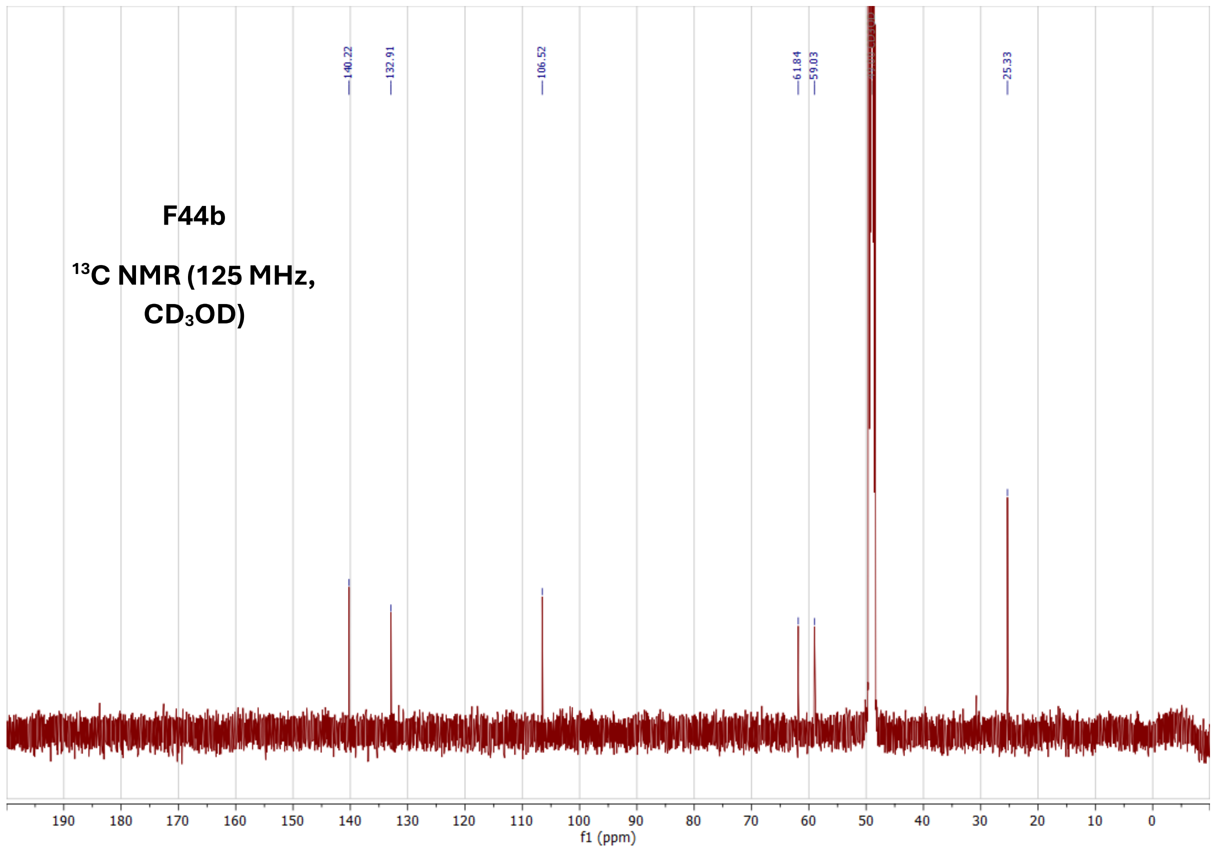
140.22
132.91
106.52
61.84
59.03
25.33
F44b
¹³C NMR (125 MHz, CD₃OD)
f1 (ppm)

F44b

^{19}F NMR (376 MHz, CD_3OD)

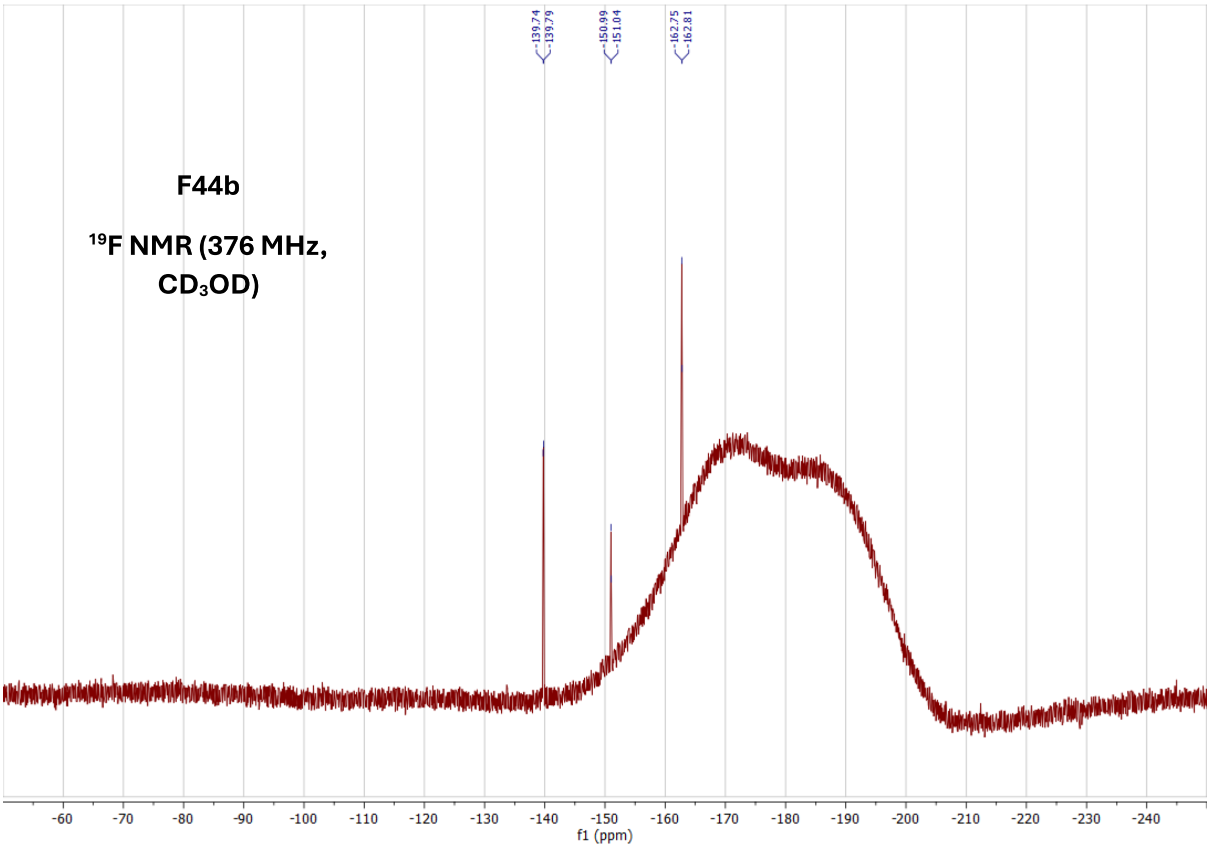

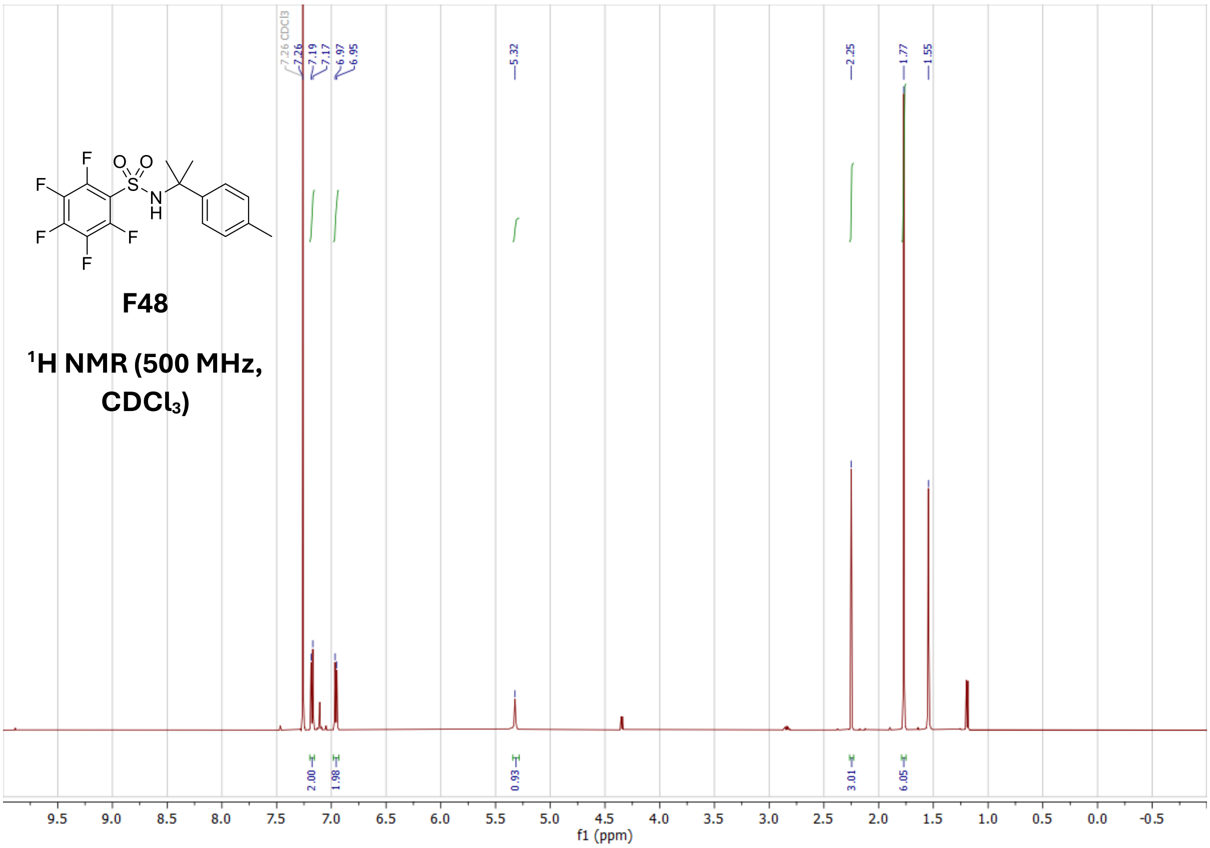
F48
^{1}H NMR (500 MHz, CDCl$_3$)
7.26 CDCl3
7.26
7.19
7.17
6.97
6.95
5.32
2.25
1.77
1.55
2.00
1.98
0.93
3.01
6.05
f1 (ppm)

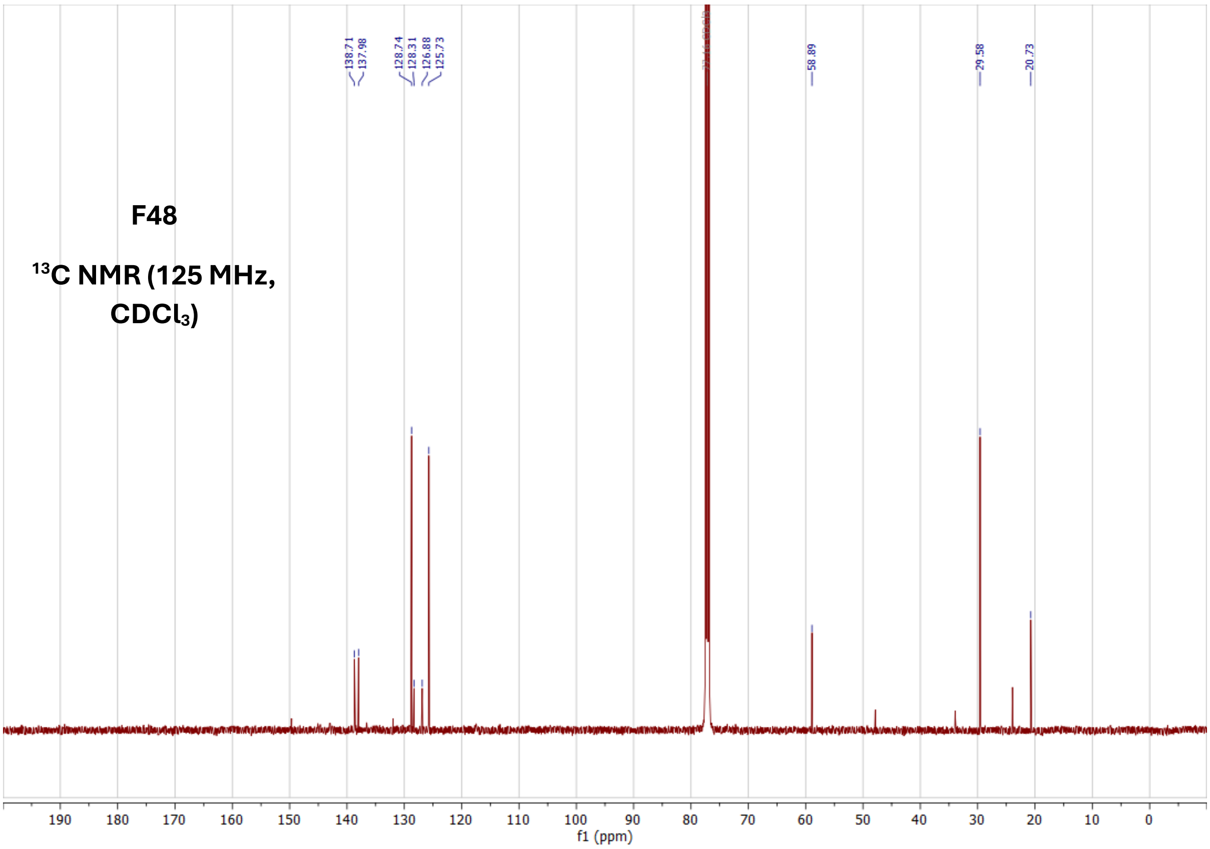
F48
^{13}C NMR (125 MHz, CDCl$_3$)
138.71
137.98
128.74
128.31
126.88
125.73
58.89
29.58
20.73
f1 (ppm)

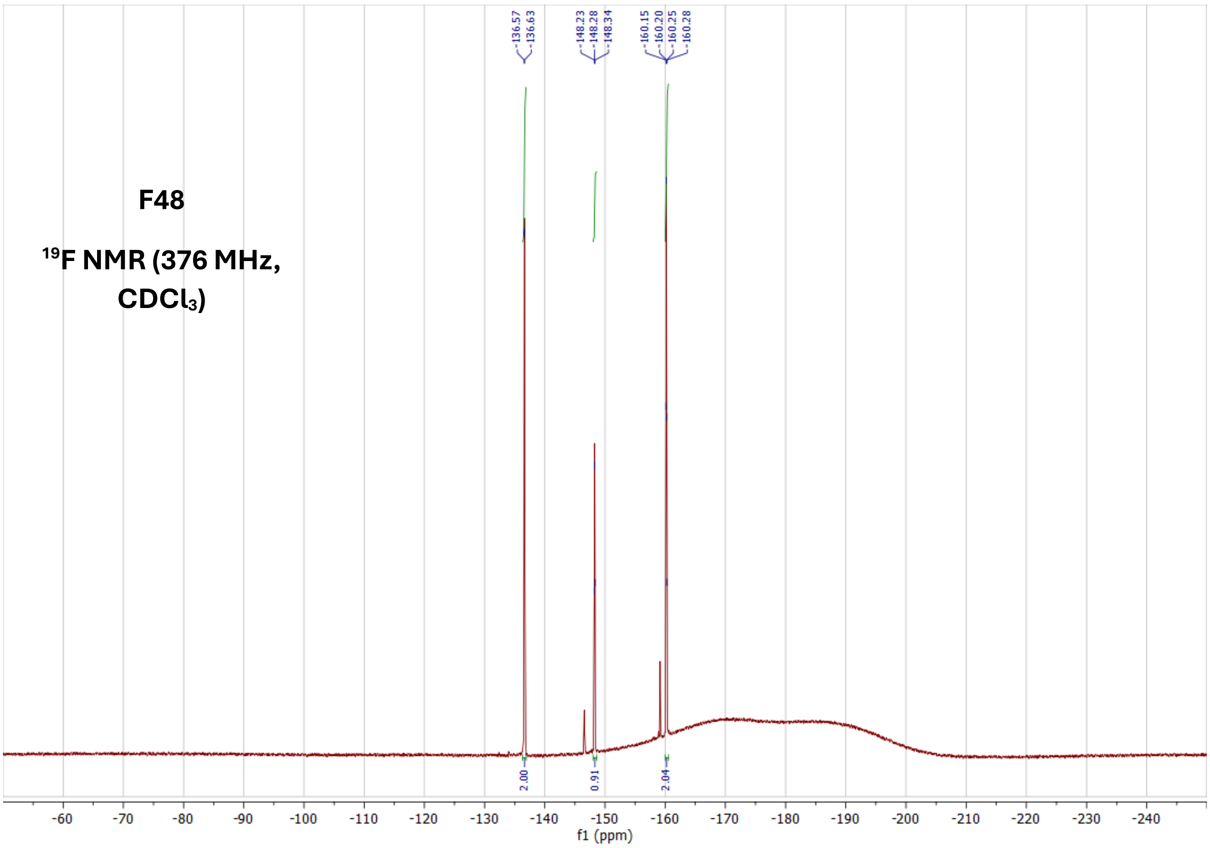
F48
19F NMR (376 MHz, CDCl3)
-136.57
-136.63
-148.23
-148.28
-148.34
-160.15
-160.20
-160.25
-160.28
2.00
0.91
2.04
-60 -70 -80 -90 -100 -110 -120 -130 -140 -150 -160 -170 -180 -190 -200 -210 -220 -230 -240
f1 (ppm)

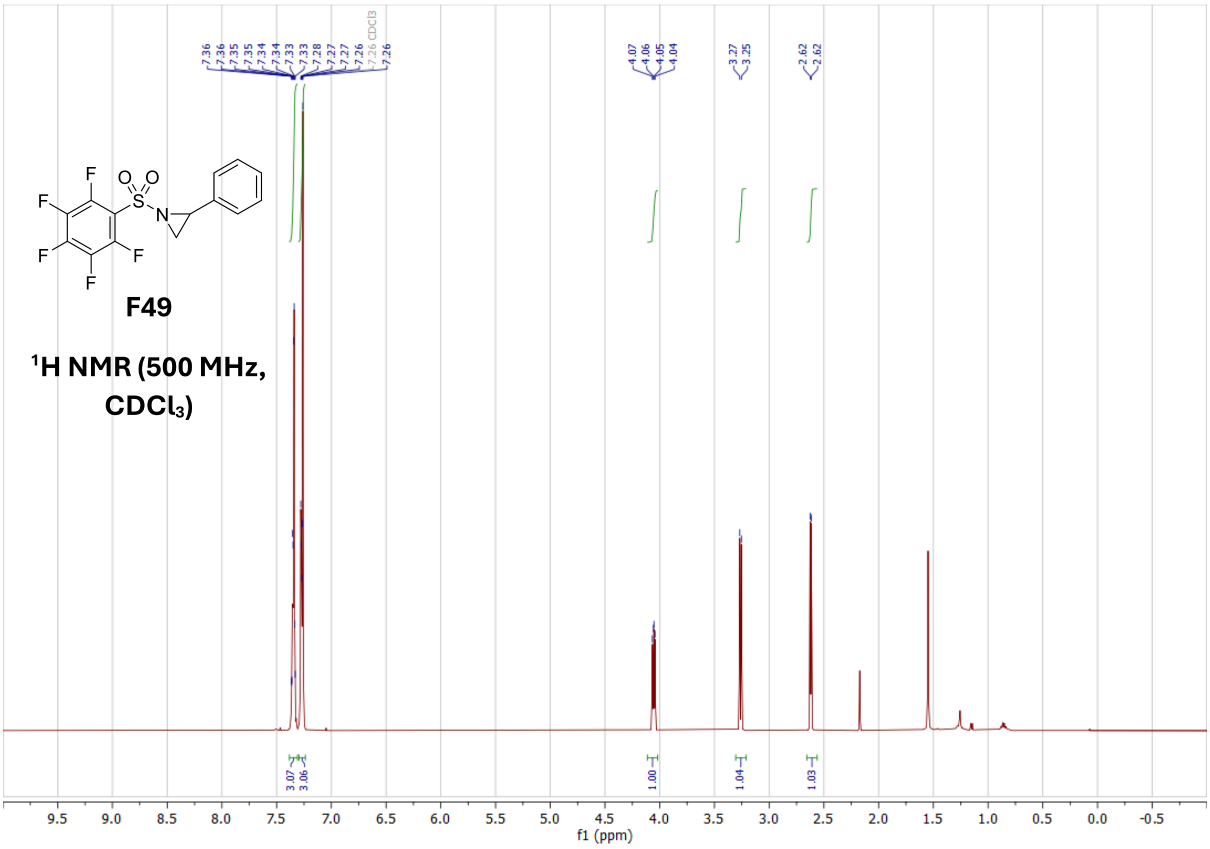

F49

^{1}H NMR (500 MHz, CDCl$_3$)

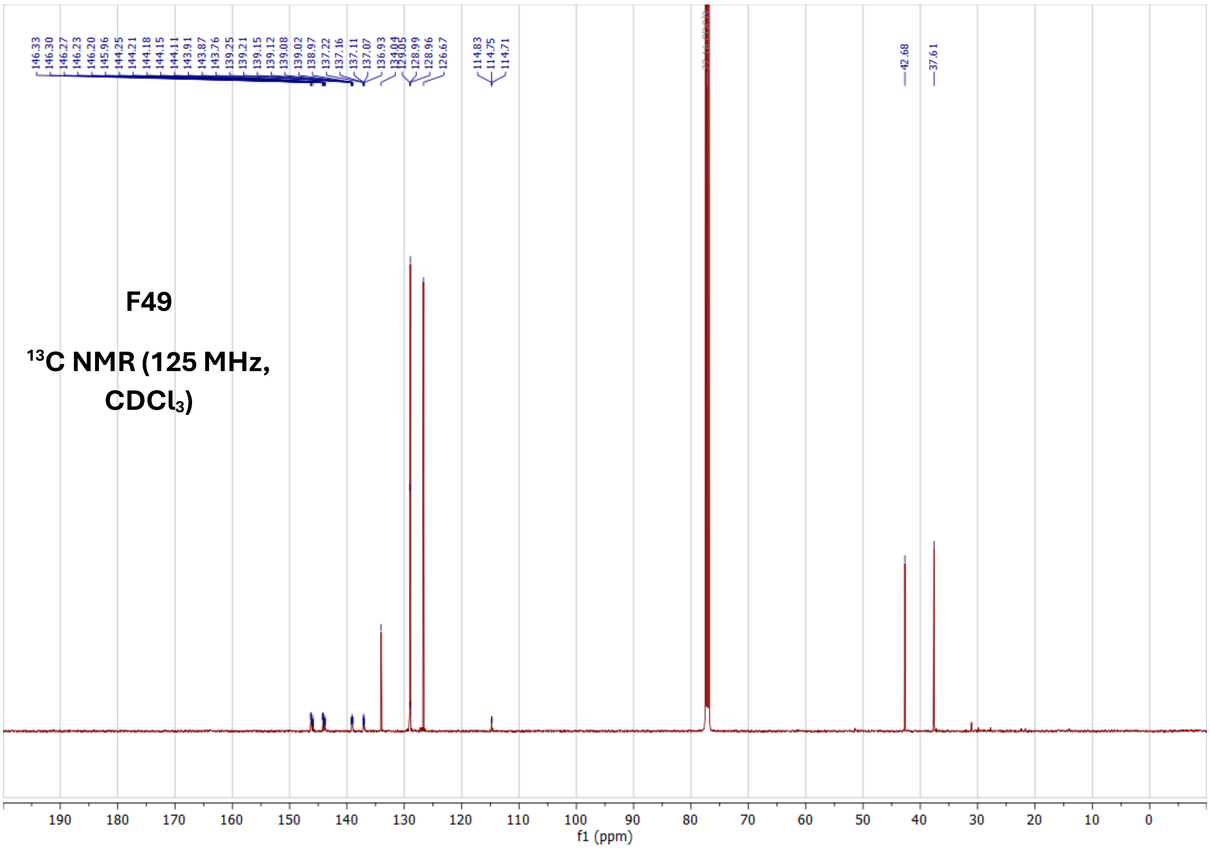

F49

^{13}C NMR (125 MHz, CDCl$_3$)

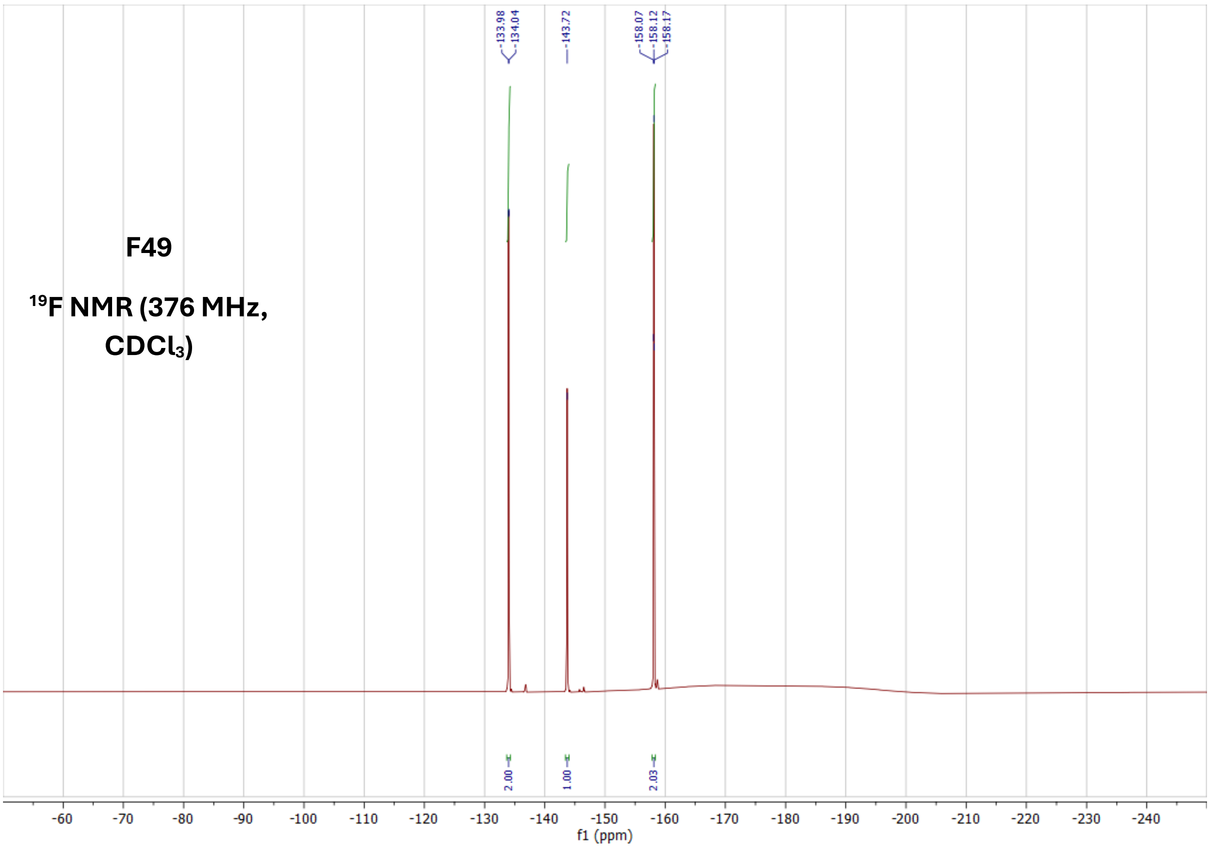

F49
^{19}F NMR (376 MHz, $CDCl_3$)
-133.98
-134.04
-143.72
-158.07
-158.12
-158.17
2.00
1.00
2.03
-60
-70
-80
-90
-100
-110
-120
-130
-140
-150
-160
-170
-180
-190
-200
-210
-220
-230
-240
f1 (ppm)

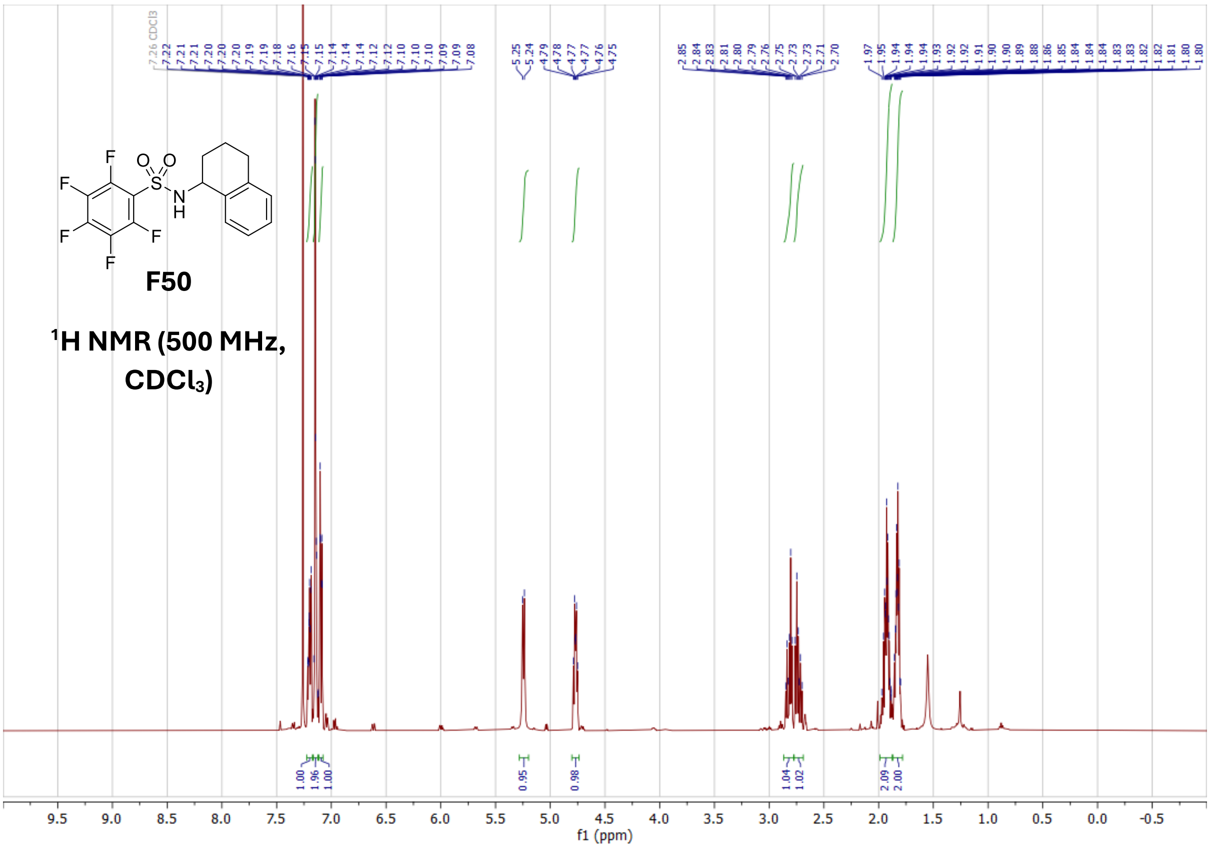

F50

^{1}H NMR (500 MHz, $CDCl_3$)

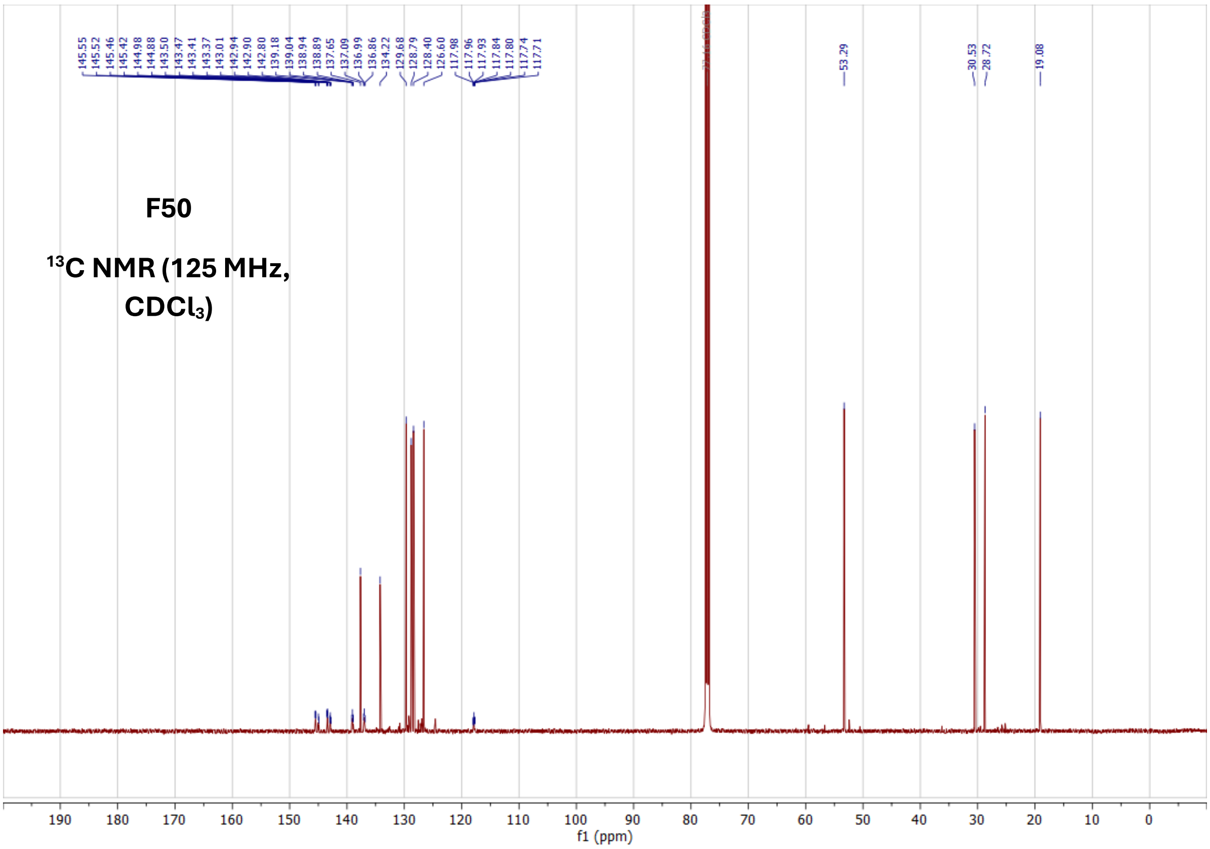

F50

^{13}C NMR (125 MHz, $CDCl_3$)

F50

^{19}F NMR (376 MHz, $CDCl_3$)

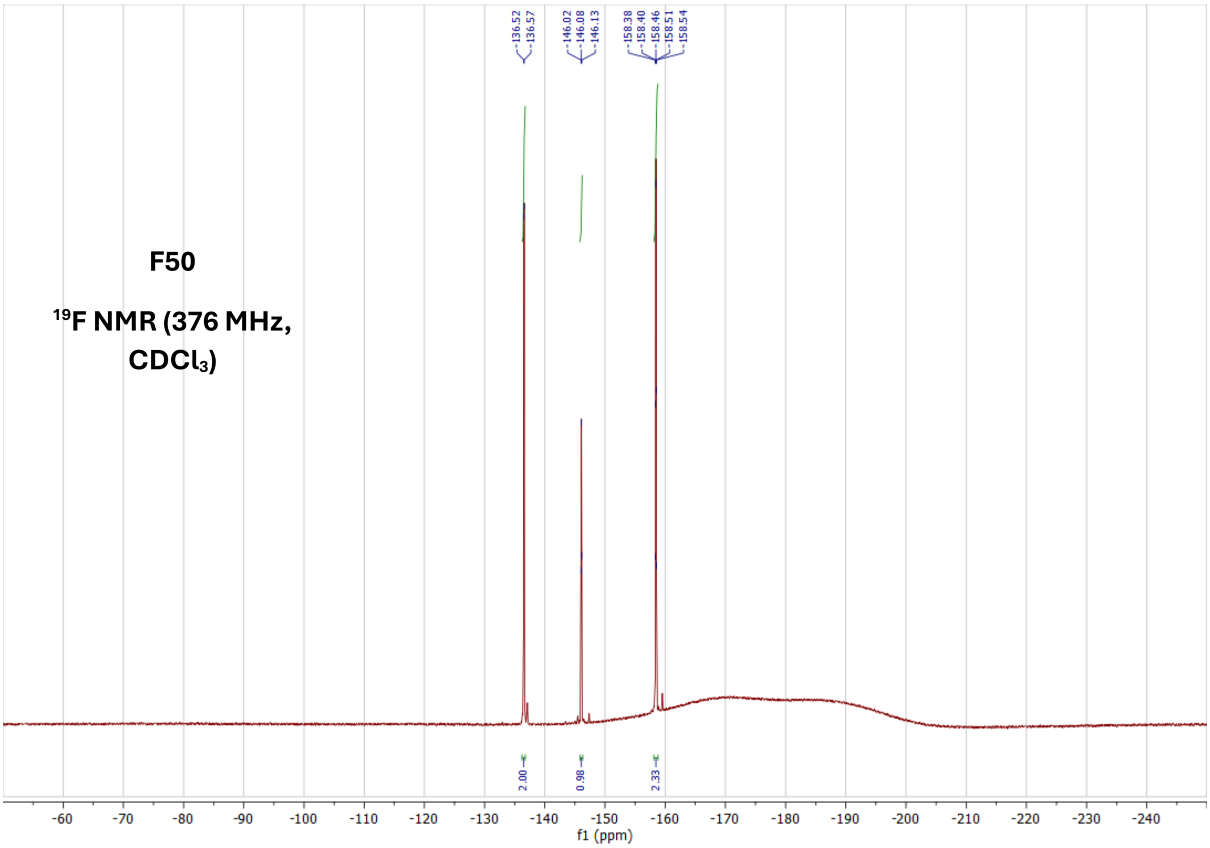

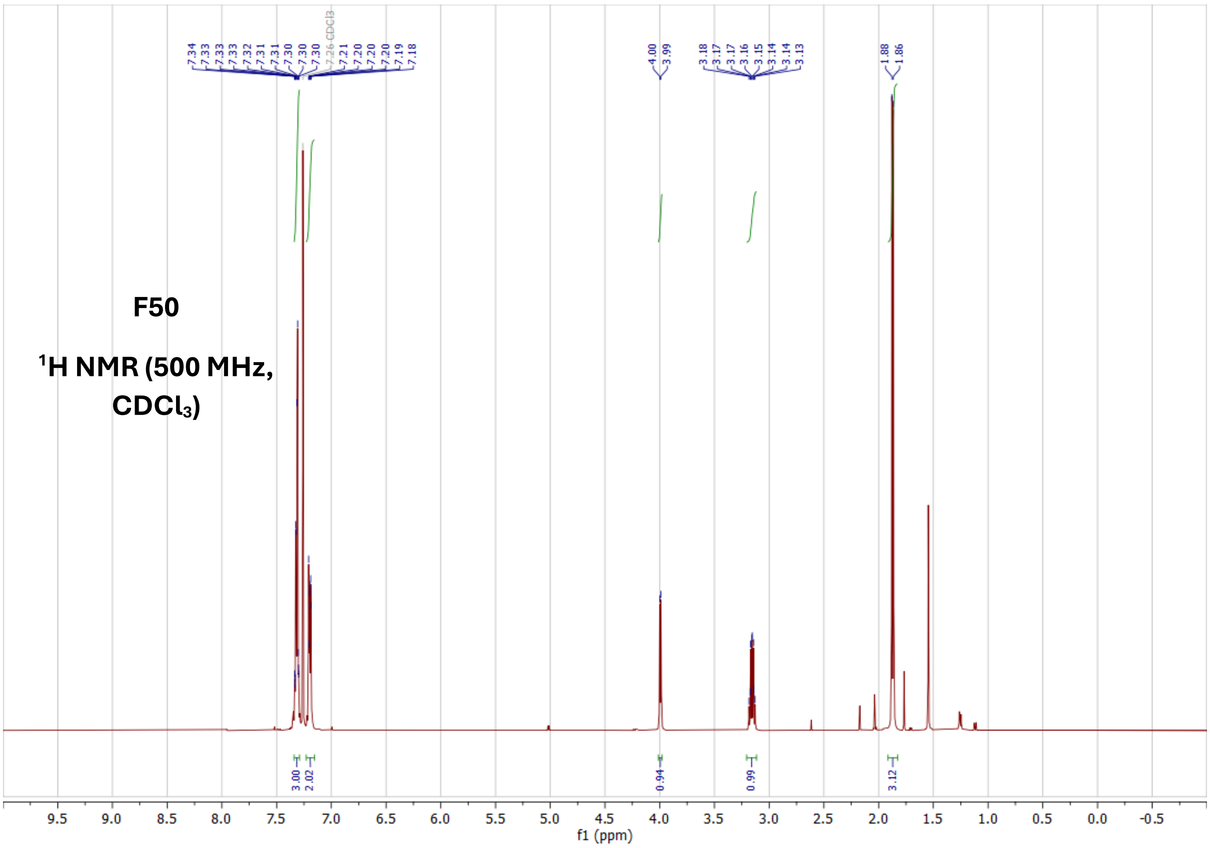
F50
^{1}H NMR (500 MHz, CDCl$_3$)
f1 (ppm)

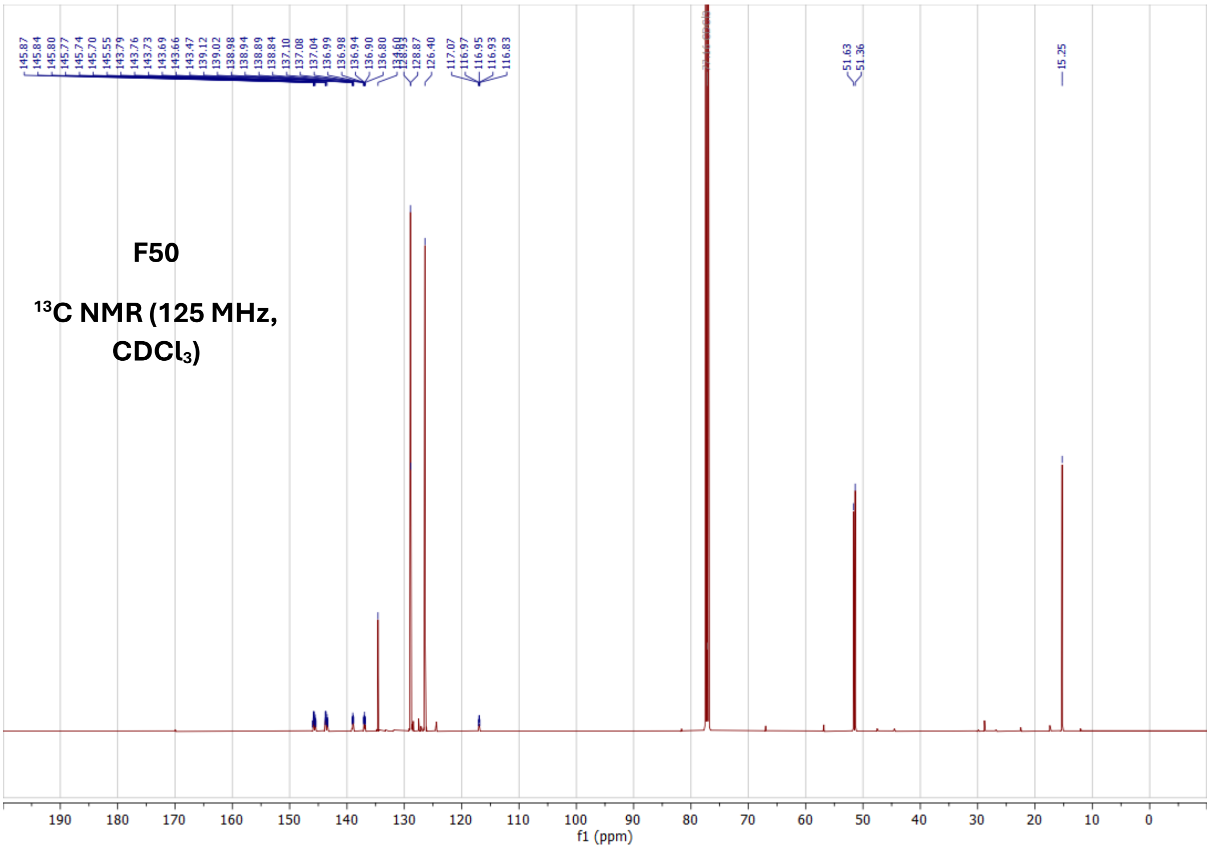
F50
^{13}C NMR (125 MHz, CDCl$_3$)
f1 (ppm)

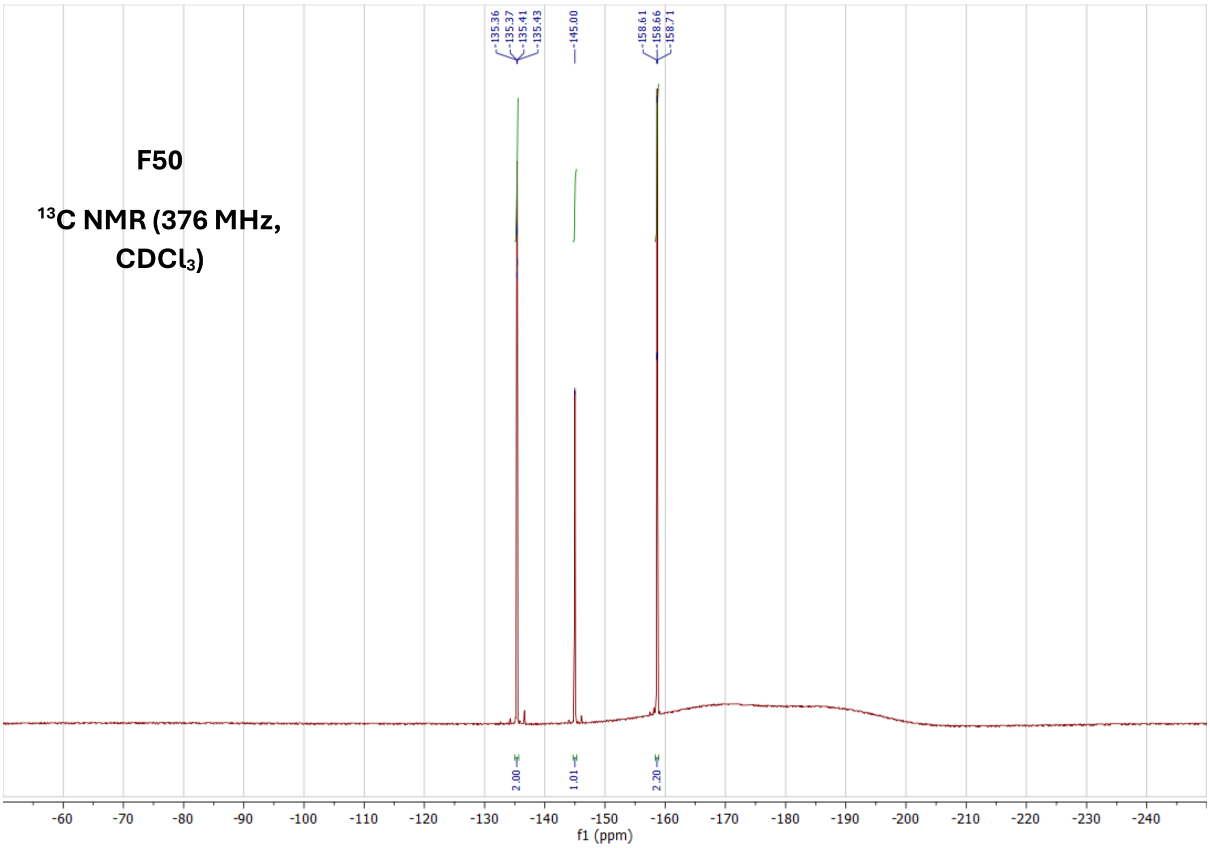

5. References

[1] M. Broncel, R. A. Serwa, P. Ciepla, E. Krause, M. J. Dallman, A. I. Magee and E. W. Tate, Multifunctional reagents for quantitative proteome-wide analysis of protein modification in human cells and dynamic profiling of protein lipidation during vertebrate development, *Angew. Chemie - Int. Ed.*, 2015, **54**, 5948–5951.

[2] M. T. Marty, A. J. Baldwin, E. G. Marklund, G. K. A. Hochberg, J. L. P. Benesch and C. V. Robinson, Bayesian Deconvolution of Mass and Ion Mobility Spectra: From Binary Interactions to Polydisperse Ensembles, *Anal. Chem.*, 2015, **87**, 4370–4376.

[3] M. C. Chambers, B. Maclean, R. Burke, D. Amodei, D. L. Ruderman, S. Neumann, L. Gatto, B. Fischer, B. Pratt, J. Egertson, K. Hoff, D. Kessner, N. Tasman, N. Shulman, B. Frewen, T. A. Baker, M.-Y. Brusniak, C. Paulse, D. Creasy, L. Flashner, K. Kani, C. Moulding, S. L. Seymour, L. M. Nuwaysir, B. Lefebvre, F. Kuhlmann, J. Roark, P. Rainer, S. Detlev, T. Hemenway, A. Huhmer, J. Langridge, B. Connolly, T. Chadick, K. Holly, J. Eckels, E. W. Deutsch, R. L. Moritz, J. E. Katz, D. B. Agus, M. MacCoss, D. L. Tabb and P. Mallick, A cross-platform toolkit for mass spectrometry and proteomics, *Nat. Biotechnol.*, 2012, **30**, 918–920.

[4] G. Laudadio, E. Barmpoutsis, C. Schotten, L. Struik, S. Govaerts, D. L. Browne and T. Noël, Sulfonamide Synthesis through Electrochemical Oxidative Coupling of Amines and Thiols, J. Am. Chem. Soc., 2019, 141, 5664–5668.

[5] G. Laudadio, E. Barmpoutsis, C. Schotten, L. Struik, S. Govaerts, D. L. Browne and T. Noël, Sulfonamide Synthesis through Electrochemical Oxidative Coupling of Amines and Thiols, *J. Am. Chem. Soc.*, 2019, **141**, 5664–5668.
